# Supplementary material for: Direct and indirect neurogenesis from radial glial progenitor cell clones in the mouse neocortex
Source: EMBO J. 2025 Nov 20;45(1):182–209. doi: 10.1038/s44318-025-00624-9 (PMC12759082; doi:10.1038/s44318-025-00624-9)
Supplement: Supplementary file 1 — Table EV1 [file 44318_2025_624_MOESM1_ESM.docx]

**Table EV1. Spatial distances between neurons in Fig. 1F.**

| **Pairwise** | **Distance (μm)** |
| --- | --- |
| neuron1-neuron2 | 354.15 |
| neuron1-neuron3 | 408.01 |
| neuron1-neuron4 | 256.35 |
| neuron1-neuron5 | 351.12 |
| neuron1-neuron6 | 310.33 |
| neuron1-neuron7 | 510.04 |
| neuron2-neuron3 | 63.55 |
| neuron2-neuron4 | 198.86 |
| neuron2-neuron5 | 93.86 |
| neuron2-neuron6 | 145.13 |
| neuron2-neuron7 | 227.31 |
| neuron3-neuron4 | 230.82 |
| neuron3-neuron5 | 124.96 |
| neuron3-neuron6 | 193.99 |
| neuron3-neuron7 | 192.13 |
| neuron4-neuron5 | 251.61 |
| neuron4-neuron6 | 245.59 |
| neuron4-neuron7 | 305.73 |
| neuron5-neuron6 | 104.45 |
| neuron5-neuron7 | 310.27 |
| neuron6-neuron7 | 359.82 |
