# Supplementary material for: Direct and indirect neurogenesis from radial glial progenitor cell clones in the mouse neocortex
Source: EMBO J. 2025 Nov 20;45(1):182–209. doi: 10.1038/s44318-025-00624-9 (PMC12759082; doi:10.1038/s44318-025-00624-9)
Supplement: Supplementary file 2 — Table EV2 [file 44318_2025_624_MOESM2_ESM.docx]

**Table EV2. Lineage features and feature descriptions related to hierarchical clustering in Fig. 2A.**

| **Features** | **Feature description** |
| --- | --- |
| **IPP division in G1** | Whether the first generation is IPP division. |
| **IPP division in G2** | Whether the second generation is IPP division. |
| **IPP division in G3** | Whether the third generation is IPP division. |
| **IPP division in G4** | Whether the fourth generation is IPP division. |
| **IPP division in G5** | Whether the fifth generation is IPP division. |
| **Neurons from N divisions** | RGP-generated neuron numbers in a clone, including apoptosis neurons. |
| **Neurons from IP divisions** | IP-generated neuron numbers in a clone, including apoptosis neurons. |
| **Neurons from IPP divisions** | IPP-generated neuron numbers in a clone, including apoptosis neurons. |
| **CThPN number** | Number of CThPN generated in a clone. |
| **SCPN number** | Number of SCPN generated in a clone. |
| **HPN number** | Number of HPN generated in a clone. |
| **CPN number** | Number of CPN generated in a clone. |
| **Pure CThPN lineage** | Only CThPN is generated in a clone. |
| **Pure SCPN lineage** | Only SCPN is generated in a clone. |
| **Pure CPN lineage** | Only CPN is generated in a clone. |
| **Neuron number in L6** | The excitatory neuron number distributed in L6 in a clone. |
| **Neuron number in L5** | The excitatory neuron number distributed in L5 in a clone. |
| **Neuron number in L4** | The excitatory neuron number distributed in L4 in a clone. |
| **Neuron number in L2/3** | The excitatory neuron number distributed in L2/3 in a clone. |
| **Layer distribution** | Description of layer distribution of clones.  Clones were recorded as 0, when neurons in a clone distributed in a distinct layer (L2/3, L4, L5, L6). Clones were recorded as 1, when neurons were distributed in deep layers (L5, L6). Clones were recorded as 2, when neurons were distributed in superficial layers (L2/3, L4). Clones were recorded as 3, when neurons were distributed both in deep layer and superficial layers. |
