# Supplementary material for: Direct and indirect neurogenesis from radial glial progenitor cell clones in the mouse neocortex
Source: EMBO J. 2025 Nov 20;45(1):182–209. doi: 10.1038/s44318-025-00624-9 (PMC12759082; doi:10.1038/s44318-025-00624-9)
Supplement: Supplementary file 4 — Movie EV1 [file 44318_2025_624_MOESM4_ESM.zip › Movie EV1/Movie EV1.docx]

**Movie EV1. N division of radial glia progenitor in clone.**

Radial glia progenitor (RGP) undergoes asymmetric division and generates a neuron directly at the ventricular surface during neurogenesis. A RGP is shown first dividing at ventricular (*t* = 0.5 h) surface and then daughter neuron is generated (*t* = 1.0 h). Cyan arrowheads: RGP, Red arrowheads: neuron.
