# Supplementary material for: Direct and indirect neurogenesis from radial glial progenitor cell clones in the mouse neocortex
Source: EMBO J. 2025 Nov 20;45(1):182–209. doi: 10.1038/s44318-025-00624-9 (PMC12759082; doi:10.1038/s44318-025-00624-9)
Supplement: Supplementary file 5 — Movie EV2 [file 44318_2025_624_MOESM5_ESM.zip › Movie EV2/Movie EV2.docx]

**Movie EV2. IP division of radial glia progenitor in clone.**

Radial glia progenitor (RGP) appears in the ventricular surface ready for indirect neurogenesis giving rise to an intermediate progenitor (IP). Two resulting daughter neurons from IP are born at subventricular zone (*t* = 14.0 h) and then migrate. Cyan arrowheads: RGP, yellow arrowheads: IP, red arrowheads: neuron.
