# Supplementary material for: Direct and indirect neurogenesis from radial glial progenitor cell clones in the mouse neocortex
Source: EMBO J. 2025 Nov 20;45(1):182–209. doi: 10.1038/s44318-025-00624-9 (PMC12759082; doi:10.1038/s44318-025-00624-9)
Supplement: Supplementary file 6 — Movie EV3 [file 44318_2025_624_MOESM6_ESM.zip › Movie EV3/Movie EV3.docx]

**Movie EV3. IPP division of radial glia progenitor in clone.**

A proliferative intermediate progenitor (IPP) is generated from a radial glia progenitor (RGP) indirectly (*t* = 0 h). IPP then symmetrically gives rise to two intermediate progenitors (IPs). Neurogenic IPs divide symmetrically in the subventricular zone (SVZ) and eventually give rise to four neurons. Cyan arrowheads: RGP, yellow arrowheads: IP or IPP, red arrowheads: neuron.
