# Supplementary figures and images for: Direct and indirect neurogenesis from radial glial progenitor cell clones in the mouse neocortex (part 1 of 3)
Source: EMBO J. 2025 Nov 20;45(1):182–209. doi: 10.1038/s44318-025-00624-9 (PMC12759082; doi:10.1038/s44318-025-00624-9)

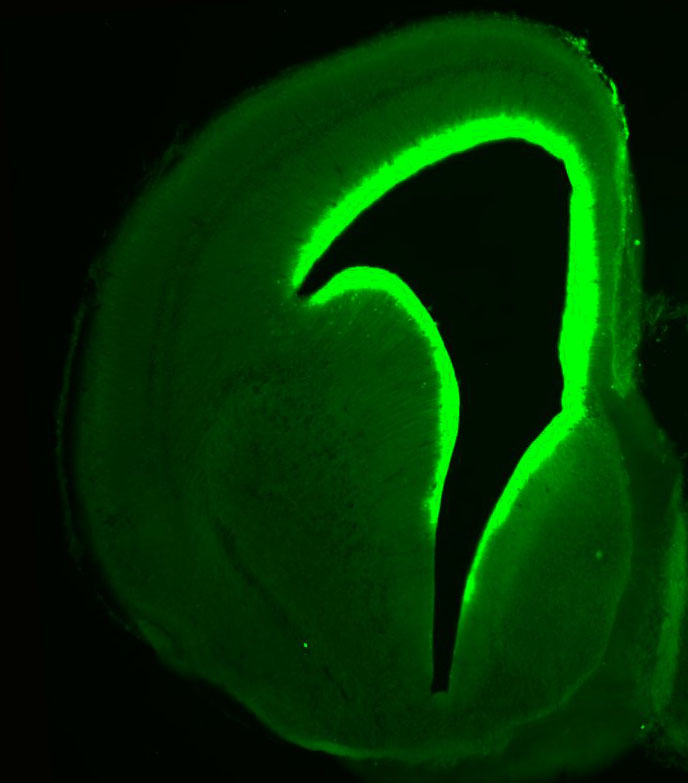

Supplement: Supplementary file 7 — Source data Fig. 1C,D,F [file 44318_2025_624_MOESM7_ESM.zip › Figure. 1C,D,F/1C/1C_left.tif]

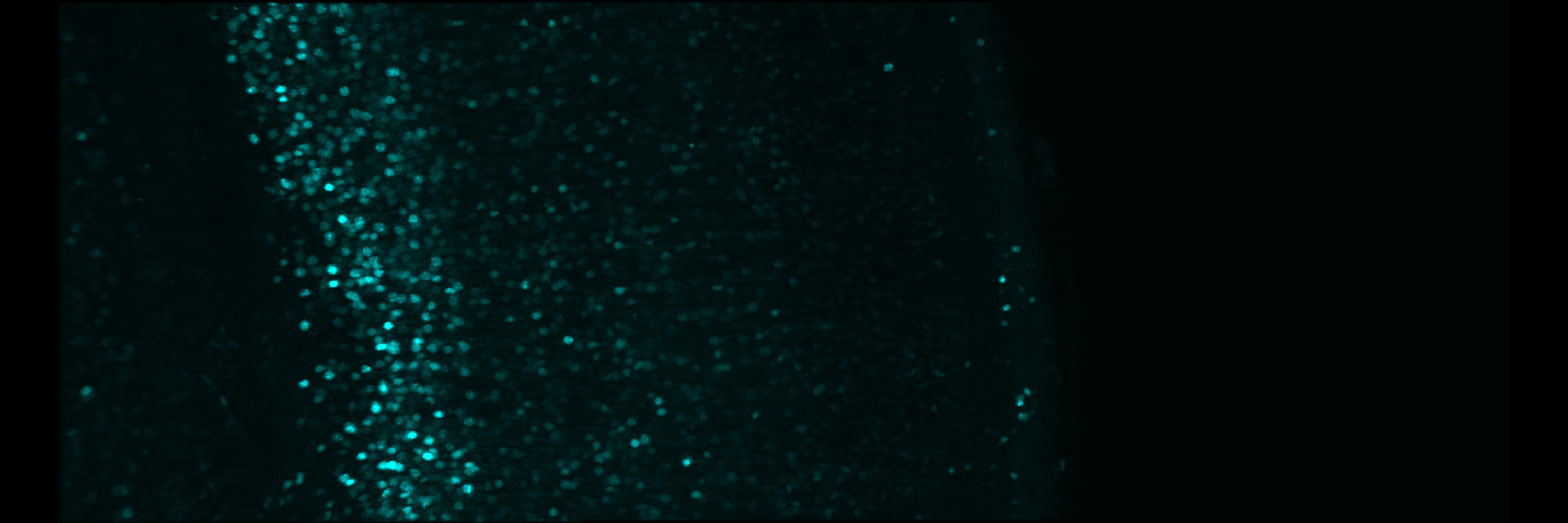

Supplement: Supplementary file 7 — Source data Fig. 1C,D,F [file 44318_2025_624_MOESM7_ESM.zip › Figure. 1C,D,F/1C/1C_right.tif]

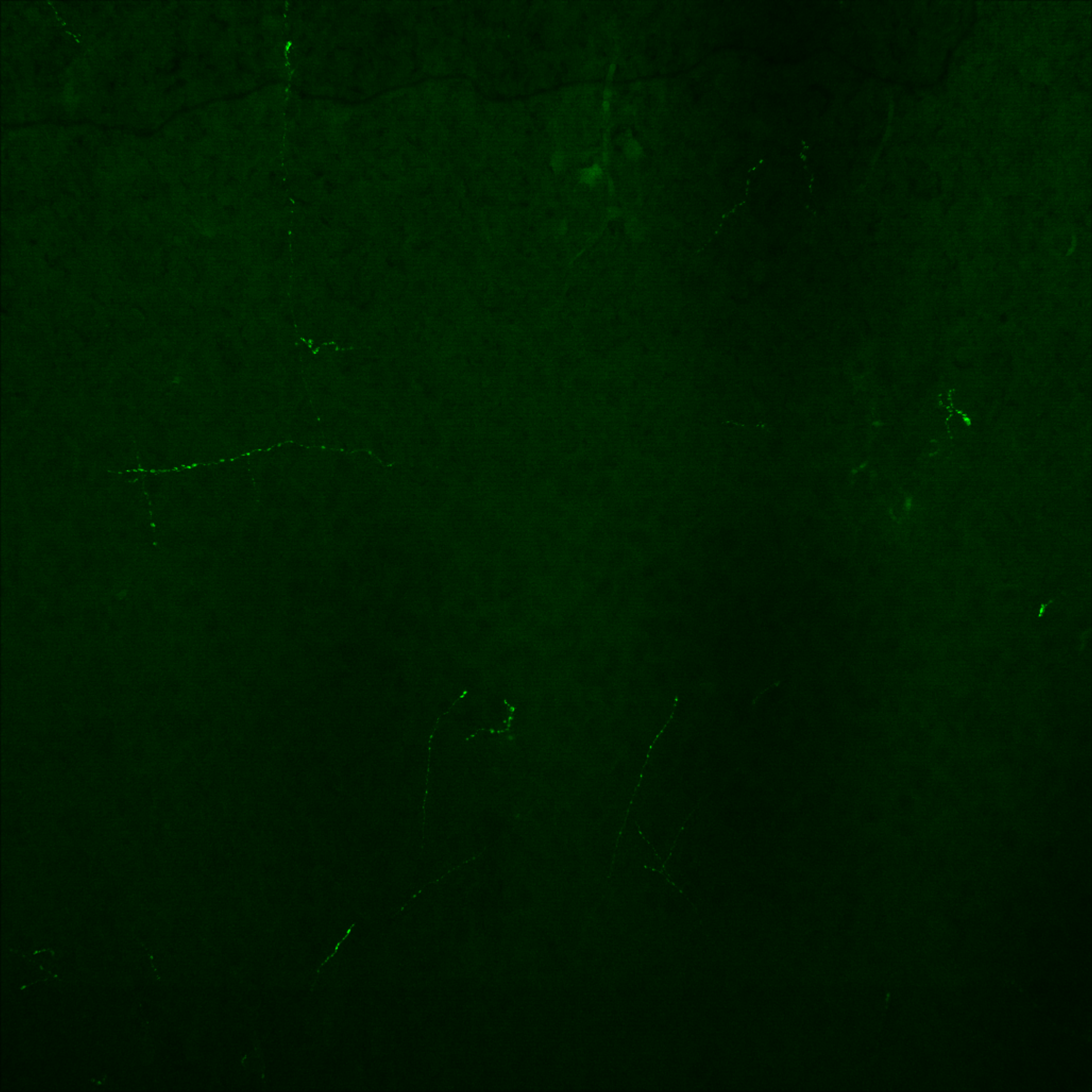

Supplement: Supplementary file 7 — Source data Fig. 1C,D,F [file 44318_2025_624_MOESM7_ESM.zip › Figure. 1C,D,F/1F/N+1/3.tif]

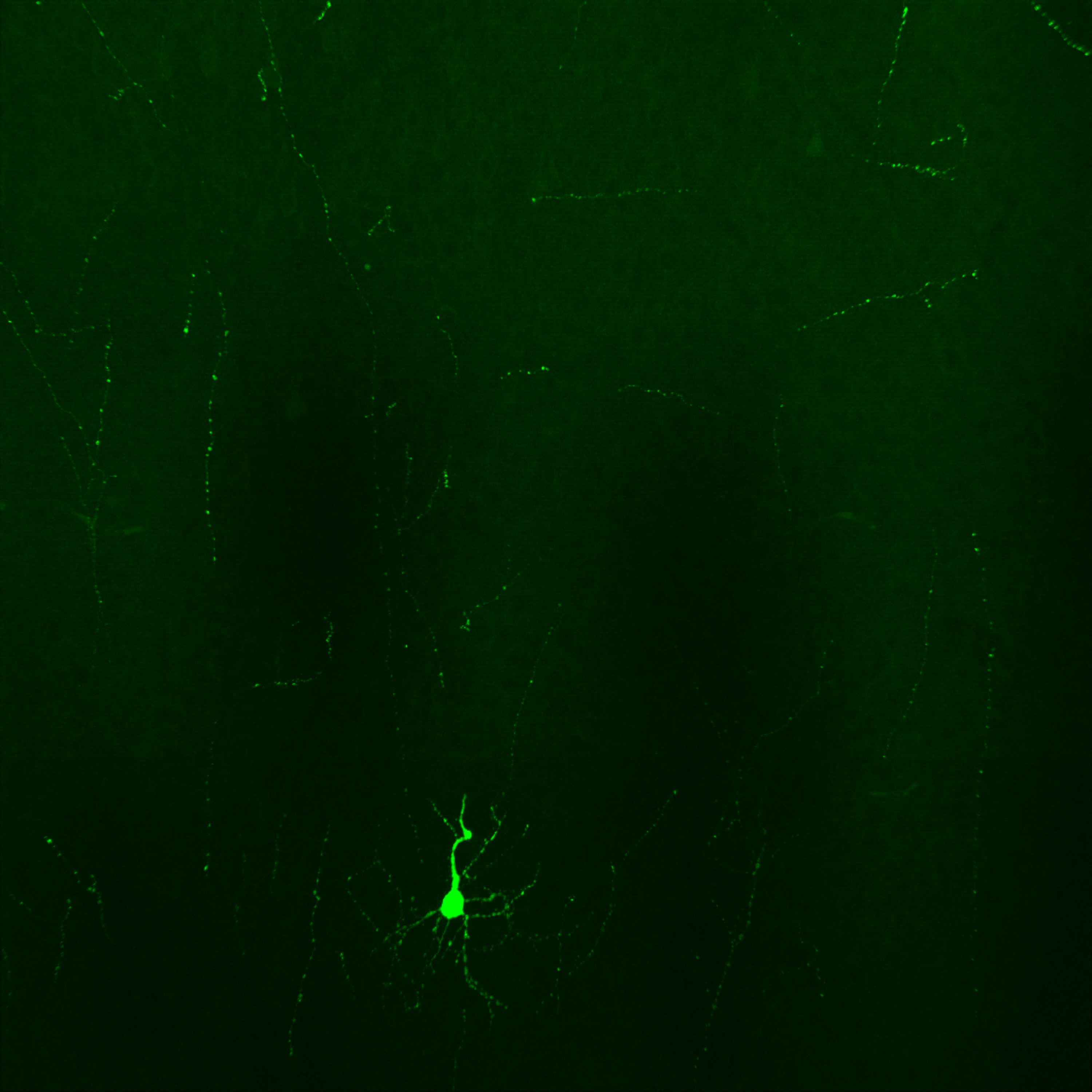

Supplement: Supplementary file 7 — Source data Fig. 1C,D,F [file 44318_2025_624_MOESM7_ESM.zip › Figure. 1C,D,F/1F/N+1/2.tif]

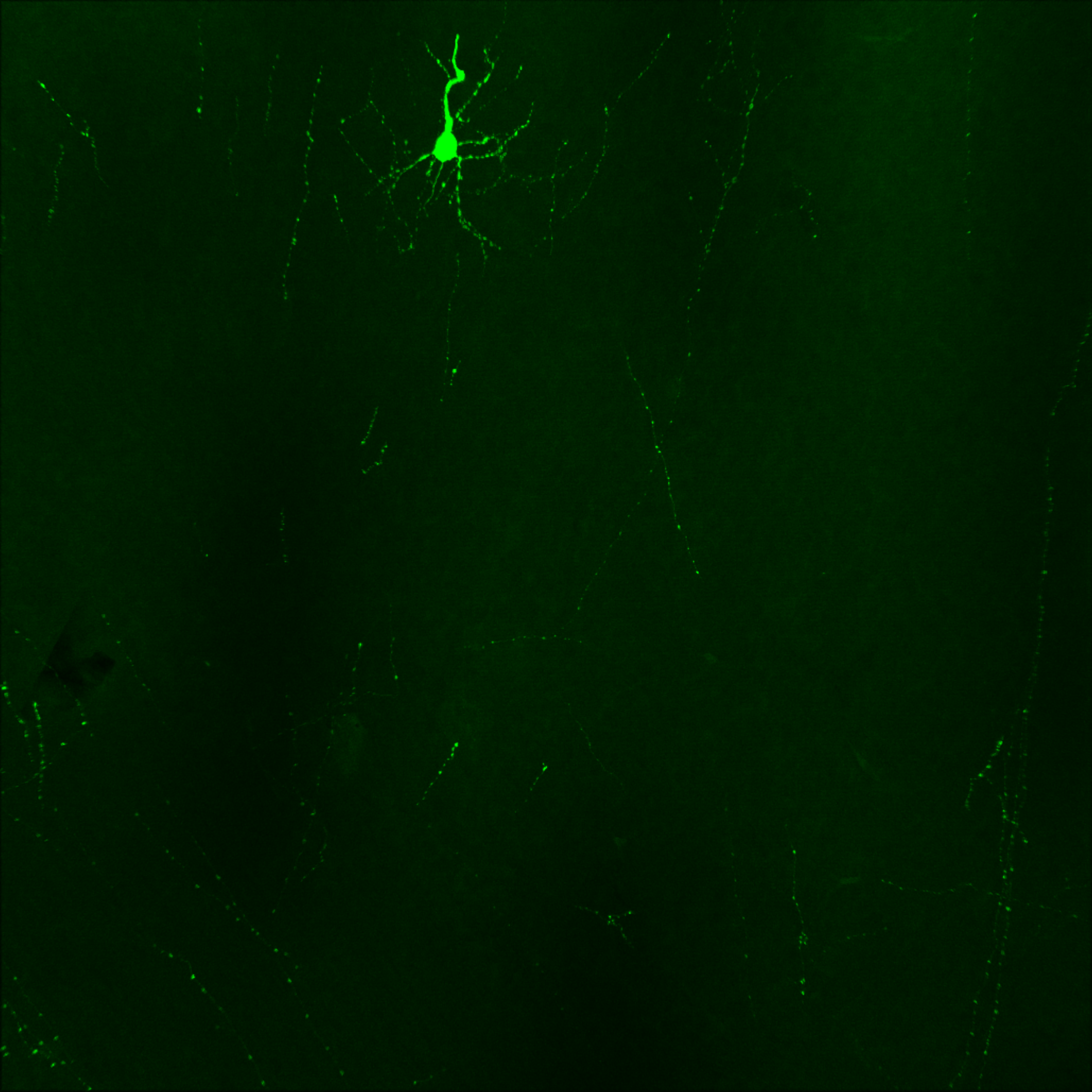

Supplement: Supplementary file 7 — Source data Fig. 1C,D,F [file 44318_2025_624_MOESM7_ESM.zip › Figure. 1C,D,F/1F/N+1/1.tif]

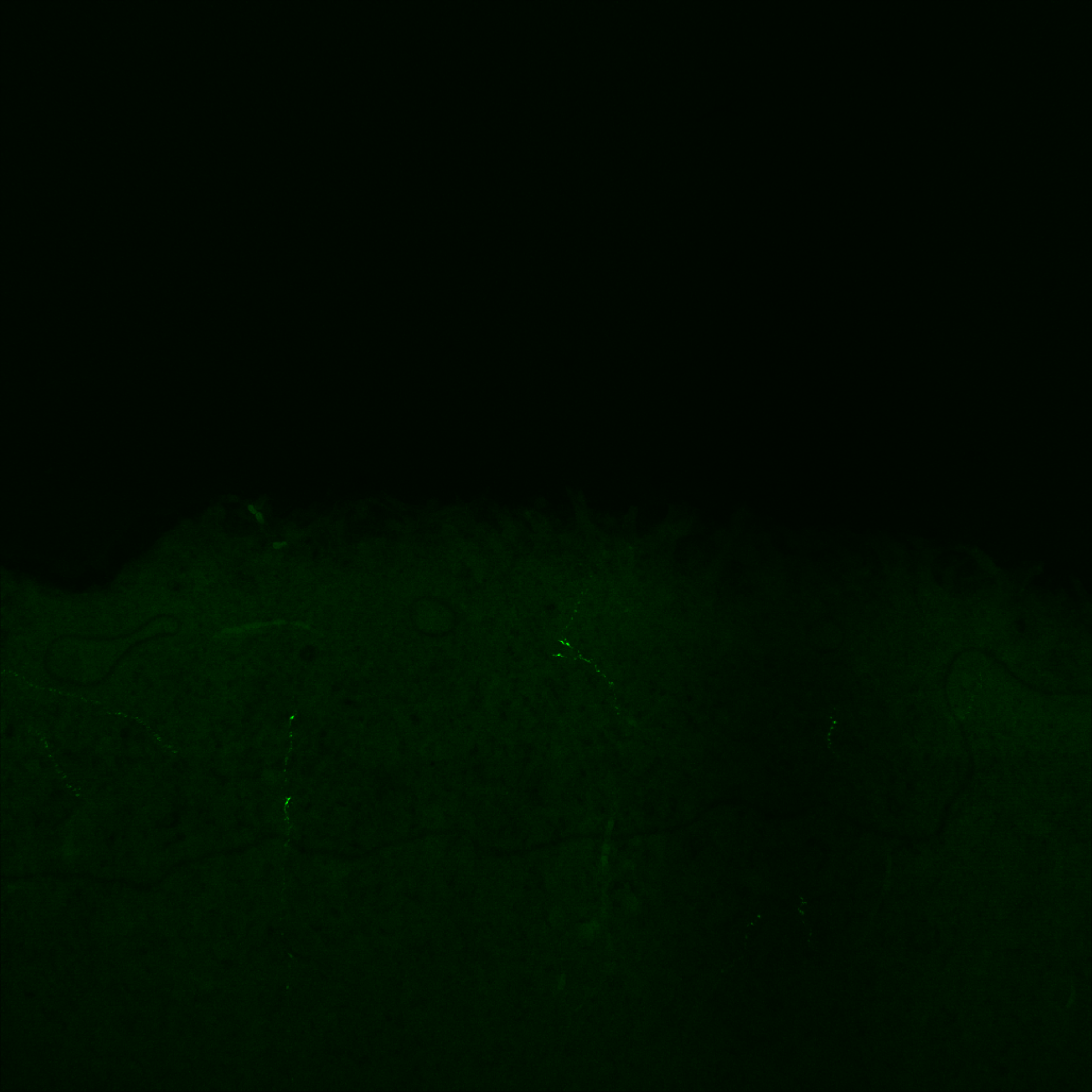

Supplement: Supplementary file 7 — Source data Fig. 1C,D,F [file 44318_2025_624_MOESM7_ESM.zip › Figure. 1C,D,F/1F/N+1/4.tif]

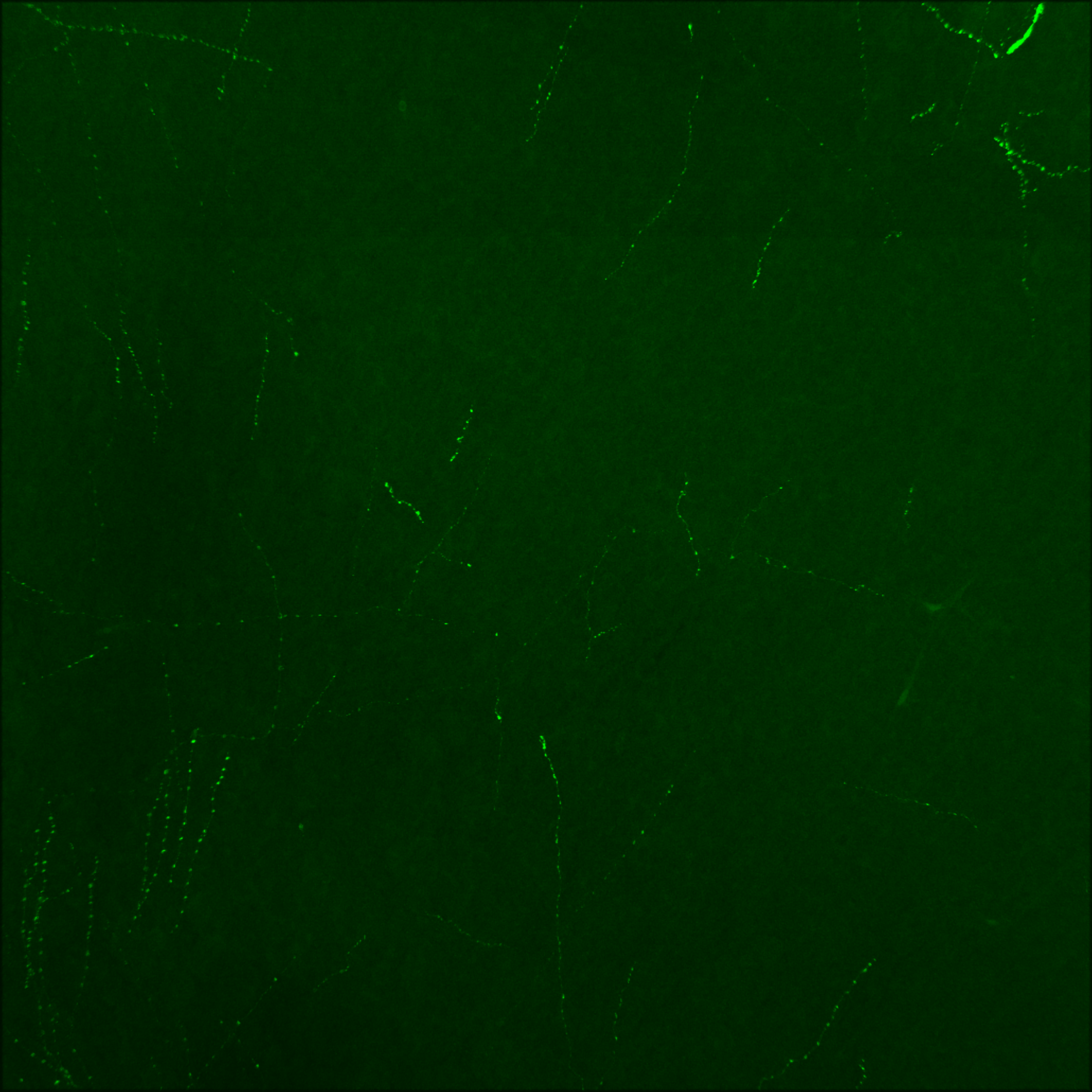

Supplement: Supplementary file 7 — Source data Fig. 1C,D,F [file 44318_2025_624_MOESM7_ESM.zip › Figure. 1C,D,F/1F/N/3.tif]

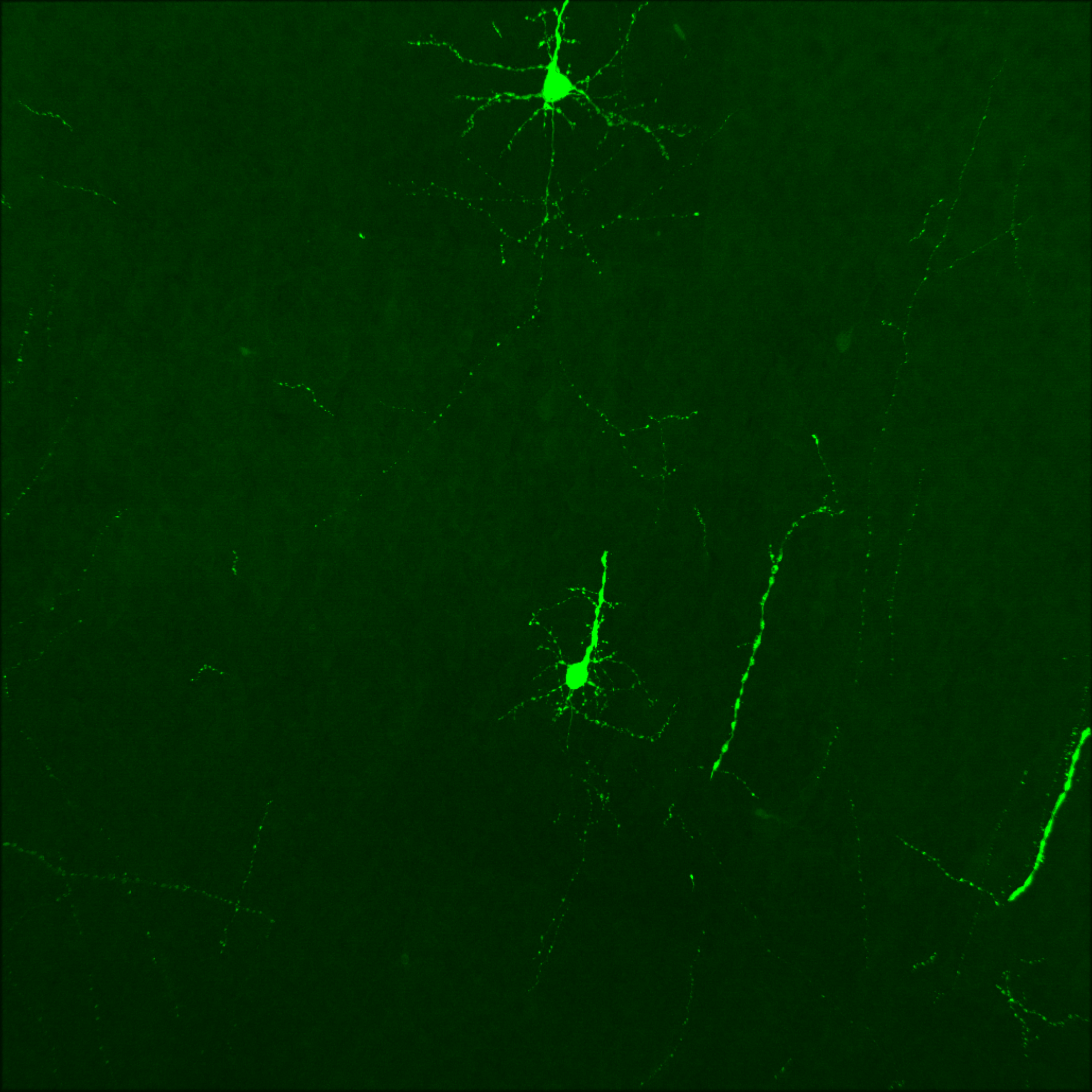

Supplement: Supplementary file 7 — Source data Fig. 1C,D,F [file 44318_2025_624_MOESM7_ESM.zip › Figure. 1C,D,F/1F/N/2.tif]

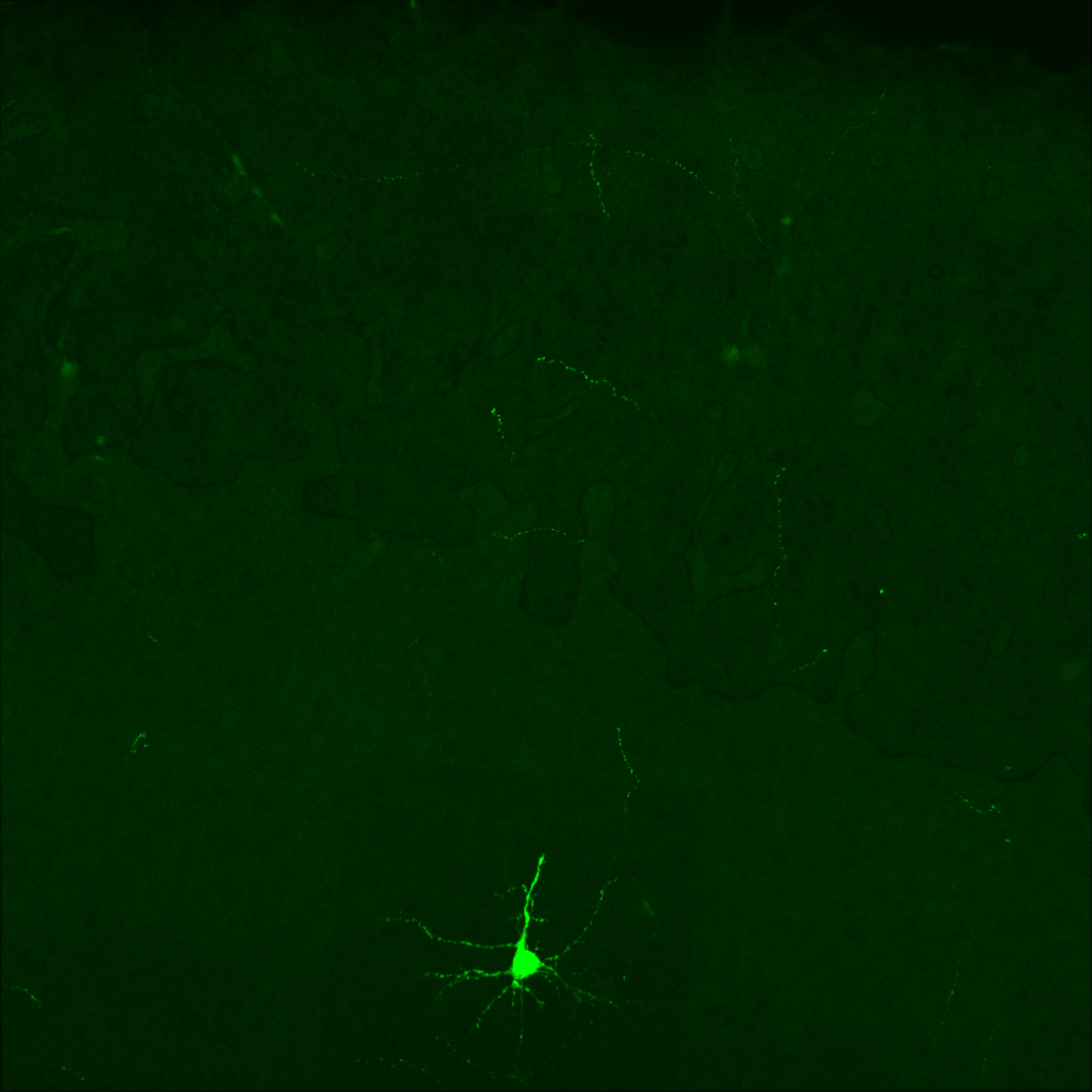

Supplement: Supplementary file 7 — Source data Fig. 1C,D,F [file 44318_2025_624_MOESM7_ESM.zip › Figure. 1C,D,F/1F/N/1.tif]

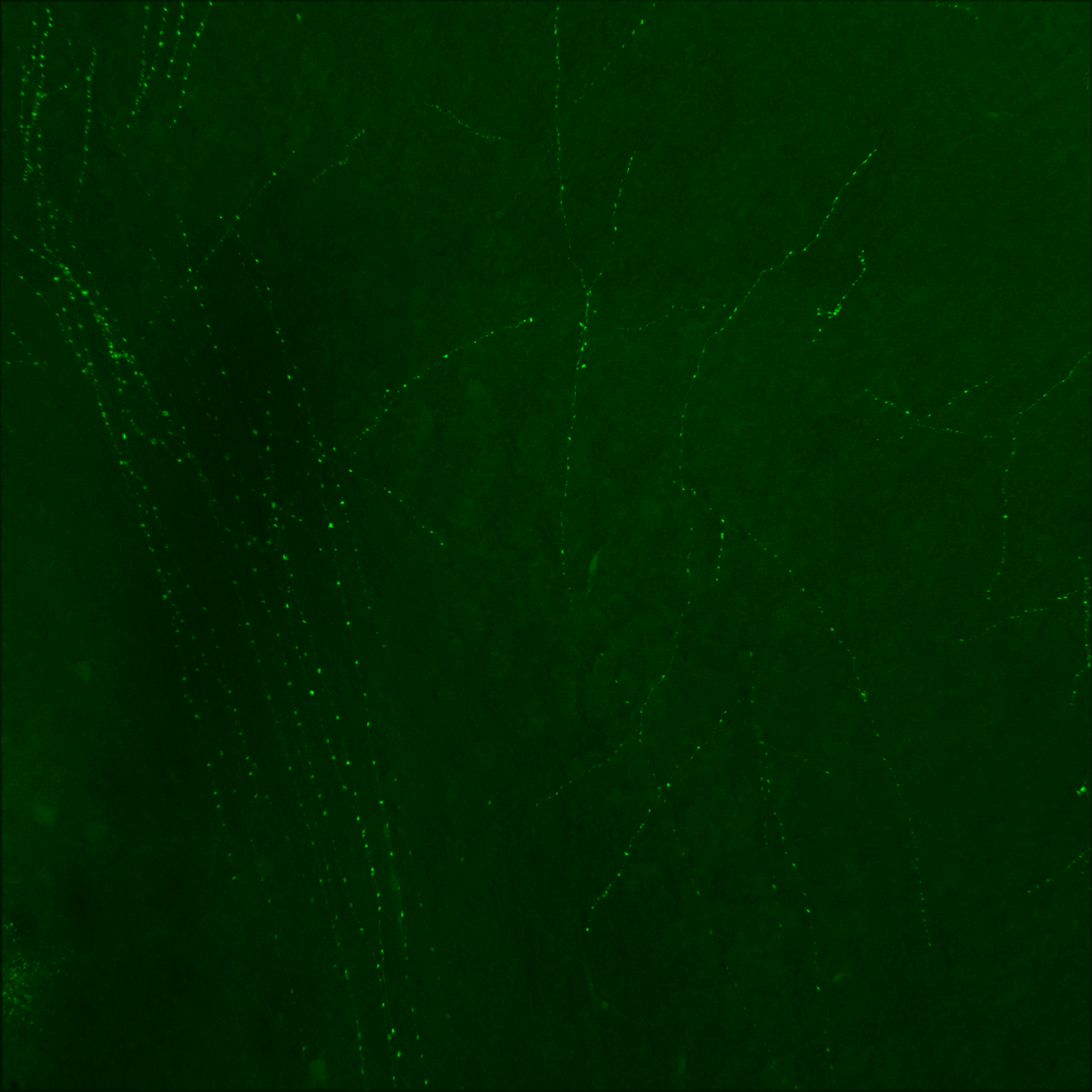

Supplement: Supplementary file 7 — Source data Fig. 1C,D,F [file 44318_2025_624_MOESM7_ESM.zip › Figure. 1C,D,F/1F/N/4.tif]

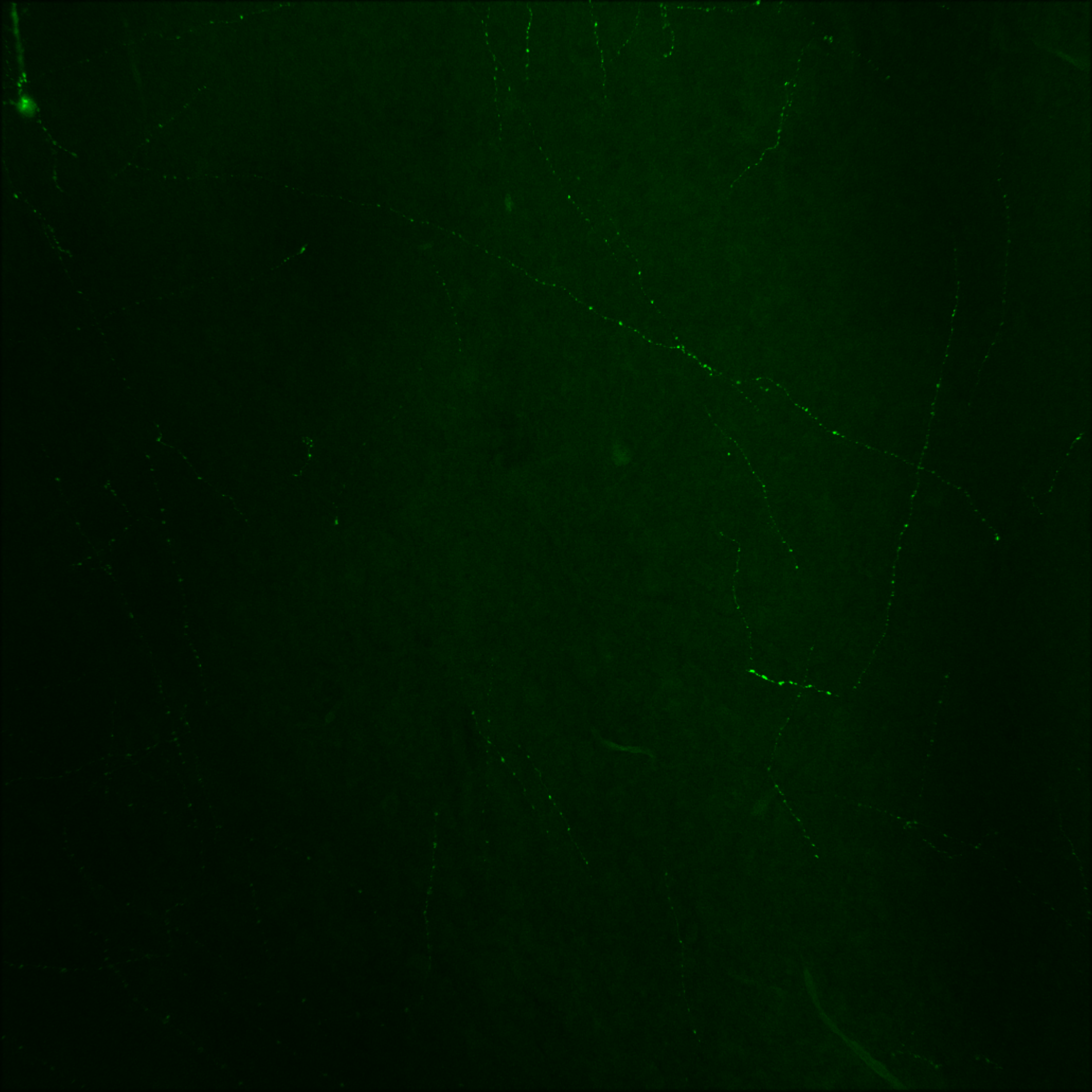

Supplement: Supplementary file 7 — Source data Fig. 1C,D,F [file 44318_2025_624_MOESM7_ESM.zip › Figure. 1C,D,F/1F/N+3/3.tif]

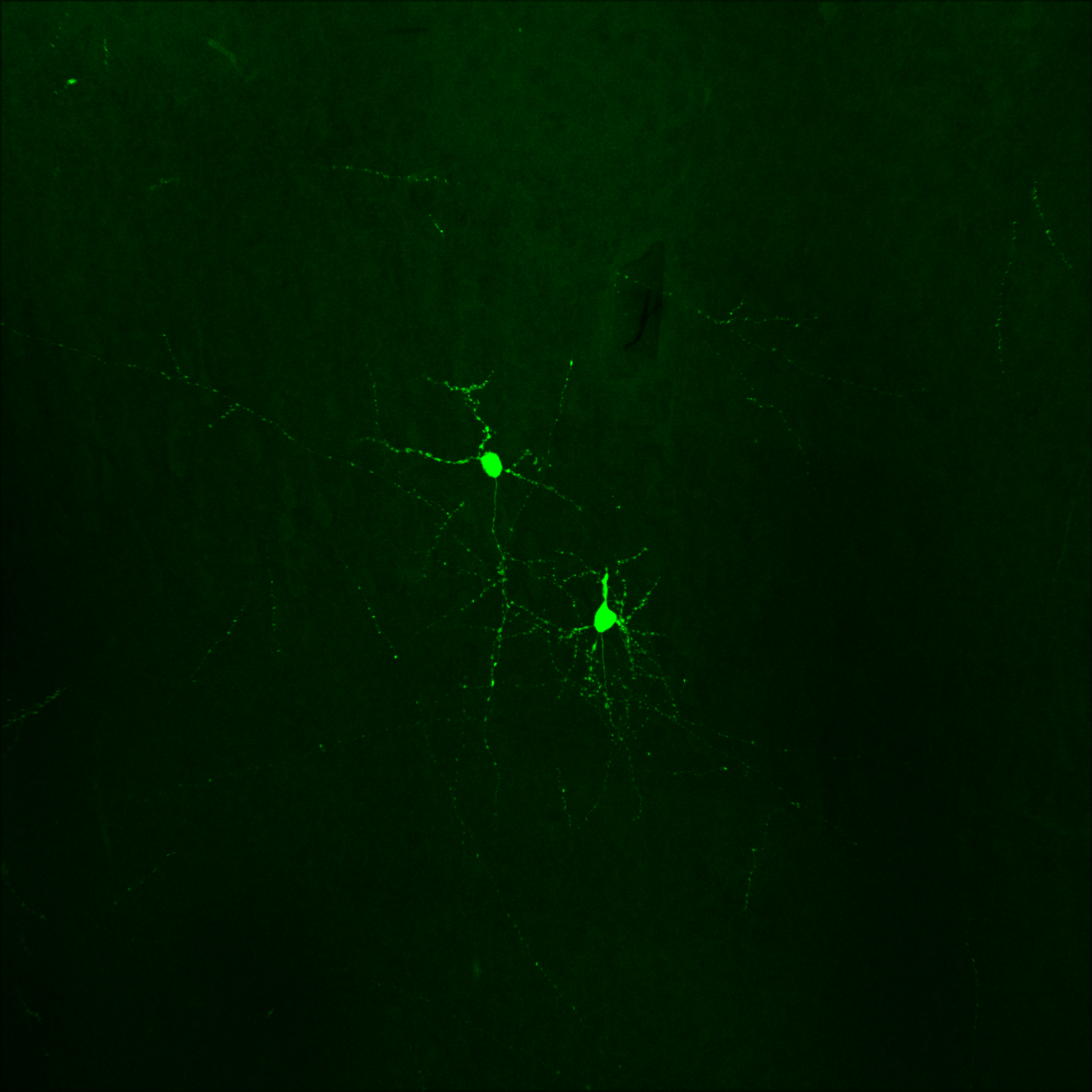

Supplement: Supplementary file 7 — Source data Fig. 1C,D,F [file 44318_2025_624_MOESM7_ESM.zip › Figure. 1C,D,F/1F/N+3/2.tif]

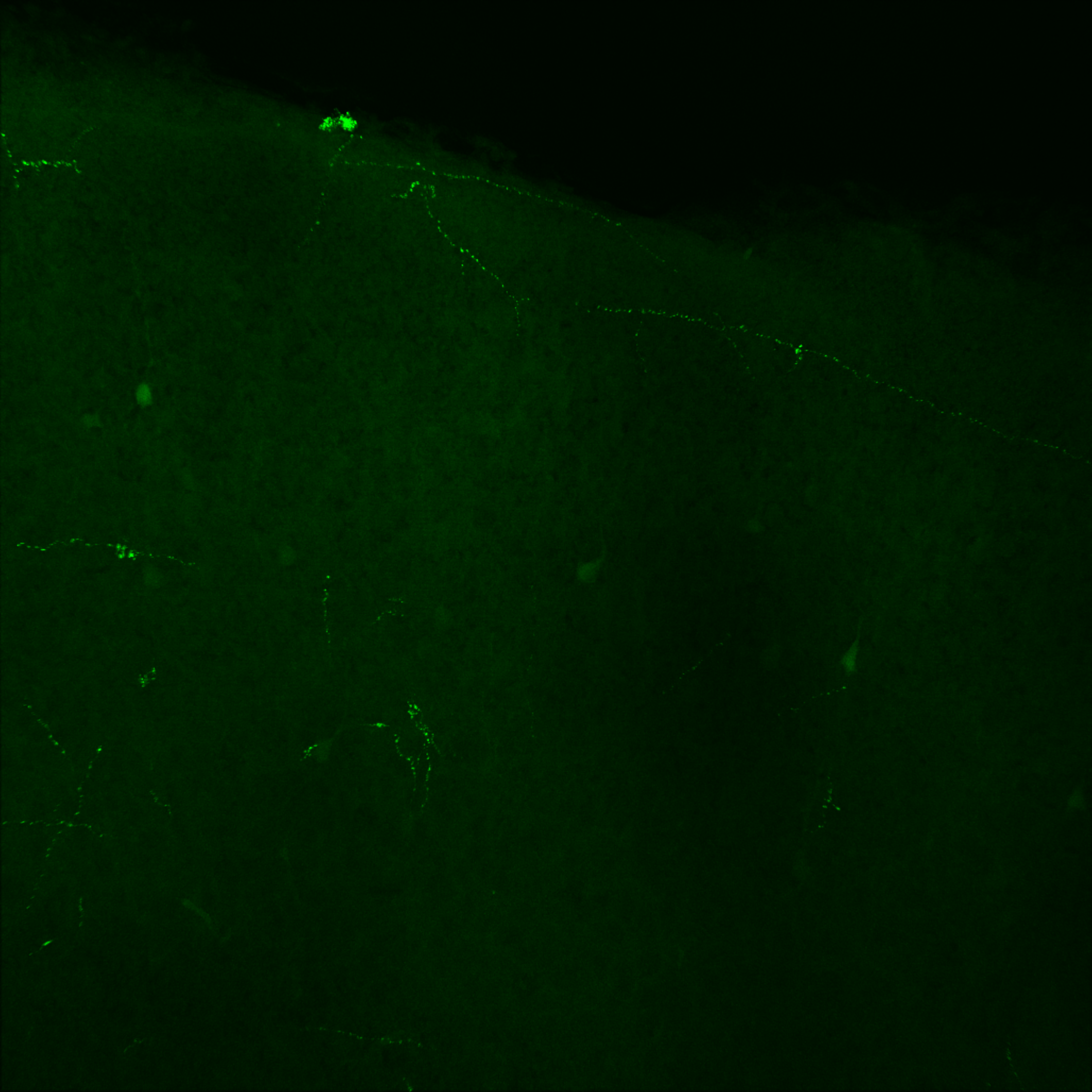

Supplement: Supplementary file 7 — Source data Fig. 1C,D,F [file 44318_2025_624_MOESM7_ESM.zip › Figure. 1C,D,F/1F/N+3/1.tif]

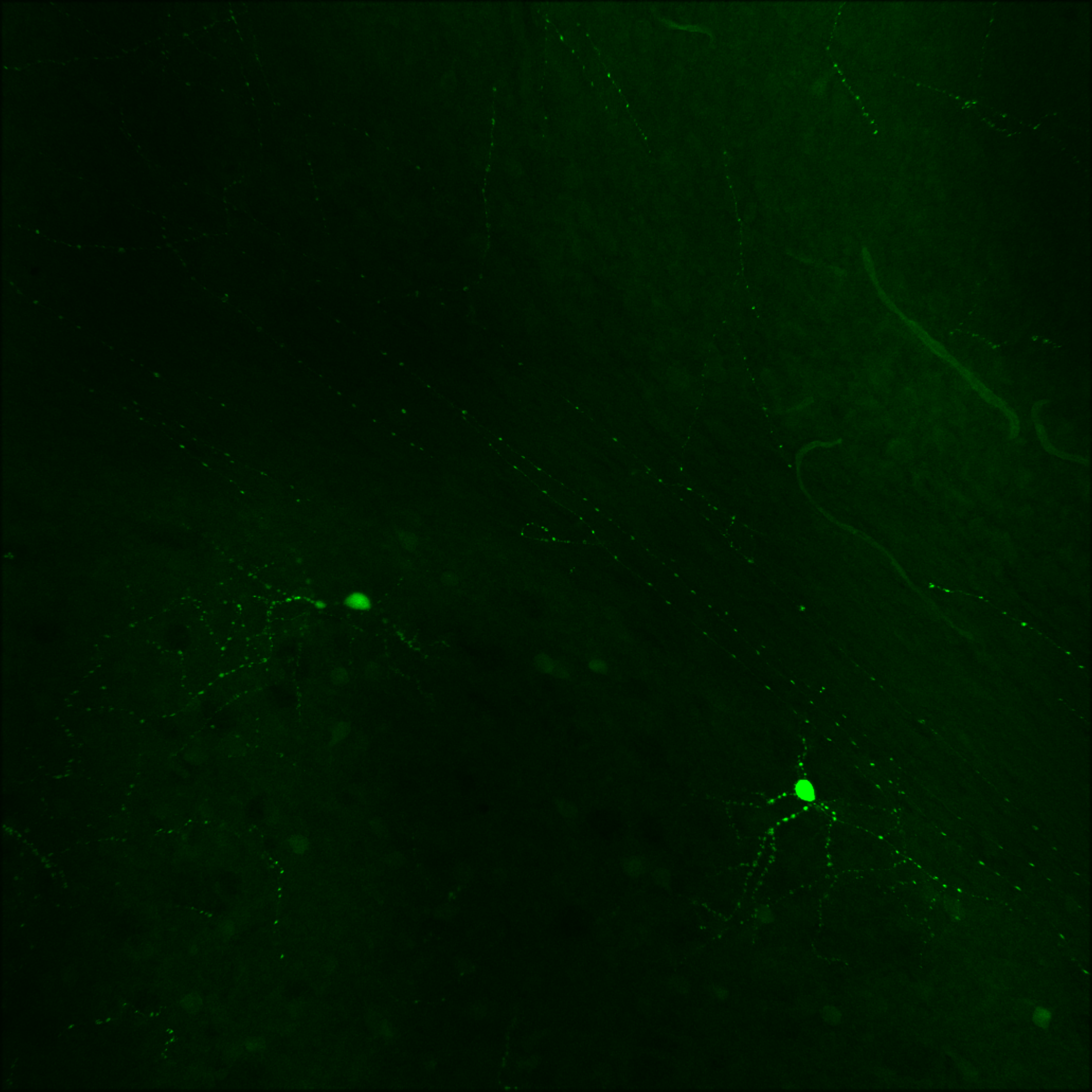

Supplement: Supplementary file 7 — Source data Fig. 1C,D,F [file 44318_2025_624_MOESM7_ESM.zip › Figure. 1C,D,F/1F/N+3/4.tif]

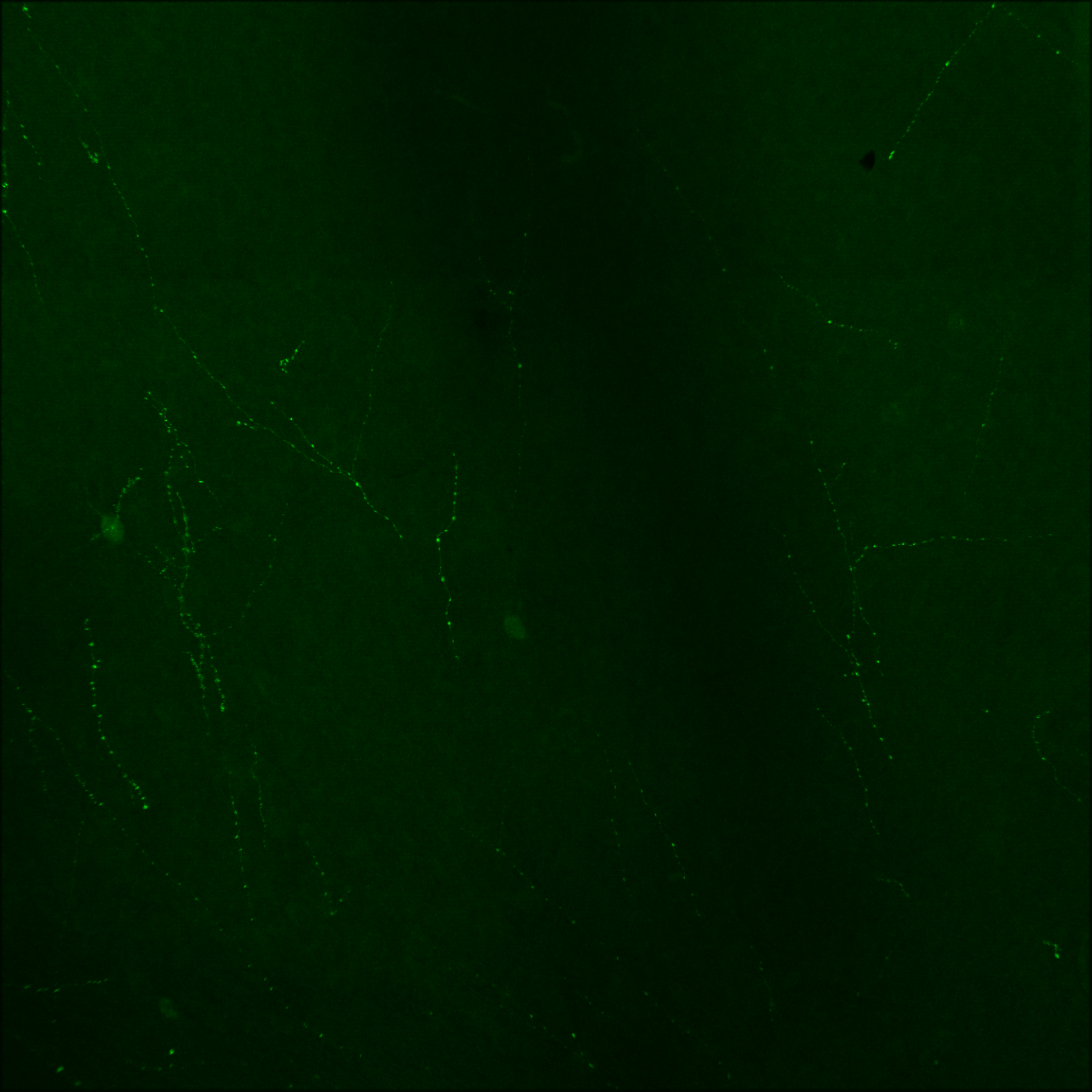

Supplement: Supplementary file 7 — Source data Fig. 1C,D,F [file 44318_2025_624_MOESM7_ESM.zip › Figure. 1C,D,F/1F/N+2/3.tif]

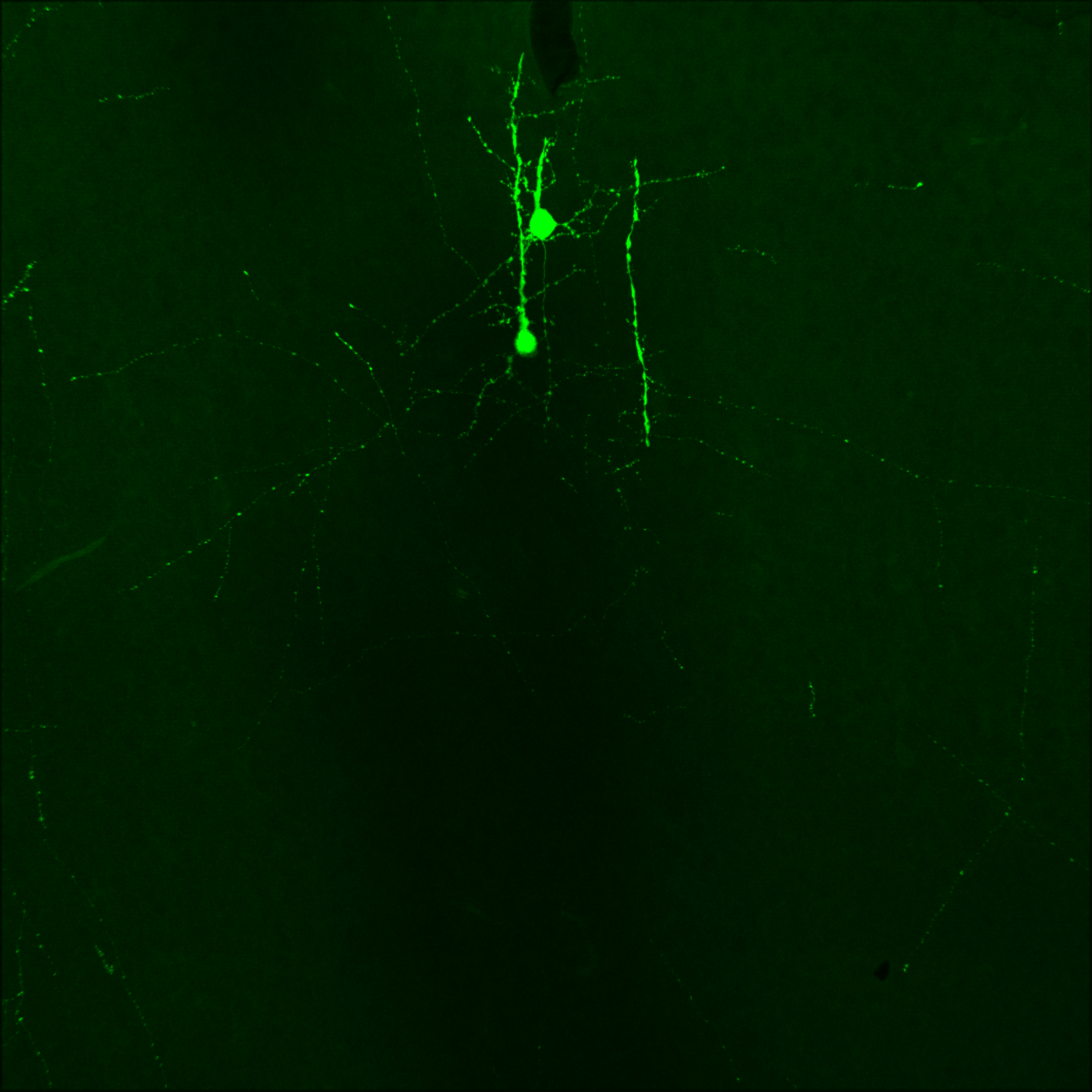

Supplement: Supplementary file 7 — Source data Fig. 1C,D,F [file 44318_2025_624_MOESM7_ESM.zip › Figure. 1C,D,F/1F/N+2/2.tif]

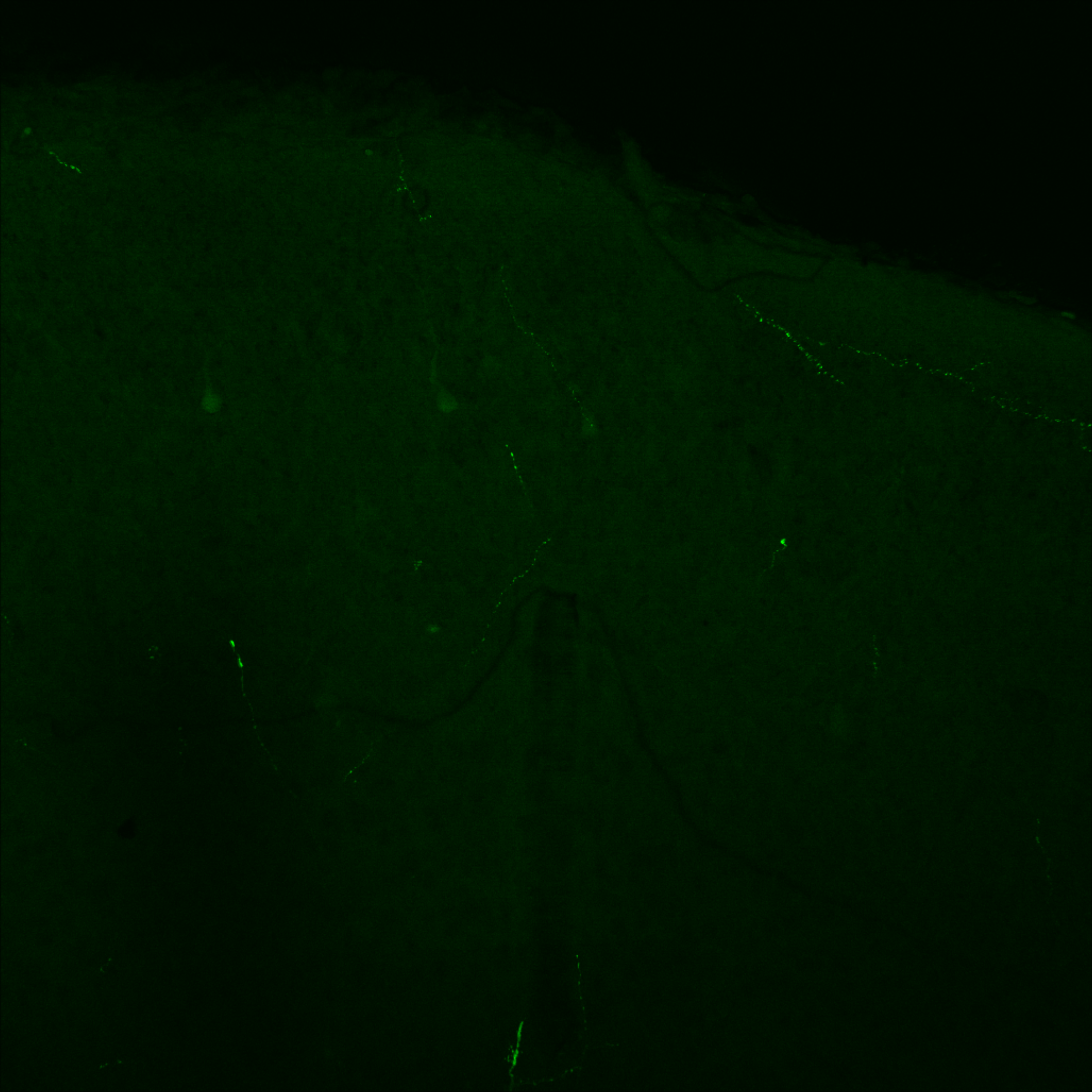

Supplement: Supplementary file 7 — Source data Fig. 1C,D,F [file 44318_2025_624_MOESM7_ESM.zip › Figure. 1C,D,F/1F/N+2/1.tif]

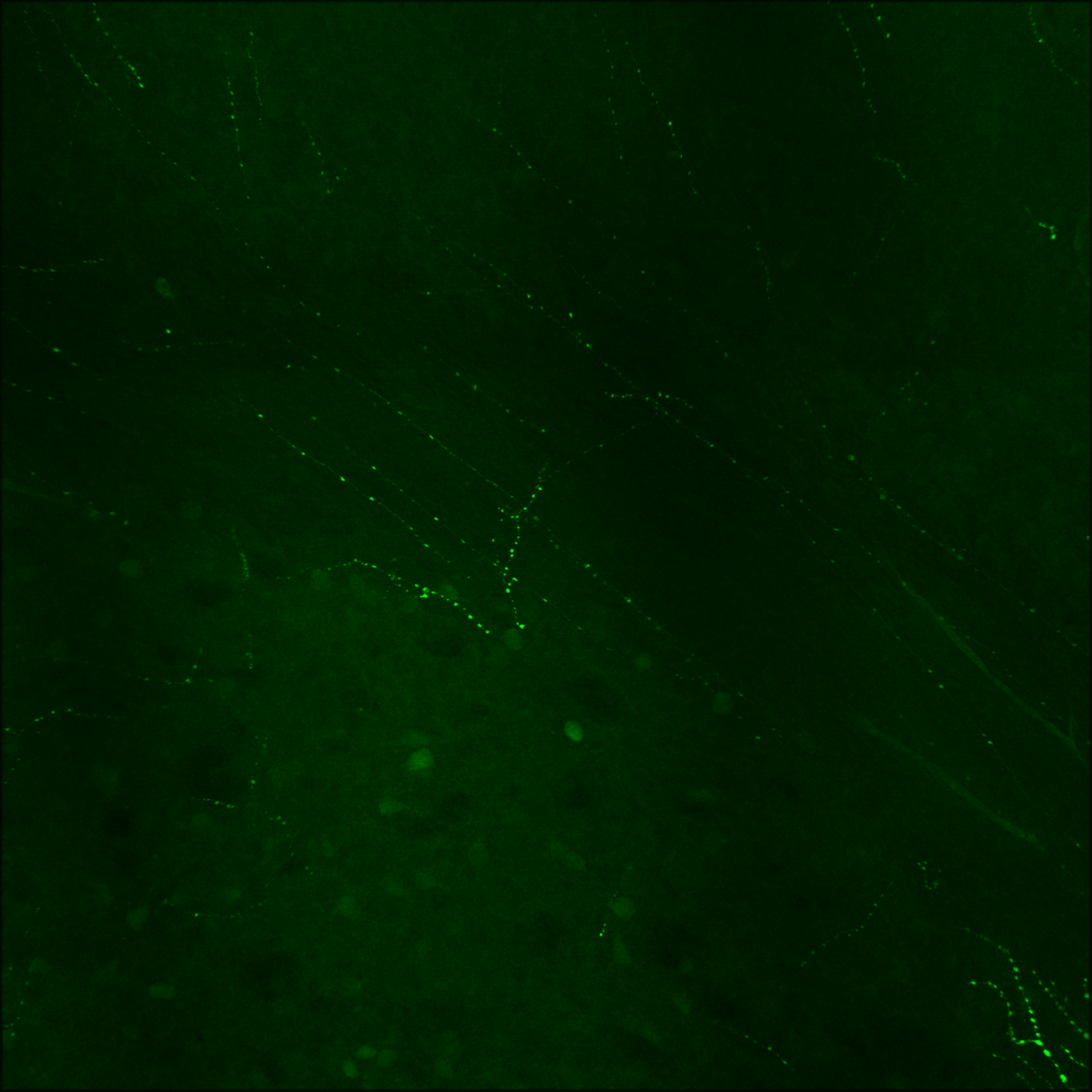

Supplement: Supplementary file 7 — Source data Fig. 1C,D,F [file 44318_2025_624_MOESM7_ESM.zip › Figure. 1C,D,F/1F/N+2/4.tif]

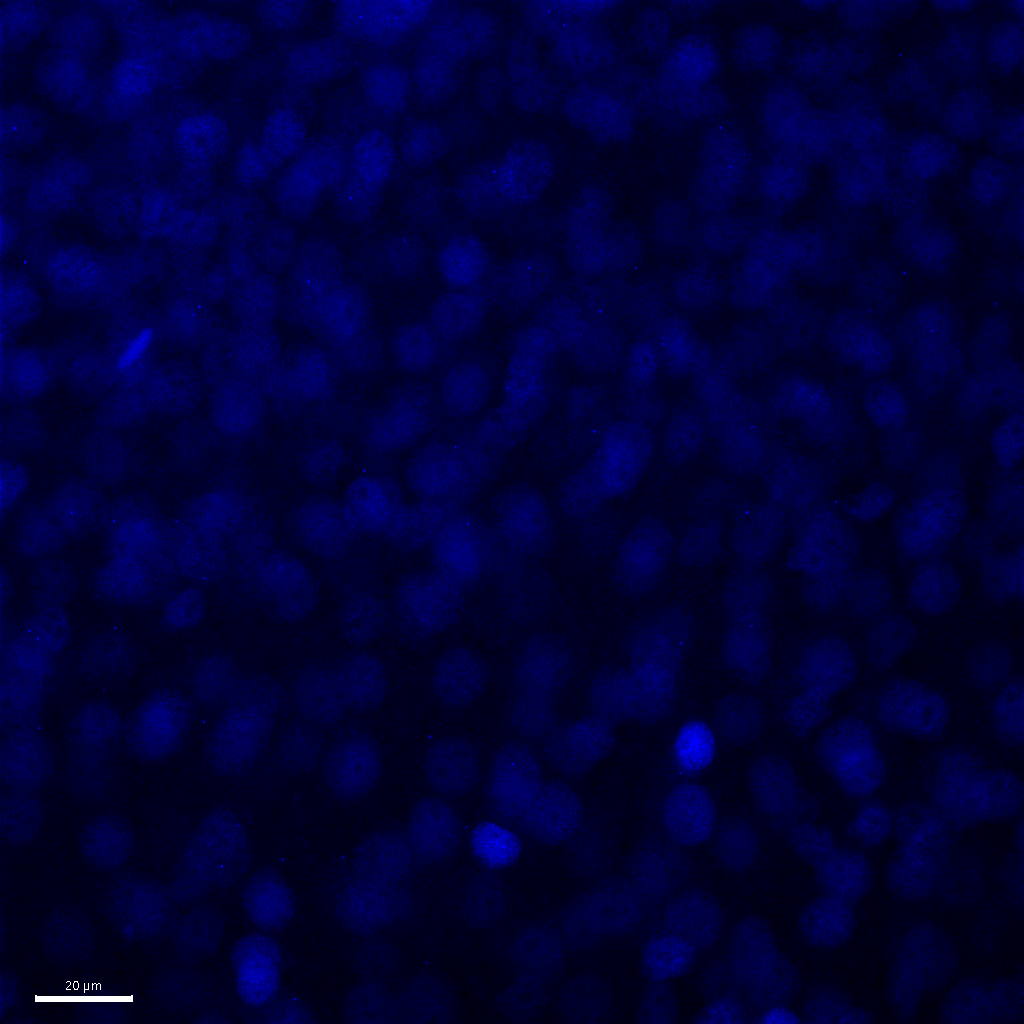

Supplement: Supplementary file 8 — Source data Fig. 1G [file 44318_2025_624_MOESM8_ESM.zip › 1G/n7/satb2+ctip2+fog2/ctip2.tif]

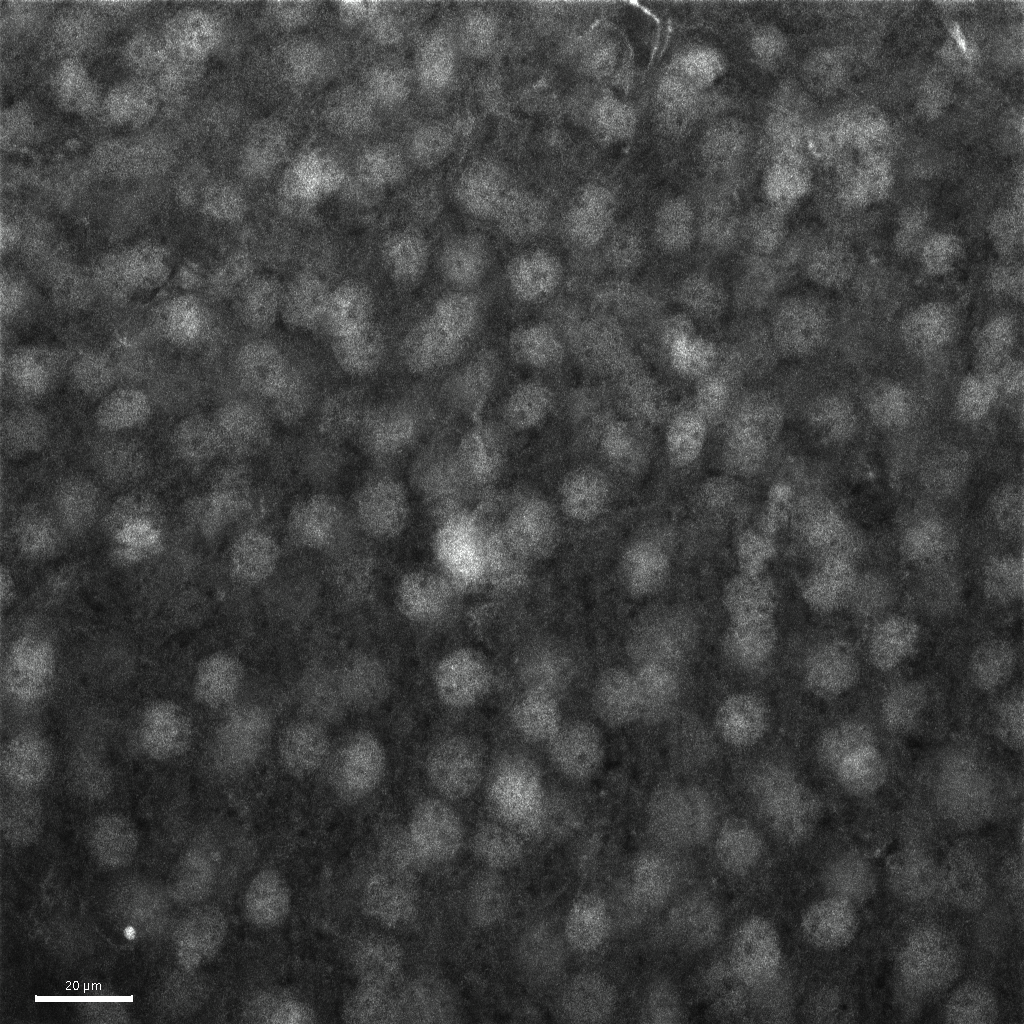

Supplement: Supplementary file 8 — Source data Fig. 1G [file 44318_2025_624_MOESM8_ESM.zip › 1G/n7/satb2+ctip2+fog2/satb2.tif]

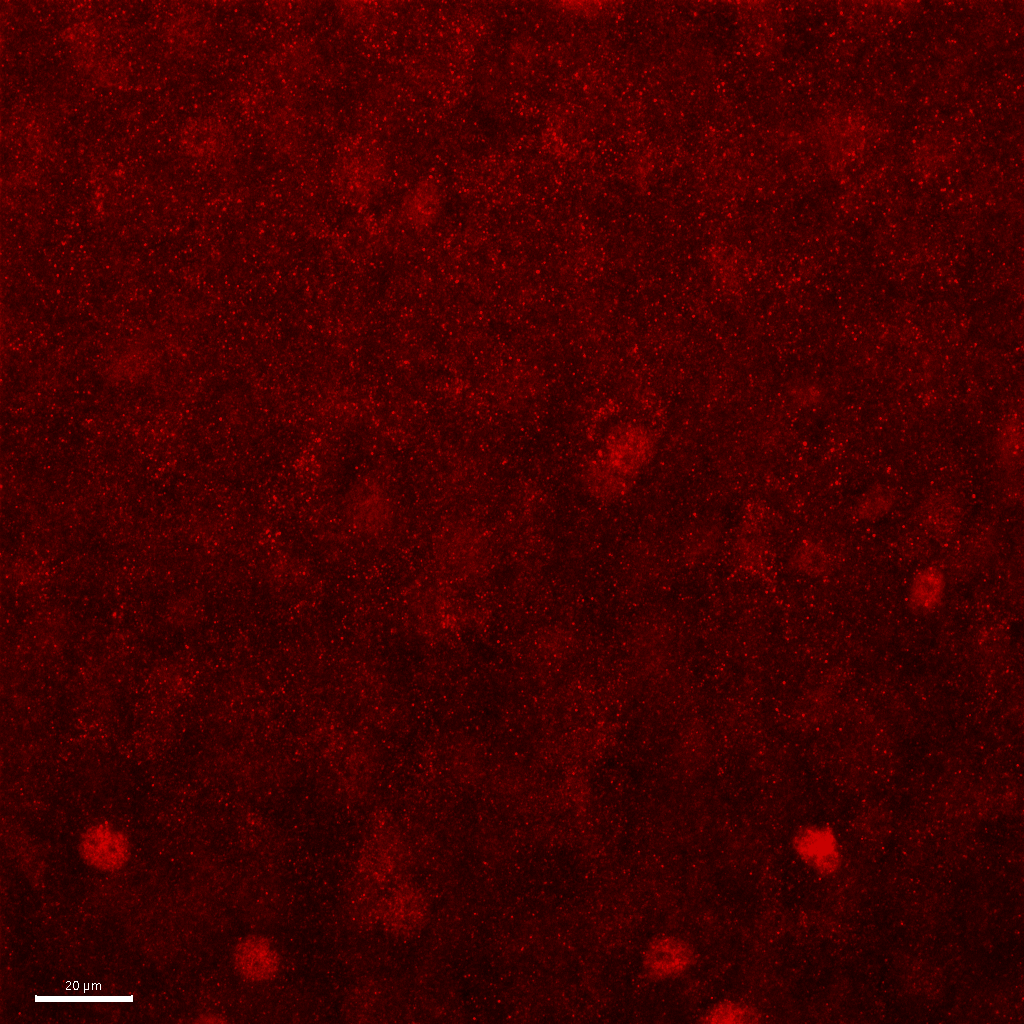

Supplement: Supplementary file 8 — Source data Fig. 1G [file 44318_2025_624_MOESM8_ESM.zip › 1G/n7/satb2+ctip2+fog2/fog2.tif]

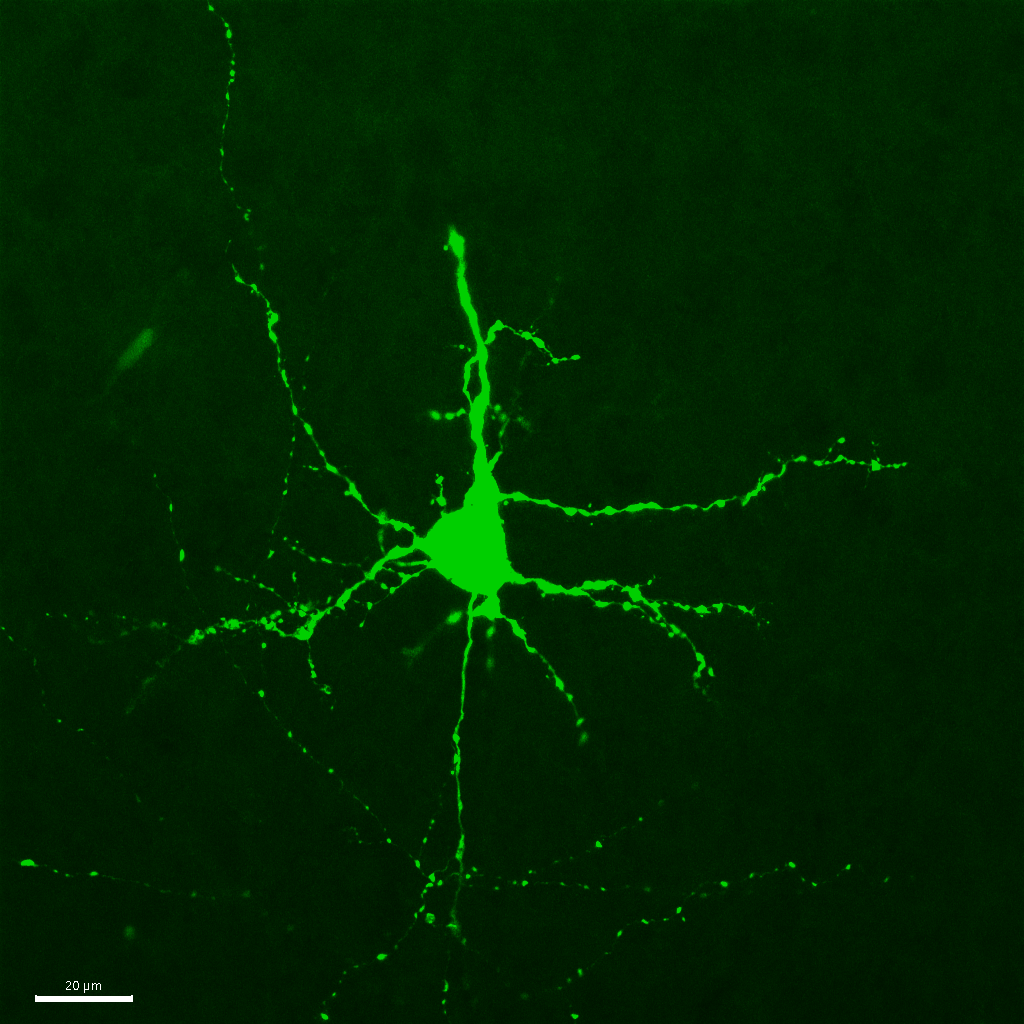

Supplement: Supplementary file 8 — Source data Fig. 1G [file 44318_2025_624_MOESM8_ESM.zip › 1G/n7/satb2+ctip2+fog2/GFP.tif]

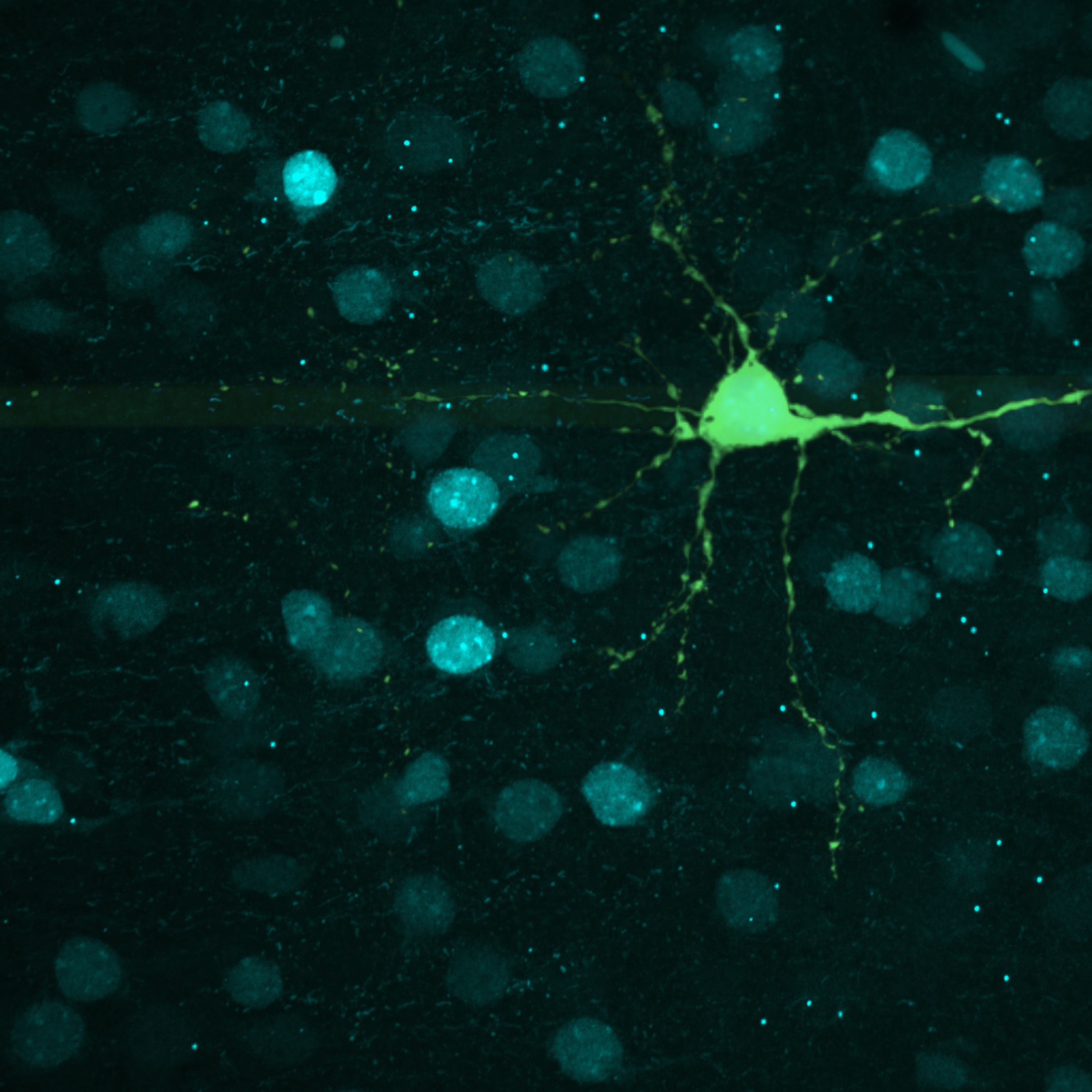

Supplement: Supplementary file 8 — Source data Fig. 1G [file 44318_2025_624_MOESM8_ESM.zip › 1G/n7/CFSE+GFP/CFSE+GFP.tif]

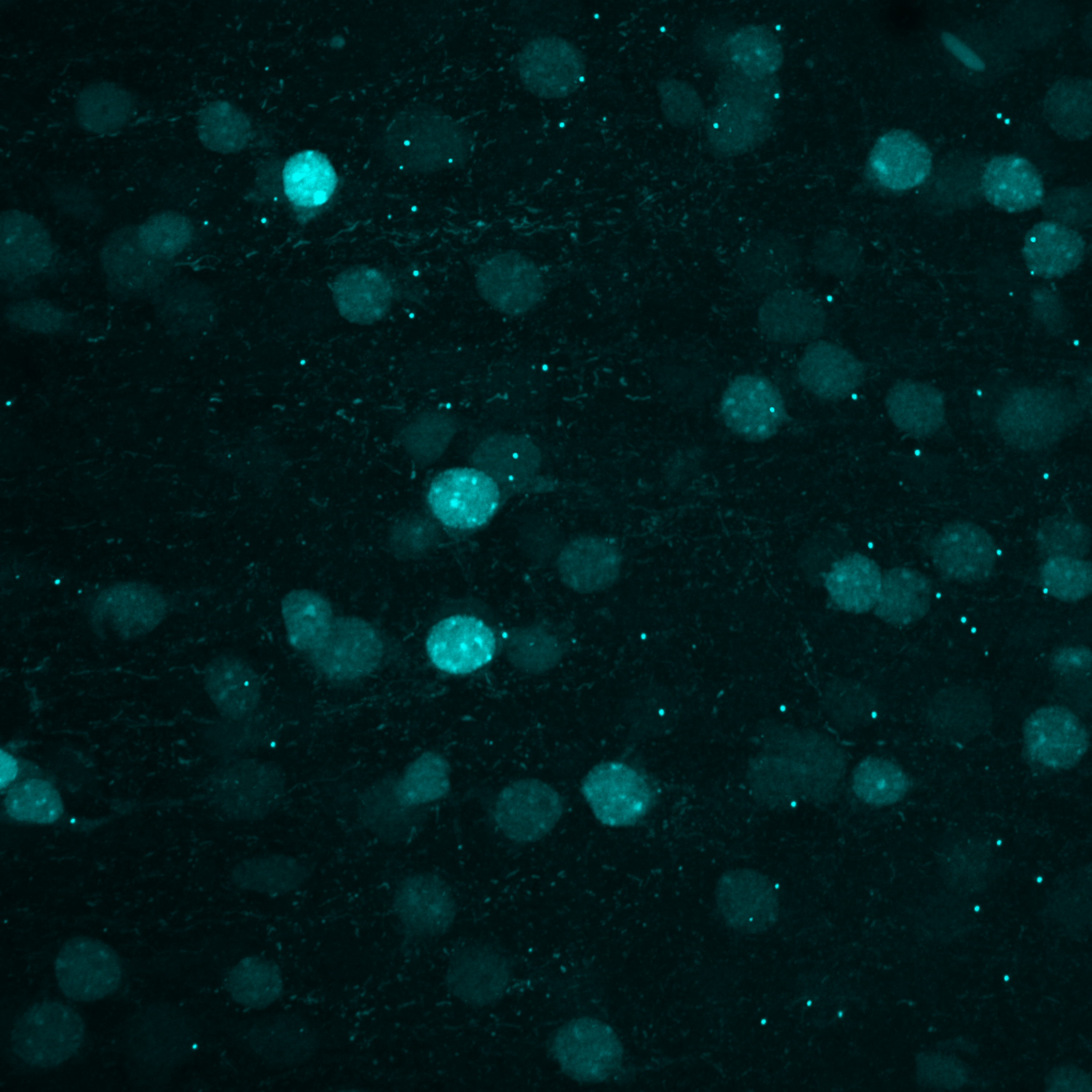

Supplement: Supplementary file 8 — Source data Fig. 1G [file 44318_2025_624_MOESM8_ESM.zip › 1G/n7/CFSE+GFP/CFSE.tif]

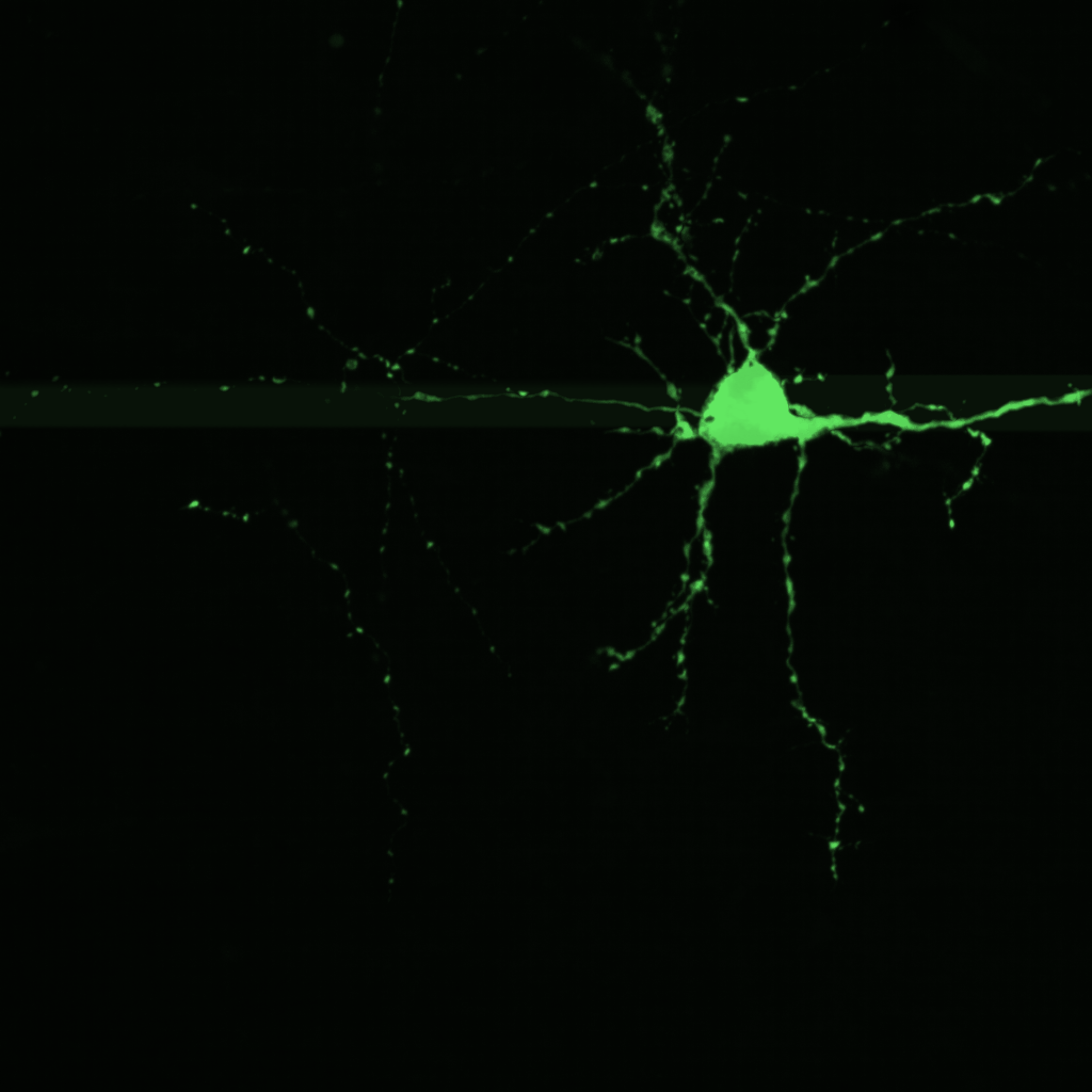

Supplement: Supplementary file 8 — Source data Fig. 1G [file 44318_2025_624_MOESM8_ESM.zip › 1G/n7/CFSE+GFP/GFP.tif]

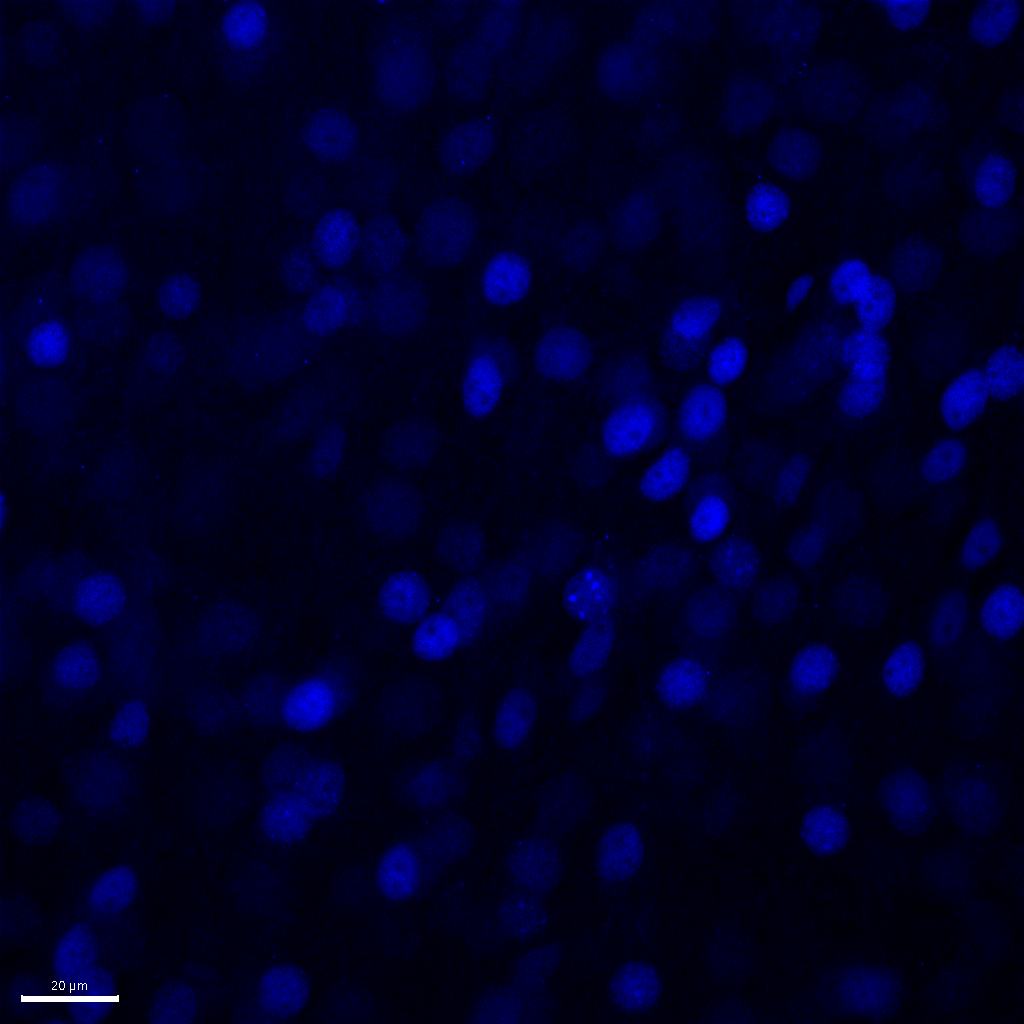

Supplement: Supplementary file 8 — Source data Fig. 1G [file 44318_2025_624_MOESM8_ESM.zip › 1G/n6/satb2+ctip2+fog2/ctip2.tif]

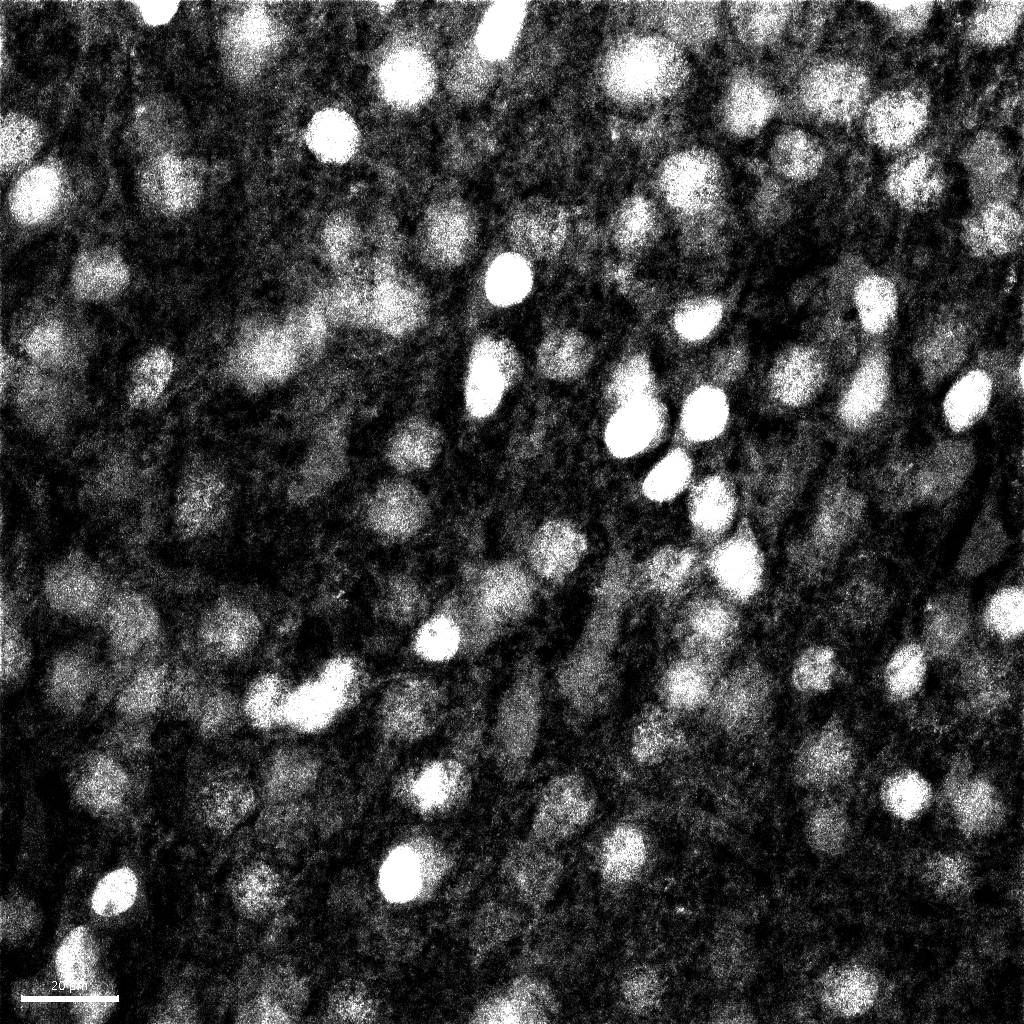

Supplement: Supplementary file 8 — Source data Fig. 1G [file 44318_2025_624_MOESM8_ESM.zip › 1G/n6/satb2+ctip2+fog2/satb2.tif]

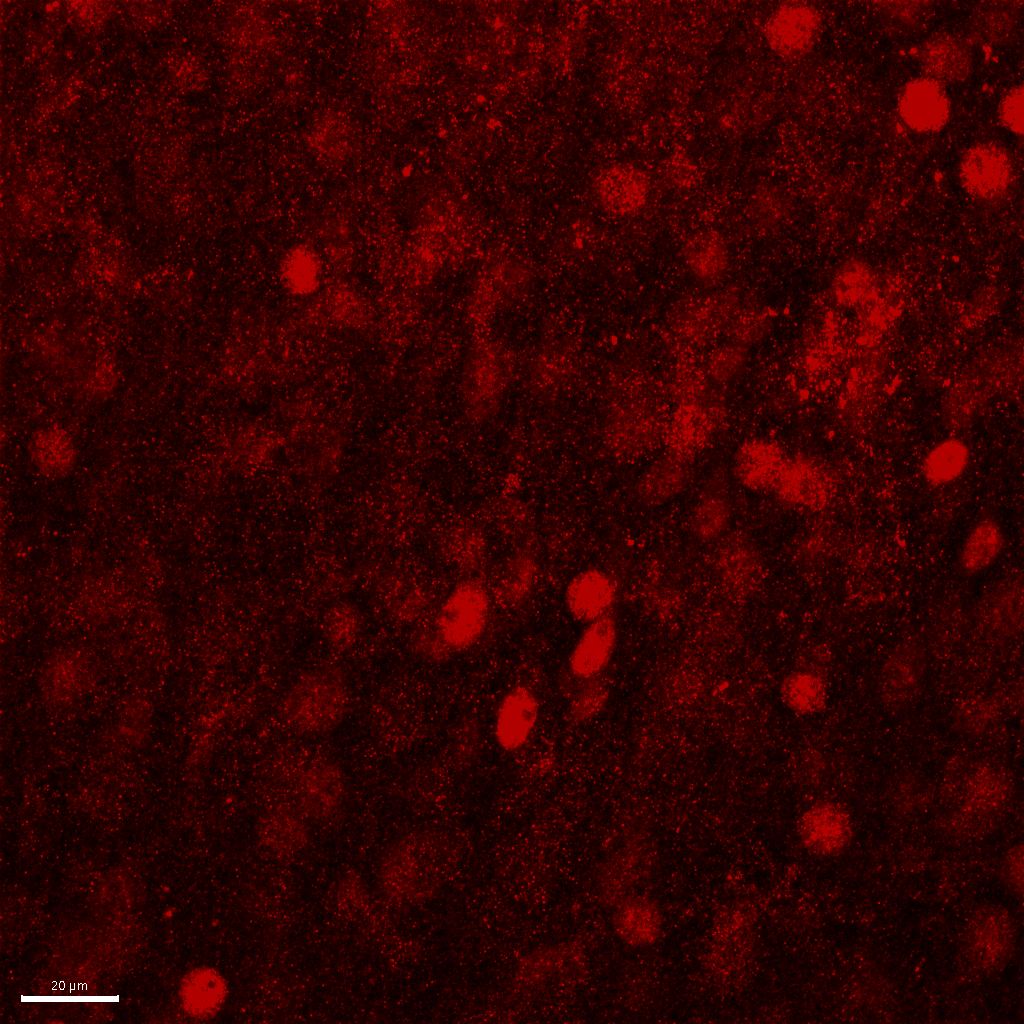

Supplement: Supplementary file 8 — Source data Fig. 1G [file 44318_2025_624_MOESM8_ESM.zip › 1G/n6/satb2+ctip2+fog2/fog2.tif]

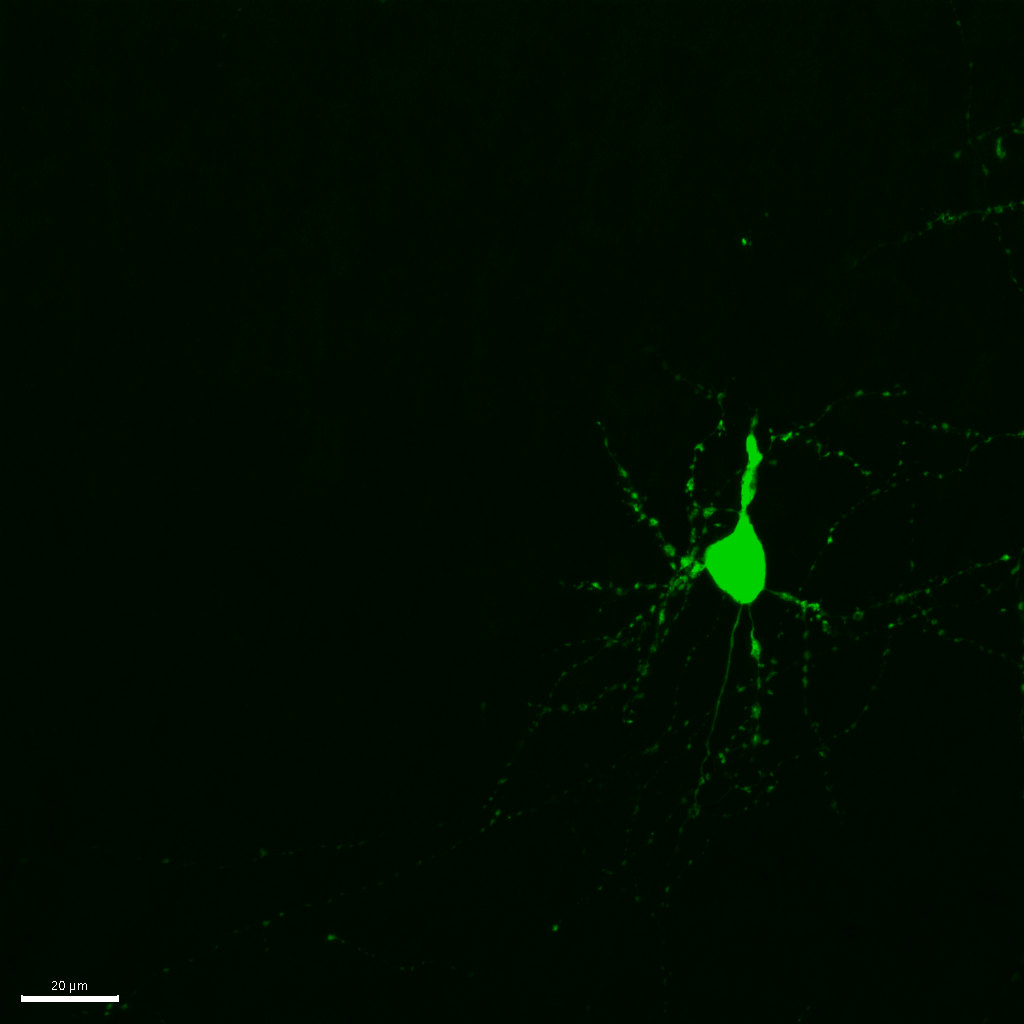

Supplement: Supplementary file 8 — Source data Fig. 1G [file 44318_2025_624_MOESM8_ESM.zip › 1G/n6/satb2+ctip2+fog2/GFP.tif]

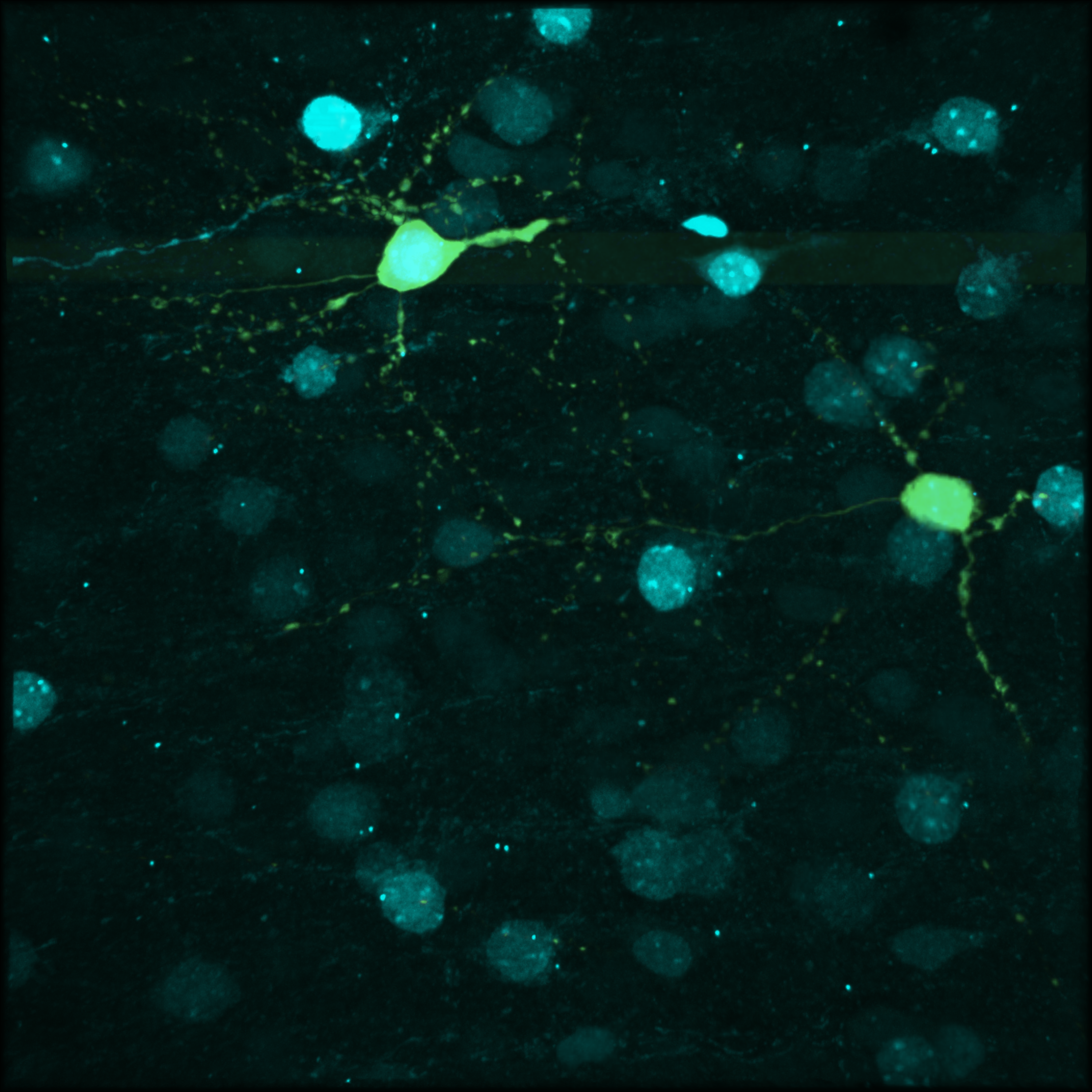

Supplement: Supplementary file 8 — Source data Fig. 1G [file 44318_2025_624_MOESM8_ESM.zip › 1G/n6/CFSE+GFP/CFSE+GFP.tif]

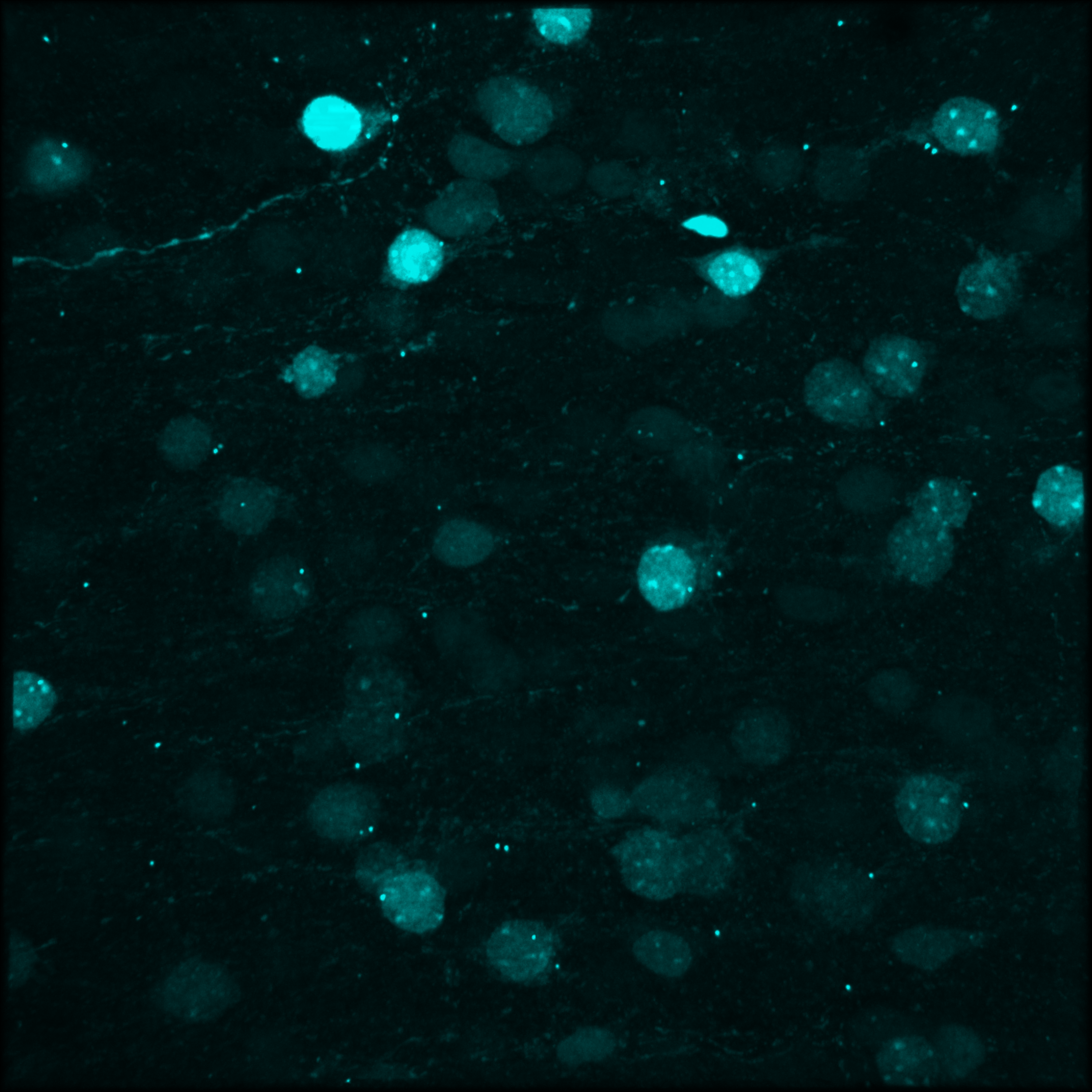

Supplement: Supplementary file 8 — Source data Fig. 1G [file 44318_2025_624_MOESM8_ESM.zip › 1G/n6/CFSE+GFP/CFSE.tif]

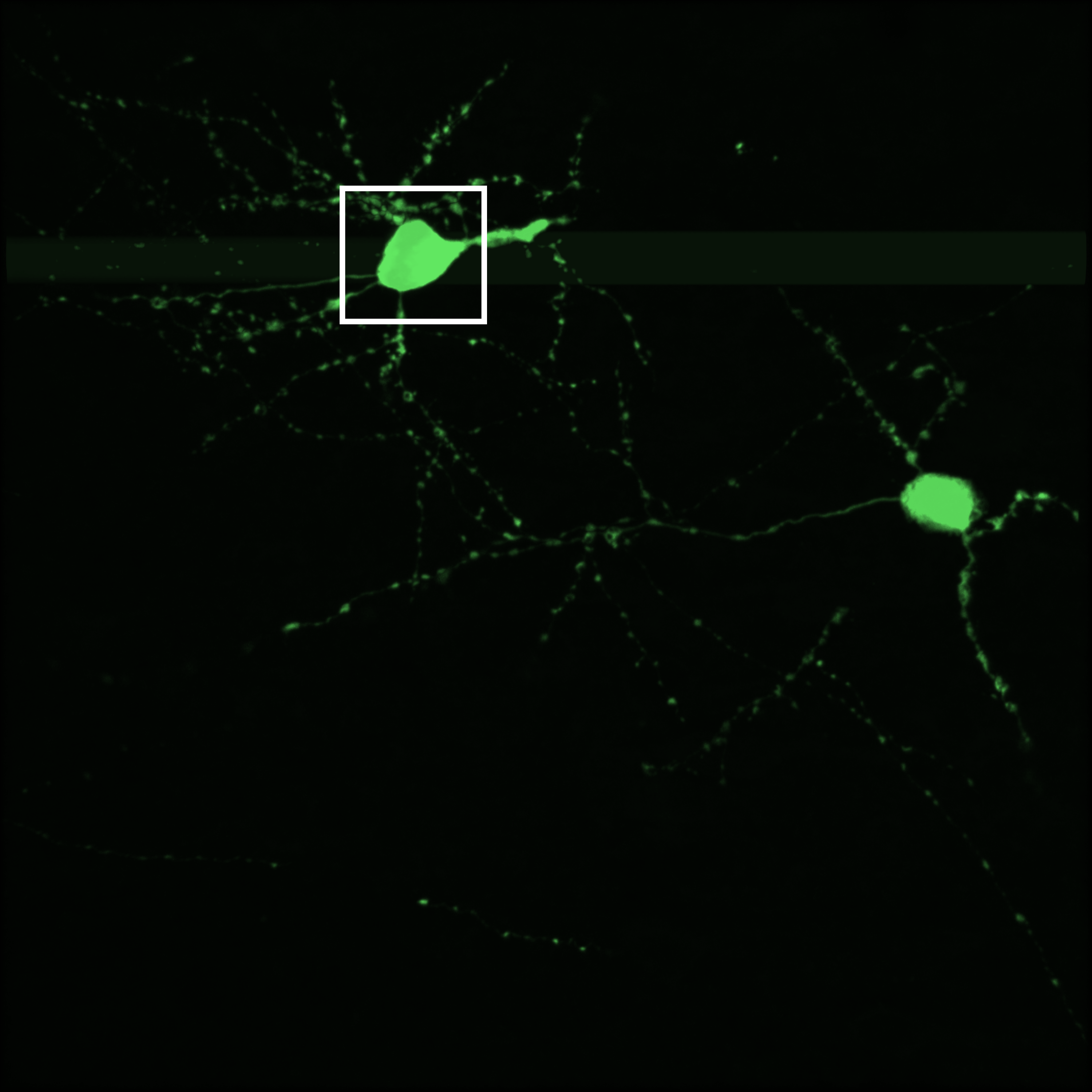

Supplement: Supplementary file 8 — Source data Fig. 1G [file 44318_2025_624_MOESM8_ESM.zip › 1G/n6/CFSE+GFP/GFP.tif]

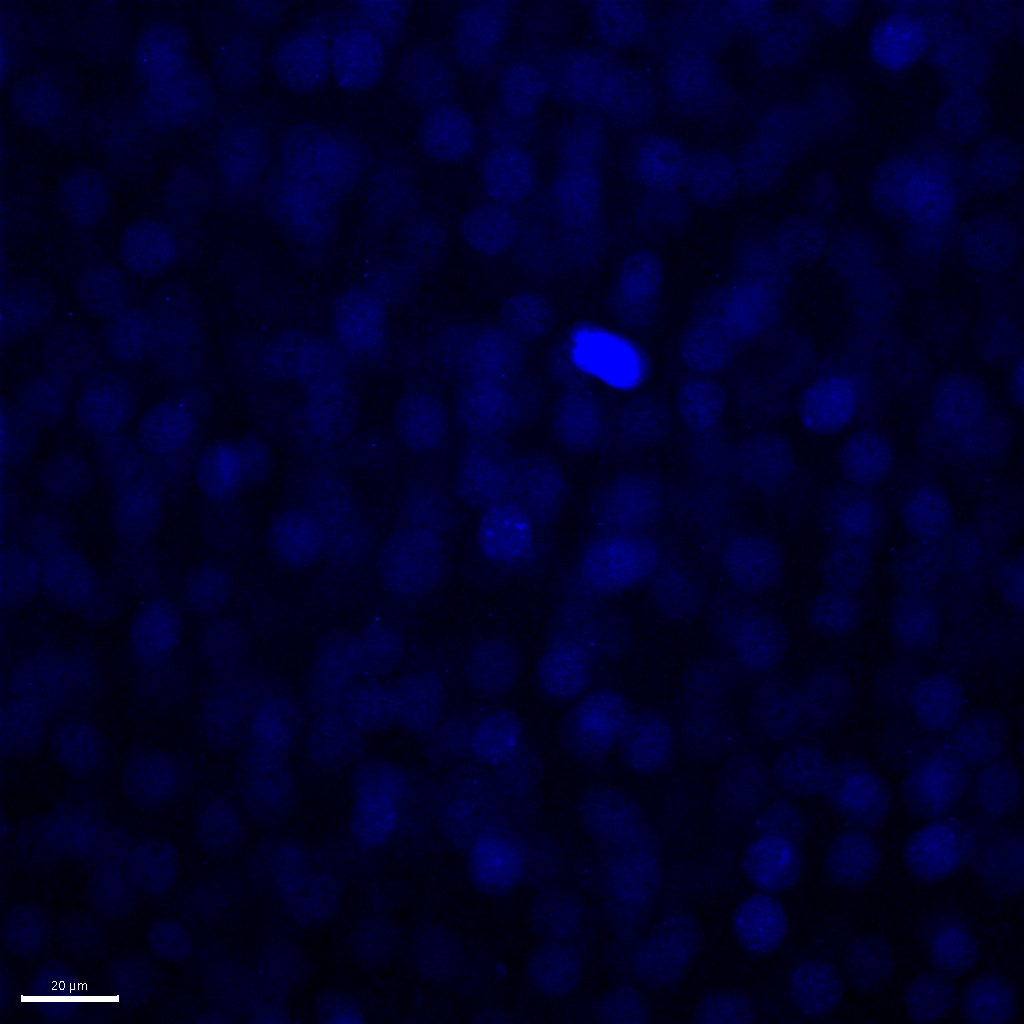

Supplement: Supplementary file 8 — Source data Fig. 1G [file 44318_2025_624_MOESM8_ESM.zip › 1G/n1/satb2+ctip2+fog2/ctip2.tif]

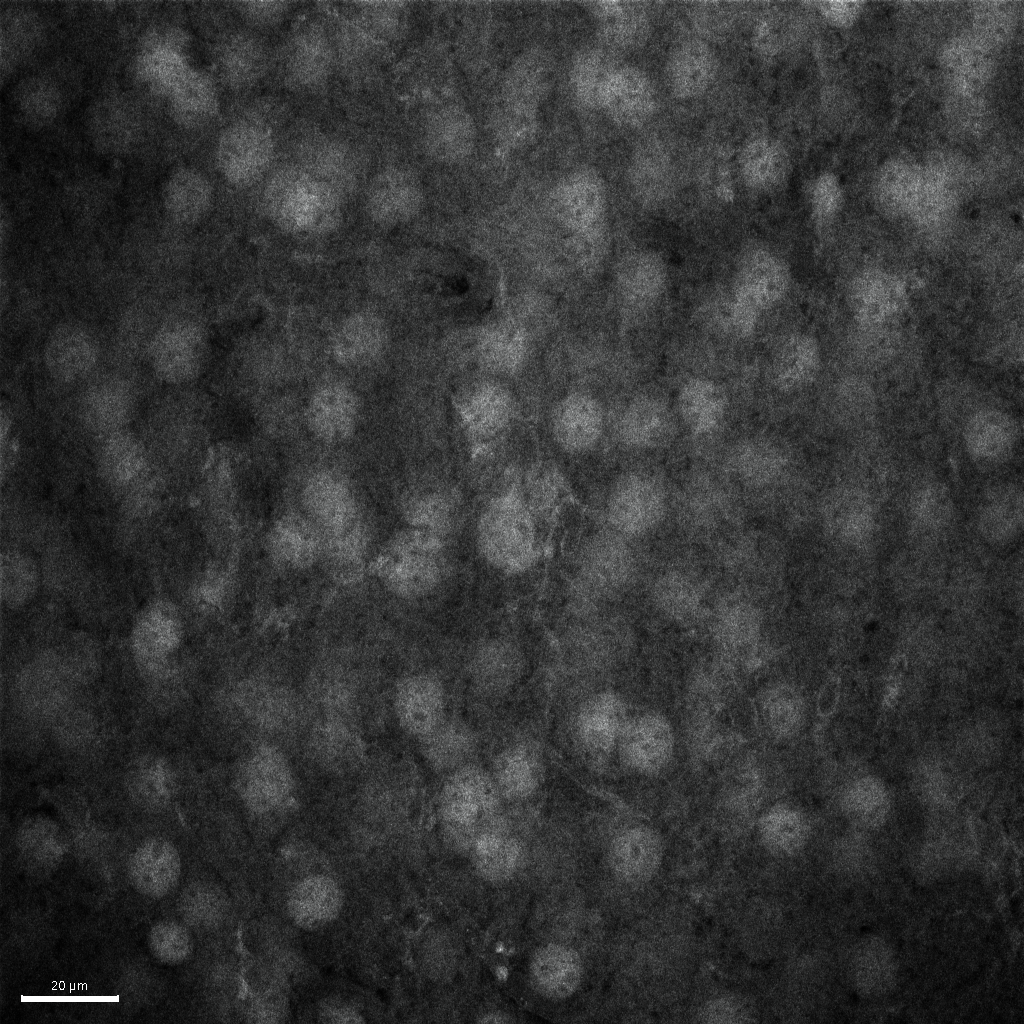

Supplement: Supplementary file 8 — Source data Fig. 1G [file 44318_2025_624_MOESM8_ESM.zip › 1G/n1/satb2+ctip2+fog2/satb2.tif]

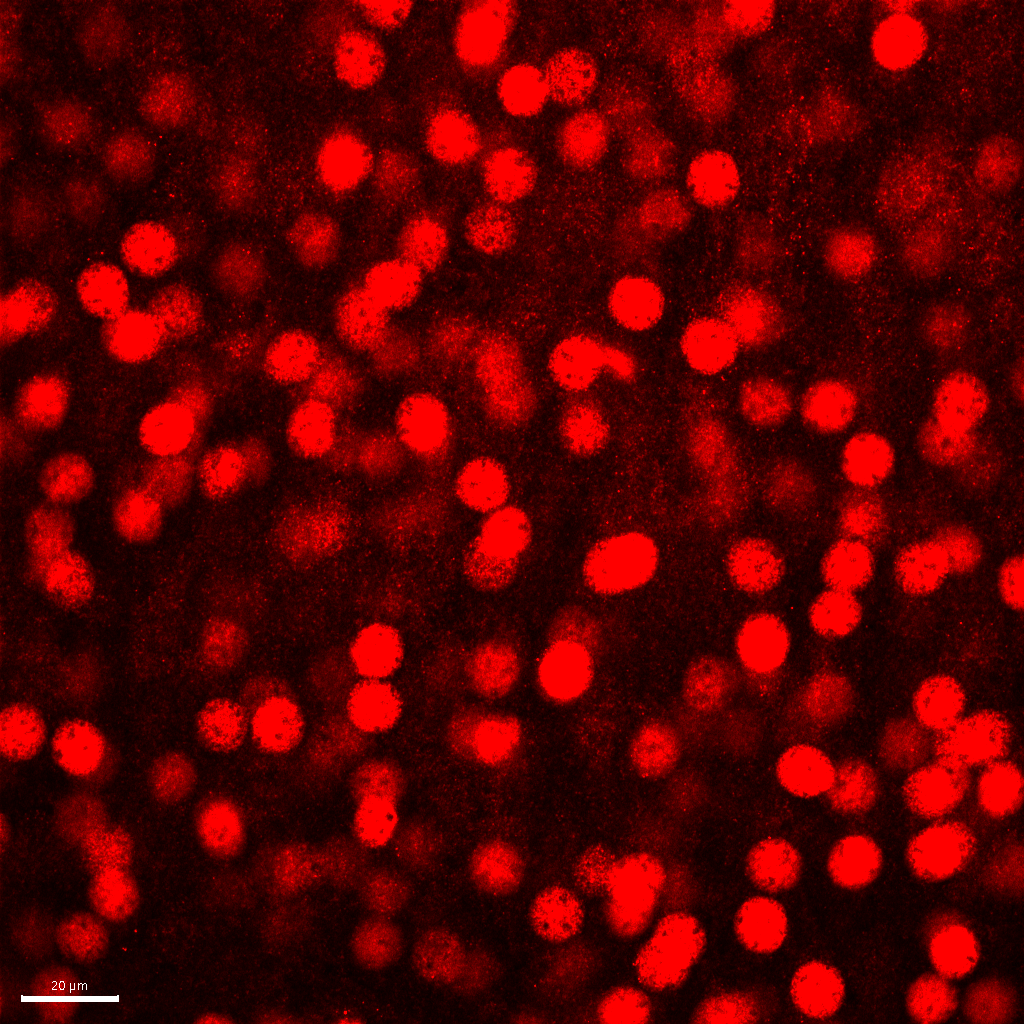

Supplement: Supplementary file 8 — Source data Fig. 1G [file 44318_2025_624_MOESM8_ESM.zip › 1G/n1/satb2+ctip2+fog2/fog2.tif]

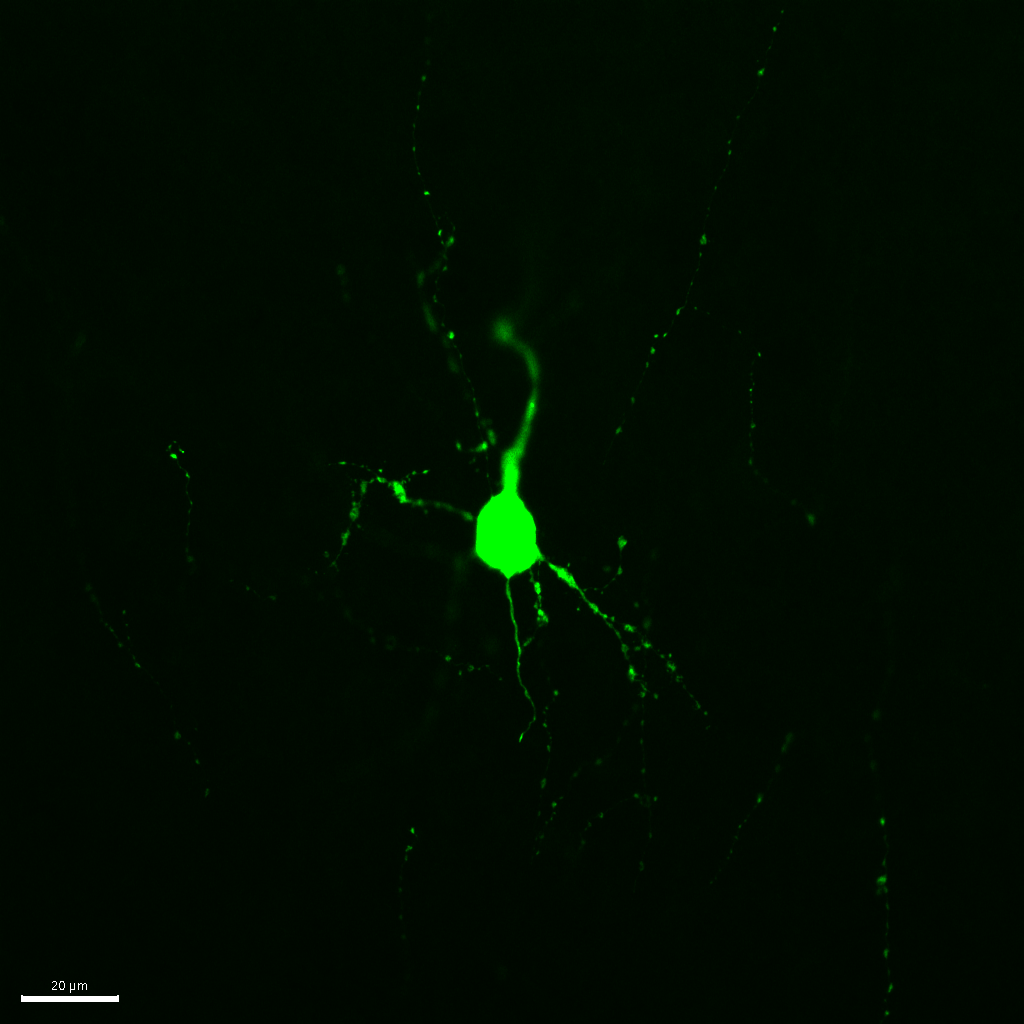

Supplement: Supplementary file 8 — Source data Fig. 1G [file 44318_2025_624_MOESM8_ESM.zip › 1G/n1/satb2+ctip2+fog2/GFP.tif]

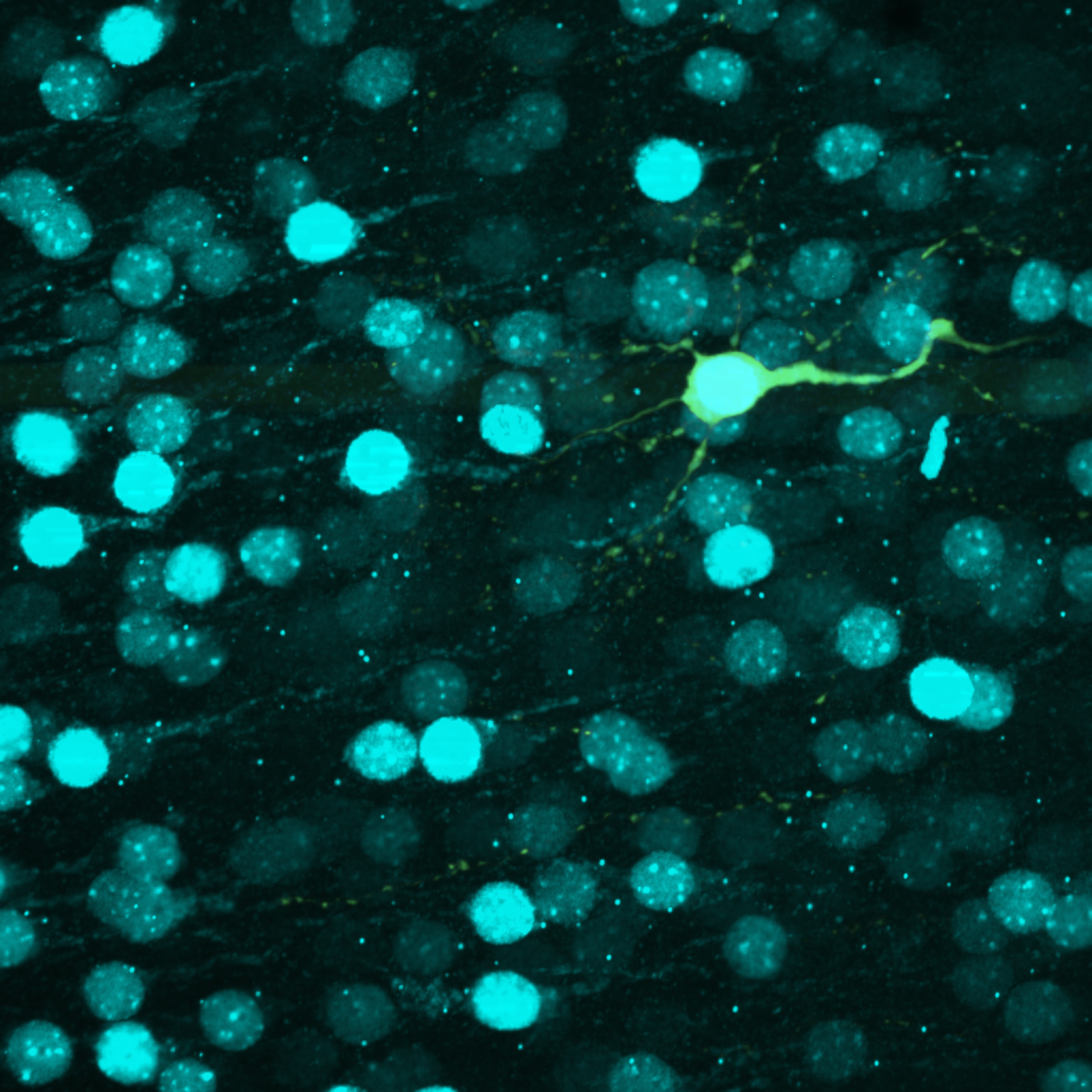

Supplement: Supplementary file 8 — Source data Fig. 1G [file 44318_2025_624_MOESM8_ESM.zip › 1G/n1/CFSE+GFP/CFSE+GFP.tif]

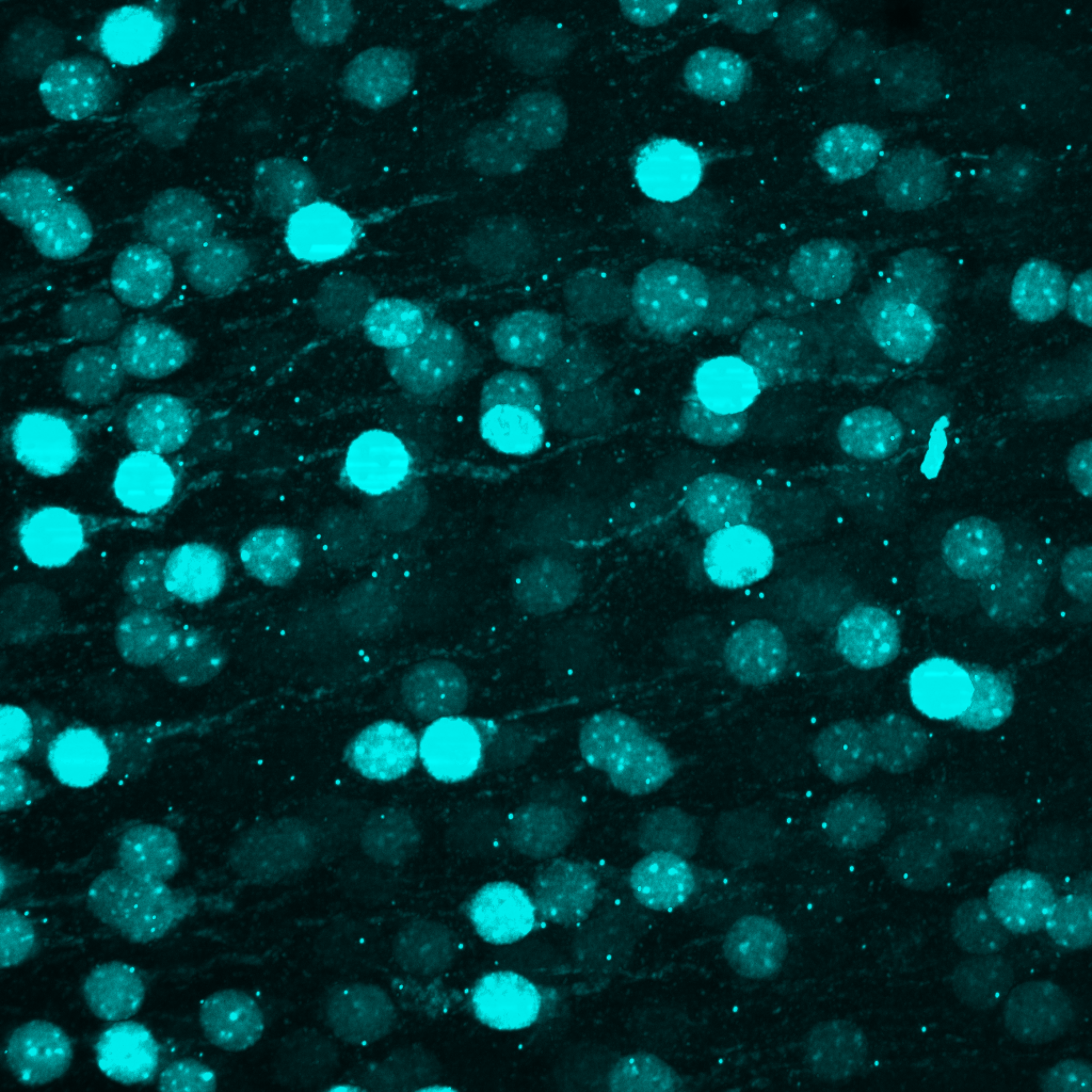

Supplement: Supplementary file 8 — Source data Fig. 1G [file 44318_2025_624_MOESM8_ESM.zip › 1G/n1/CFSE+GFP/CFSE.tif]

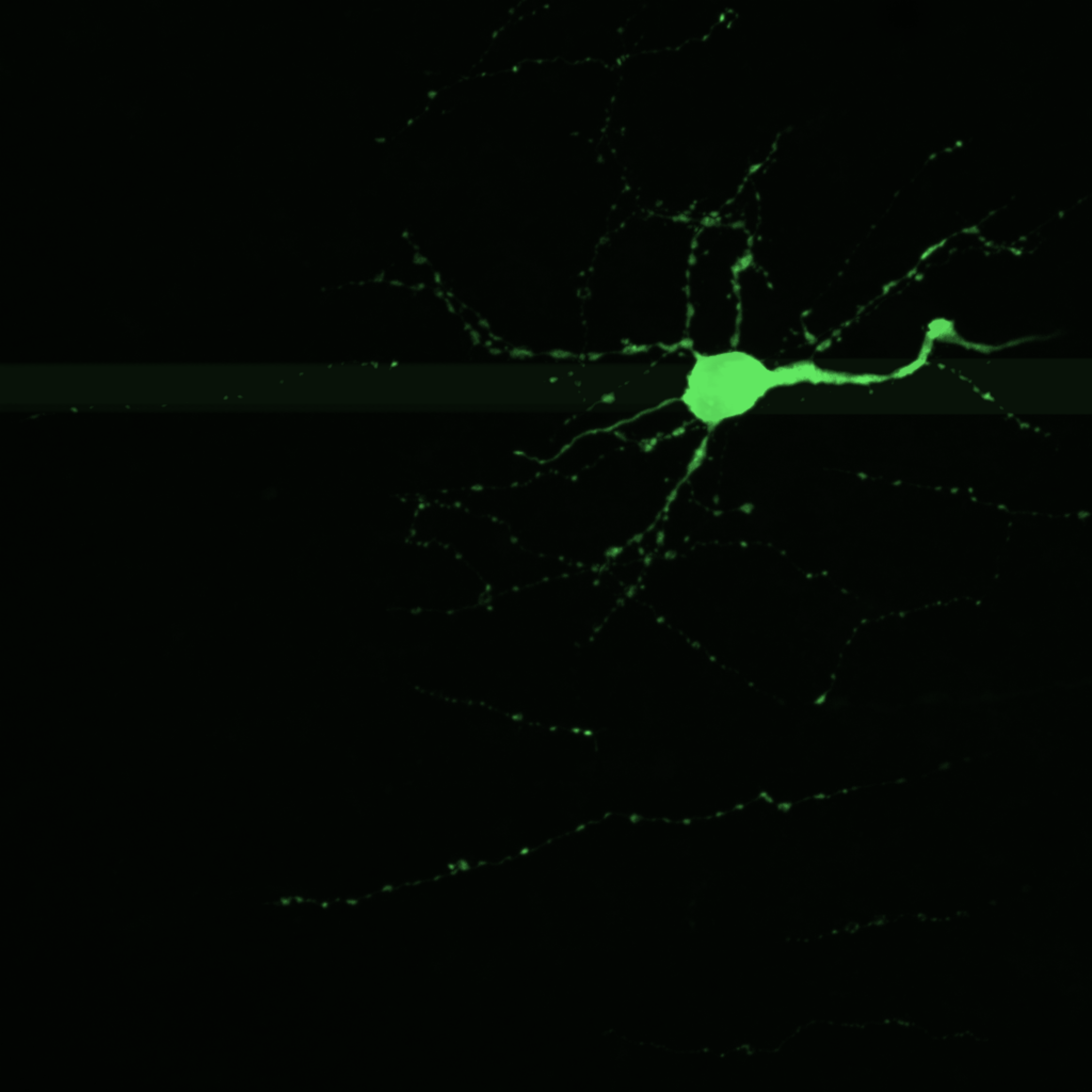

Supplement: Supplementary file 8 — Source data Fig. 1G [file 44318_2025_624_MOESM8_ESM.zip › 1G/n1/CFSE+GFP/GFP.tif]

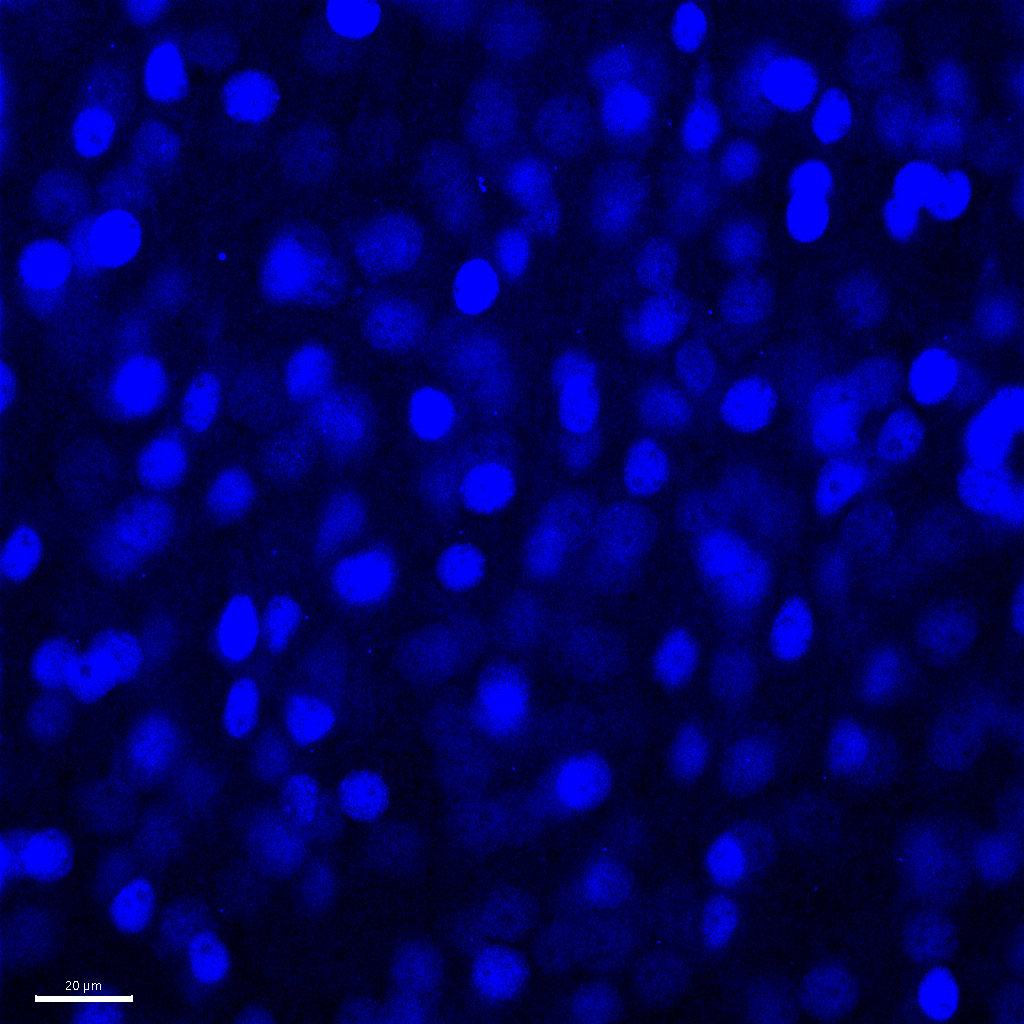

Supplement: Supplementary file 8 — Source data Fig. 1G [file 44318_2025_624_MOESM8_ESM.zip › 1G/n4/satb2+ctip2+fog2/ctip2.tif]

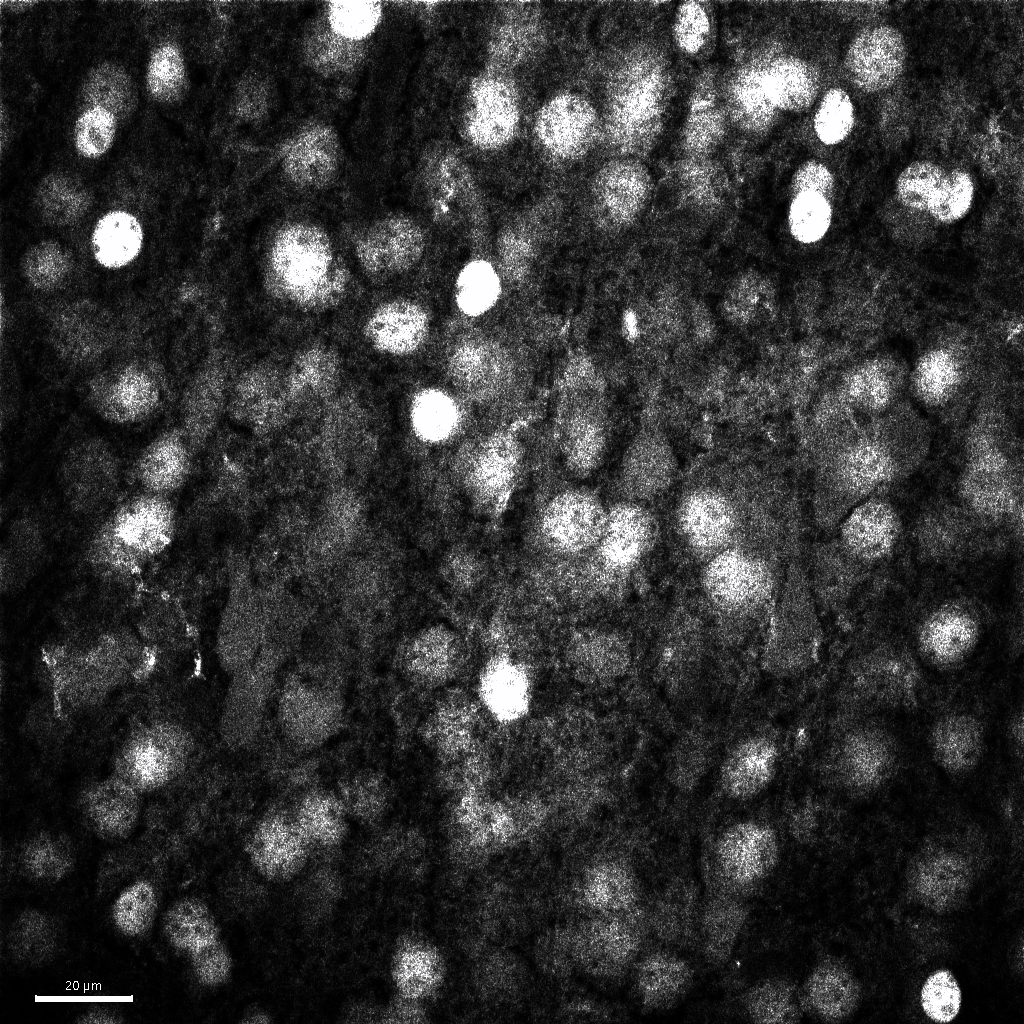

Supplement: Supplementary file 8 — Source data Fig. 1G [file 44318_2025_624_MOESM8_ESM.zip › 1G/n4/satb2+ctip2+fog2/satb2.tif]

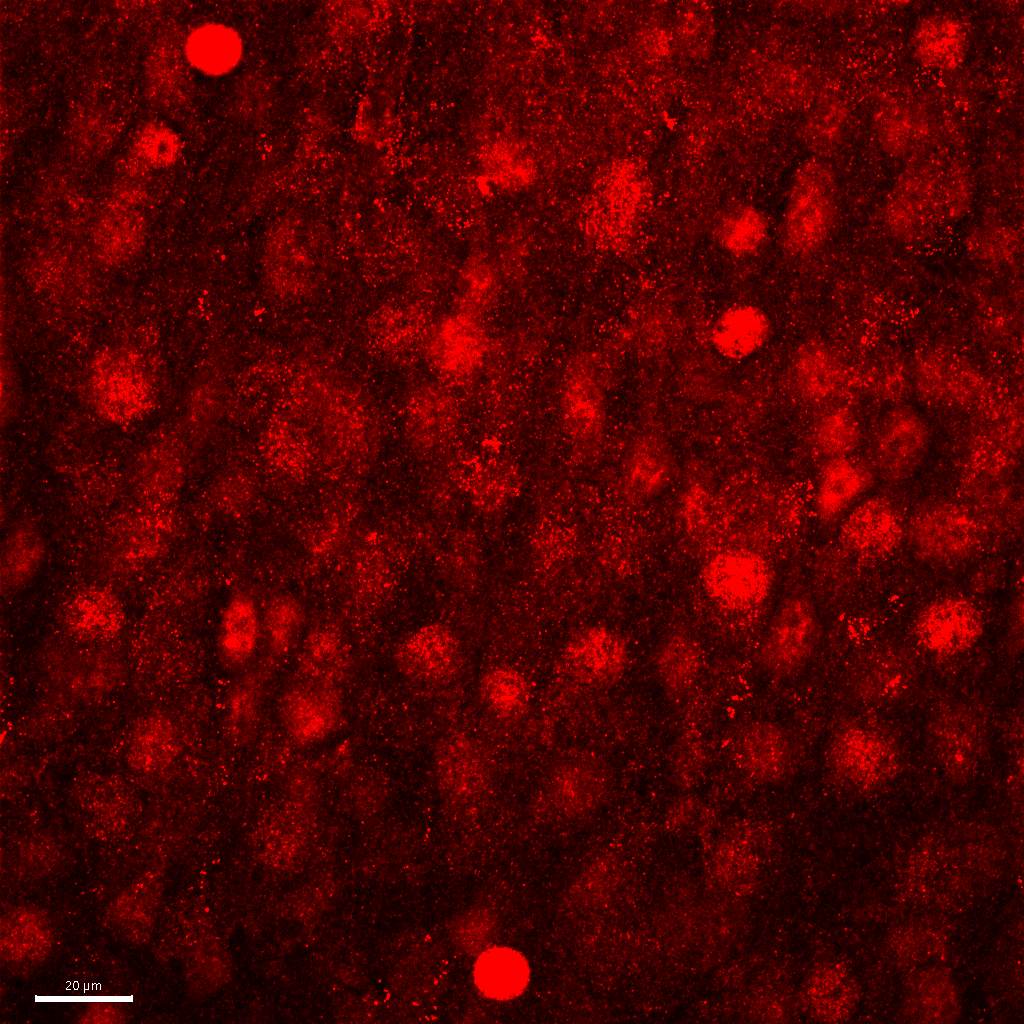

Supplement: Supplementary file 8 — Source data Fig. 1G [file 44318_2025_624_MOESM8_ESM.zip › 1G/n4/satb2+ctip2+fog2/fog2.tif]

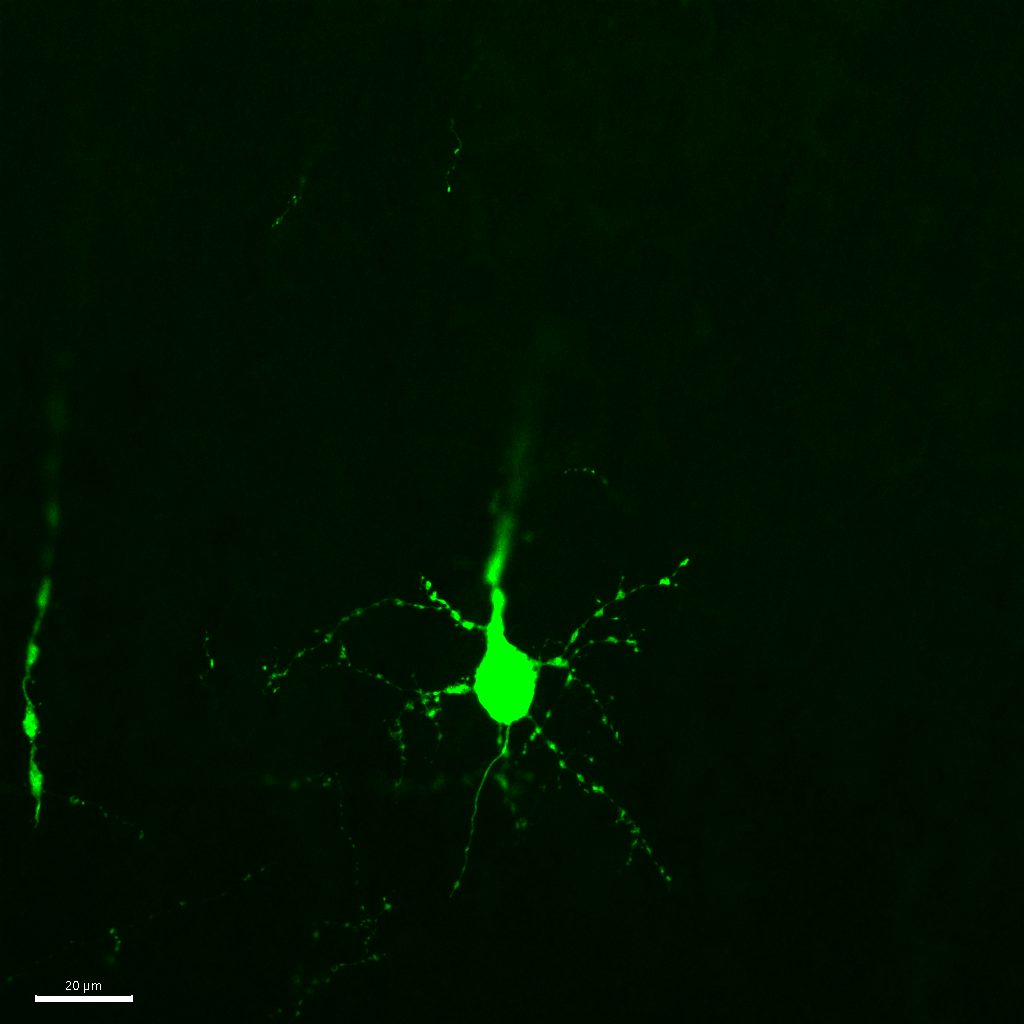

Supplement: Supplementary file 8 — Source data Fig. 1G [file 44318_2025_624_MOESM8_ESM.zip › 1G/n4/satb2+ctip2+fog2/GFP.tif]

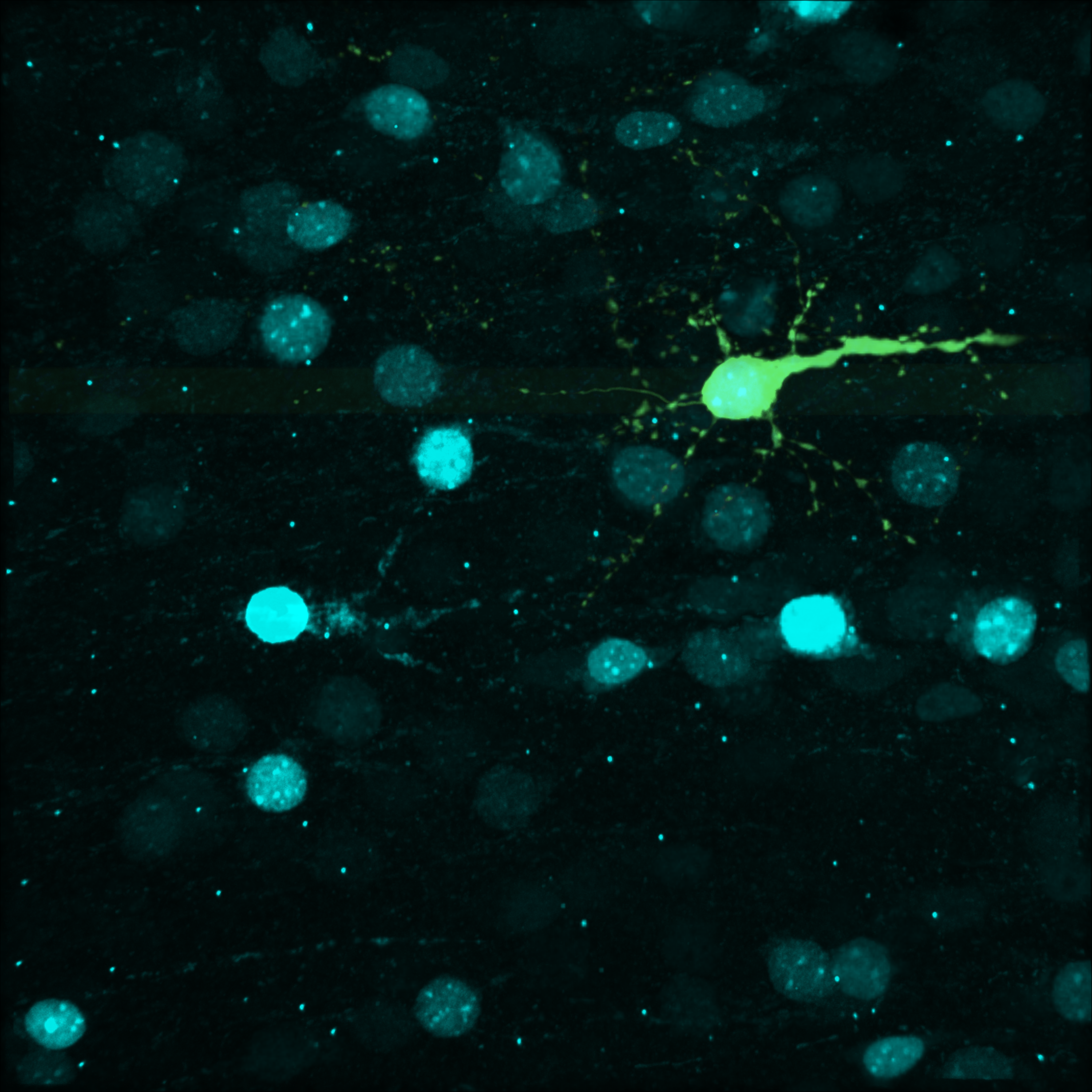

Supplement: Supplementary file 8 — Source data Fig. 1G [file 44318_2025_624_MOESM8_ESM.zip › 1G/n4/CFSE+GFP/CFSE+GFP.tif]

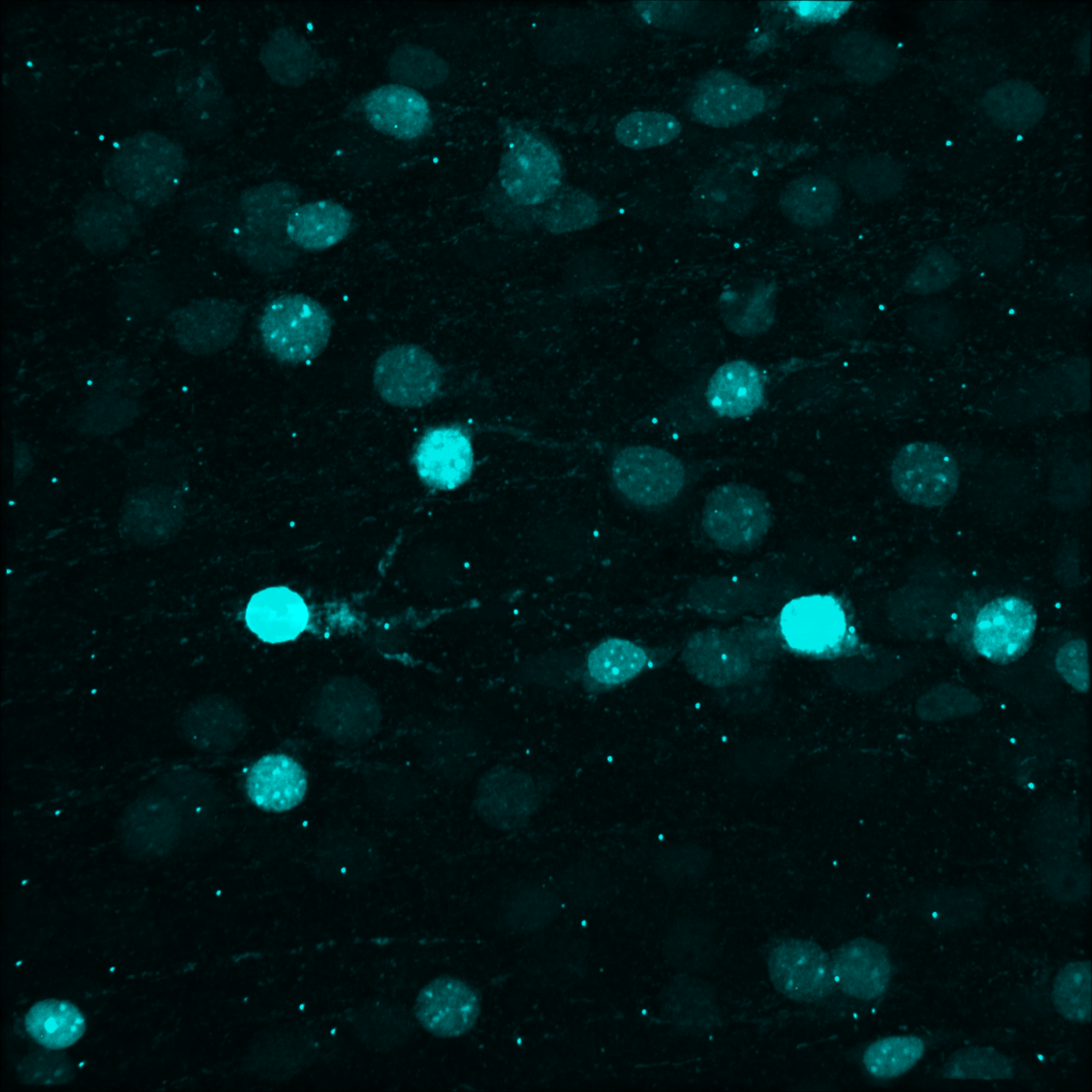

Supplement: Supplementary file 8 — Source data Fig. 1G [file 44318_2025_624_MOESM8_ESM.zip › 1G/n4/CFSE+GFP/CFSE.tif]

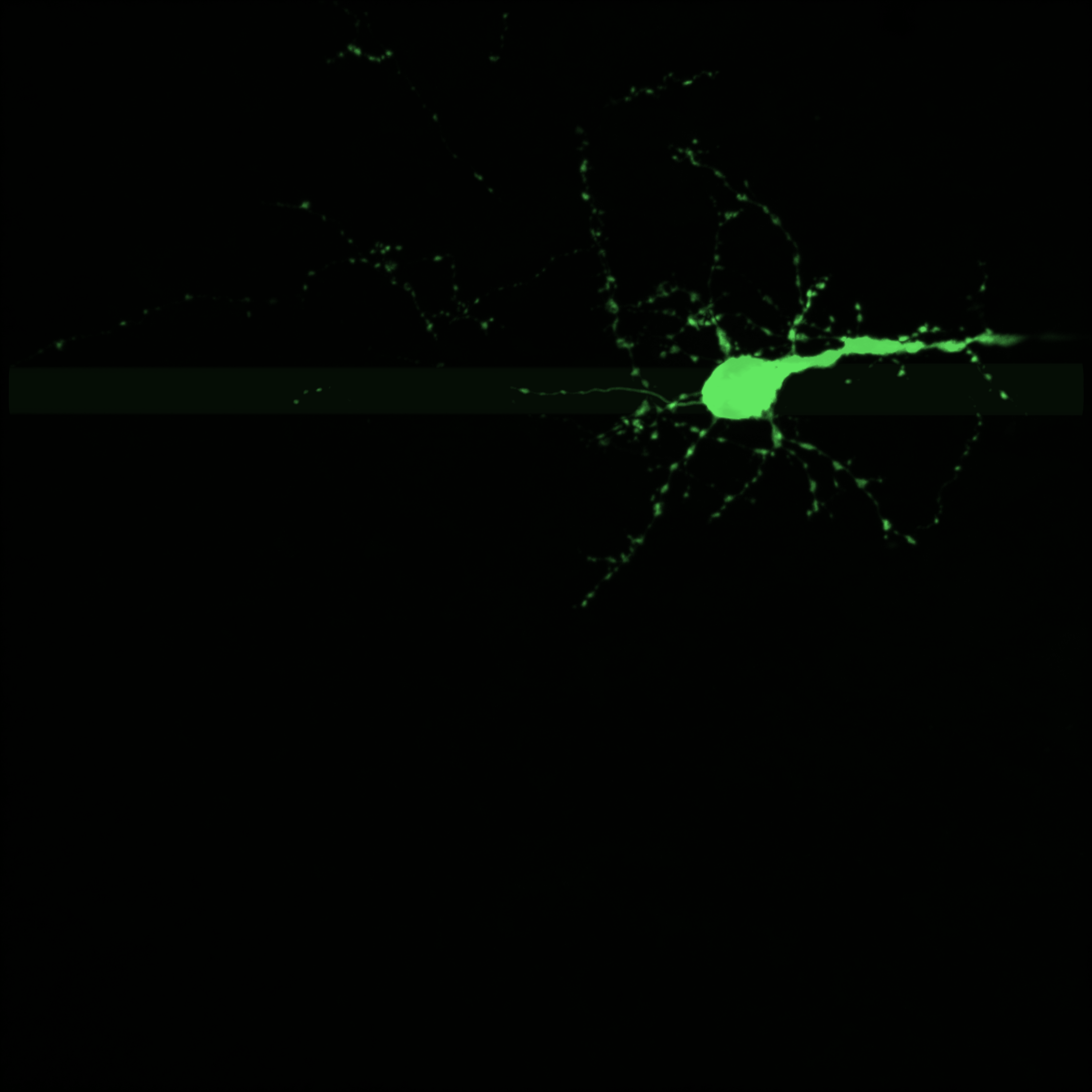

Supplement: Supplementary file 8 — Source data Fig. 1G [file 44318_2025_624_MOESM8_ESM.zip › 1G/n4/CFSE+GFP/GFP.tif]

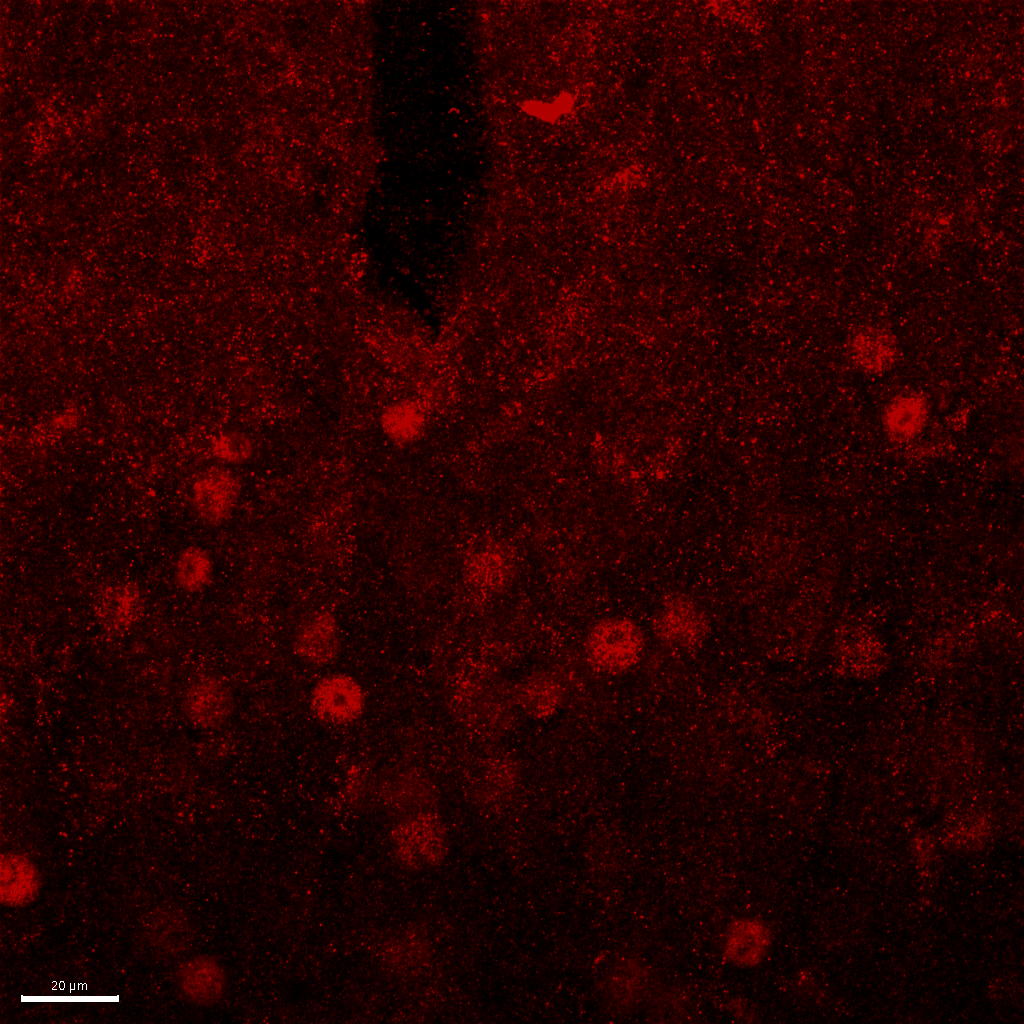

Supplement: Supplementary file 8 — Source data Fig. 1G [file 44318_2025_624_MOESM8_ESM.zip › 1G/n3/satb2+ctip2+fog2/60X CELL6_2025-05-14T15-44-36.300.tif]

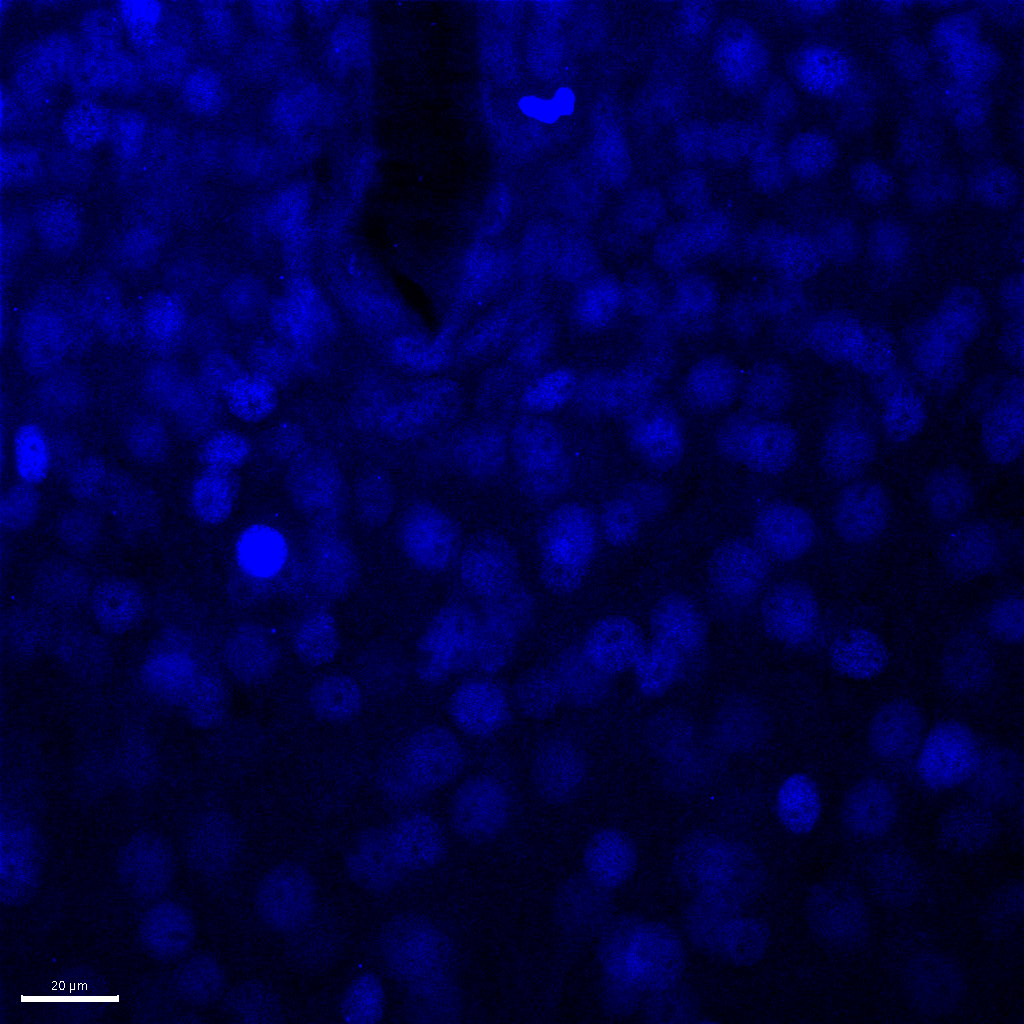

Supplement: Supplementary file 8 — Source data Fig. 1G [file 44318_2025_624_MOESM8_ESM.zip › 1G/n3/satb2+ctip2+fog2/60X CELL6_2025-05-14T15-44-33.320.tif]

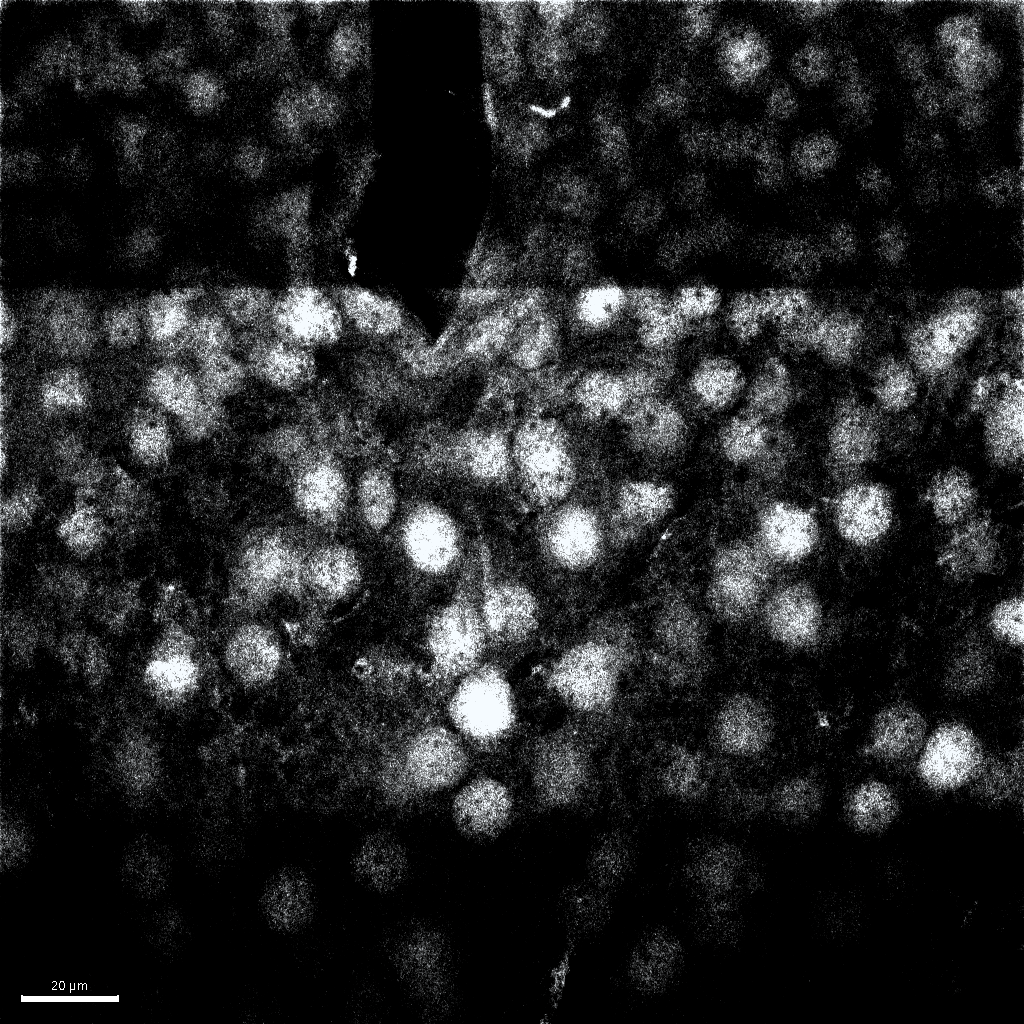

Supplement: Supplementary file 8 — Source data Fig. 1G [file 44318_2025_624_MOESM8_ESM.zip › 1G/n3/satb2+ctip2+fog2/60X CELL6_2025-05-14T15-44-27.581.tif]

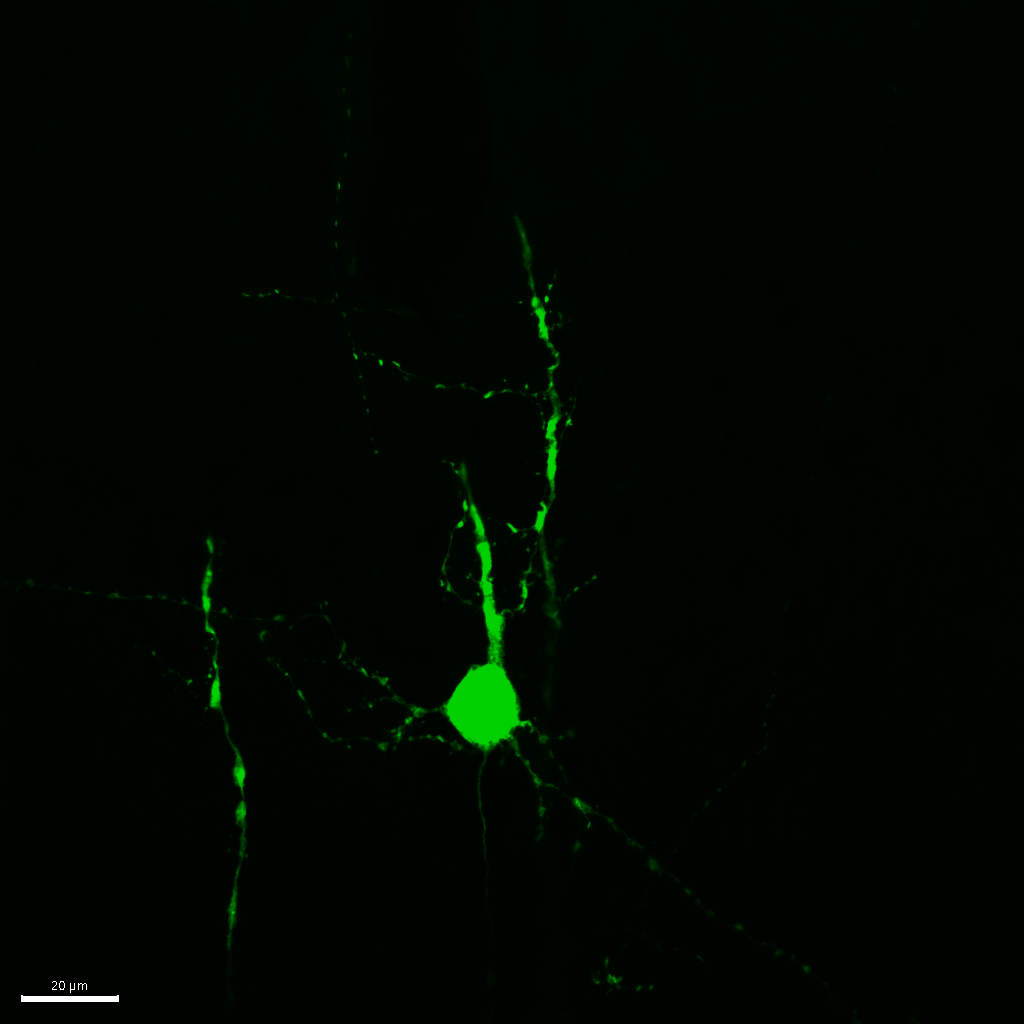

Supplement: Supplementary file 8 — Source data Fig. 1G [file 44318_2025_624_MOESM8_ESM.zip › 1G/n3/satb2+ctip2+fog2/60X CELL6_2025-05-14T15-44-30.539.tif]

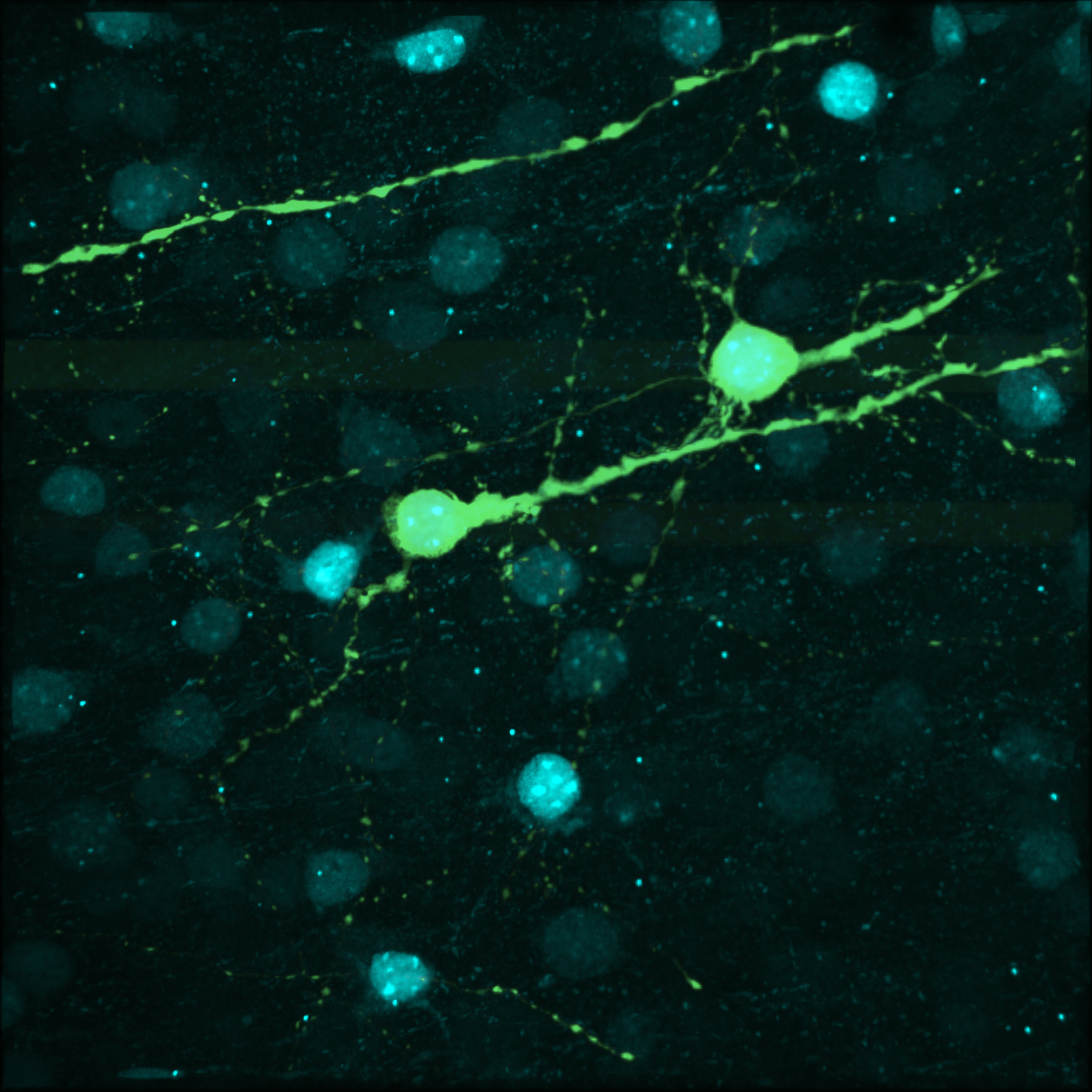

Supplement: Supplementary file 8 — Source data Fig. 1G [file 44318_2025_624_MOESM8_ESM.zip › 1G/n3/CFSE+GFP/11_1_2025-07-11T20-26-55.658.tif]

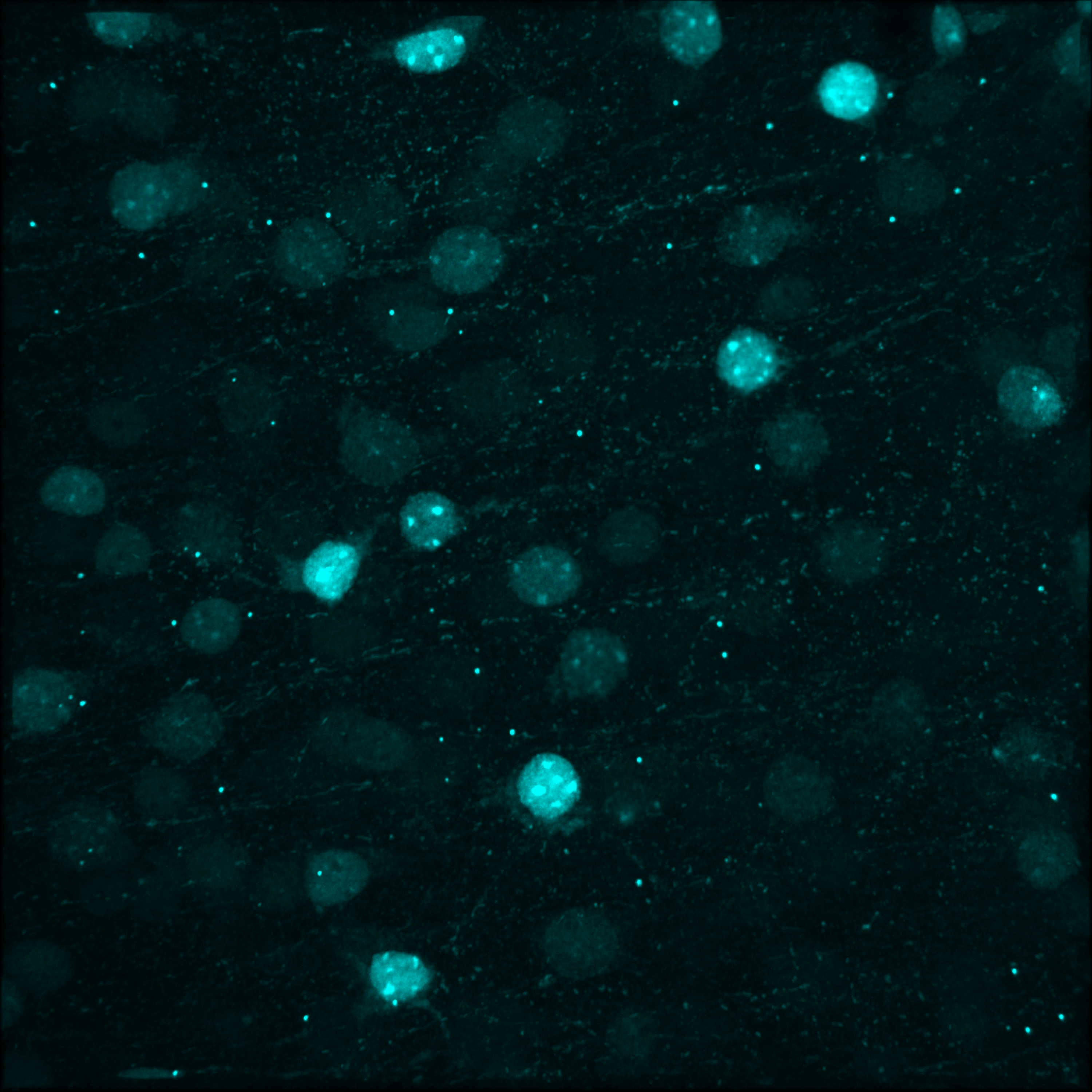

Supplement: Supplementary file 8 — Source data Fig. 1G [file 44318_2025_624_MOESM8_ESM.zip › 1G/n3/CFSE+GFP/11_1_2025-07-11T20-26-42.489.tif]

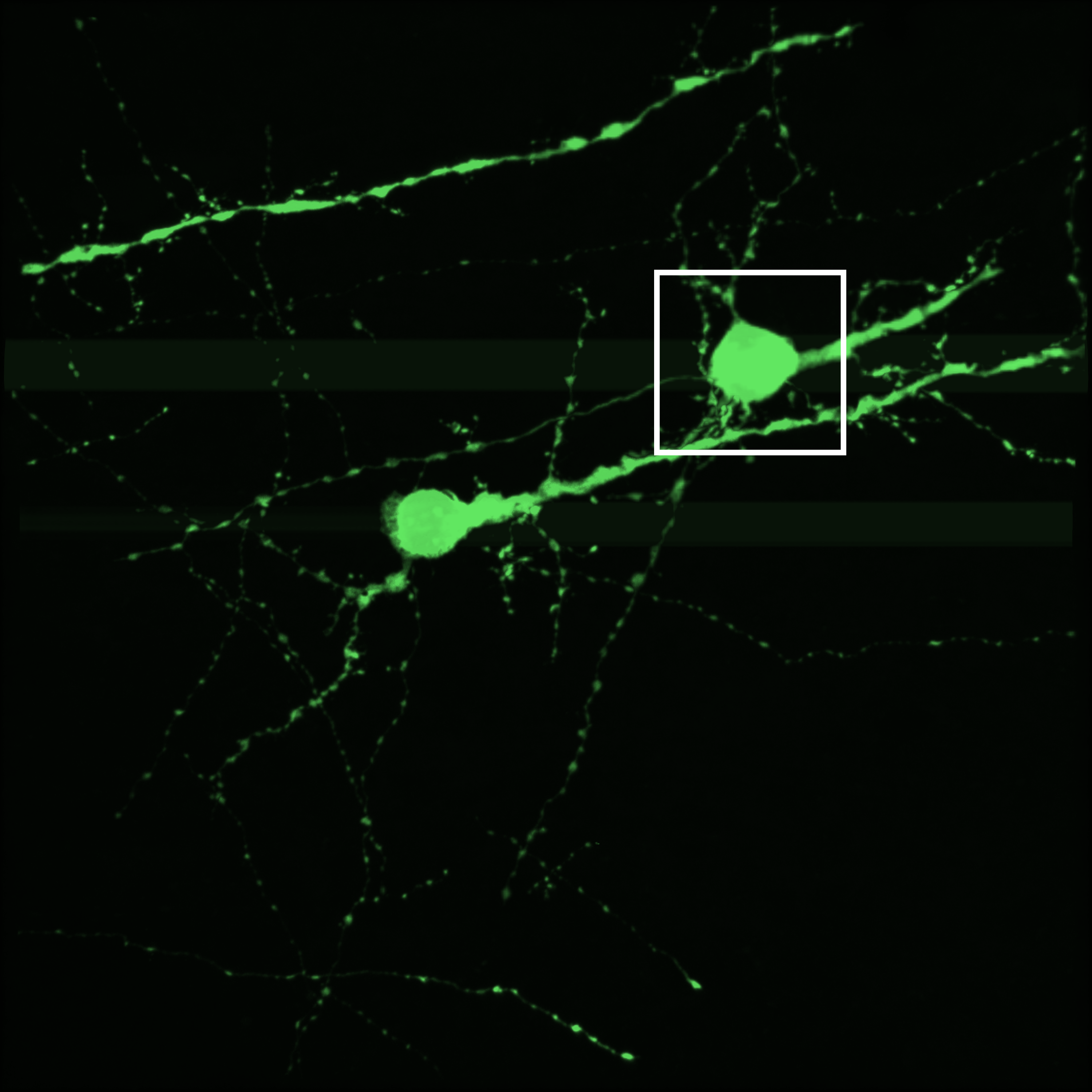

Supplement: Supplementary file 8 — Source data Fig. 1G [file 44318_2025_624_MOESM8_ESM.zip › 1G/n3/CFSE+GFP/11_1_2025-07-11T20-26-49.893.tif]

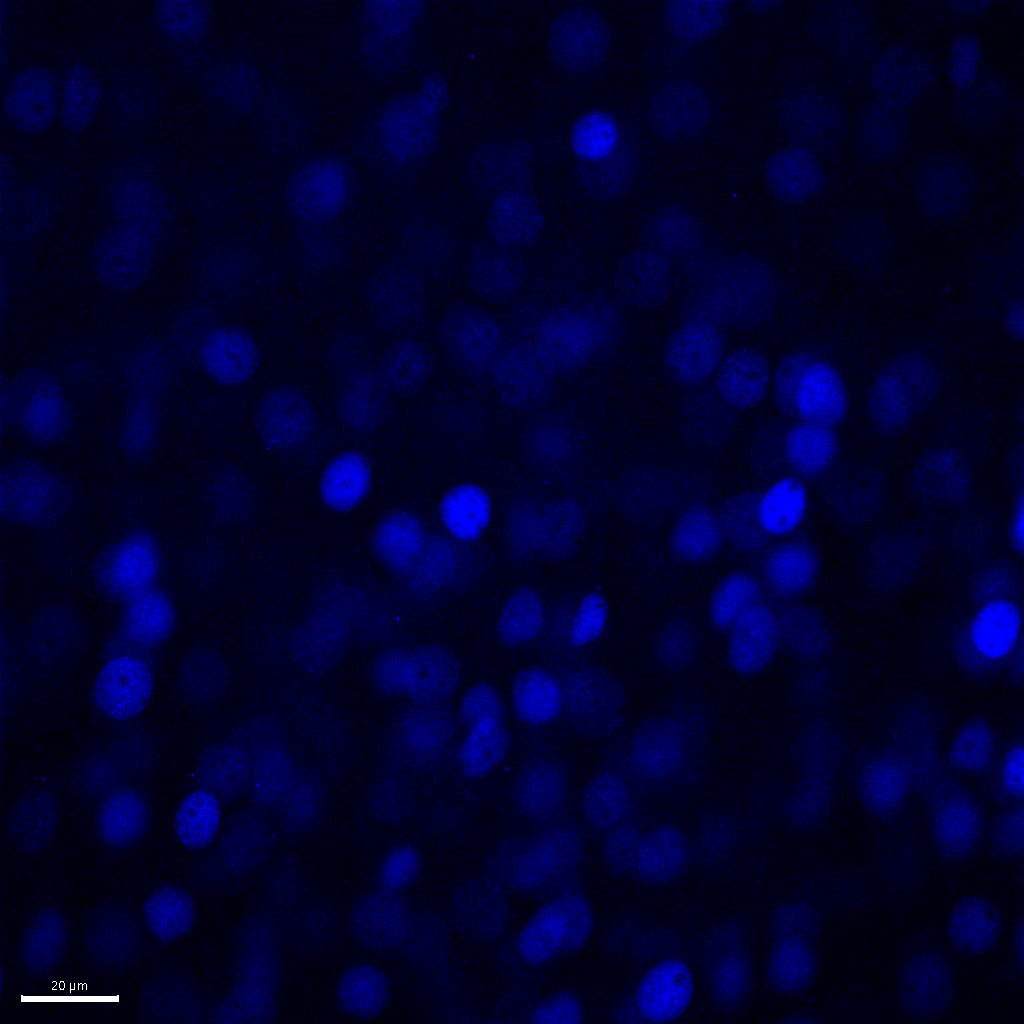

Supplement: Supplementary file 8 — Source data Fig. 1G [file 44318_2025_624_MOESM8_ESM.zip › 1G/n2/satb2+ctip2+fog2/ctip2.tif]

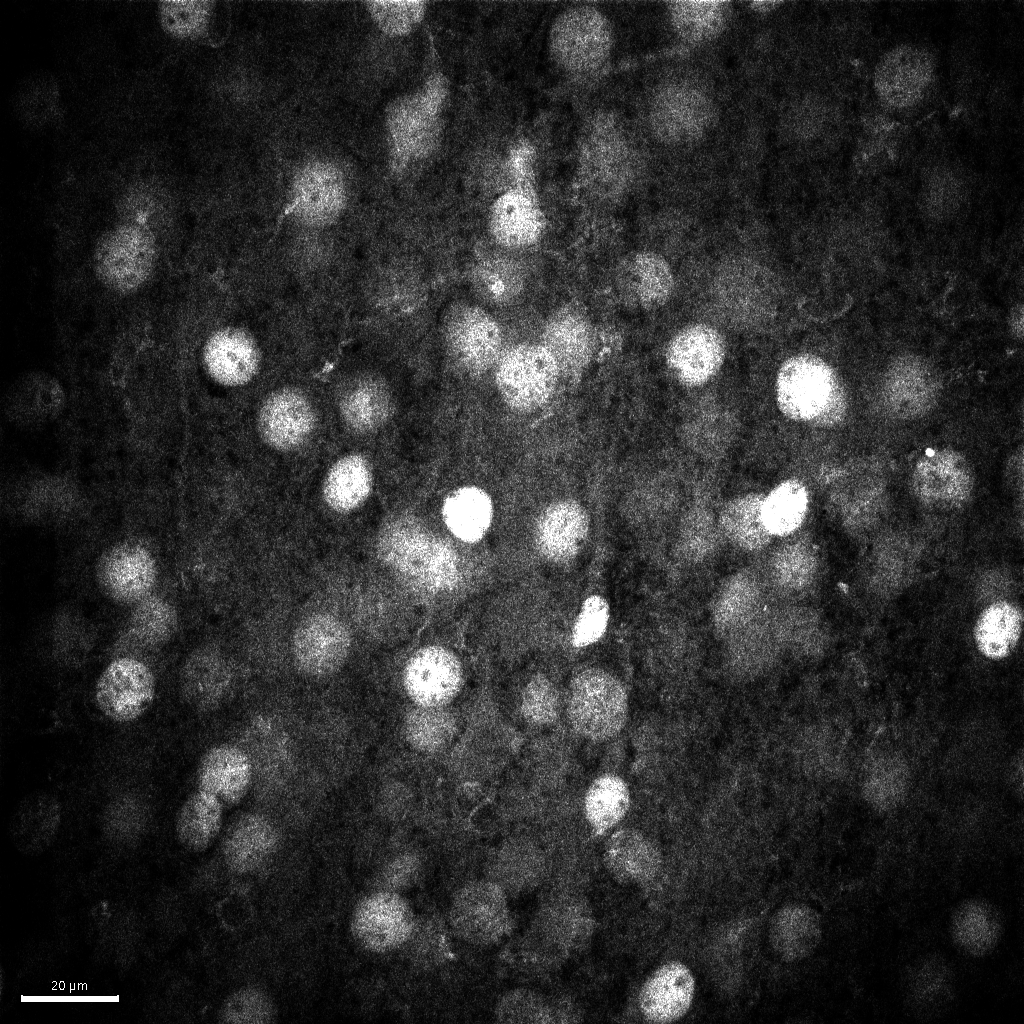

Supplement: Supplementary file 8 — Source data Fig. 1G [file 44318_2025_624_MOESM8_ESM.zip › 1G/n2/satb2+ctip2+fog2/satb2.tif]

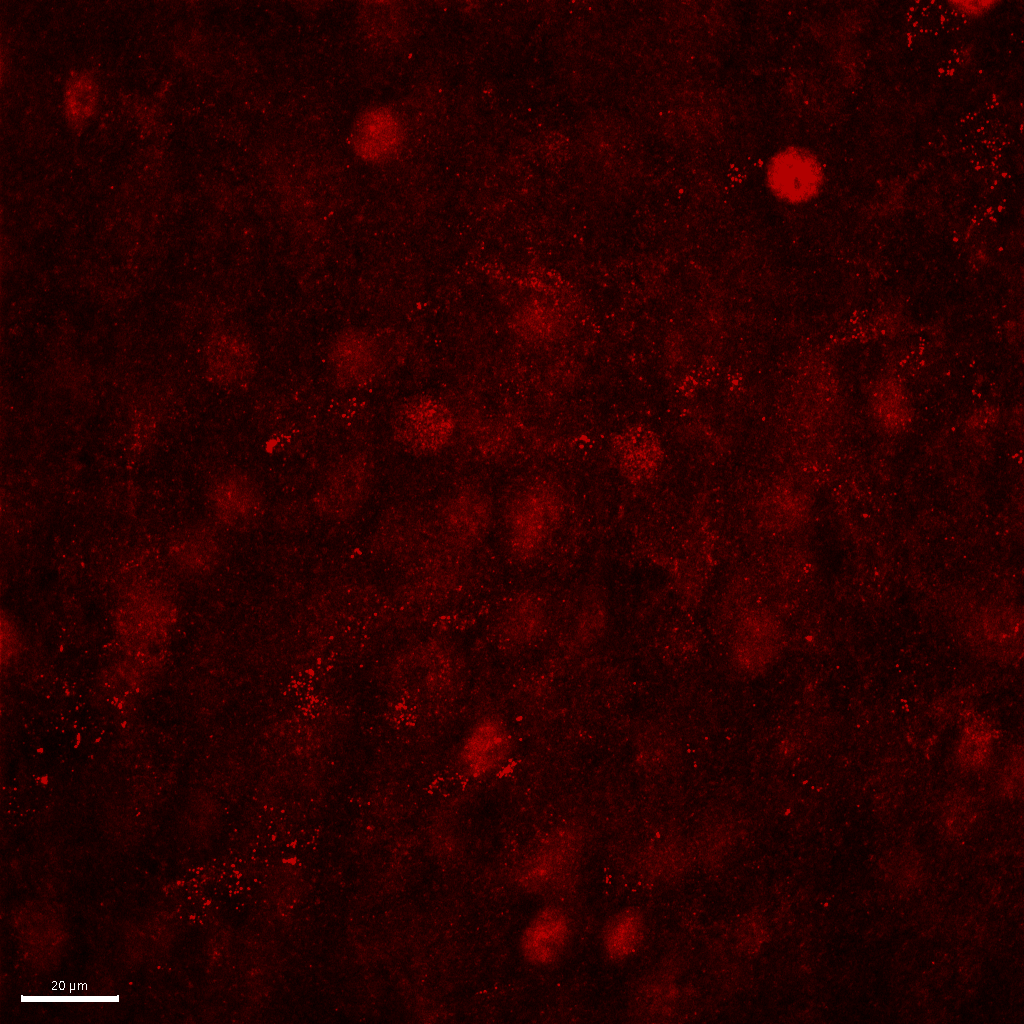

Supplement: Supplementary file 8 — Source data Fig. 1G [file 44318_2025_624_MOESM8_ESM.zip › 1G/n2/satb2+ctip2+fog2/fog2.tif]

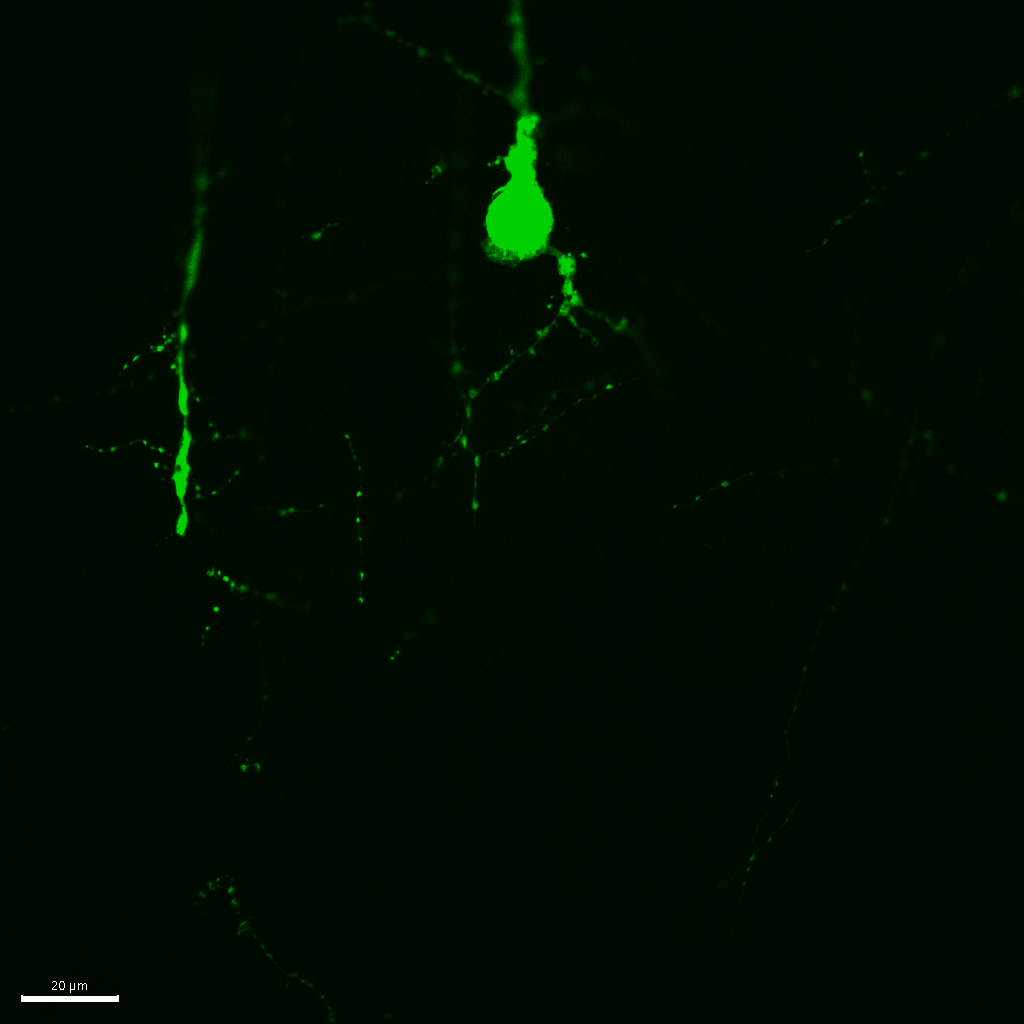

Supplement: Supplementary file 8 — Source data Fig. 1G [file 44318_2025_624_MOESM8_ESM.zip › 1G/n2/satb2+ctip2+fog2/GFP.tif]

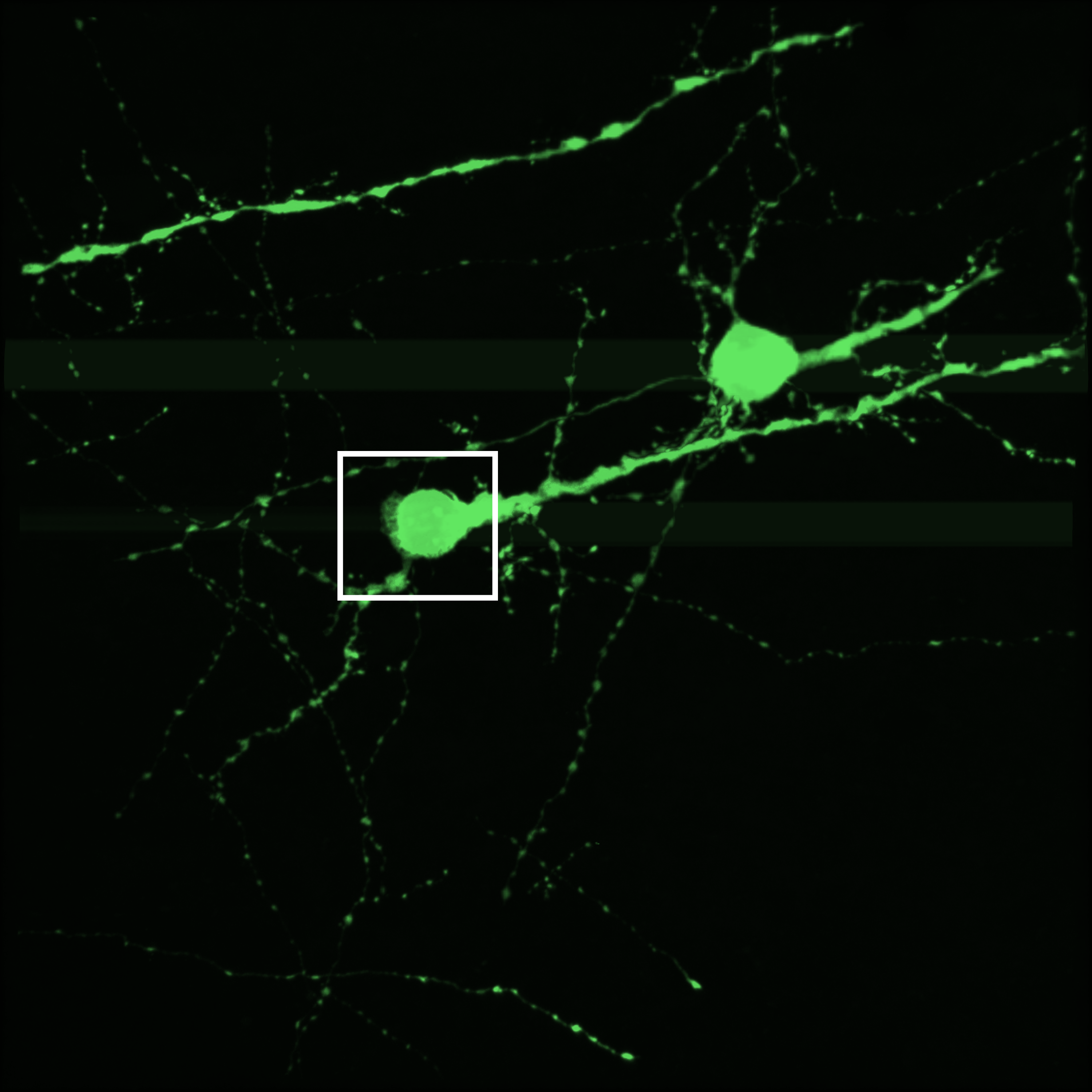

Supplement: Supplementary file 8 — Source data Fig. 1G [file 44318_2025_624_MOESM8_ESM.zip › 1G/n2/CFSE+GFP/GFP.tif]

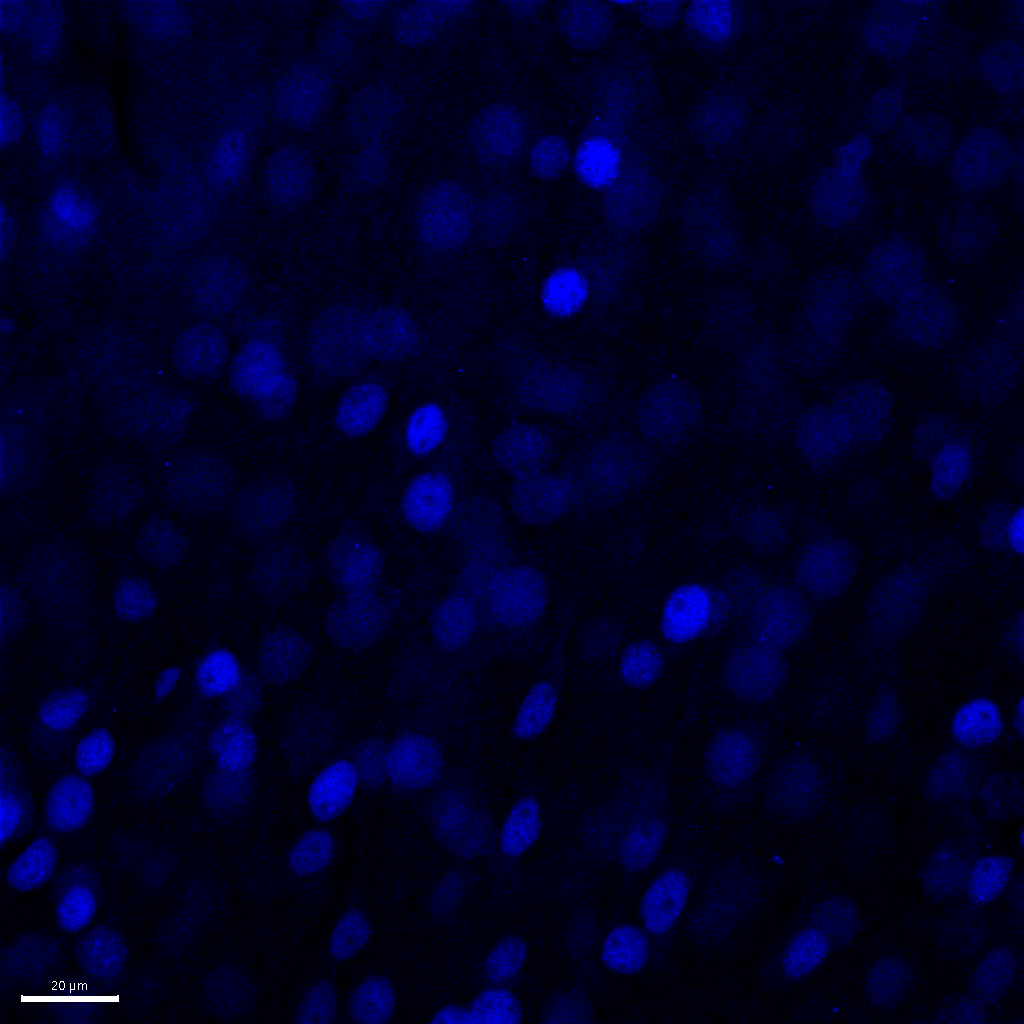

Supplement: Supplementary file 8 — Source data Fig. 1G [file 44318_2025_624_MOESM8_ESM.zip › 1G/n5/satb2+ctip2+fog2/ctip2.tif]

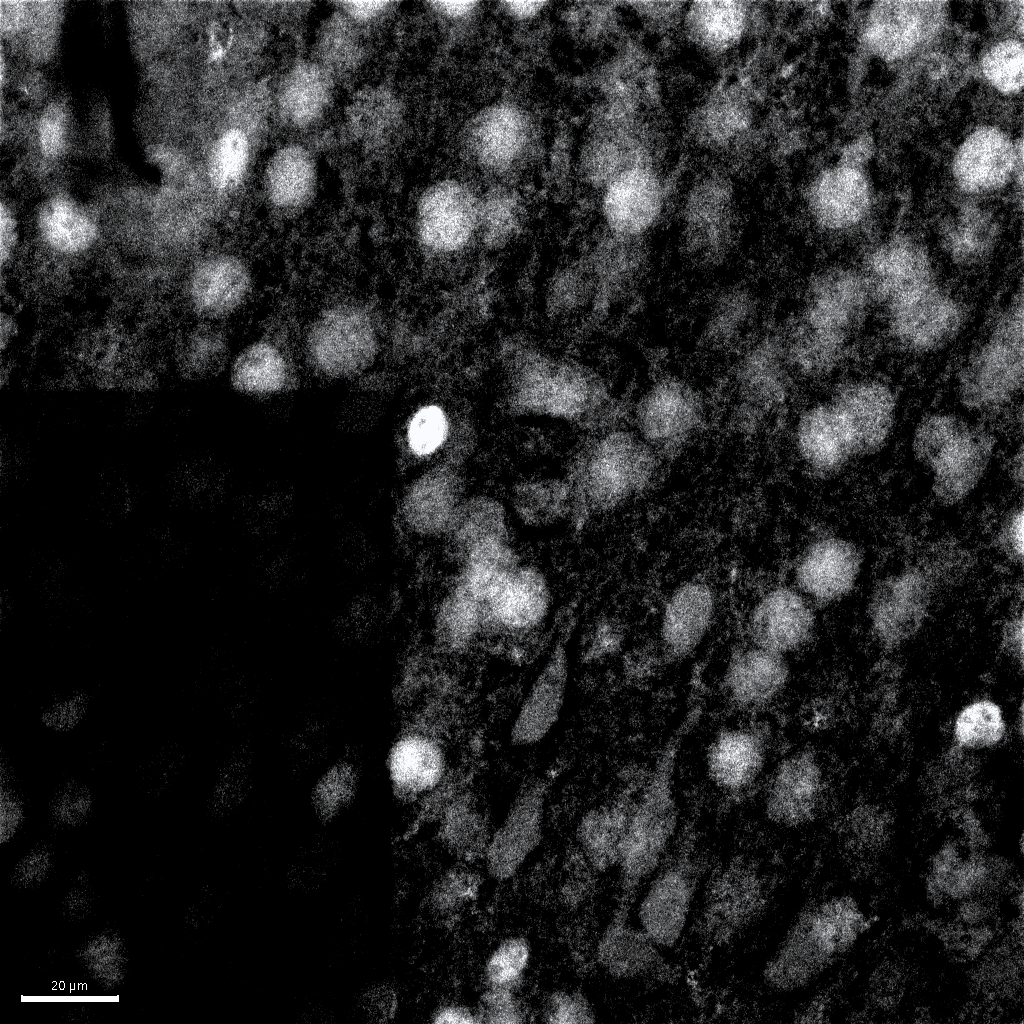

Supplement: Supplementary file 8 — Source data Fig. 1G [file 44318_2025_624_MOESM8_ESM.zip › 1G/n5/satb2+ctip2+fog2/satb2.tif]

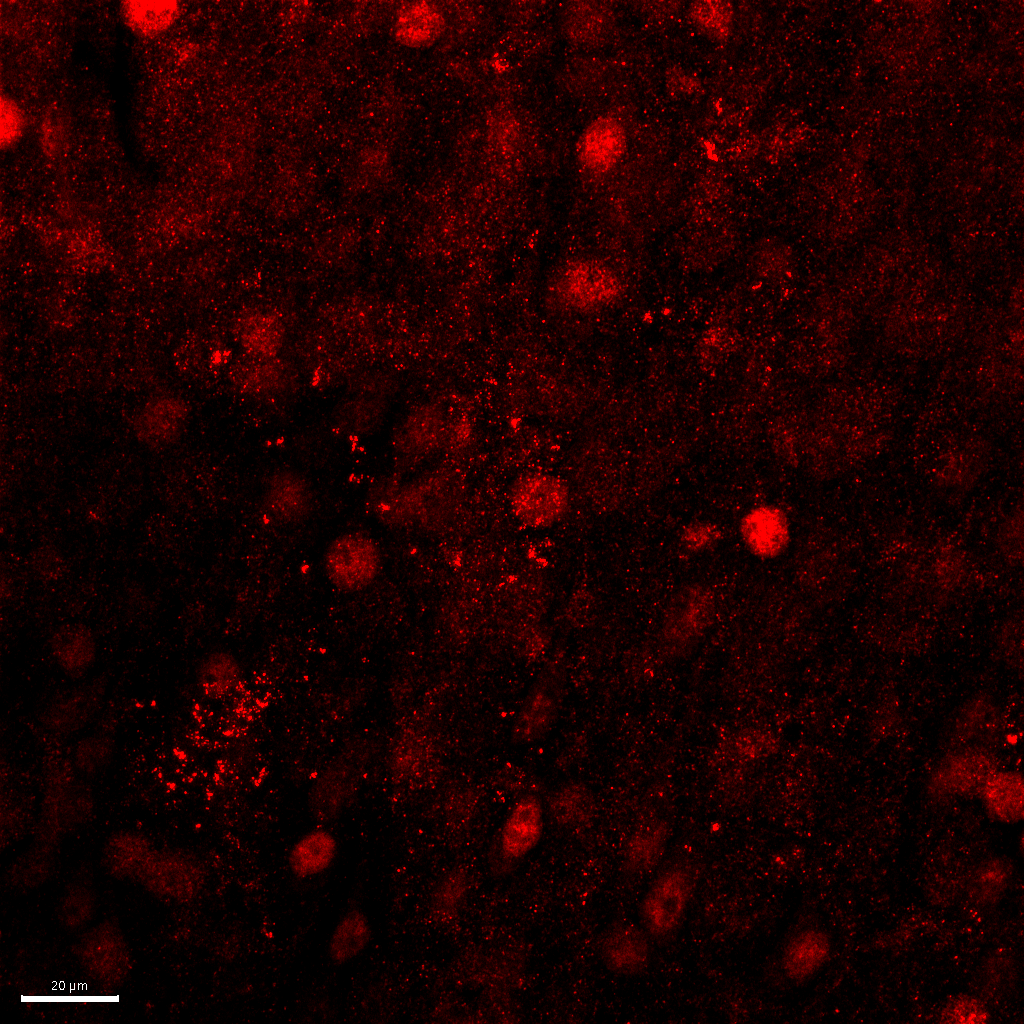

Supplement: Supplementary file 8 — Source data Fig. 1G [file 44318_2025_624_MOESM8_ESM.zip › 1G/n5/satb2+ctip2+fog2/fog2.tif]

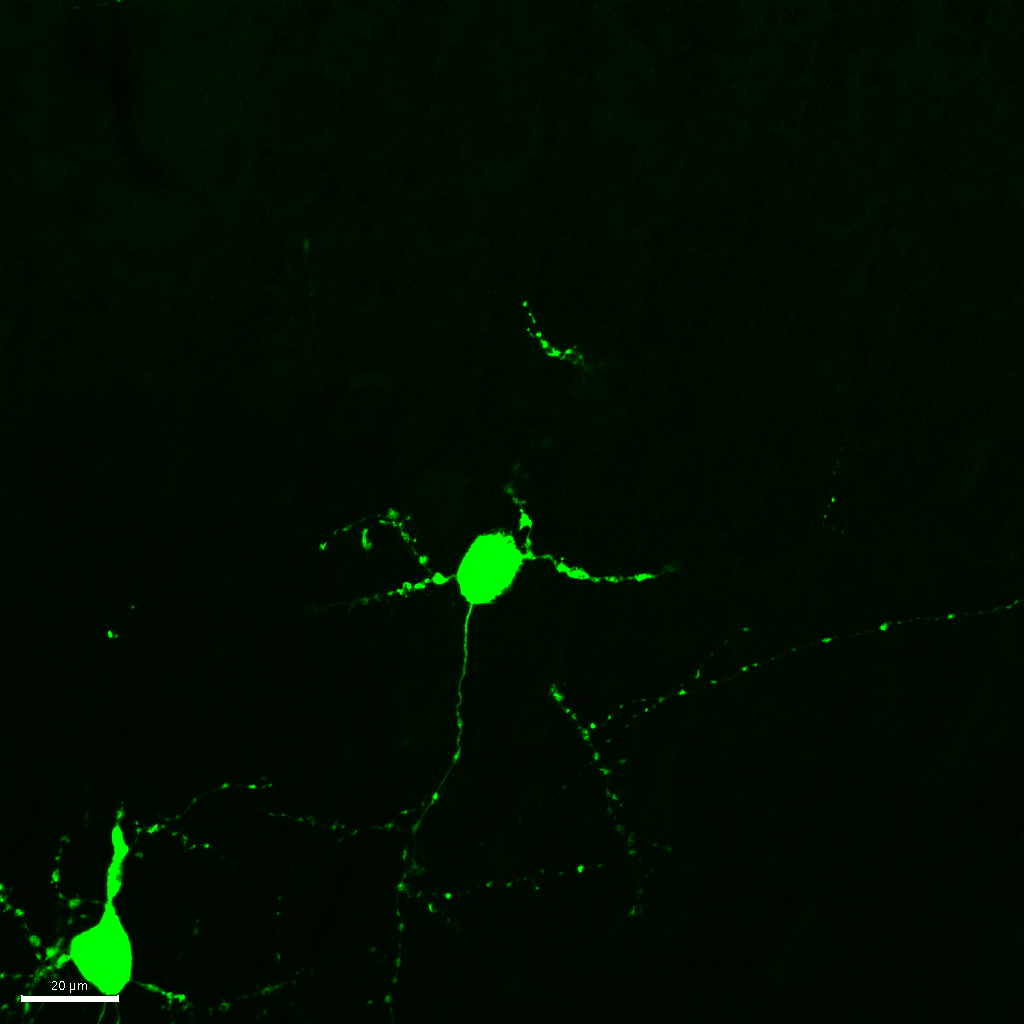

Supplement: Supplementary file 8 — Source data Fig. 1G [file 44318_2025_624_MOESM8_ESM.zip › 1G/n5/satb2+ctip2+fog2/GFP.tif]

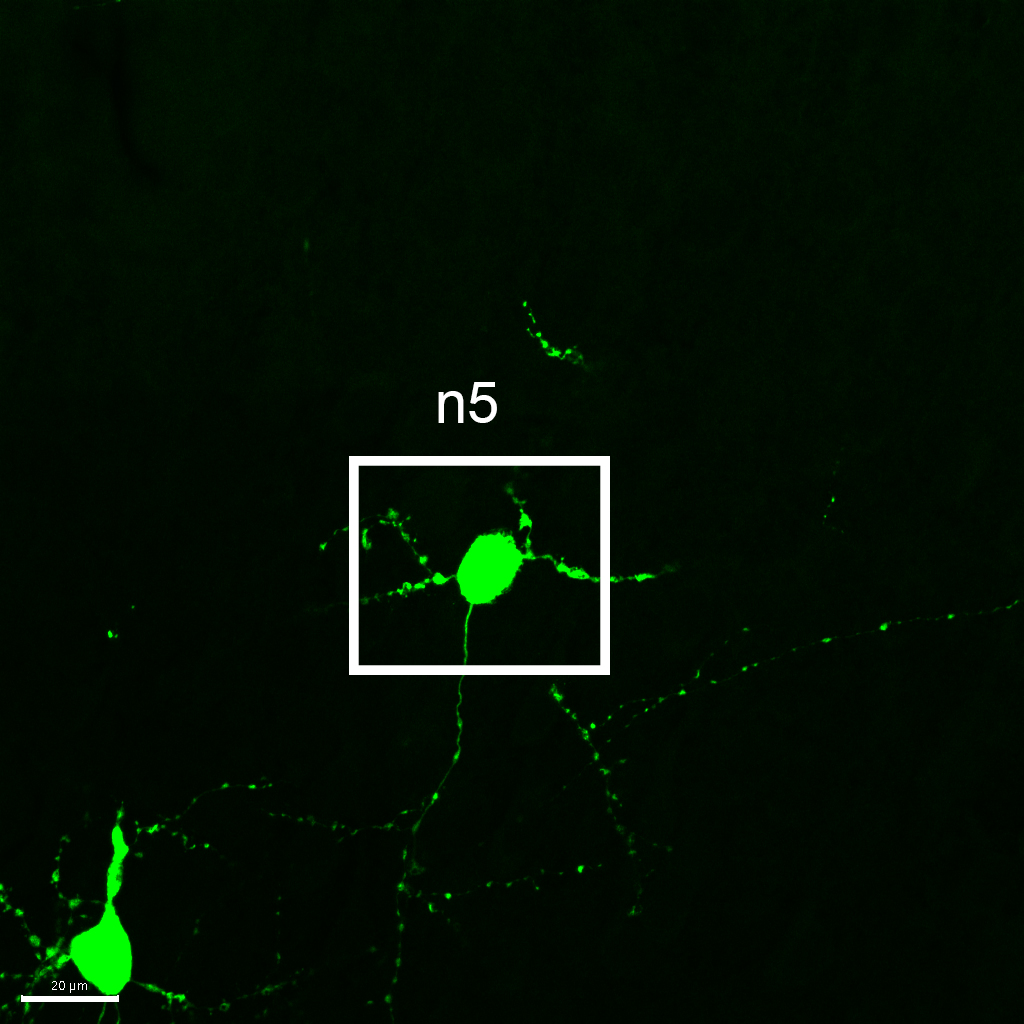

Supplement: Supplementary file 8 — Source data Fig. 1G [file 44318_2025_624_MOESM8_ESM.zip › 1G/n5/satb2+ctip2+fog2/README_The cell in the frame is n5.jpg]

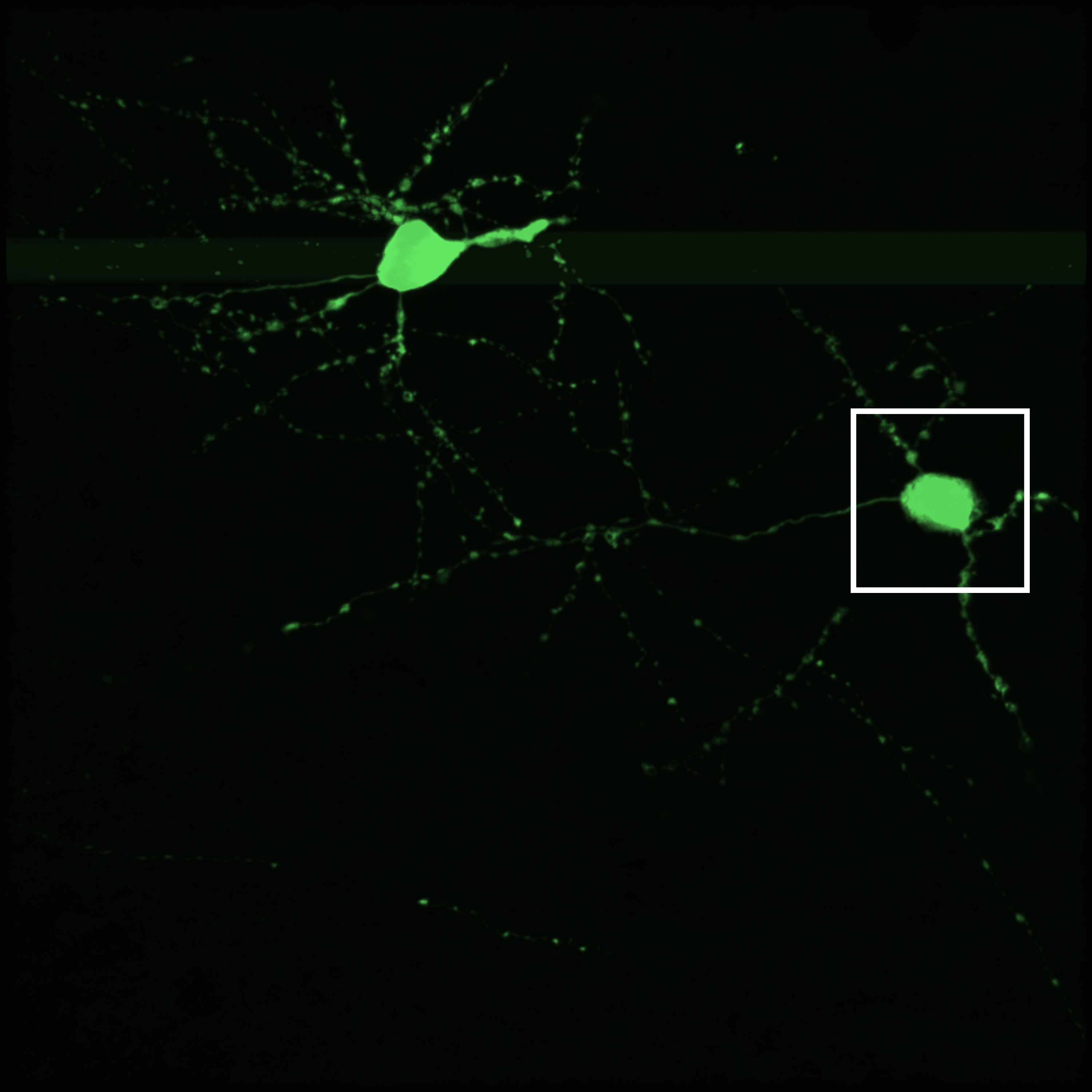

Supplement: Supplementary file 8 — Source data Fig. 1G [file 44318_2025_624_MOESM8_ESM.zip › 1G/n5/CFSE+GFP/GFP.tif]

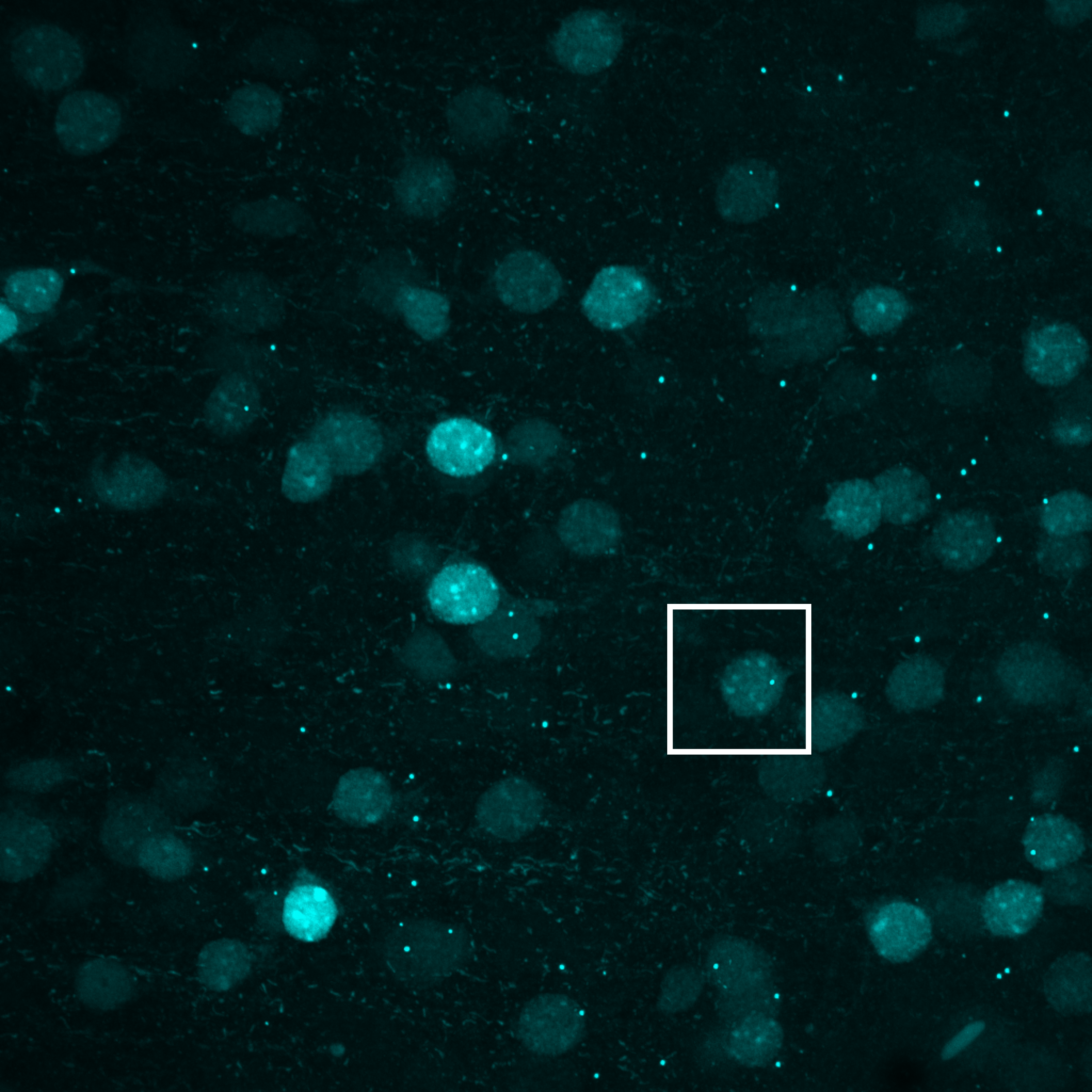

Supplement: Supplementary file 9 — Source data Fig. 1H [file 44318_2025_624_MOESM9_ESM.zip › 1H/n7/CFSE.tif]

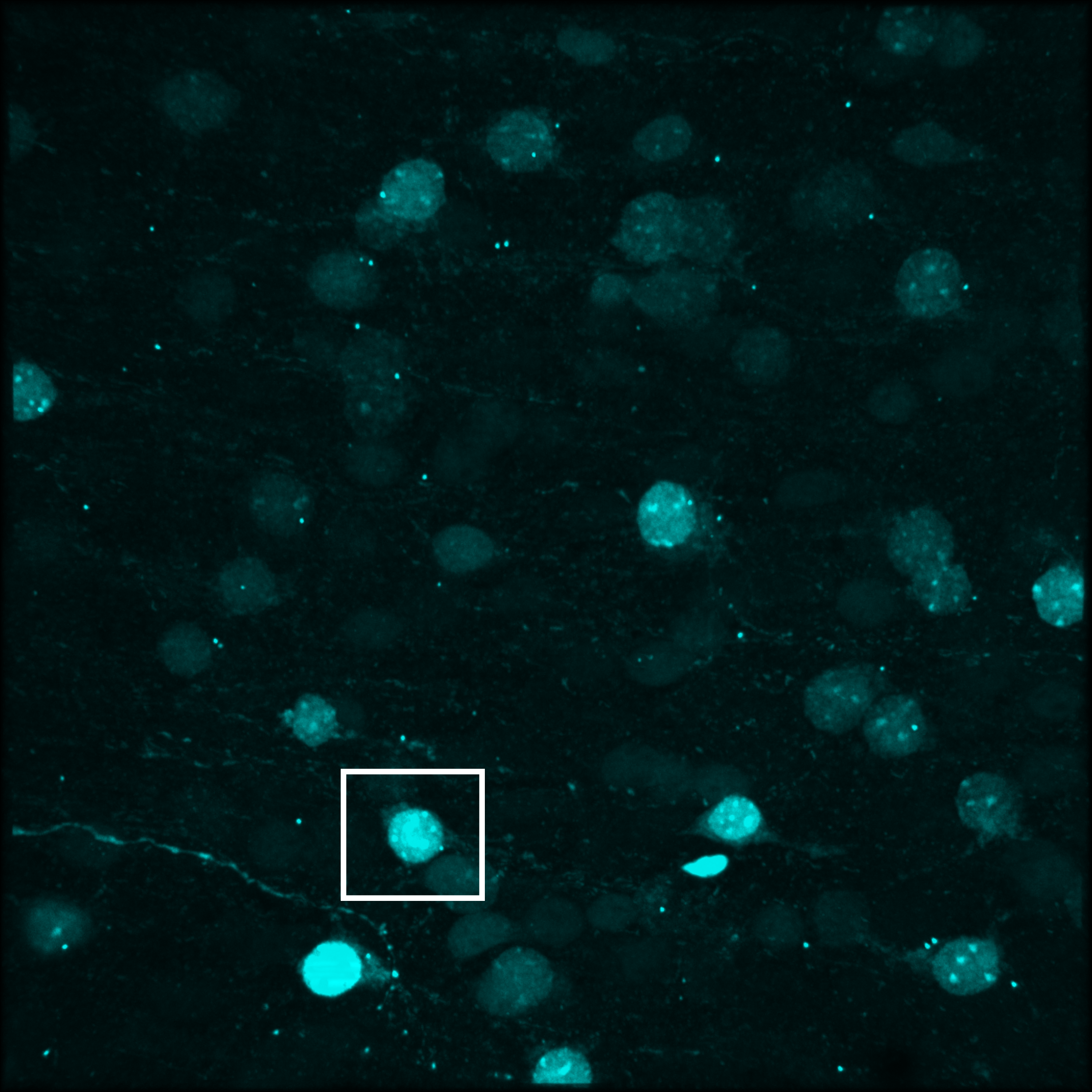

Supplement: Supplementary file 9 — Source data Fig. 1H [file 44318_2025_624_MOESM9_ESM.zip › 1H/n6/CFSE.tif]

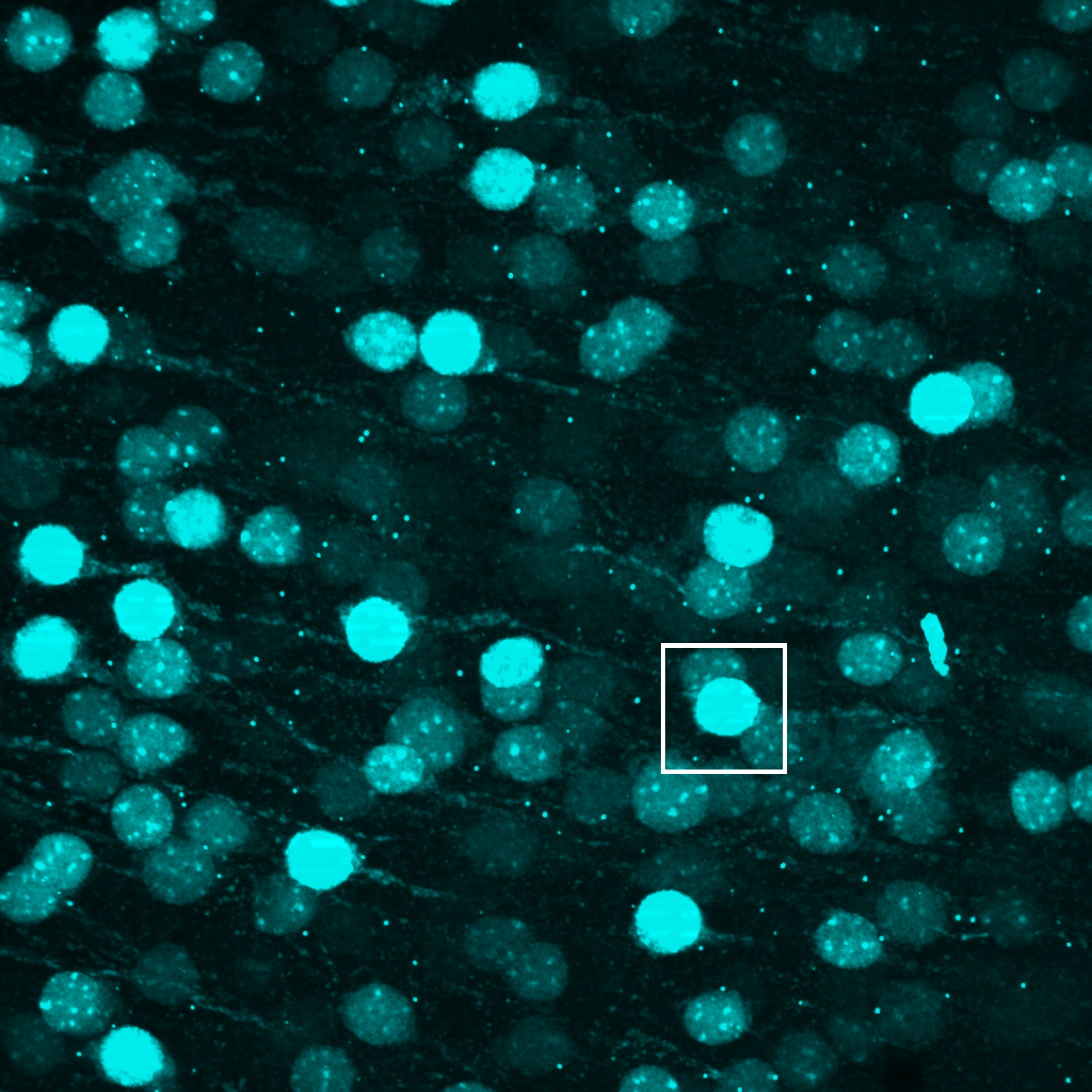

Supplement: Supplementary file 9 — Source data Fig. 1H [file 44318_2025_624_MOESM9_ESM.zip › 1H/n1/CFSE.tif]

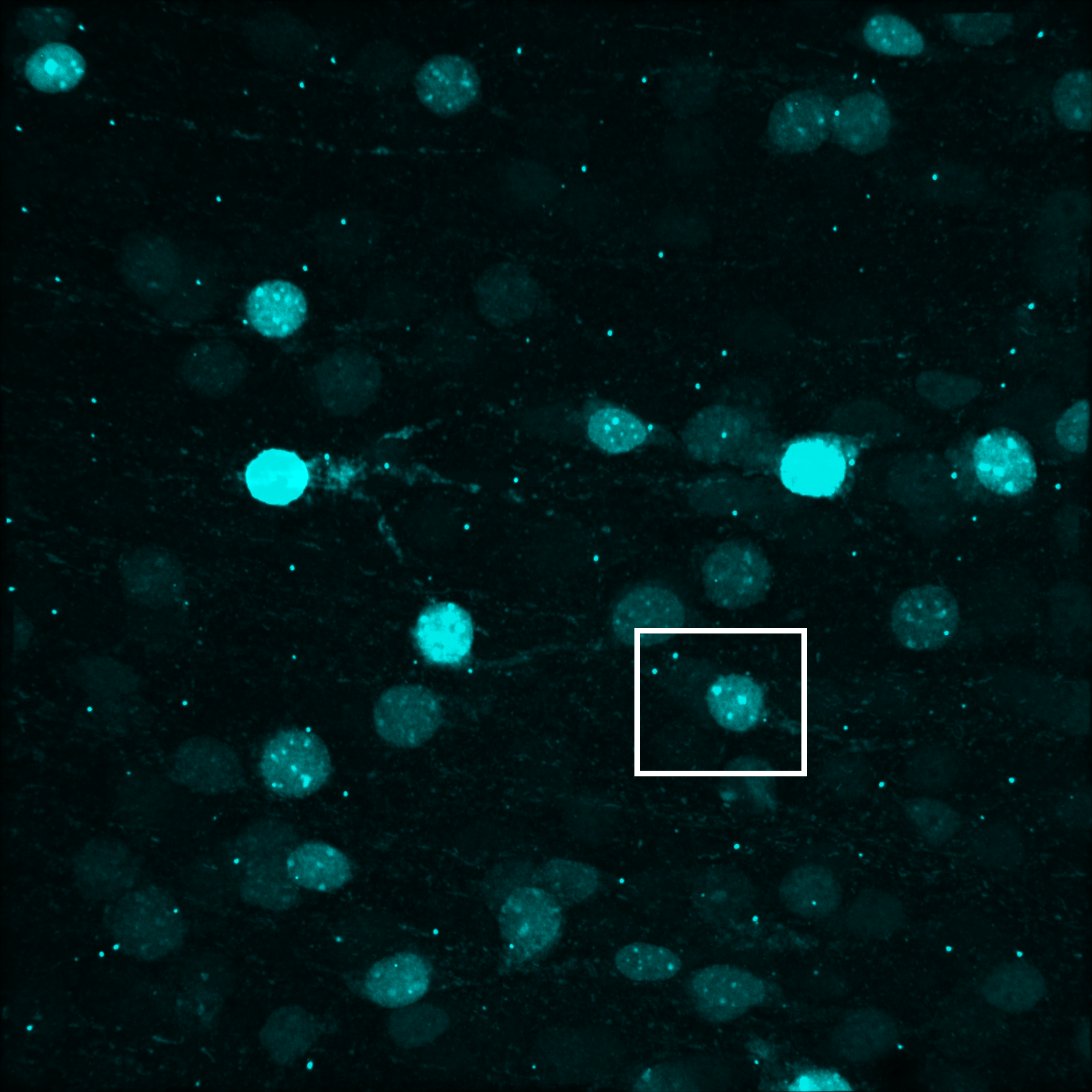

Supplement: Supplementary file 9 — Source data Fig. 1H [file 44318_2025_624_MOESM9_ESM.zip › 1H/n4/CFSE.tif]

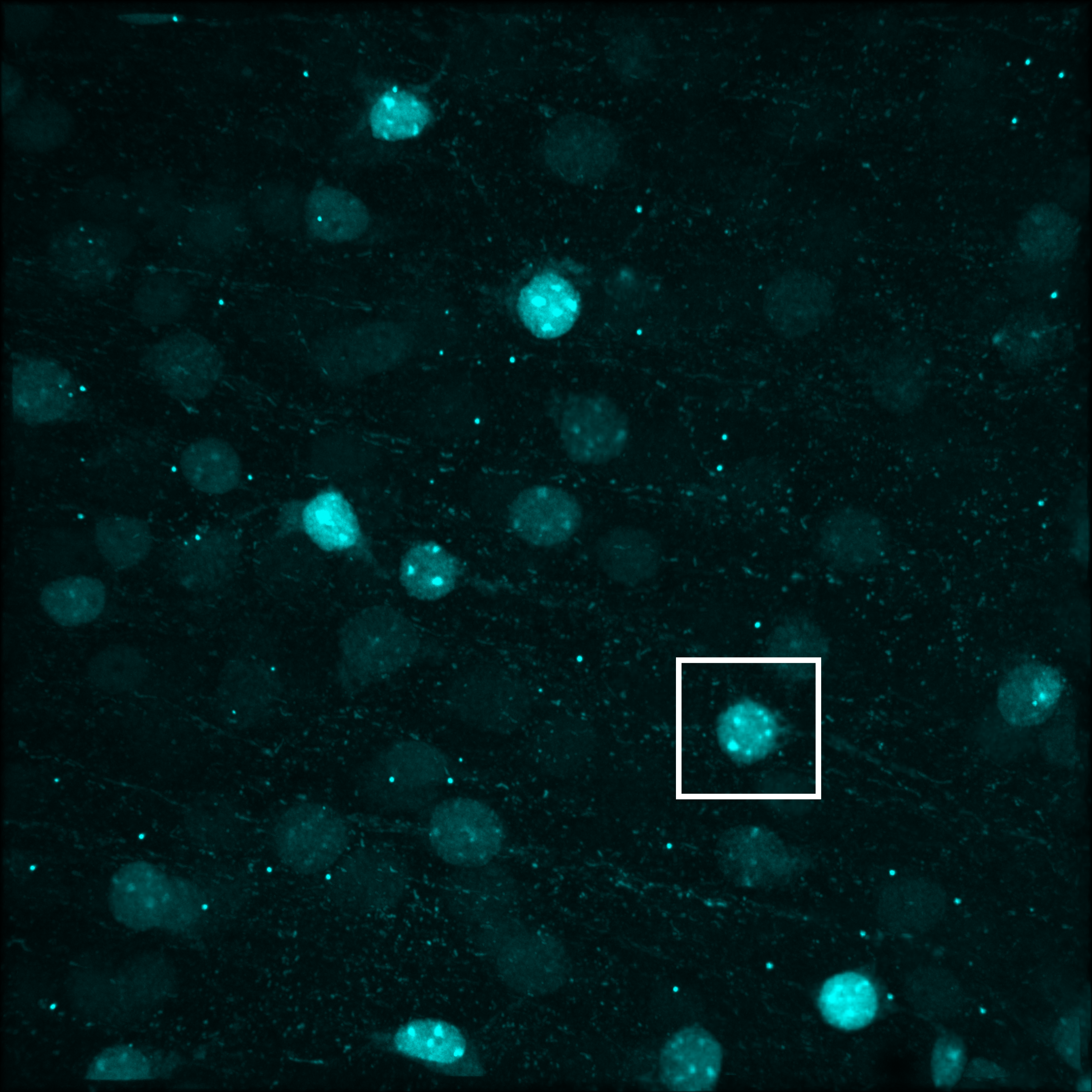

Supplement: Supplementary file 9 — Source data Fig. 1H [file 44318_2025_624_MOESM9_ESM.zip › 1H/n3/CFSE.tif]

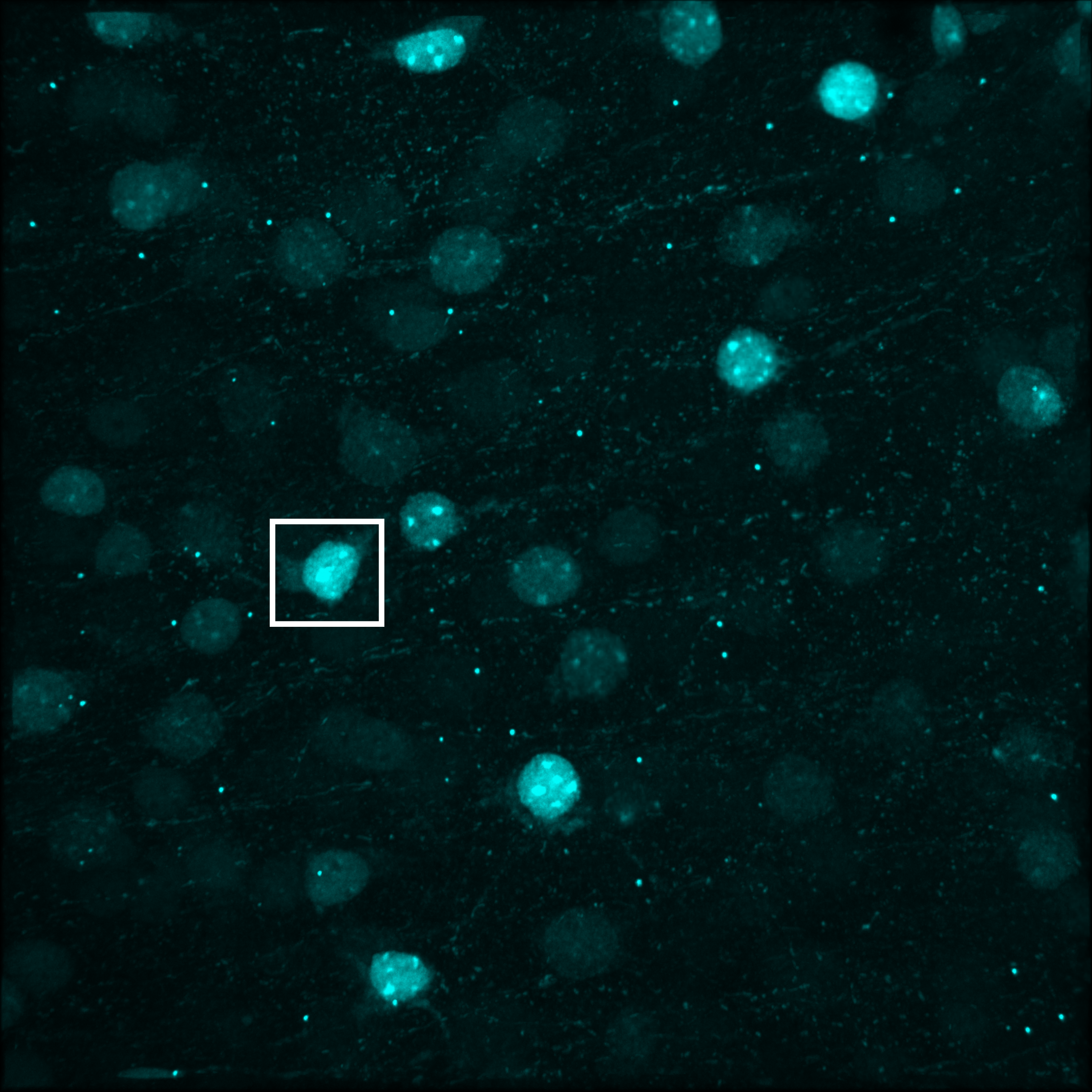

Supplement: Supplementary file 9 — Source data Fig. 1H [file 44318_2025_624_MOESM9_ESM.zip › 1H/n2/CFSE.tif]

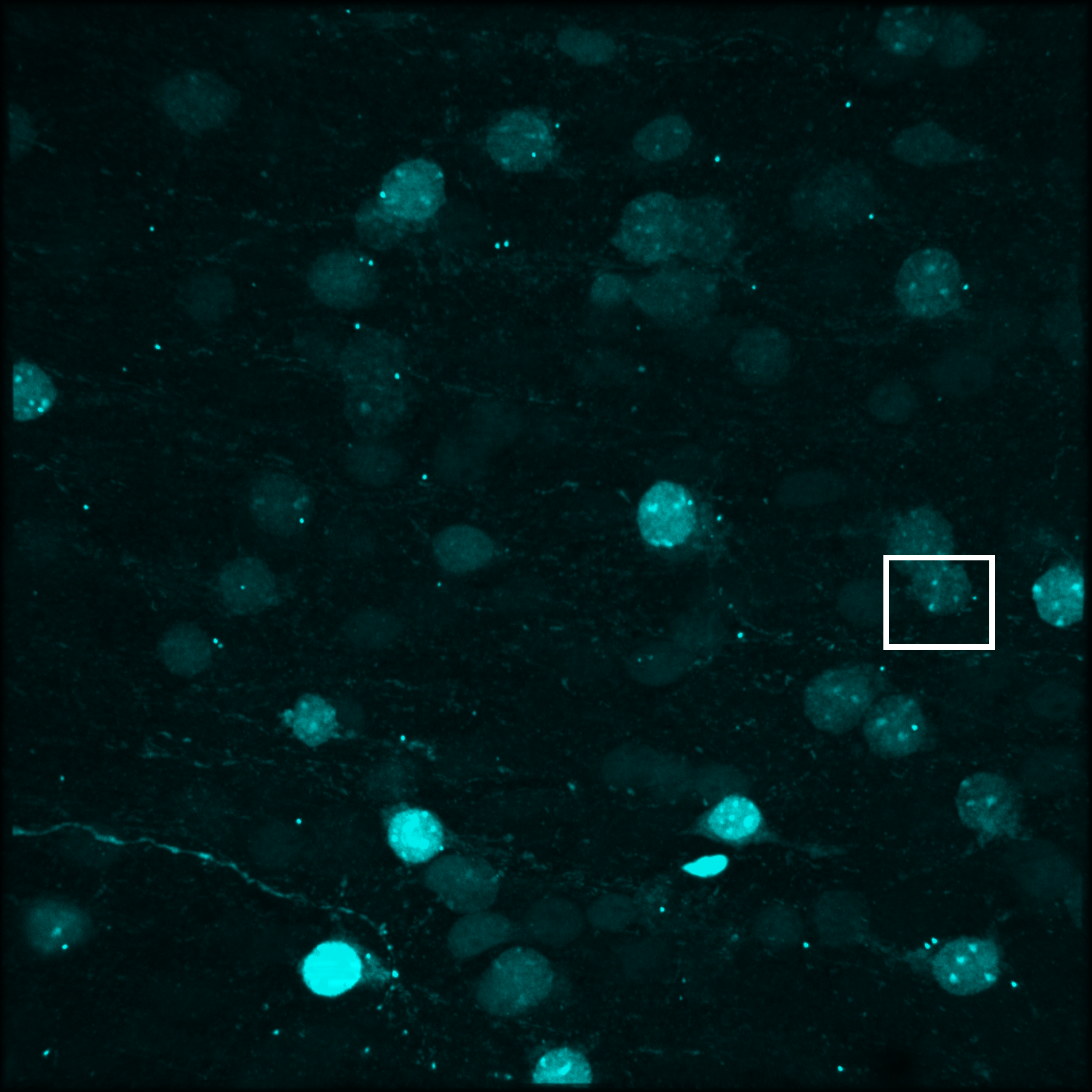

Supplement: Supplementary file 9 — Source data Fig. 1H [file 44318_2025_624_MOESM9_ESM.zip › 1H/n5/CFSE.tif]

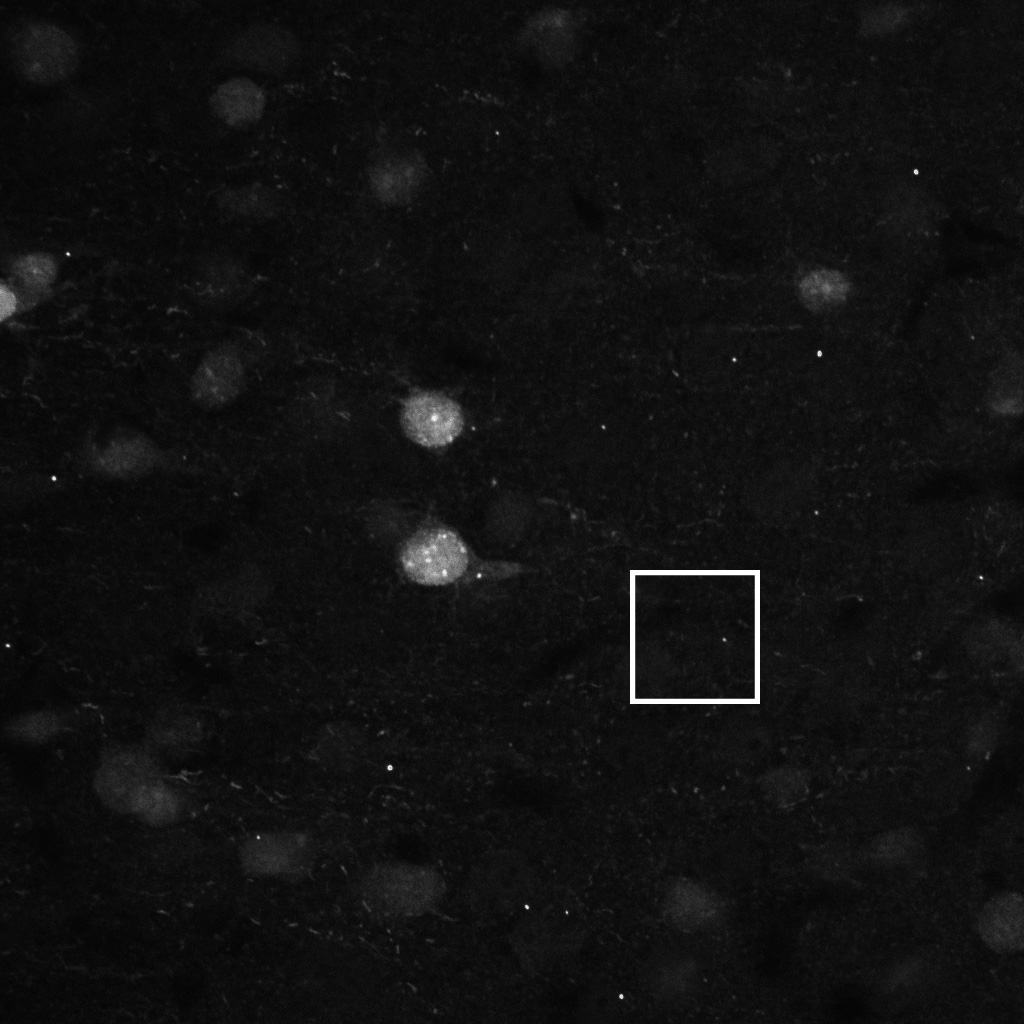

Supplement: Supplementary file 9 — Source data Fig. 1H [file 44318_2025_624_MOESM9_ESM.zip › 1H/n7/CFSE_Z stacks/8_2_C0_Z000 (3).jpg]

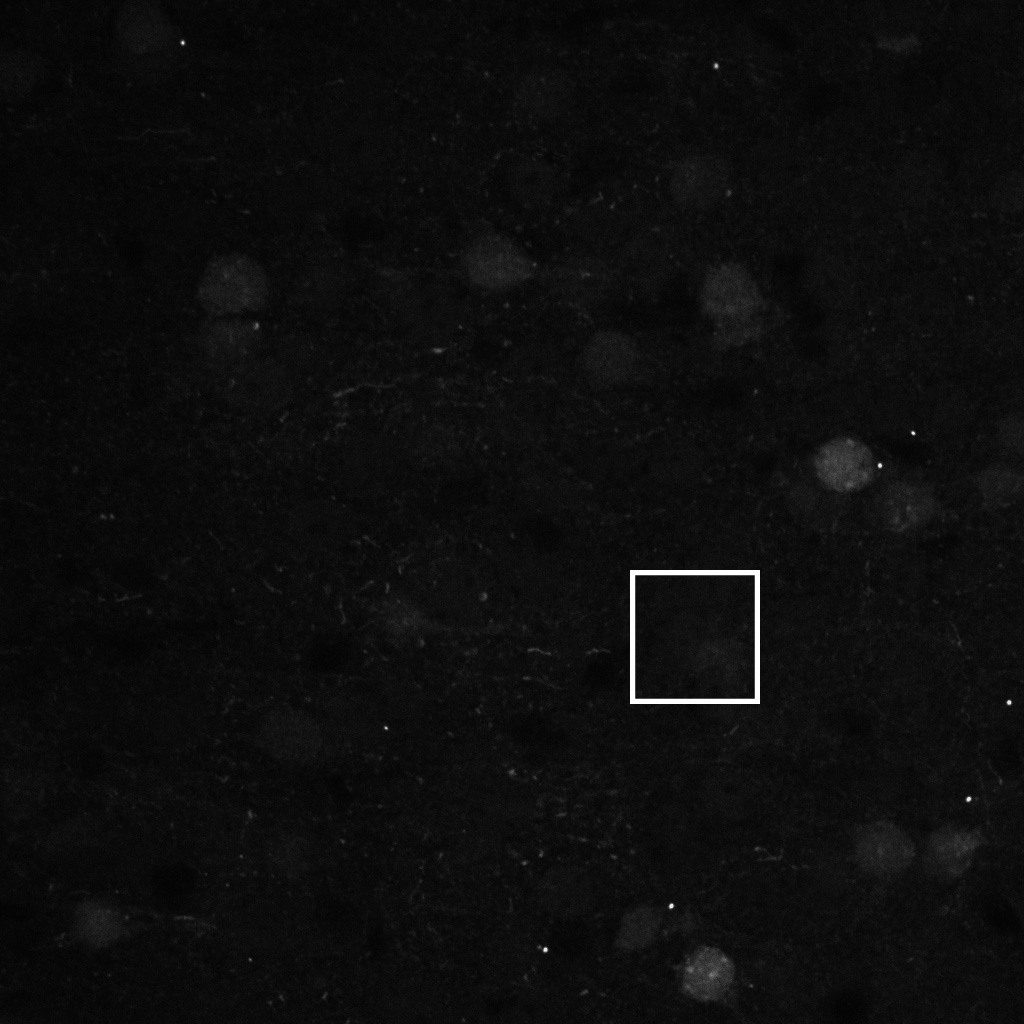

Supplement: Supplementary file 9 — Source data Fig. 1H [file 44318_2025_624_MOESM9_ESM.zip › 1H/n7/CFSE_Z stacks/8_2_C0_Z000 (35).jpg]

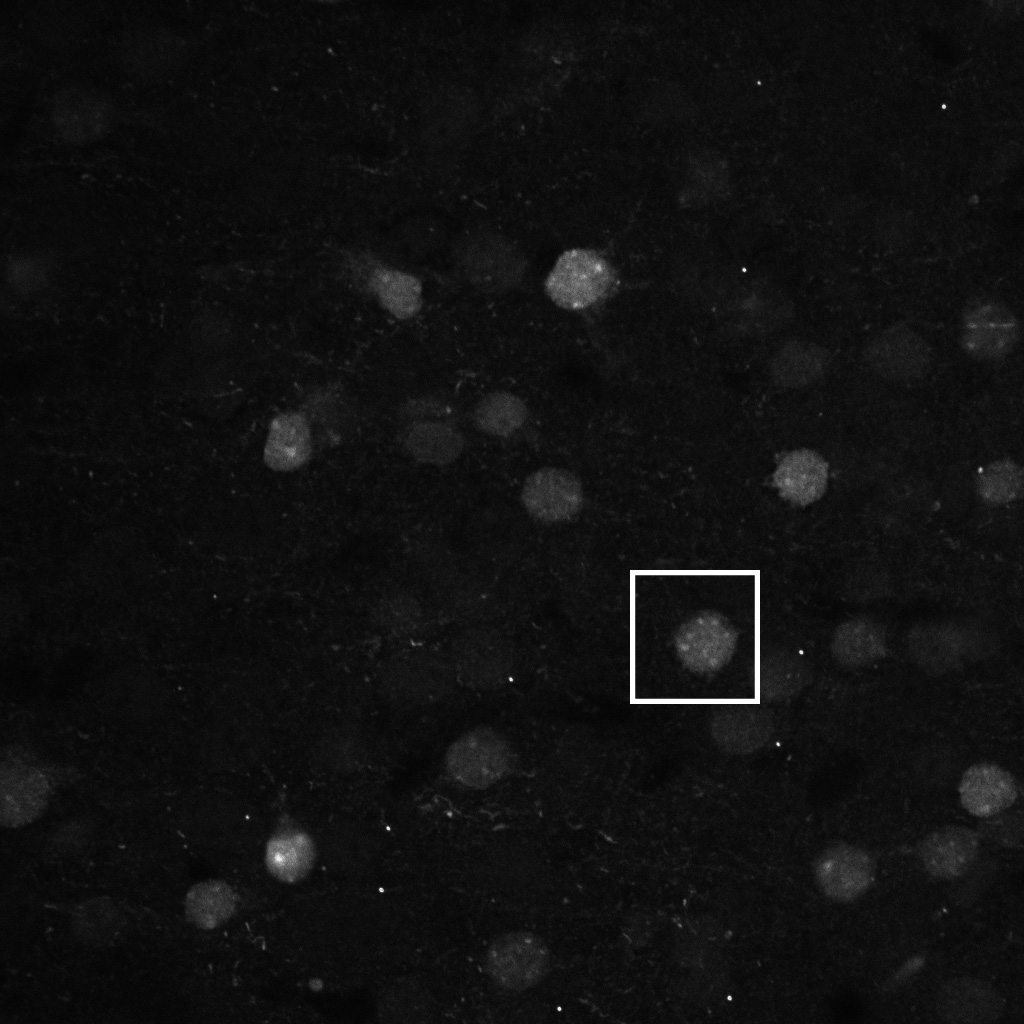

Supplement: Supplementary file 9 — Source data Fig. 1H [file 44318_2025_624_MOESM9_ESM.zip › 1H/n7/CFSE_Z stacks/8_2_C0_Z000 (23).jpg]

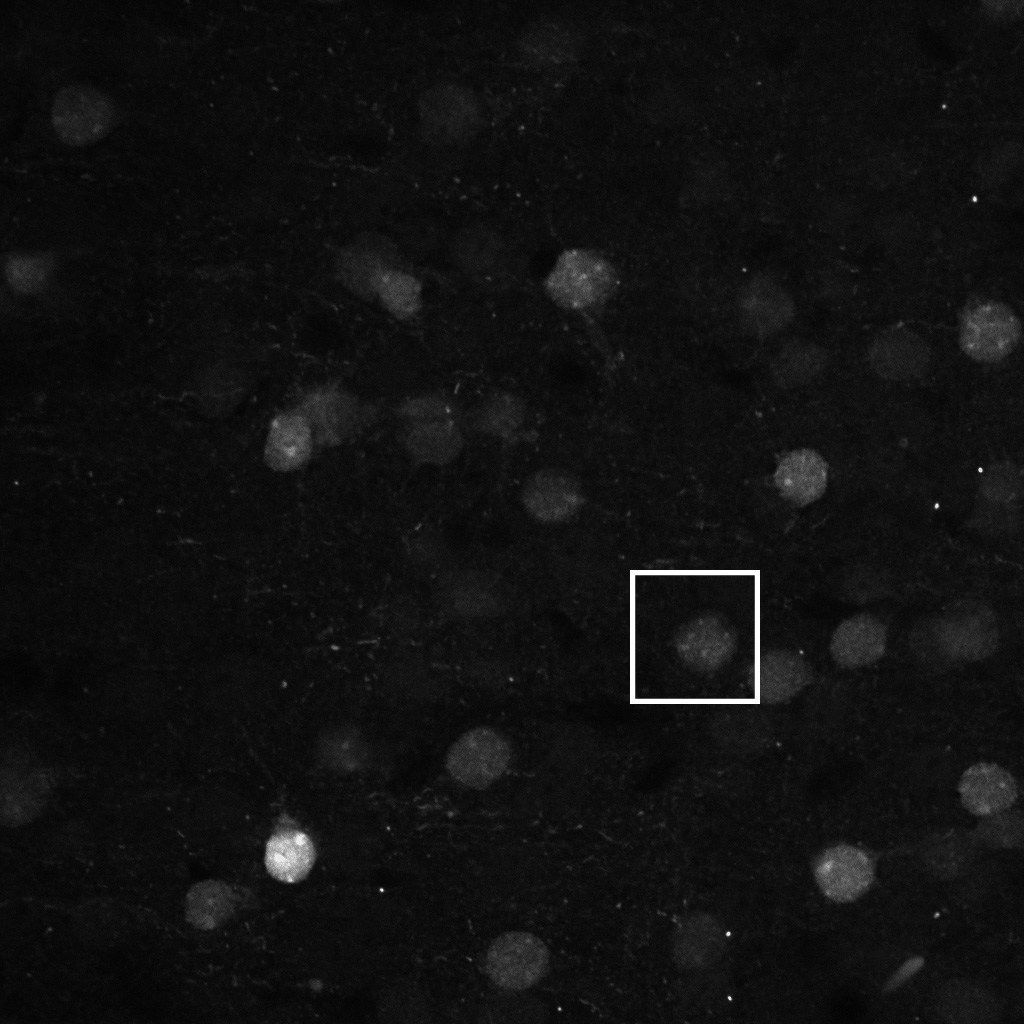

Supplement: Supplementary file 9 — Source data Fig. 1H [file 44318_2025_624_MOESM9_ESM.zip › 1H/n7/CFSE_Z stacks/8_2_C0_Z000 (19).jpg]

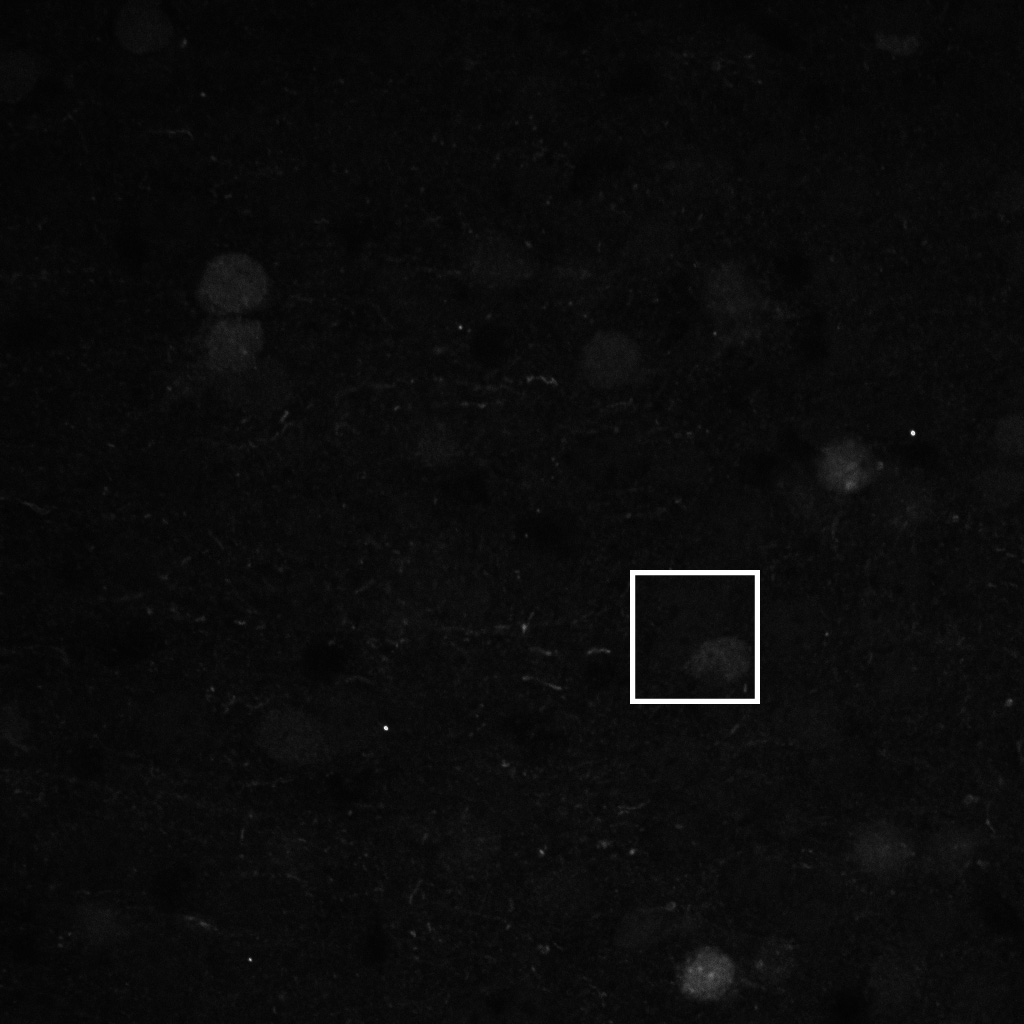

Supplement: Supplementary file 9 — Source data Fig. 1H [file 44318_2025_624_MOESM9_ESM.zip › 1H/n7/CFSE_Z stacks/8_2_C0_Z000 (39).jpg]

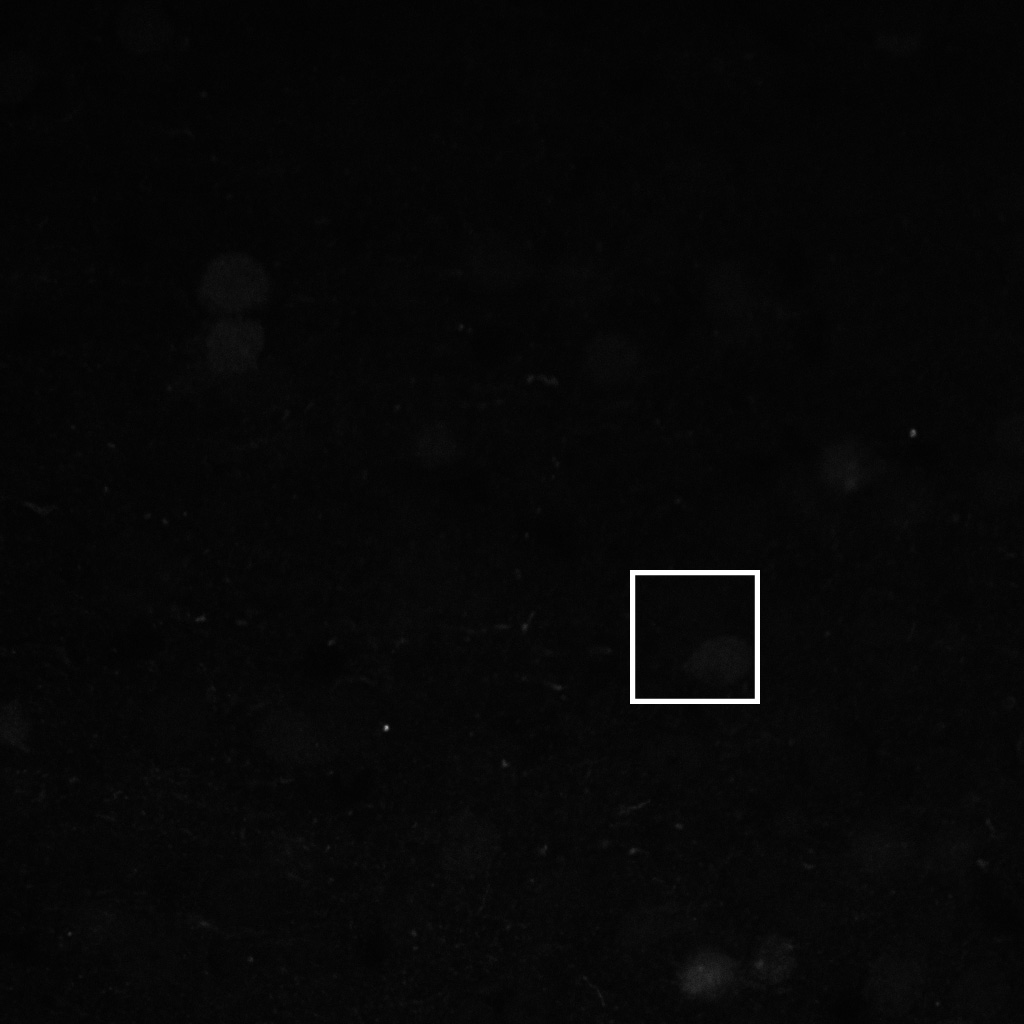

Supplement: Supplementary file 9 — Source data Fig. 1H [file 44318_2025_624_MOESM9_ESM.zip › 1H/n7/CFSE_Z stacks/8_2_C0_Z000 (42).jpg]

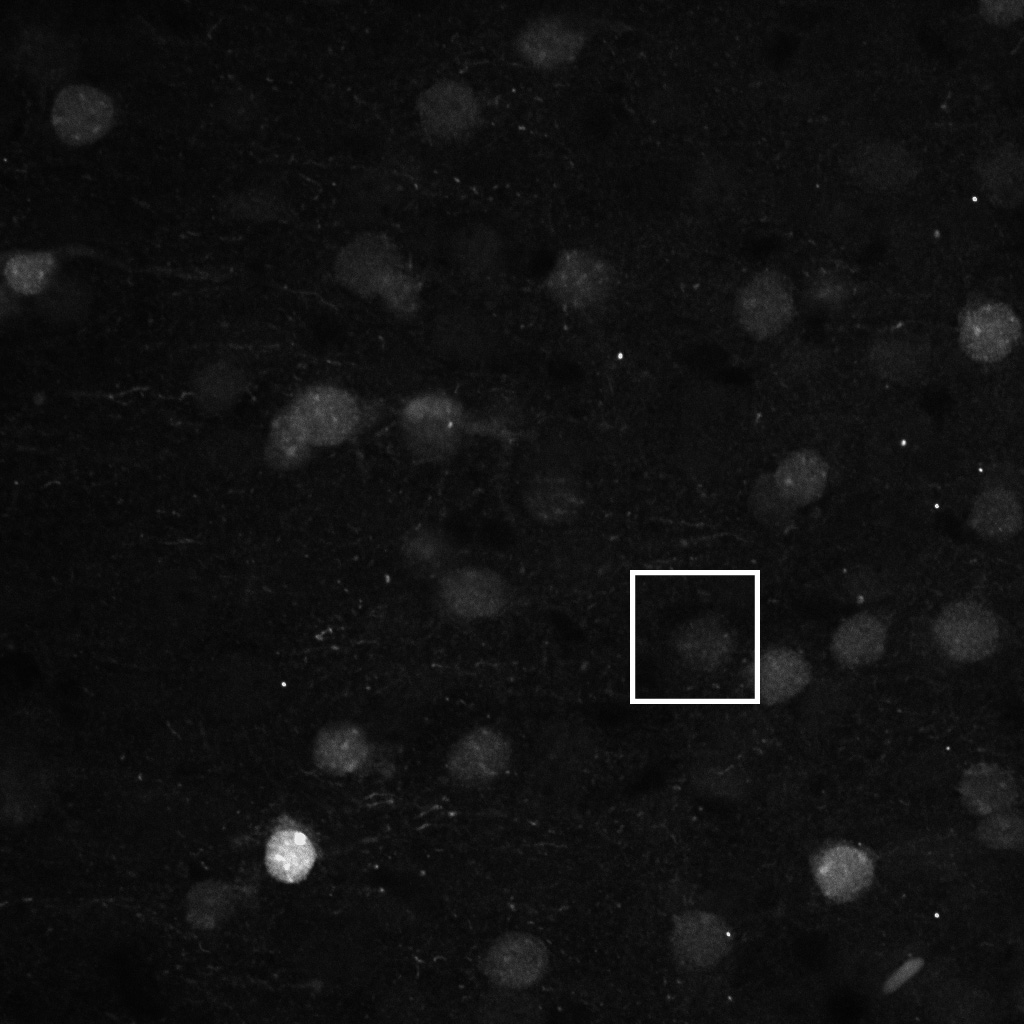

Supplement: Supplementary file 9 — Source data Fig. 1H [file 44318_2025_624_MOESM9_ESM.zip › 1H/n7/CFSE_Z stacks/8_2_C0_Z000 (15).jpg]

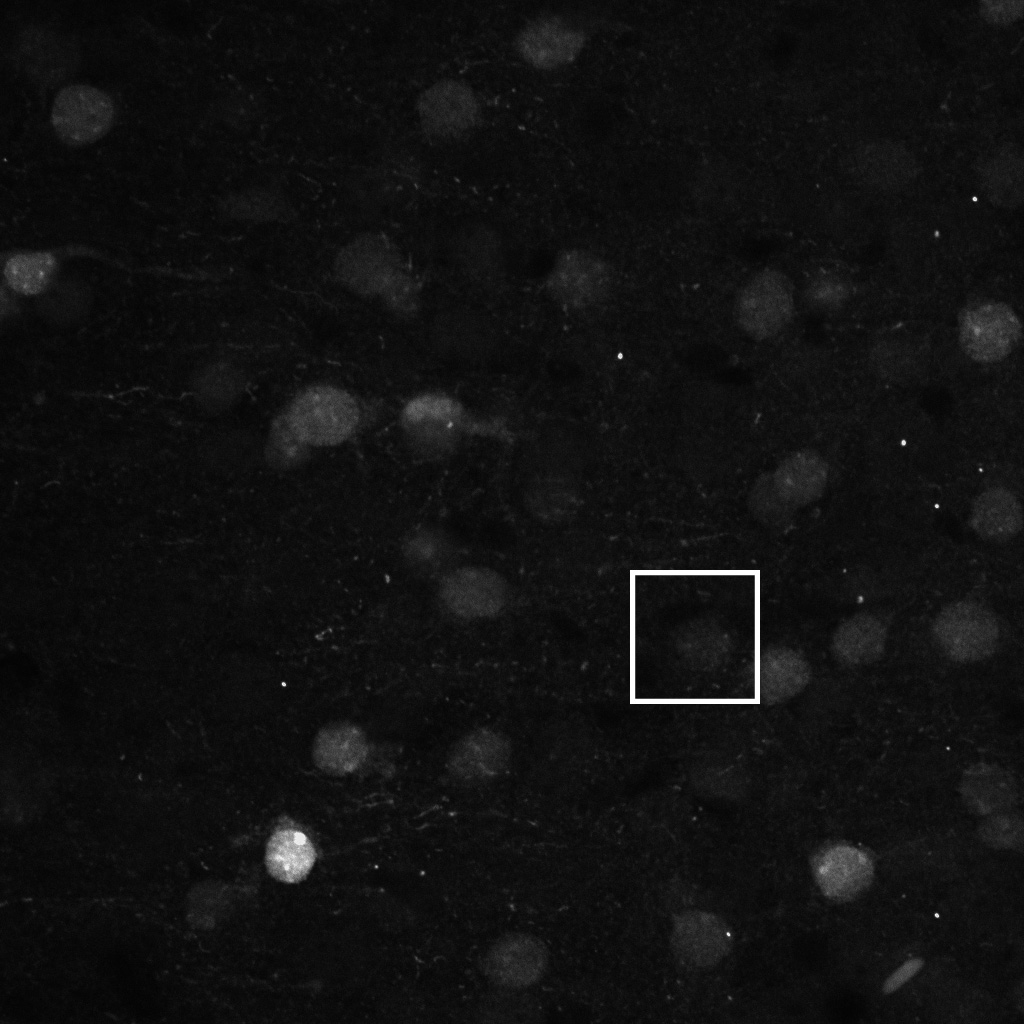

Supplement: Supplementary file 9 — Source data Fig. 1H [file 44318_2025_624_MOESM9_ESM.zip › 1H/n7/CFSE_Z stacks/8_2_C0_Z000 (14).jpg]

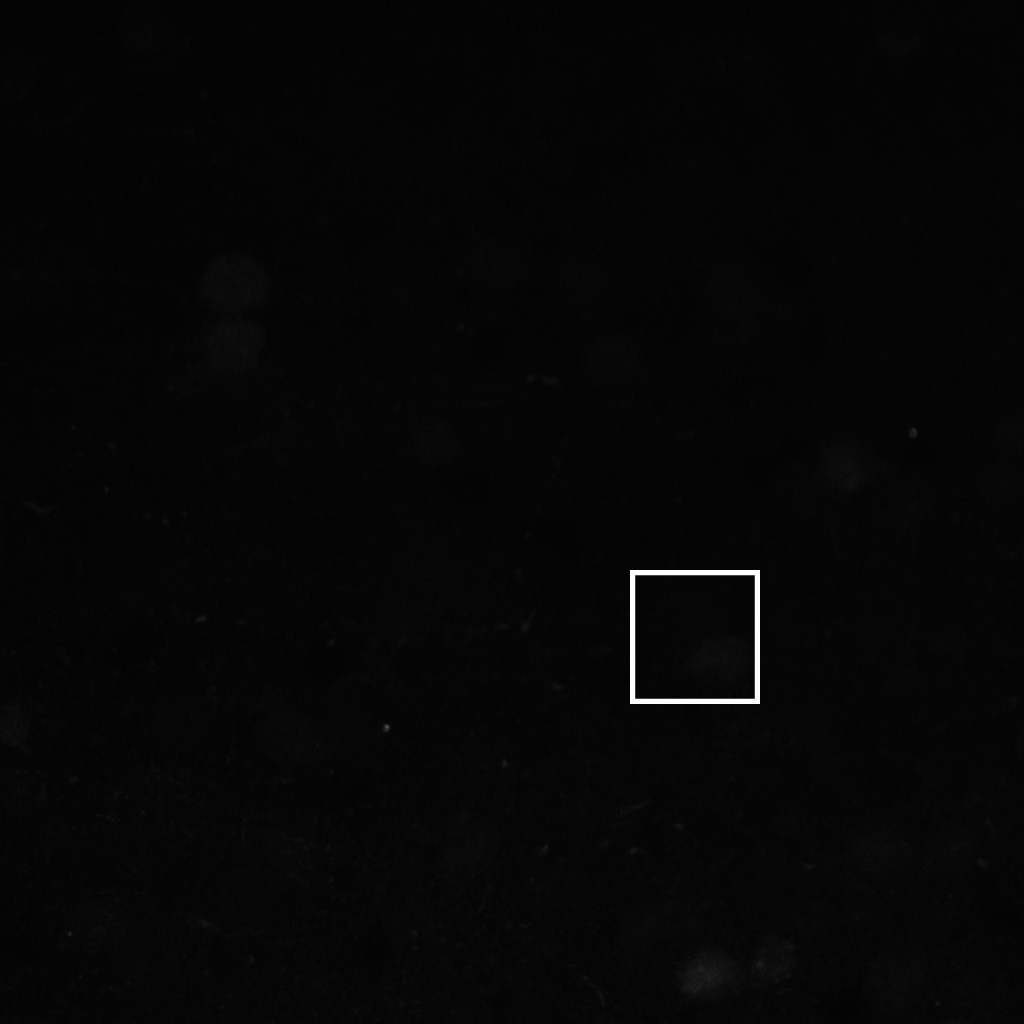

Supplement: Supplementary file 9 — Source data Fig. 1H [file 44318_2025_624_MOESM9_ESM.zip › 1H/n7/CFSE_Z stacks/8_2_C0_Z000 (43).jpg]

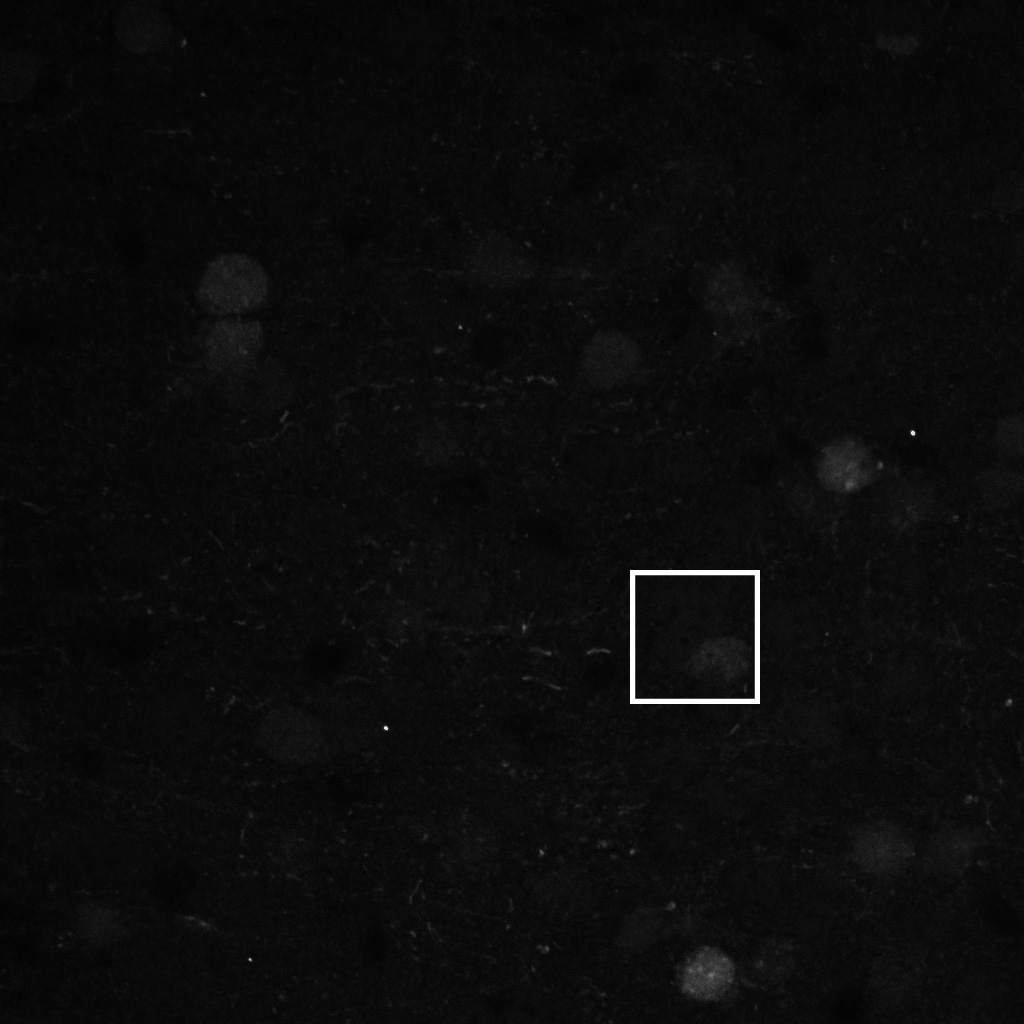

Supplement: Supplementary file 9 — Source data Fig. 1H [file 44318_2025_624_MOESM9_ESM.zip › 1H/n7/CFSE_Z stacks/8_2_C0_Z000 (38).jpg]

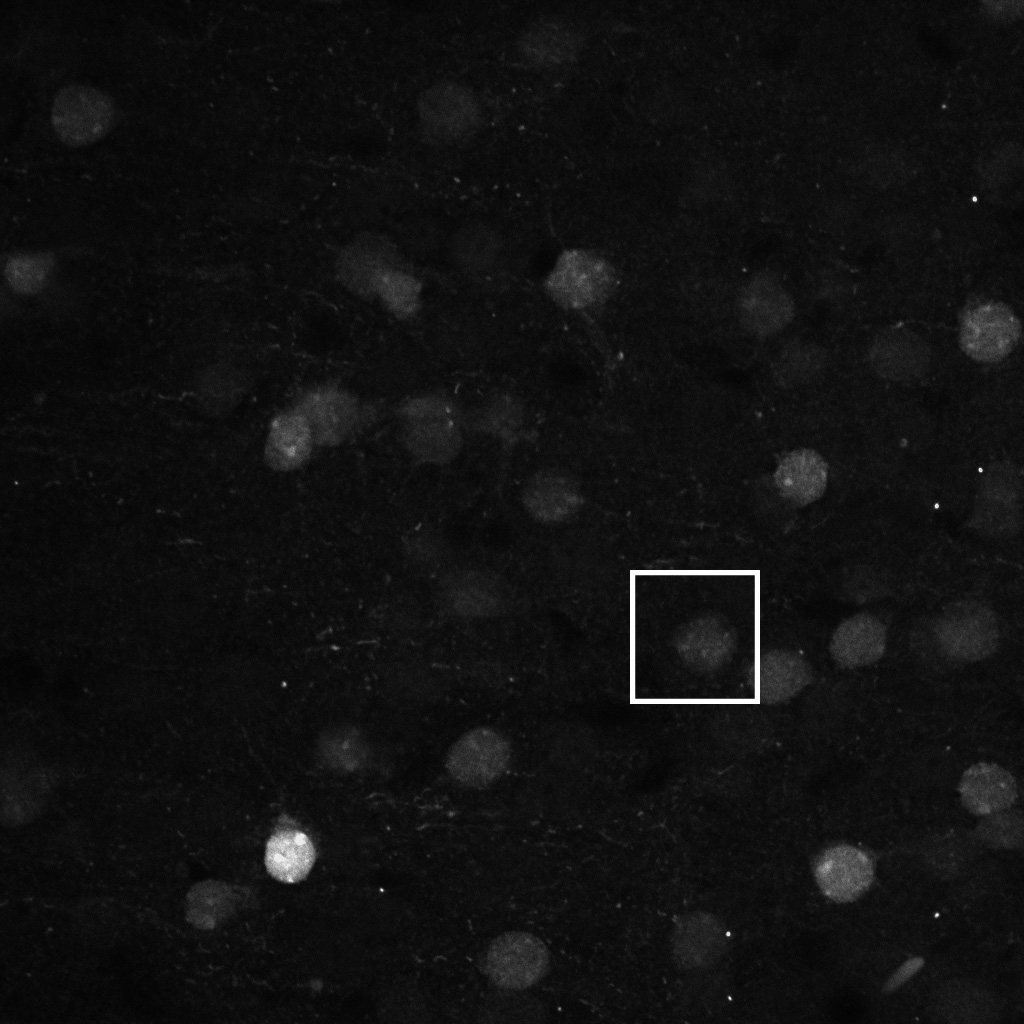

Supplement: Supplementary file 9 — Source data Fig. 1H [file 44318_2025_624_MOESM9_ESM.zip › 1H/n7/CFSE_Z stacks/8_2_C0_Z000 (18).jpg]

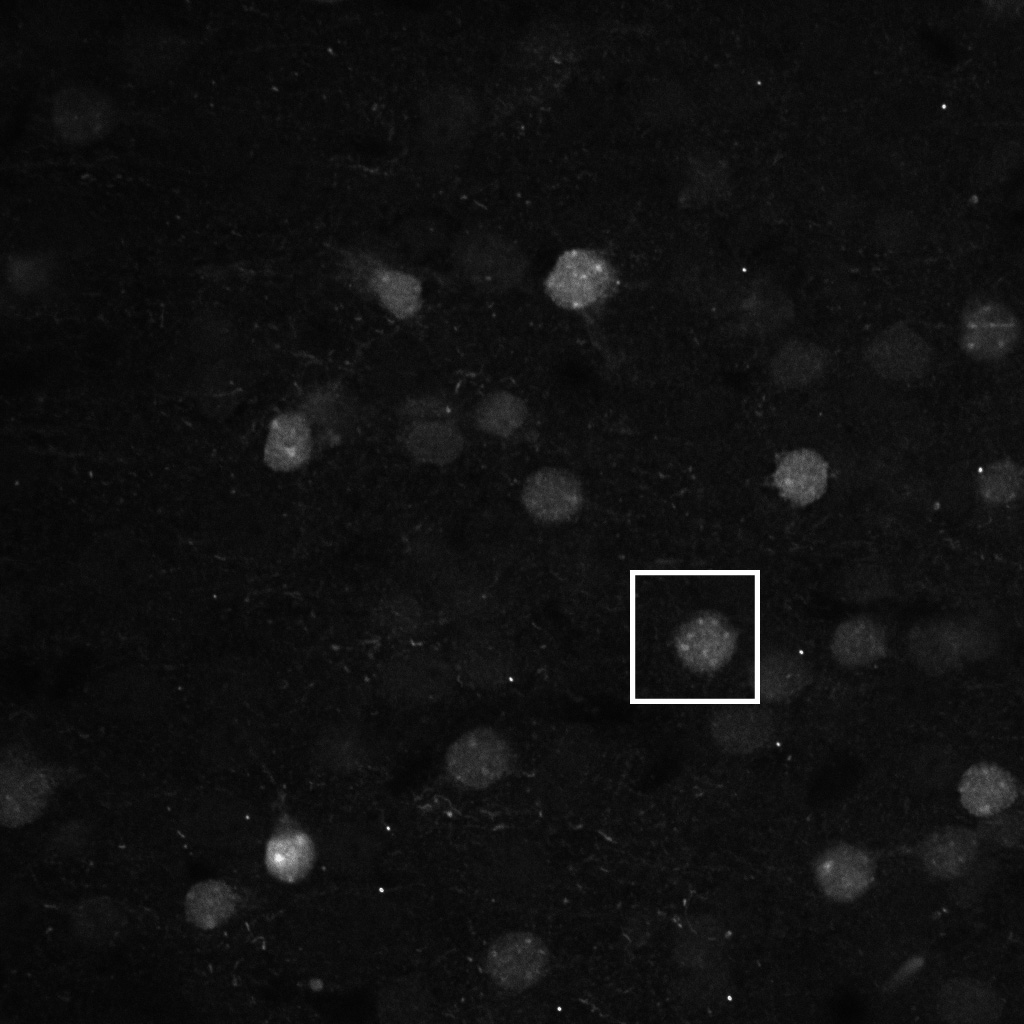

Supplement: Supplementary file 9 — Source data Fig. 1H [file 44318_2025_624_MOESM9_ESM.zip › 1H/n7/CFSE_Z stacks/8_2_C0_Z000 (22).jpg]

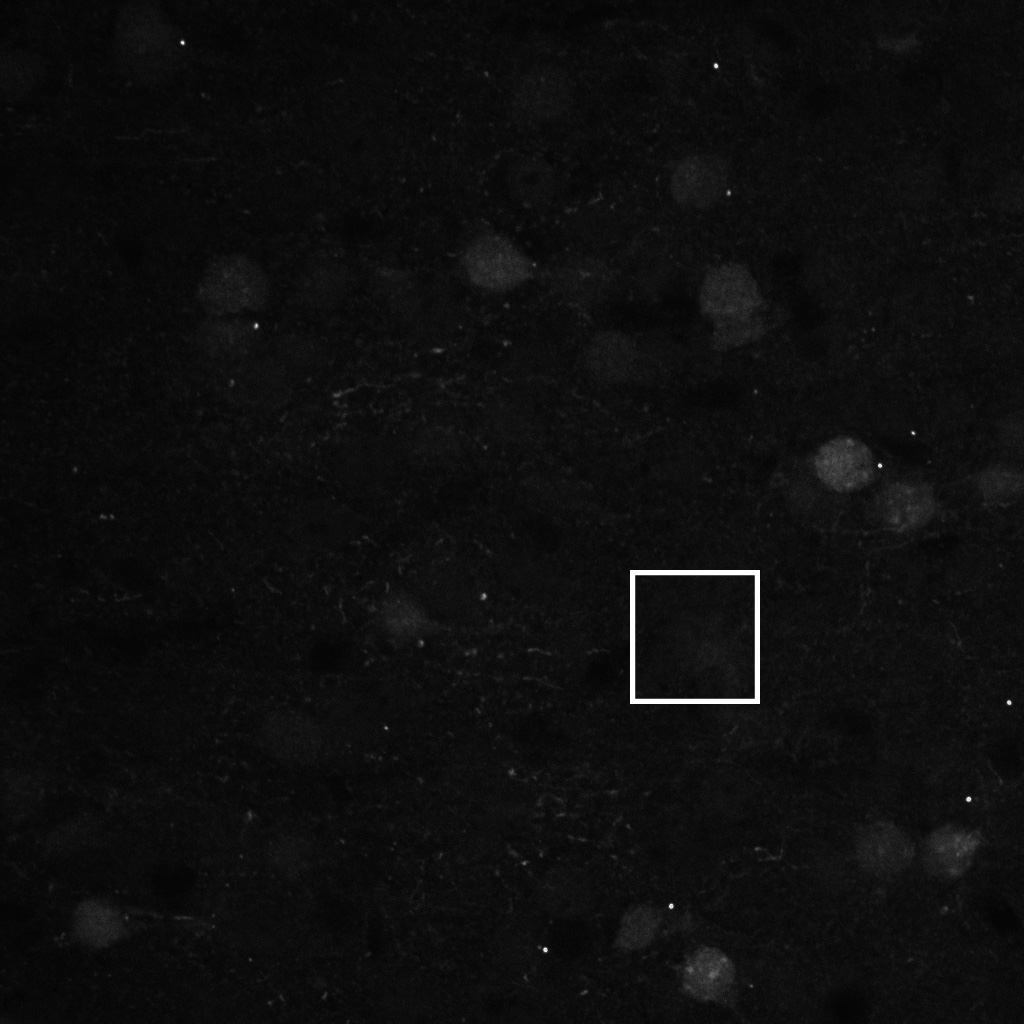

Supplement: Supplementary file 9 — Source data Fig. 1H [file 44318_2025_624_MOESM9_ESM.zip › 1H/n7/CFSE_Z stacks/8_2_C0_Z000 (34).jpg]

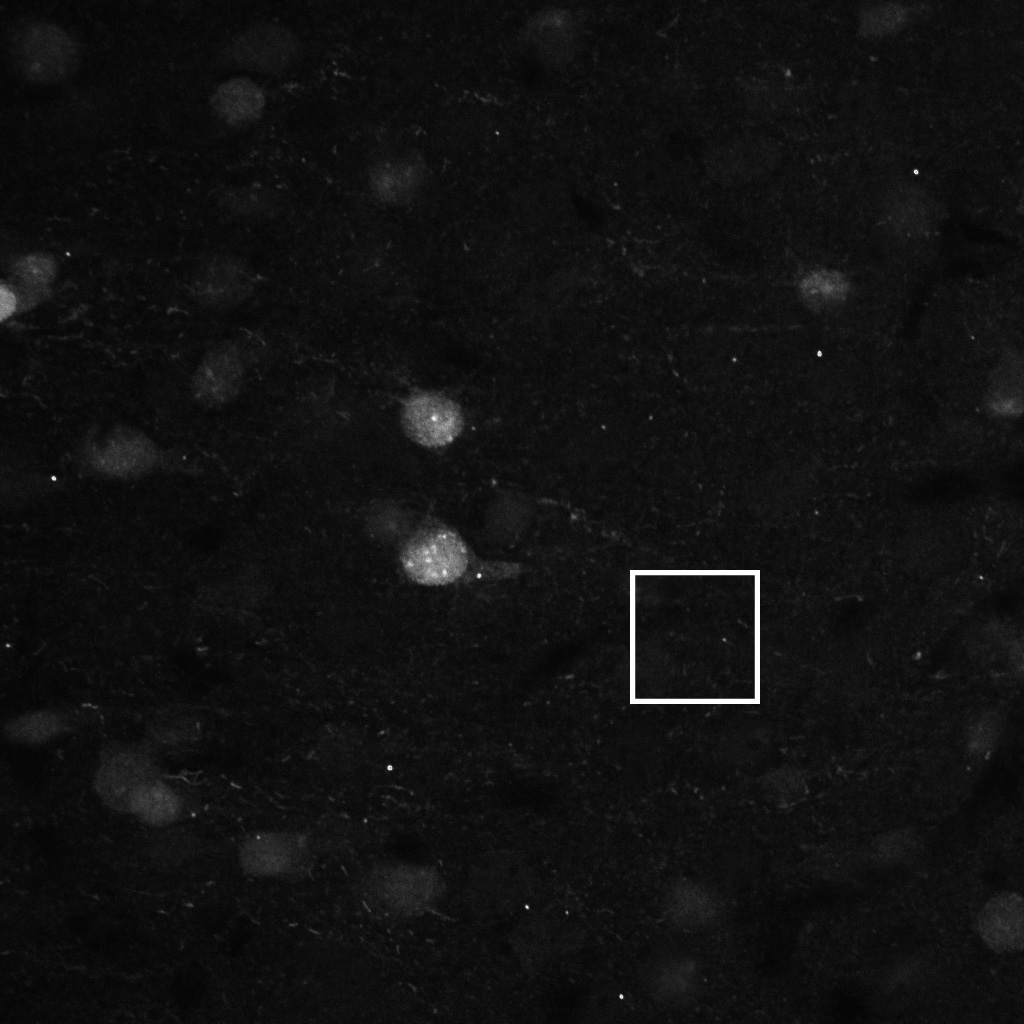

Supplement: Supplementary file 9 — Source data Fig. 1H [file 44318_2025_624_MOESM9_ESM.zip › 1H/n7/CFSE_Z stacks/8_2_C0_Z000 (2).jpg]

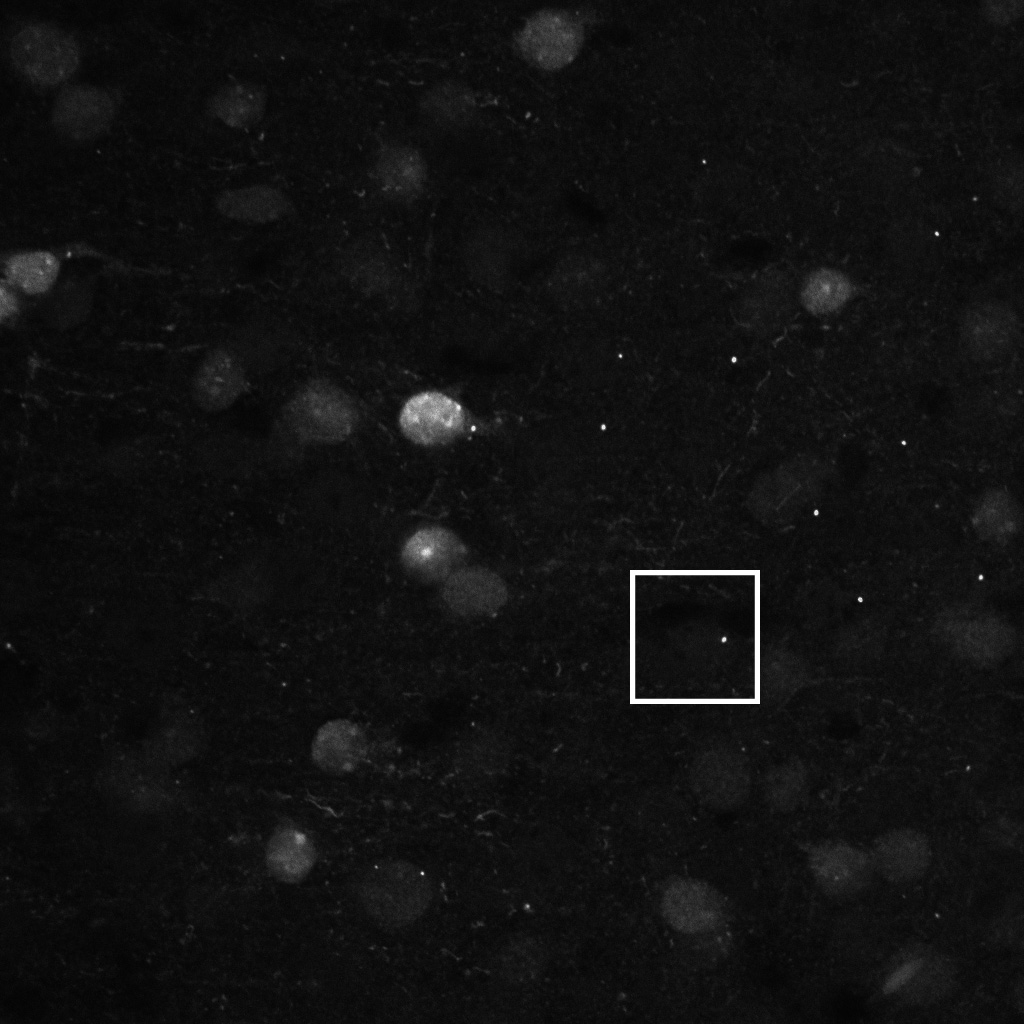

Supplement: Supplementary file 9 — Source data Fig. 1H [file 44318_2025_624_MOESM9_ESM.zip › 1H/n7/CFSE_Z stacks/8_2_C0_Z000 (9).jpg]

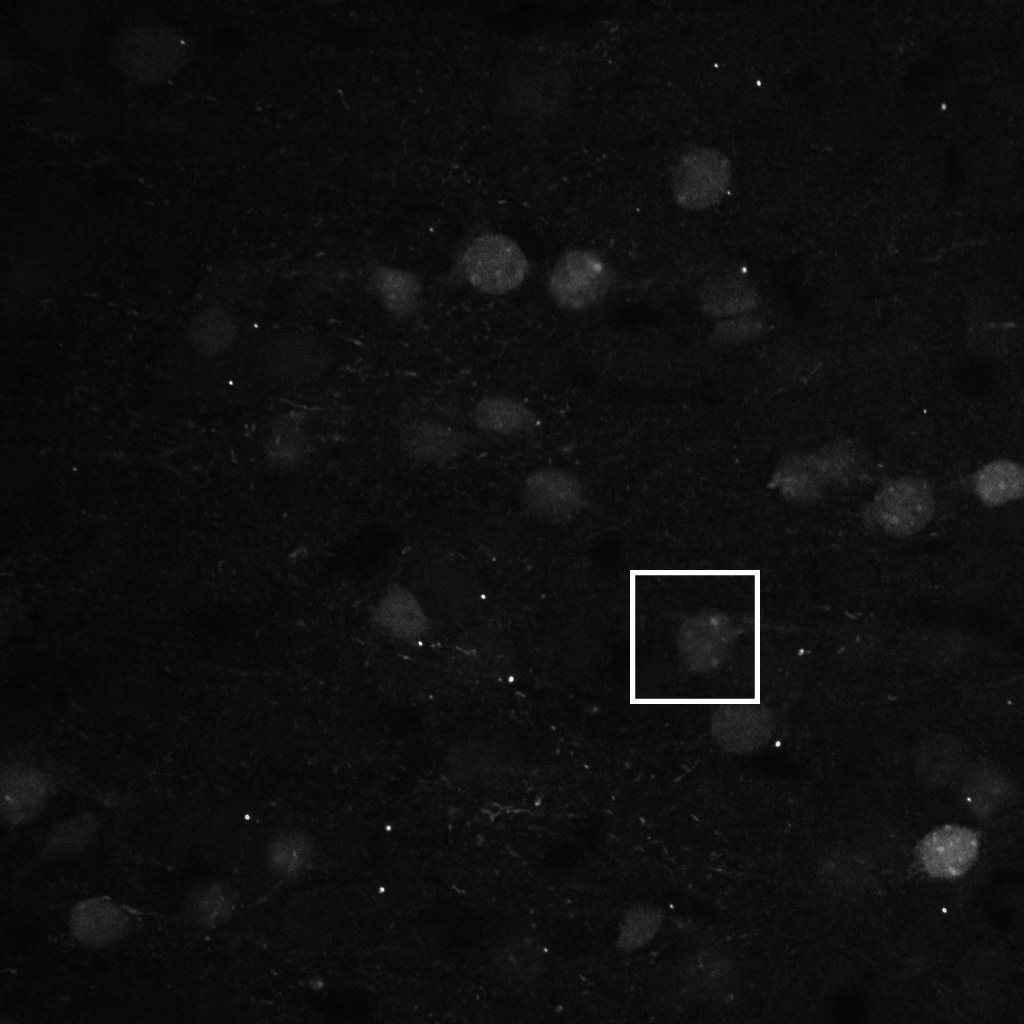

Supplement: Supplementary file 9 — Source data Fig. 1H [file 44318_2025_624_MOESM9_ESM.zip › 1H/n7/CFSE_Z stacks/8_2_C0_Z000 (29).jpg]

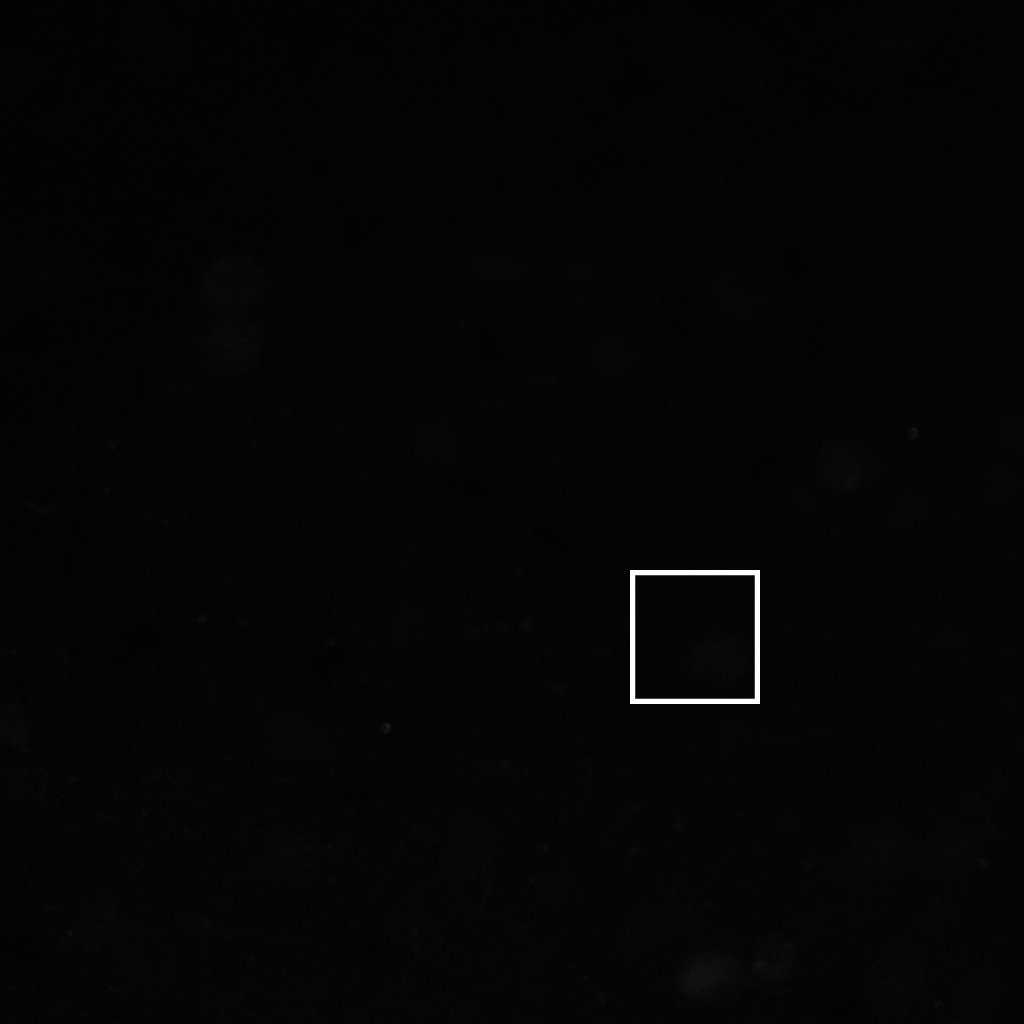

Supplement: Supplementary file 9 — Source data Fig. 1H [file 44318_2025_624_MOESM9_ESM.zip › 1H/n7/CFSE_Z stacks/8_2_C0_Z000 (44).jpg]

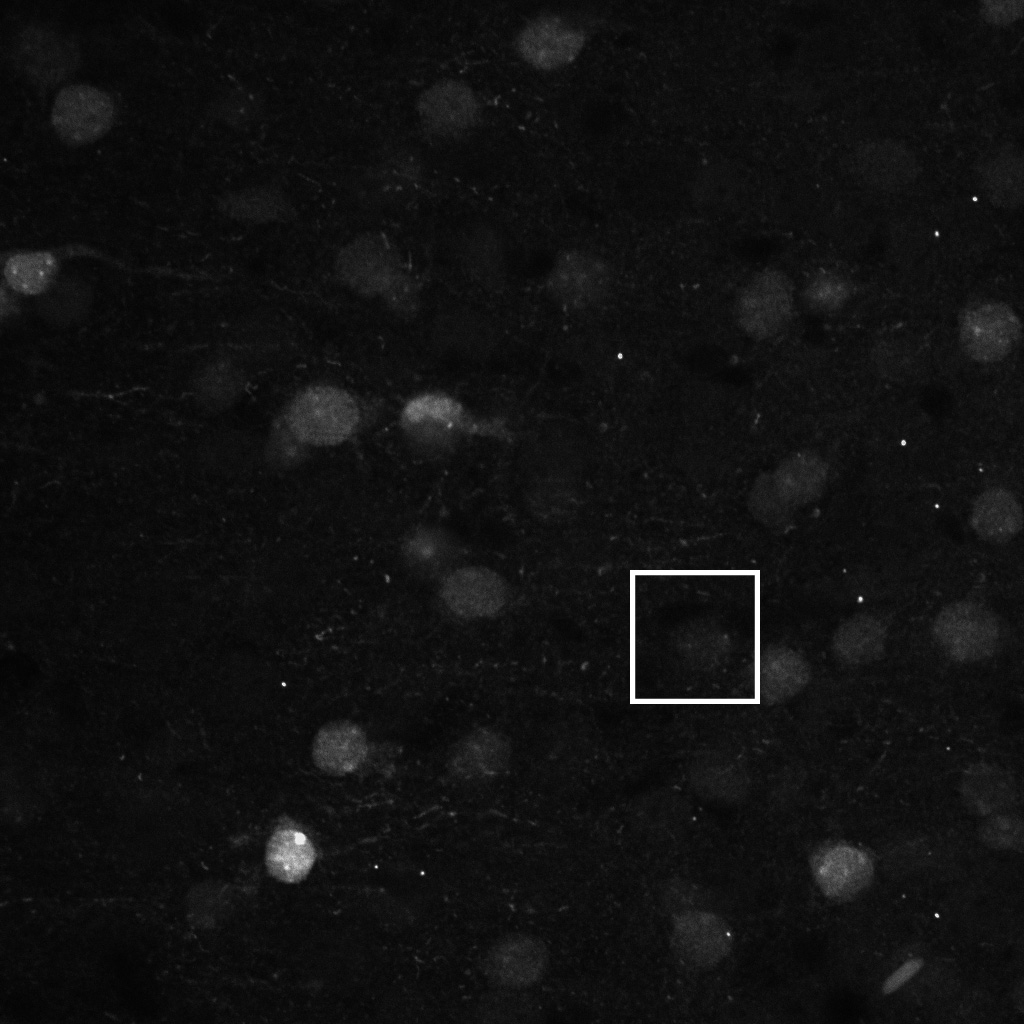

Supplement: Supplementary file 9 — Source data Fig. 1H [file 44318_2025_624_MOESM9_ESM.zip › 1H/n7/CFSE_Z stacks/8_2_C0_Z000 (13).jpg]

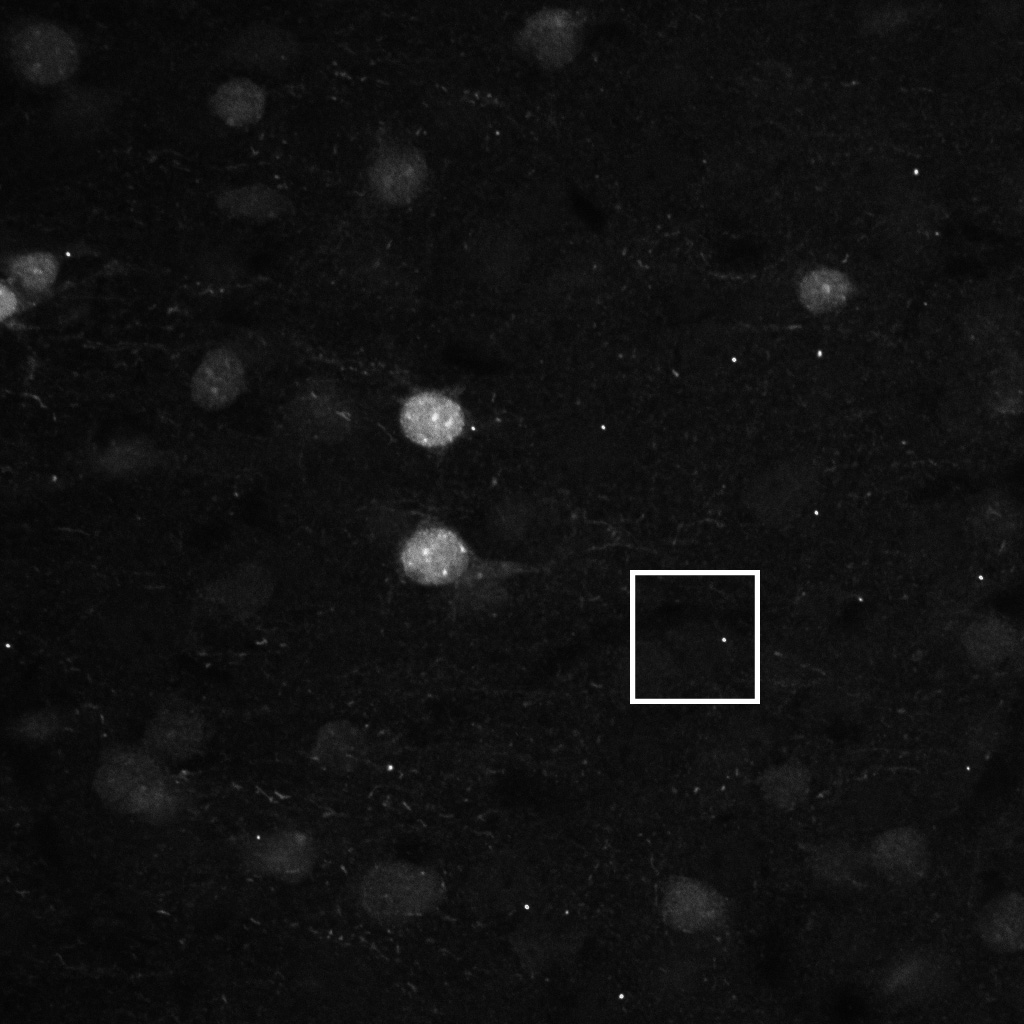

Supplement: Supplementary file 9 — Source data Fig. 1H [file 44318_2025_624_MOESM9_ESM.zip › 1H/n7/CFSE_Z stacks/8_2_C0_Z000 (5).jpg]

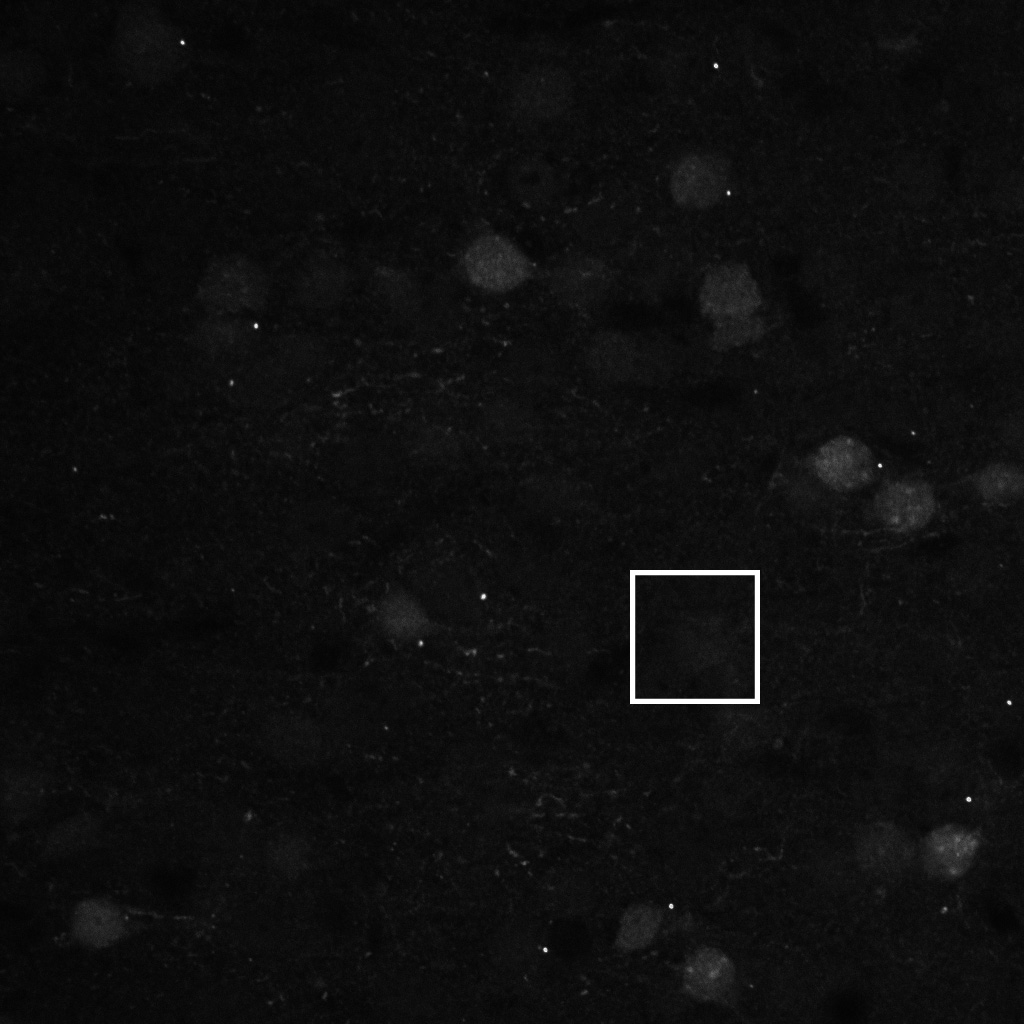

Supplement: Supplementary file 9 — Source data Fig. 1H [file 44318_2025_624_MOESM9_ESM.zip › 1H/n7/CFSE_Z stacks/8_2_C0_Z000 (33).jpg]

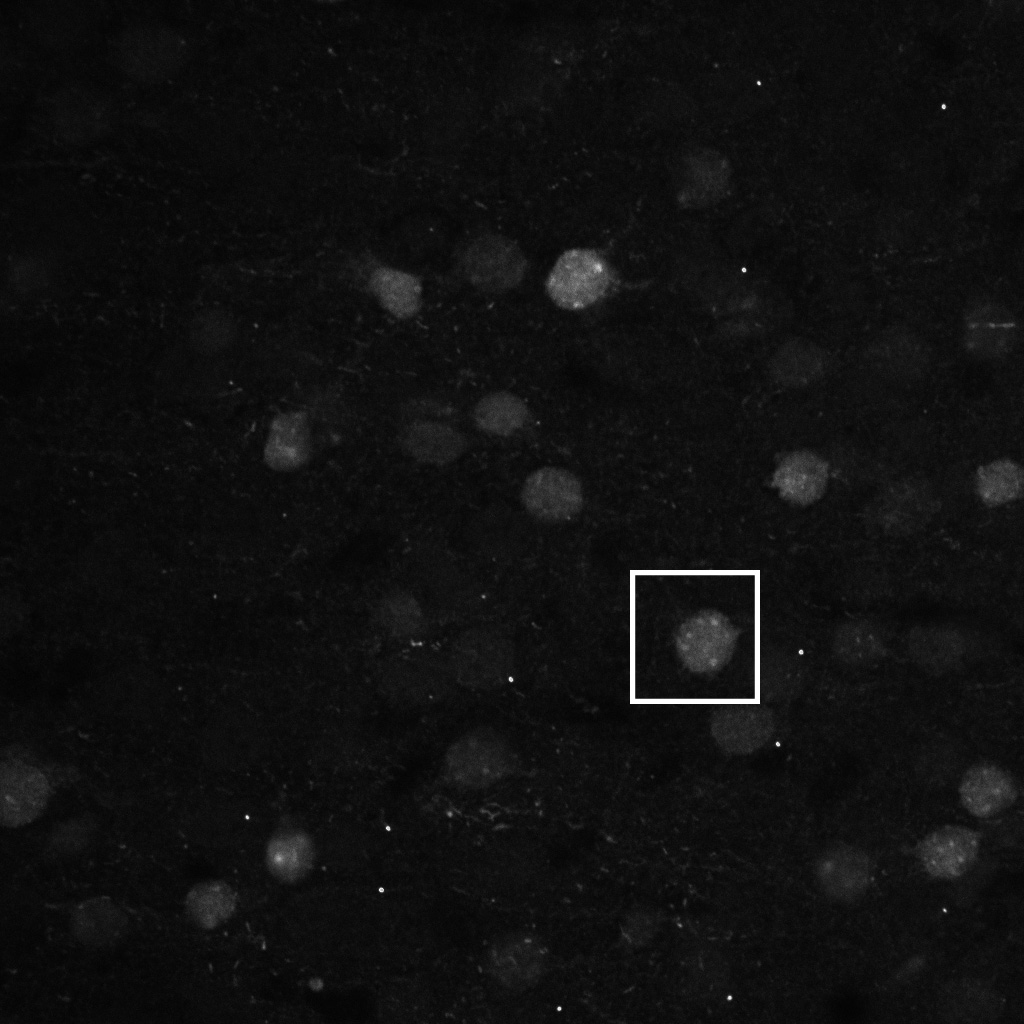

Supplement: Supplementary file 9 — Source data Fig. 1H [file 44318_2025_624_MOESM9_ESM.zip › 1H/n7/CFSE_Z stacks/8_2_C0_Z000 (25).jpg]

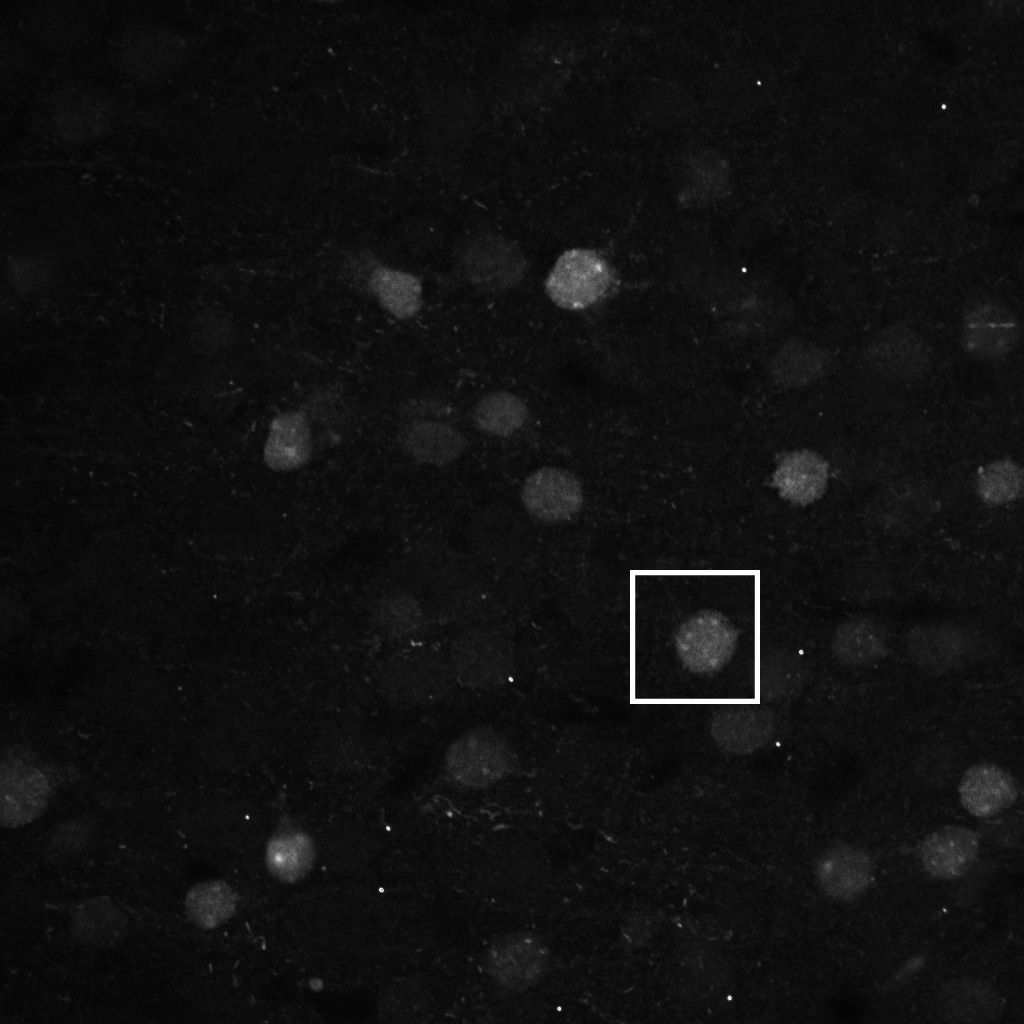

Supplement: Supplementary file 9 — Source data Fig. 1H [file 44318_2025_624_MOESM9_ESM.zip › 1H/n7/CFSE_Z stacks/8_2_C0_Z000 (24).jpg]

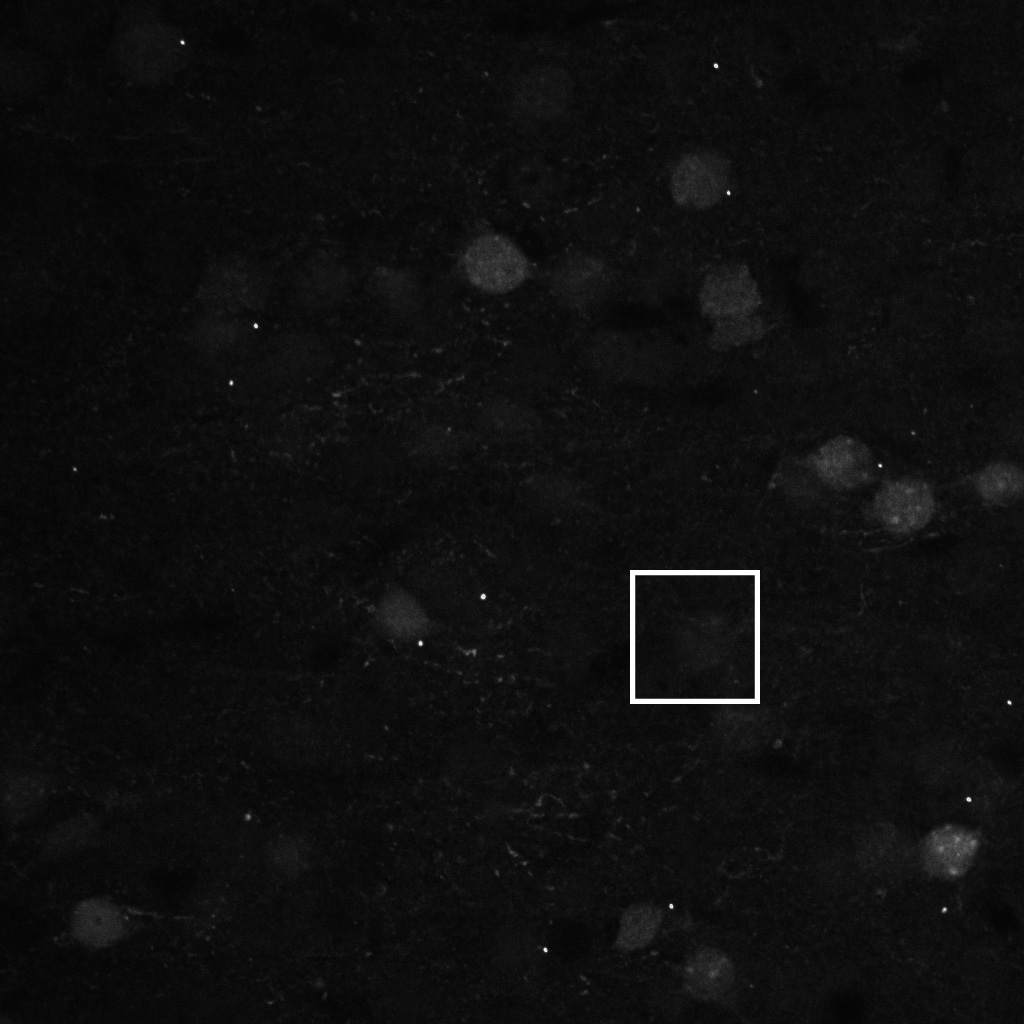

Supplement: Supplementary file 9 — Source data Fig. 1H [file 44318_2025_624_MOESM9_ESM.zip › 1H/n7/CFSE_Z stacks/8_2_C0_Z000 (32).jpg]

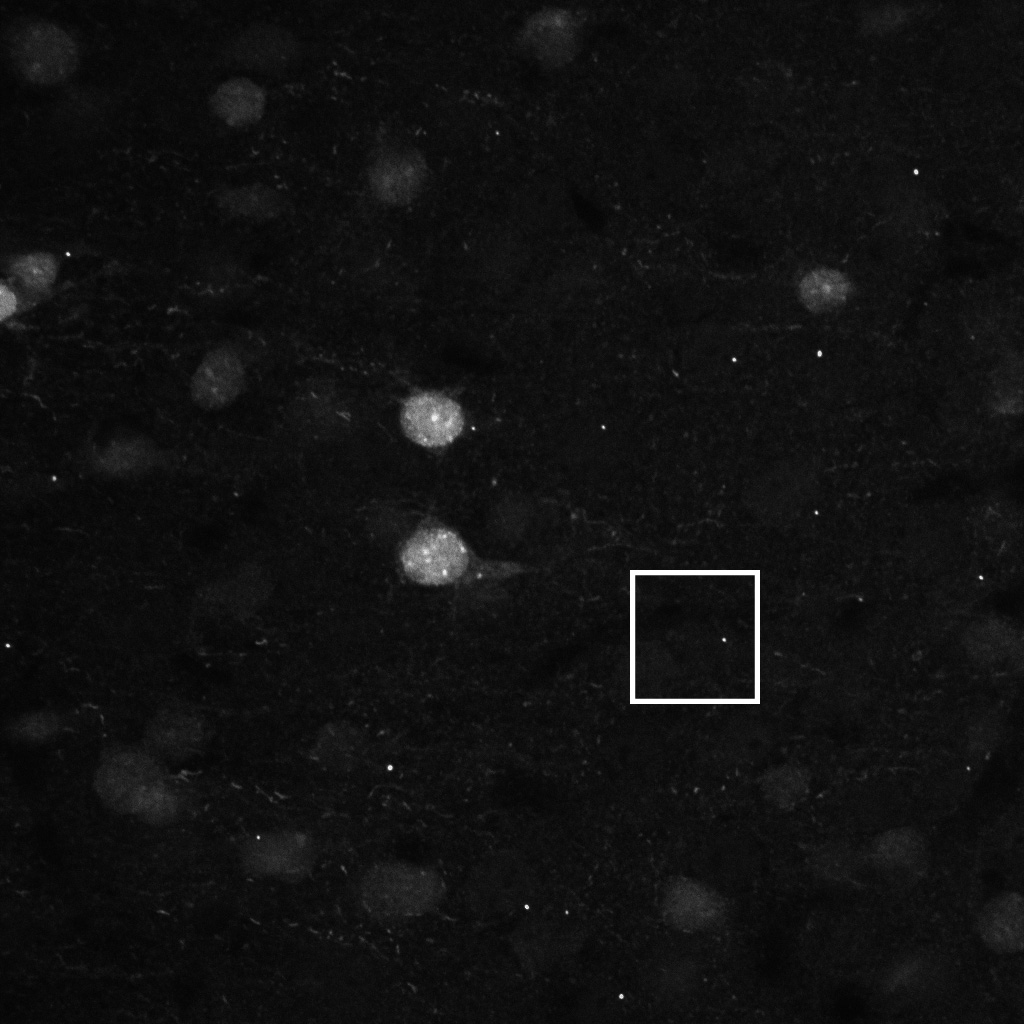

Supplement: Supplementary file 9 — Source data Fig. 1H [file 44318_2025_624_MOESM9_ESM.zip › 1H/n7/CFSE_Z stacks/8_2_C0_Z000 (4).jpg]

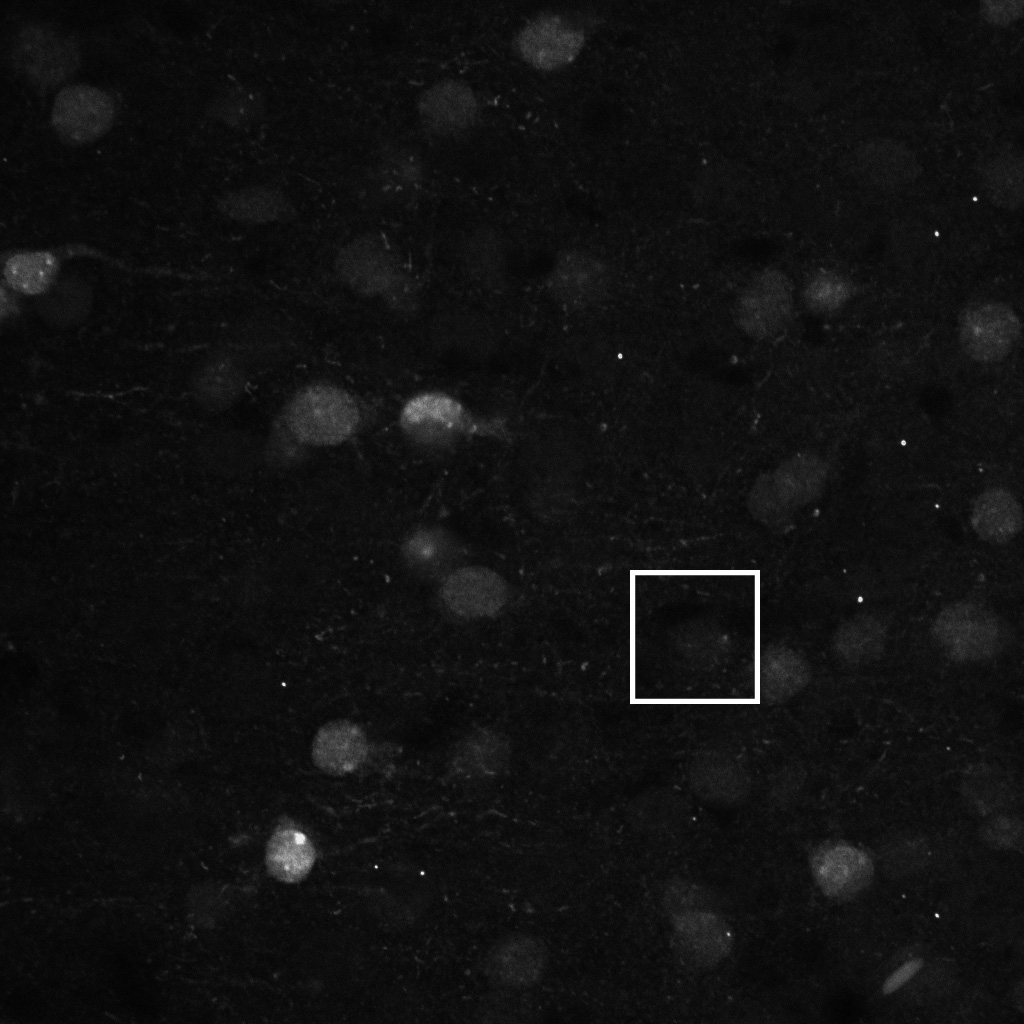

Supplement: Supplementary file 9 — Source data Fig. 1H [file 44318_2025_624_MOESM9_ESM.zip › 1H/n7/CFSE_Z stacks/8_2_C0_Z000 (12).jpg]

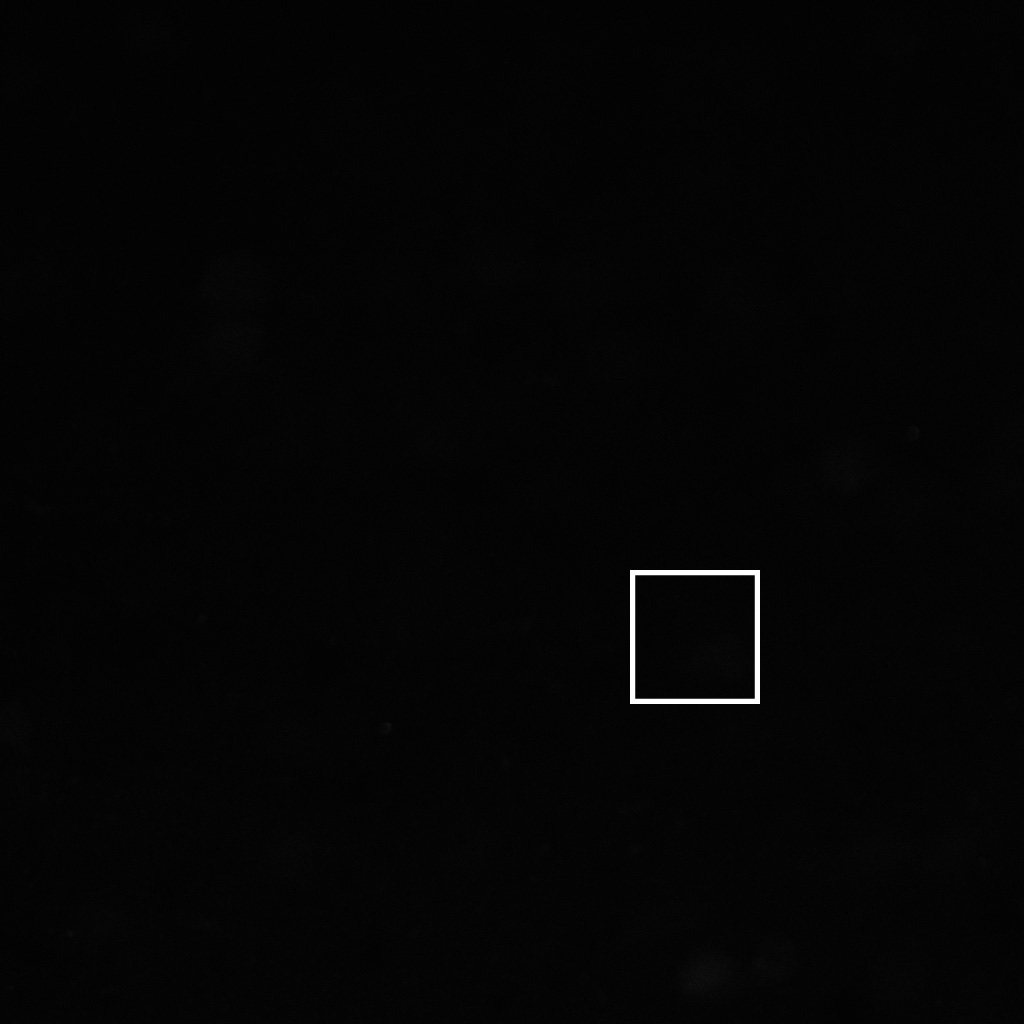

Supplement: Supplementary file 9 — Source data Fig. 1H [file 44318_2025_624_MOESM9_ESM.zip › 1H/n7/CFSE_Z stacks/8_2_C0_Z000 (45).jpg]

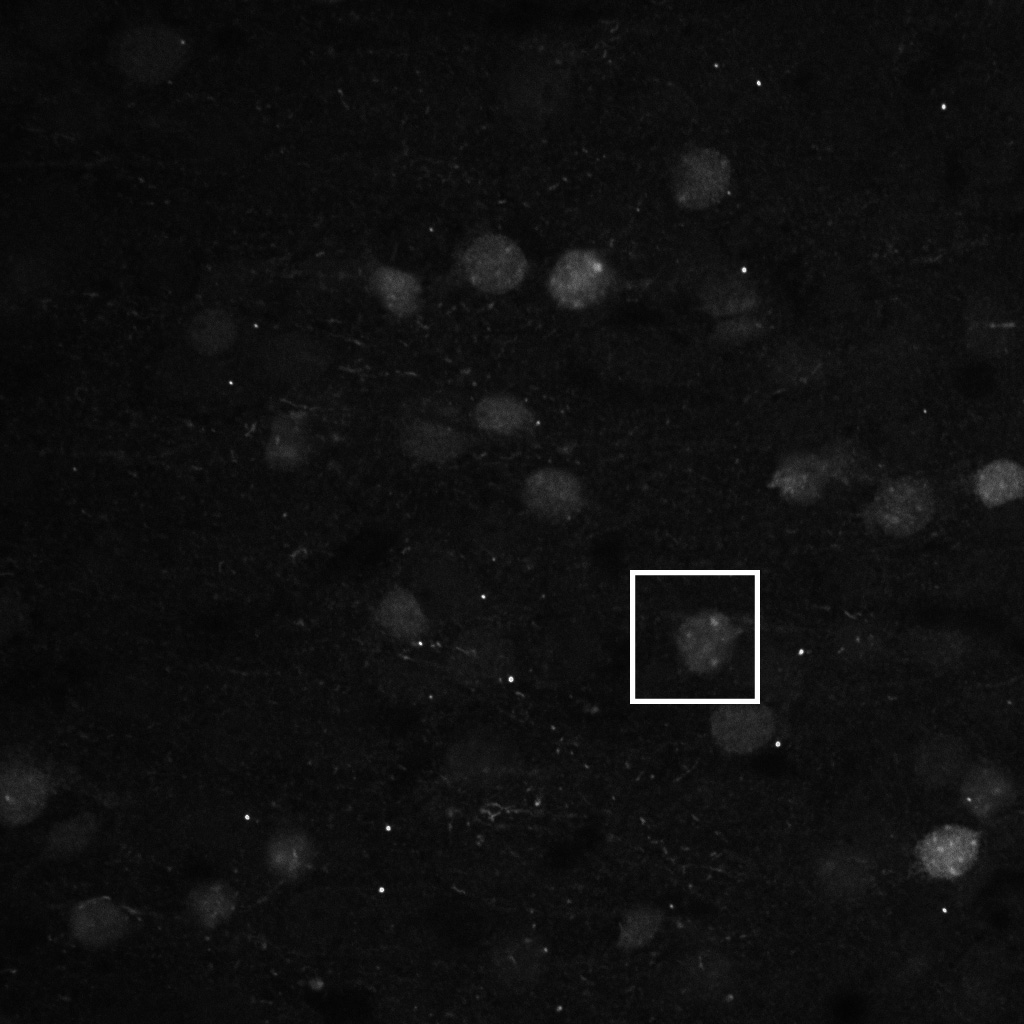

Supplement: Supplementary file 9 — Source data Fig. 1H [file 44318_2025_624_MOESM9_ESM.zip › 1H/n7/CFSE_Z stacks/8_2_C0_Z000 (28).jpg]

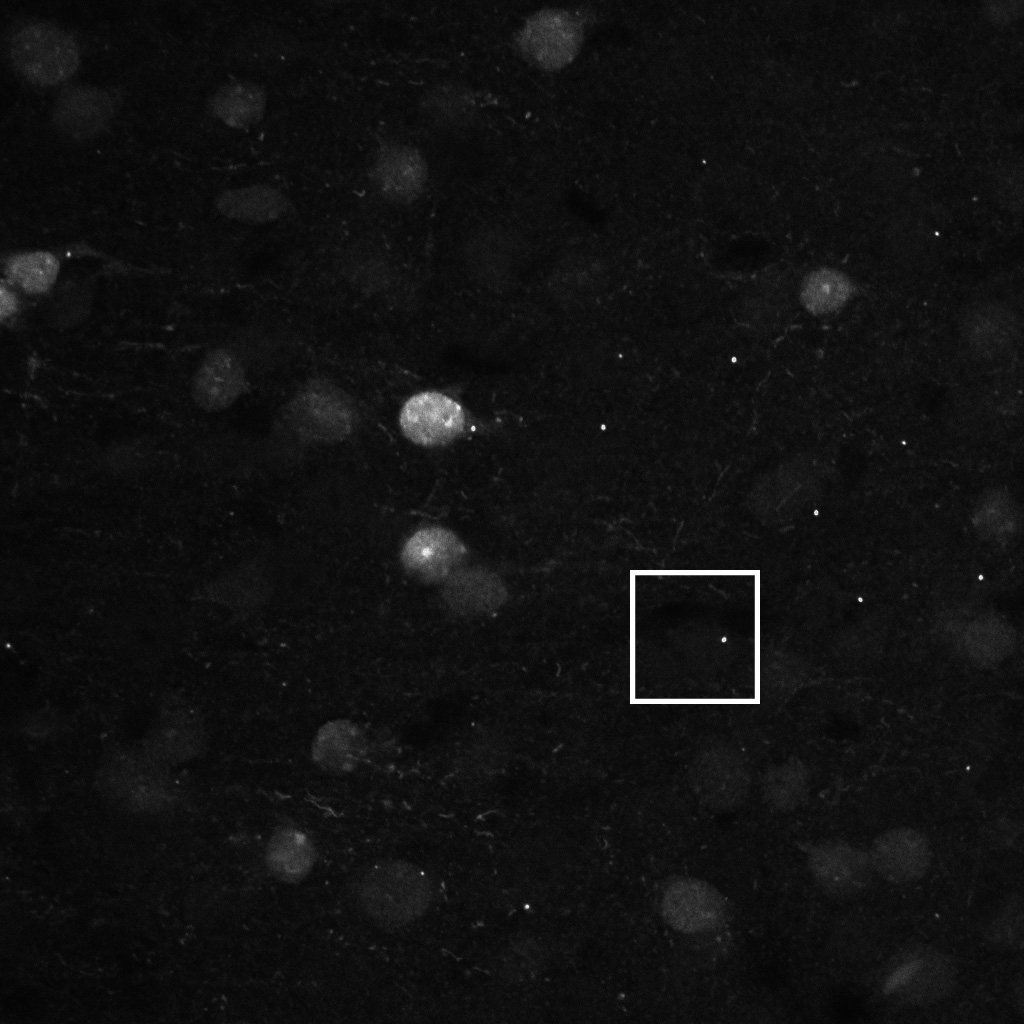

Supplement: Supplementary file 9 — Source data Fig. 1H [file 44318_2025_624_MOESM9_ESM.zip › 1H/n7/CFSE_Z stacks/8_2_C0_Z000 (8).jpg]

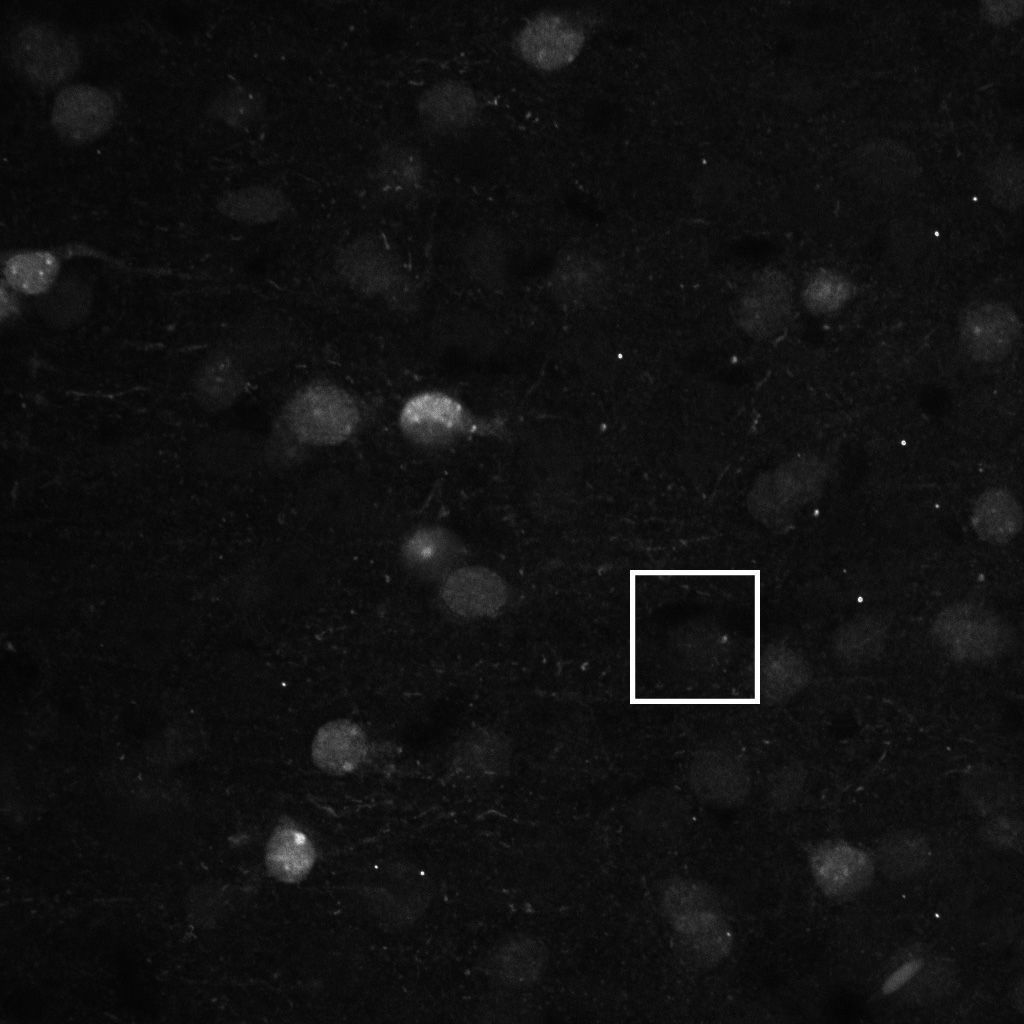

Supplement: Supplementary file 9 — Source data Fig. 1H [file 44318_2025_624_MOESM9_ESM.zip › 1H/n7/CFSE_Z stacks/8_2_C0_Z000 (11).jpg]
