# Supplementary figures and images for: Direct and indirect neurogenesis from radial glial progenitor cell clones in the mouse neocortex (part 3 of 3)
Source: EMBO J. 2025 Nov 20;45(1):182–209. doi: 10.1038/s44318-025-00624-9 (PMC12759082; doi:10.1038/s44318-025-00624-9)

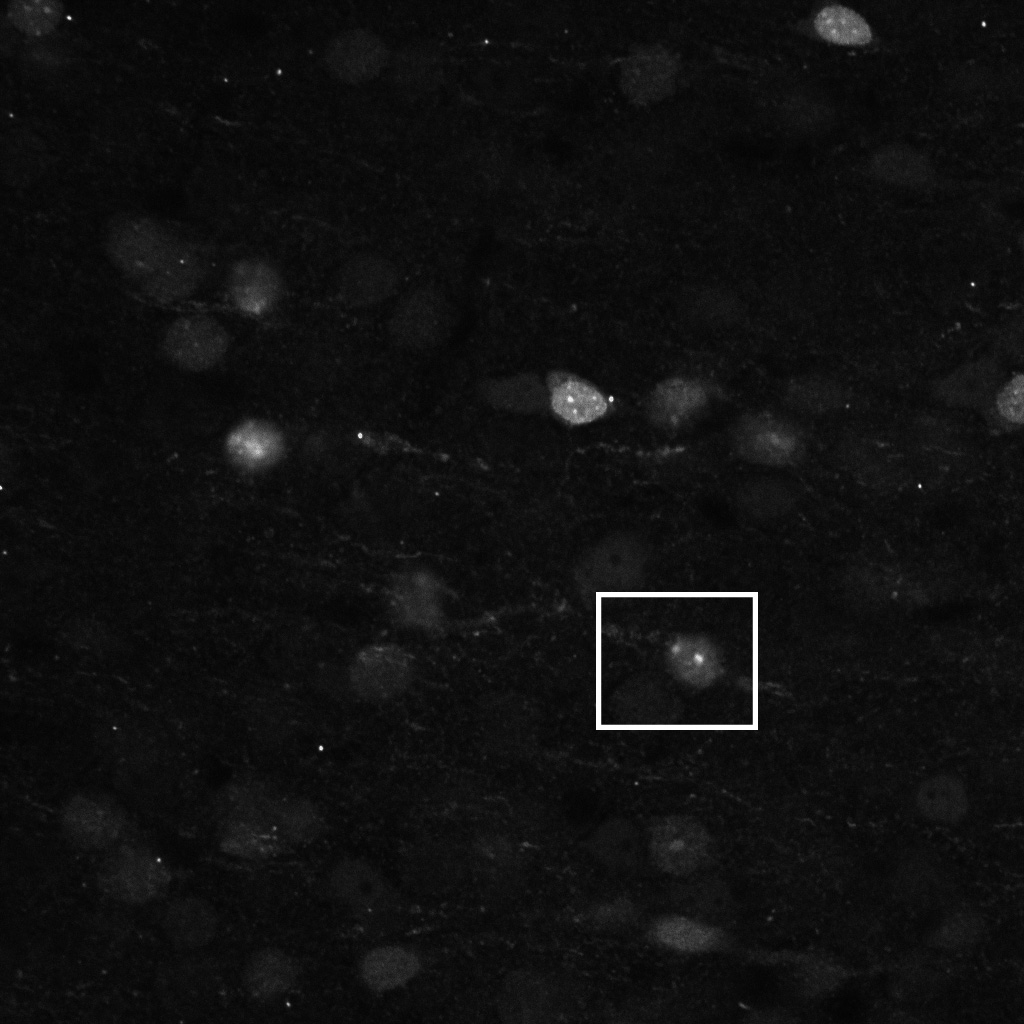

Supplement: Supplementary file 9 — Source data Fig. 1H [file 44318_2025_624_MOESM9_ESM.zip › 1H/n4/CFSE_Z stacks/8_1_C0_Z000 (17).jpg]

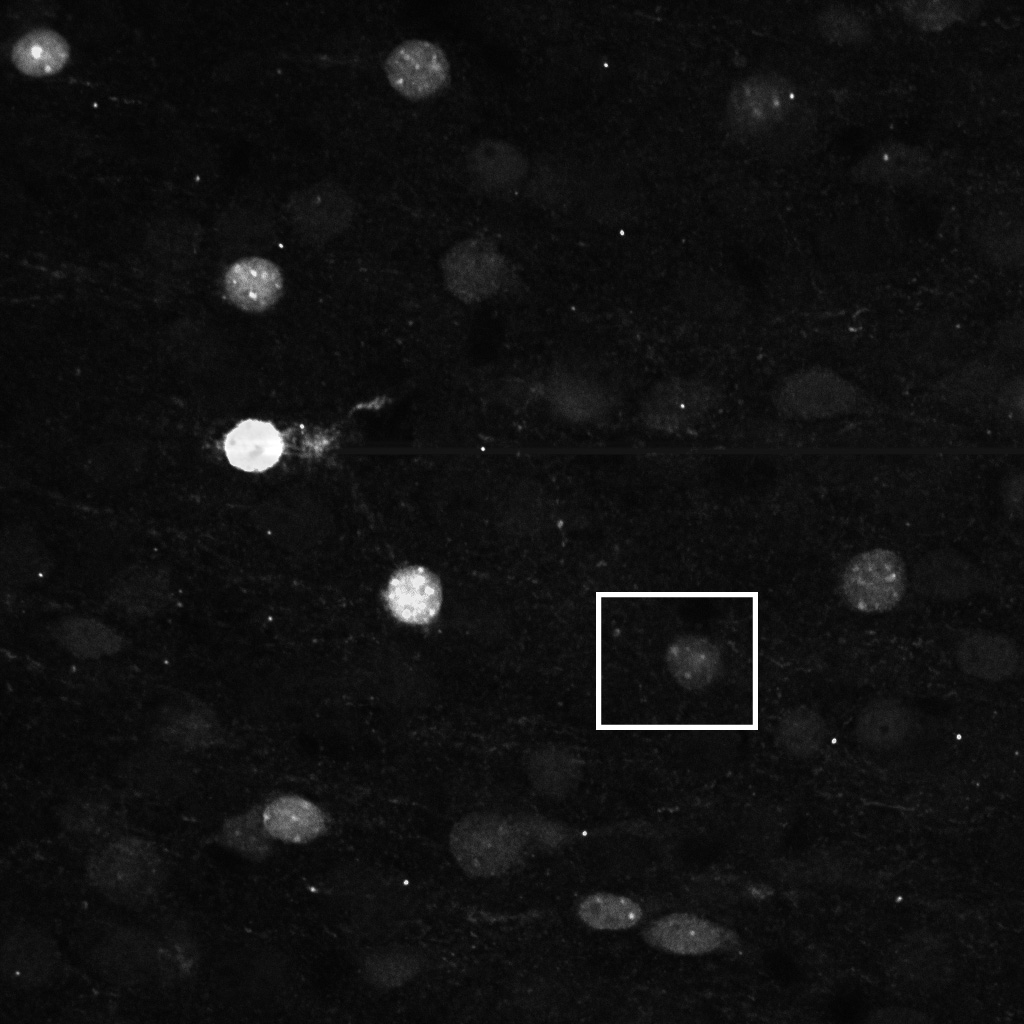

Supplement: Supplementary file 9 — Source data Fig. 1H [file 44318_2025_624_MOESM9_ESM.zip › 1H/n4/CFSE_Z stacks/8_1_C0_Z000 (6).jpg]

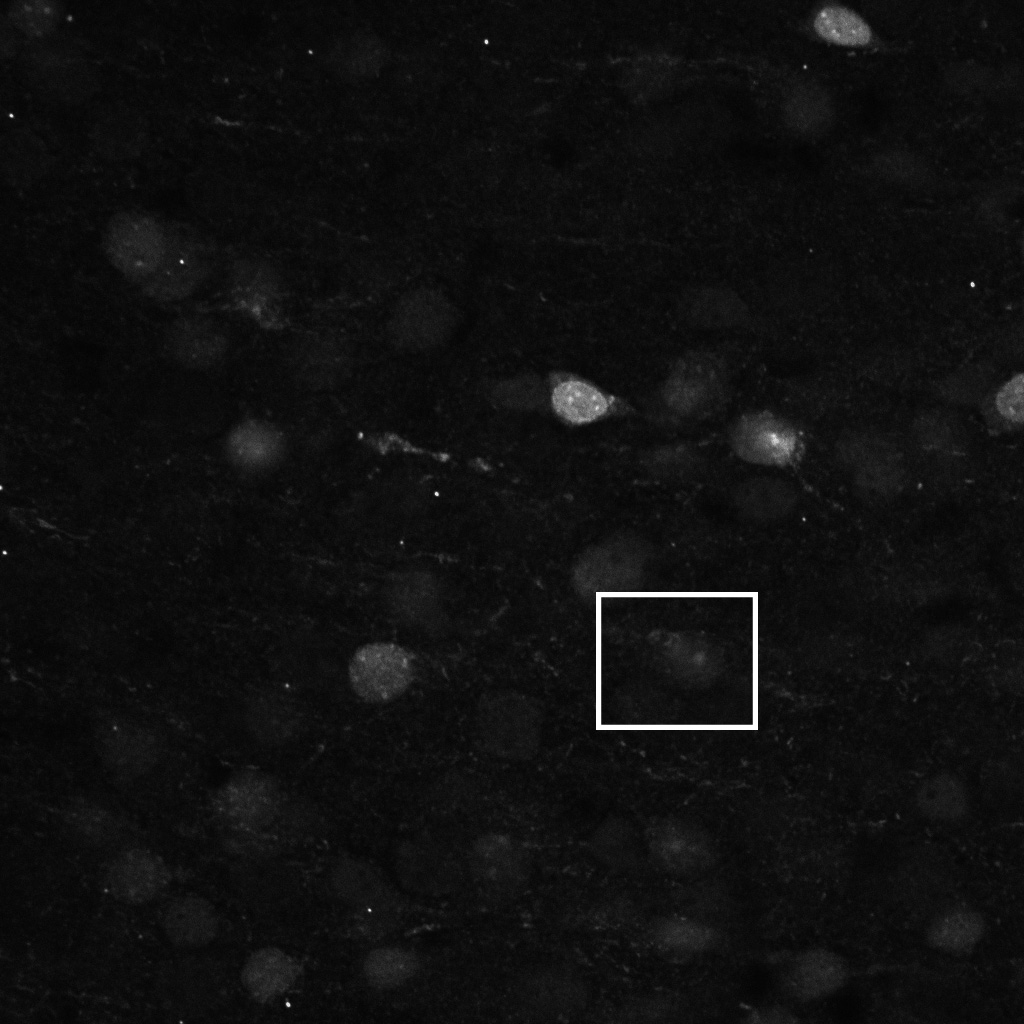

Supplement: Supplementary file 9 — Source data Fig. 1H [file 44318_2025_624_MOESM9_ESM.zip › 1H/n4/CFSE_Z stacks/8_1_C0_Z000 (21).jpg]

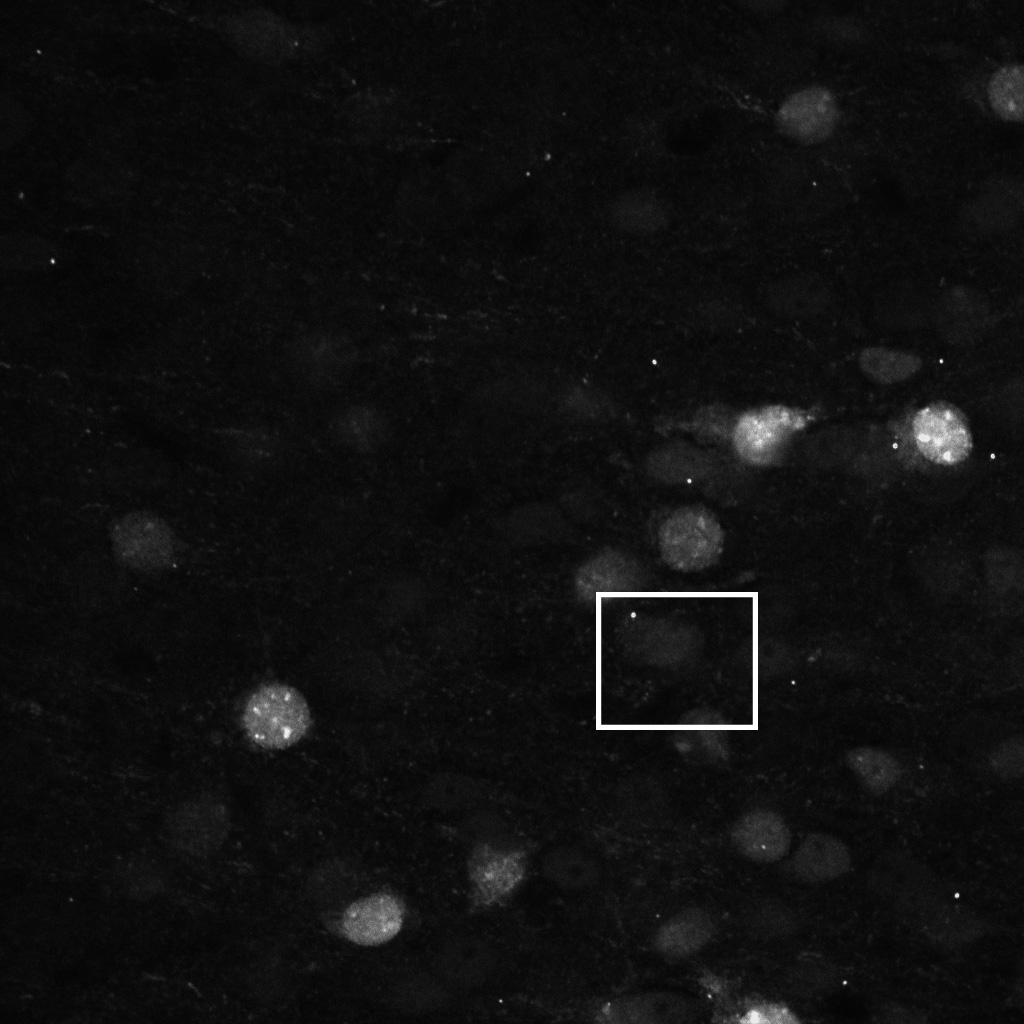

Supplement: Supplementary file 9 — Source data Fig. 1H [file 44318_2025_624_MOESM9_ESM.zip › 1H/n4/CFSE_Z stacks/8_1_C0_Z000 (37).jpg]

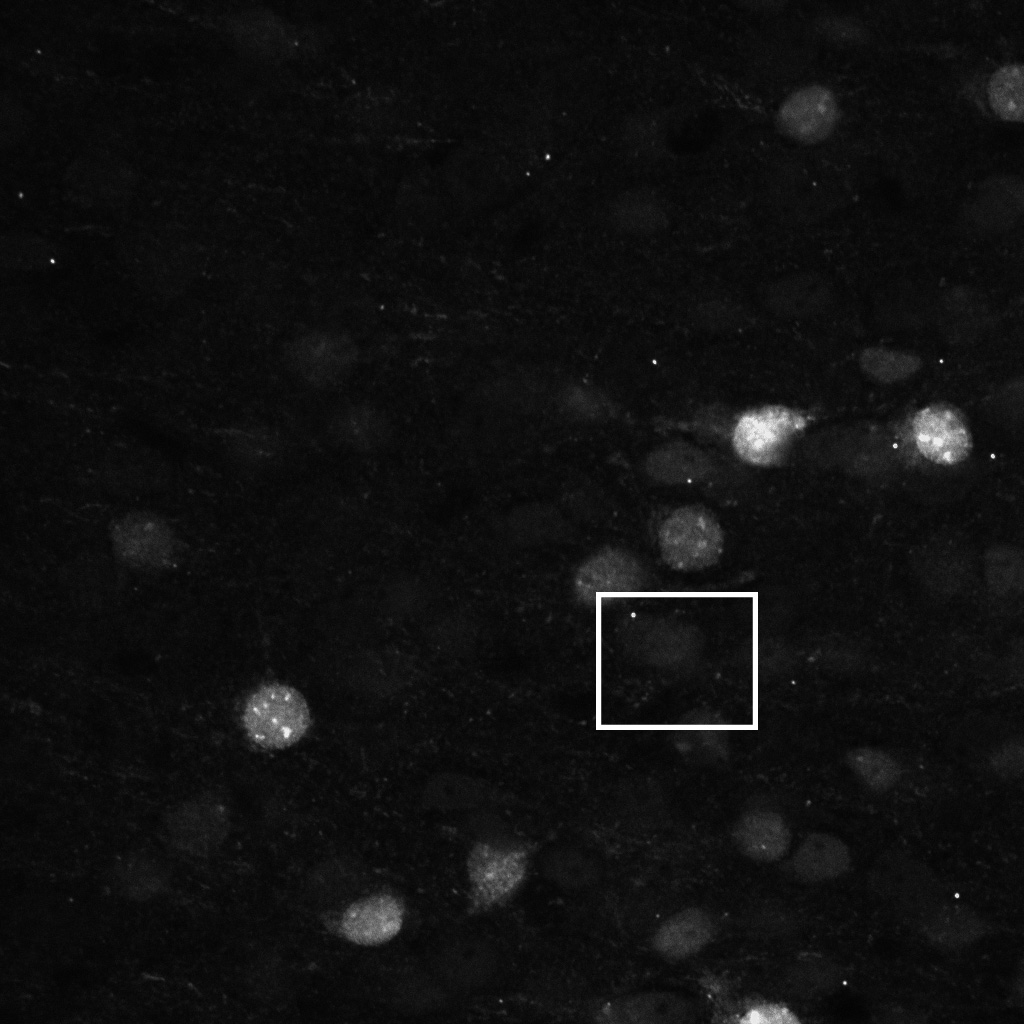

Supplement: Supplementary file 9 — Source data Fig. 1H [file 44318_2025_624_MOESM9_ESM.zip › 1H/n4/CFSE_Z stacks/8_1_C0_Z000 (36).jpg]

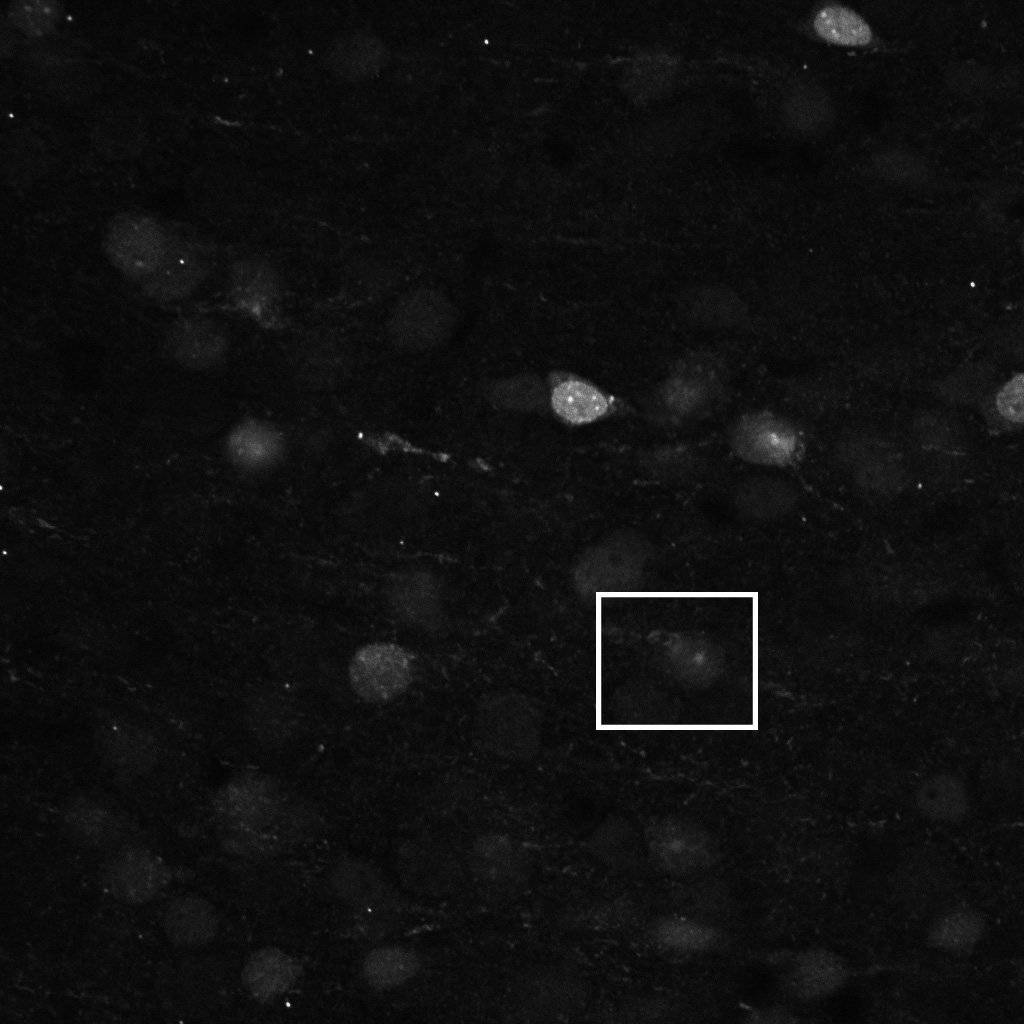

Supplement: Supplementary file 9 — Source data Fig. 1H [file 44318_2025_624_MOESM9_ESM.zip › 1H/n4/CFSE_Z stacks/8_1_C0_Z000 (20).jpg]

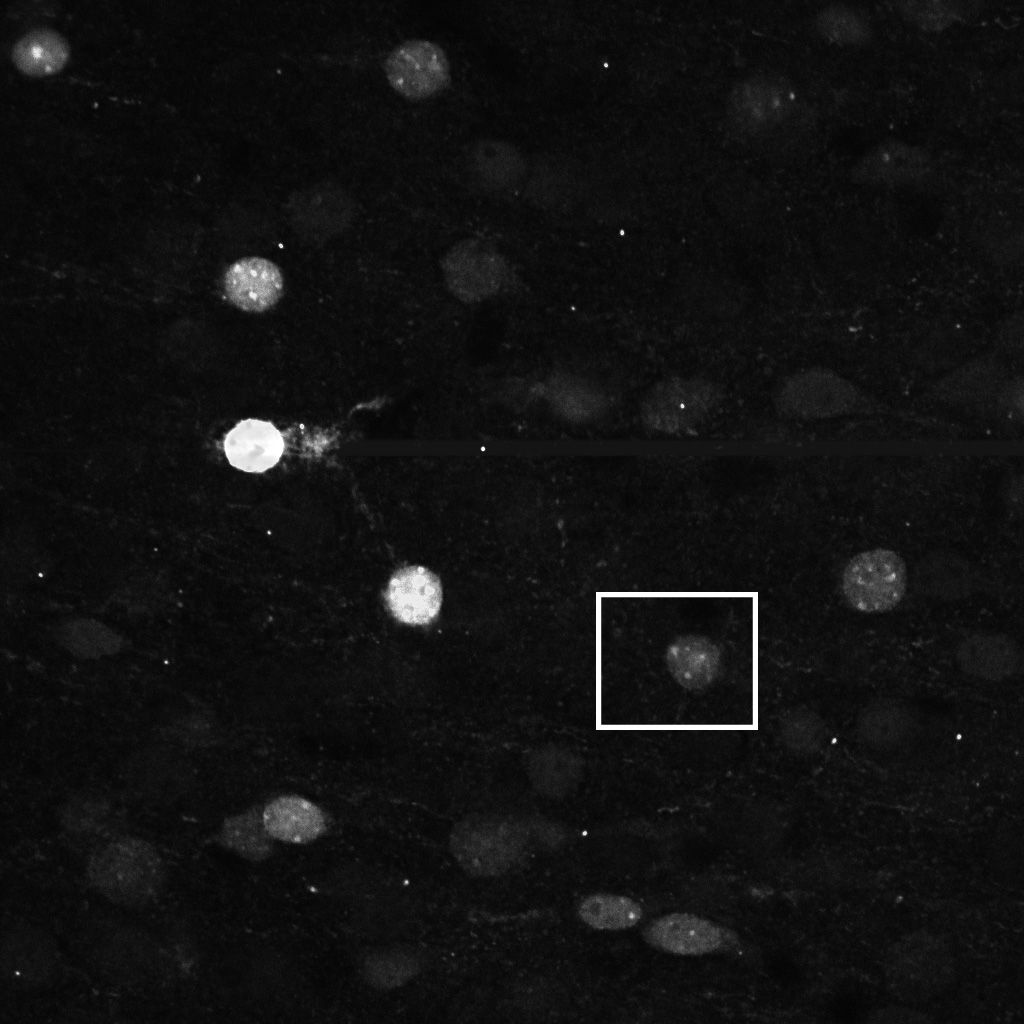

Supplement: Supplementary file 9 — Source data Fig. 1H [file 44318_2025_624_MOESM9_ESM.zip › 1H/n4/CFSE_Z stacks/8_1_C0_Z000 (7).jpg]

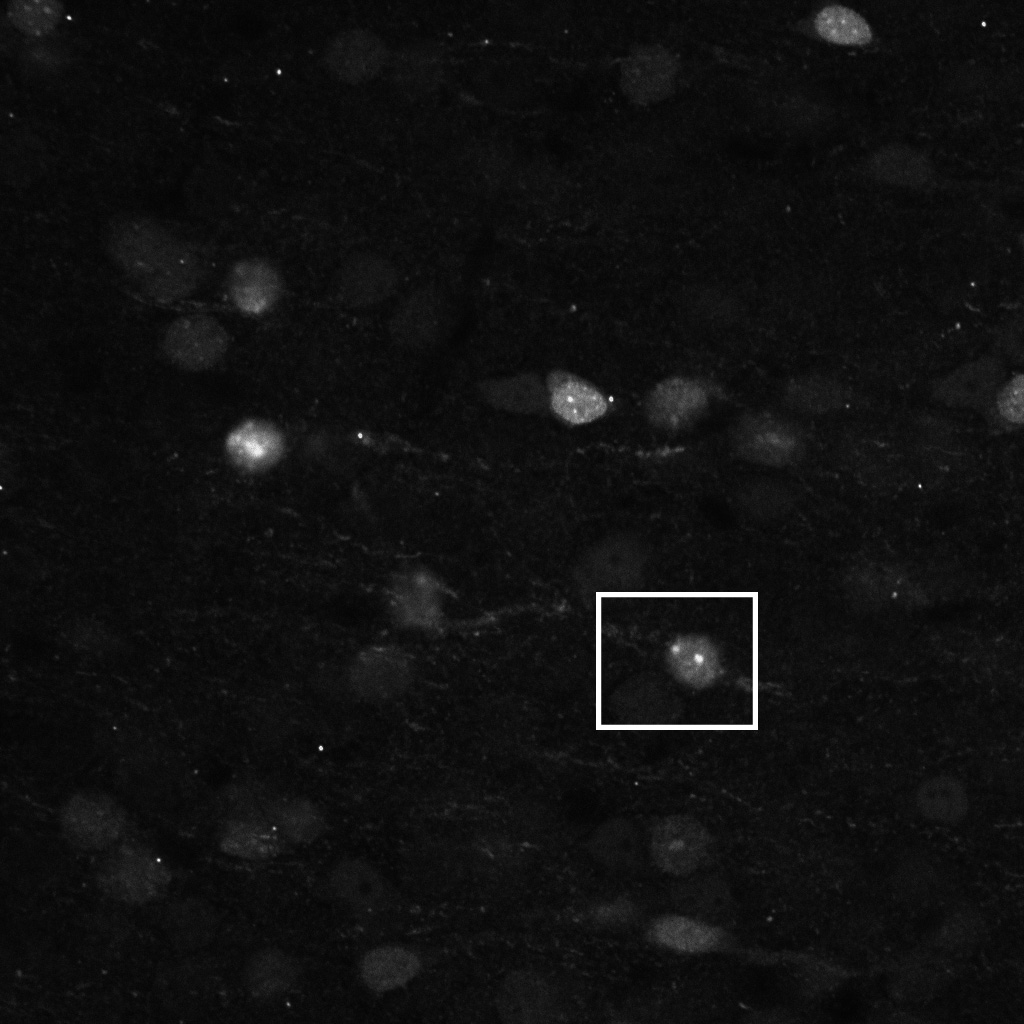

Supplement: Supplementary file 9 — Source data Fig. 1H [file 44318_2025_624_MOESM9_ESM.zip › 1H/n4/CFSE_Z stacks/8_1_C0_Z000 (16).jpg]

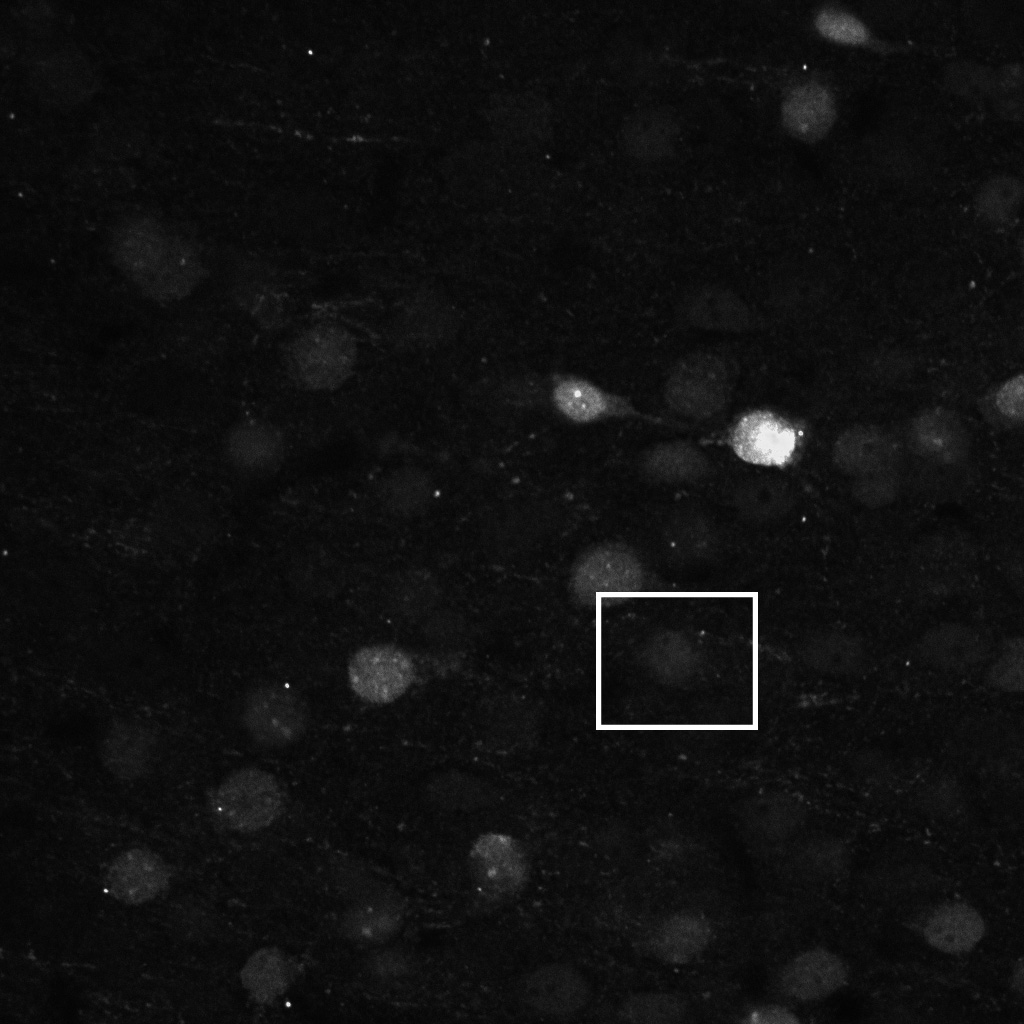

Supplement: Supplementary file 9 — Source data Fig. 1H [file 44318_2025_624_MOESM9_ESM.zip › 1H/n4/CFSE_Z stacks/8_1_C0_Z000 (27).jpg]

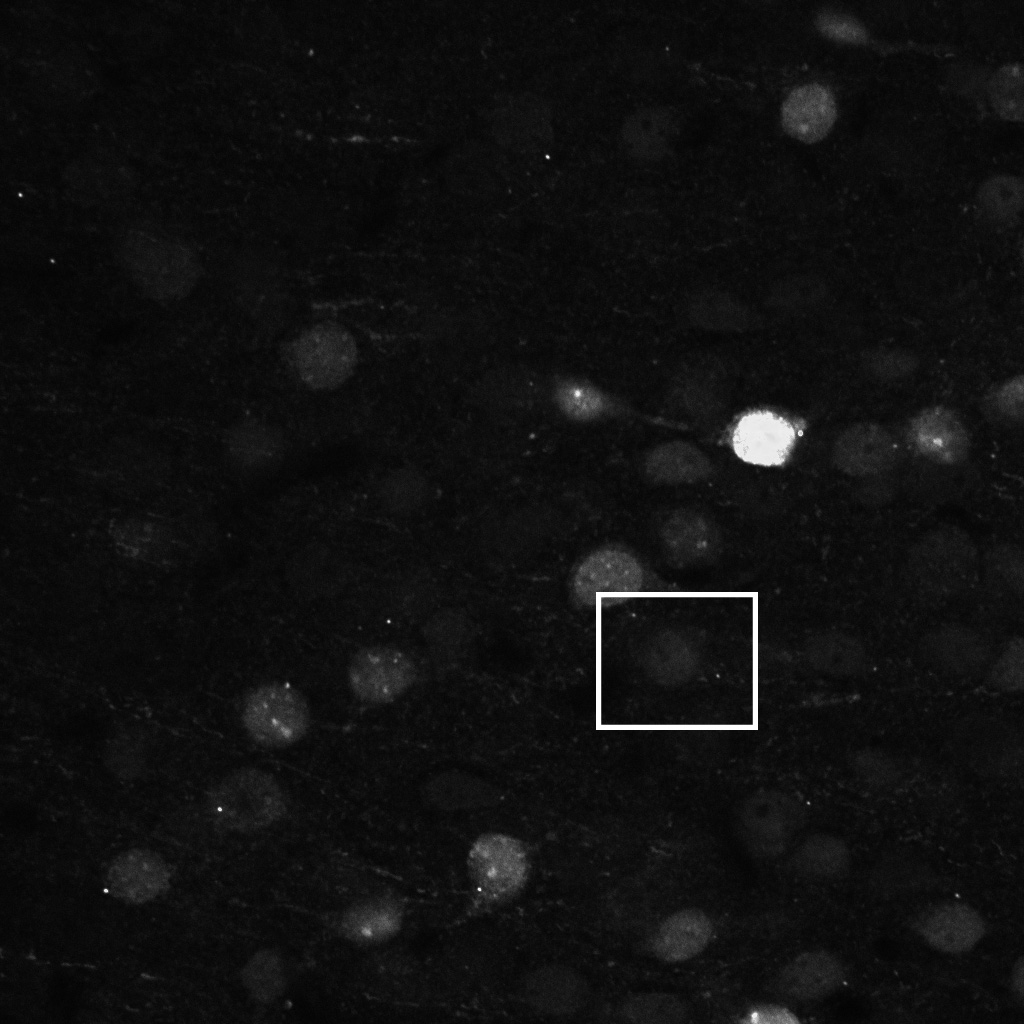

Supplement: Supplementary file 9 — Source data Fig. 1H [file 44318_2025_624_MOESM9_ESM.zip › 1H/n4/CFSE_Z stacks/8_1_C0_Z000 (31).jpg]

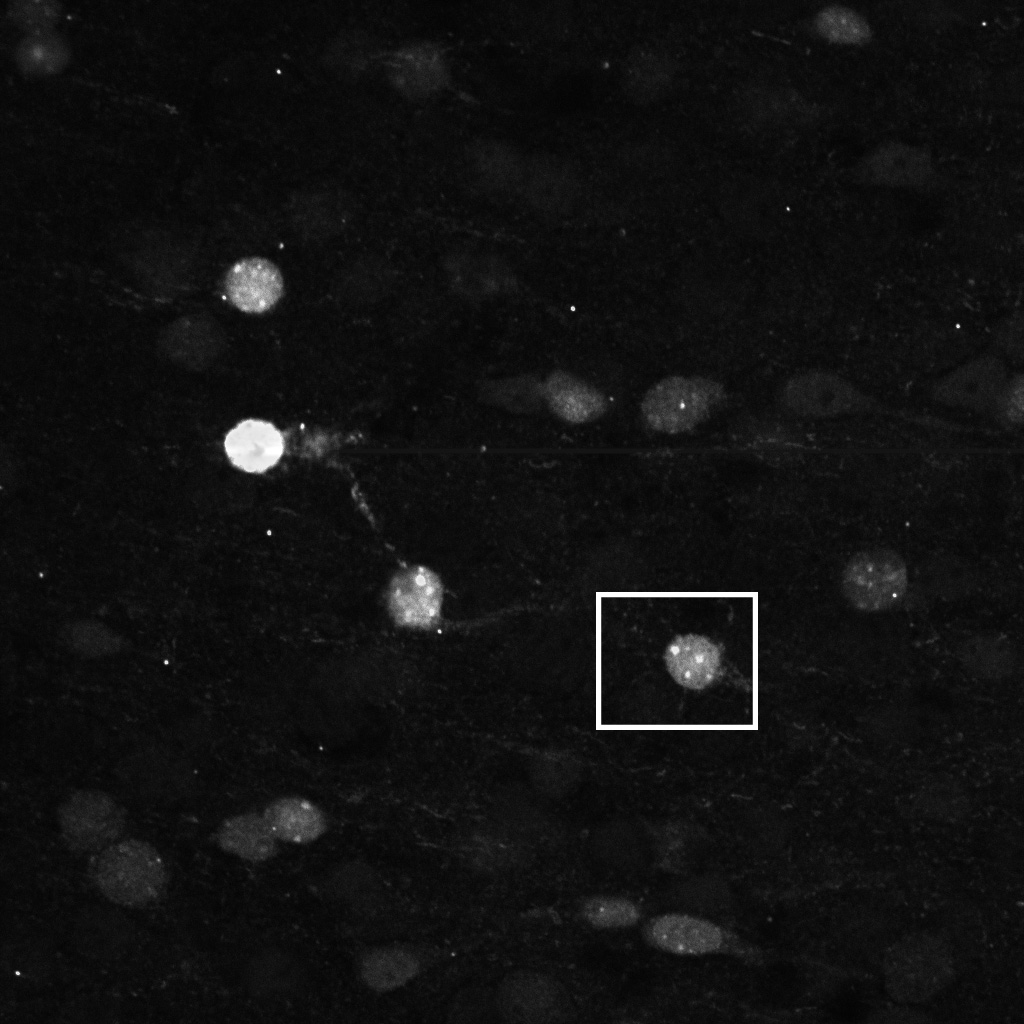

Supplement: Supplementary file 9 — Source data Fig. 1H [file 44318_2025_624_MOESM9_ESM.zip › 1H/n4/CFSE_Z stacks/8_1_C0_Z000 (11).jpg]

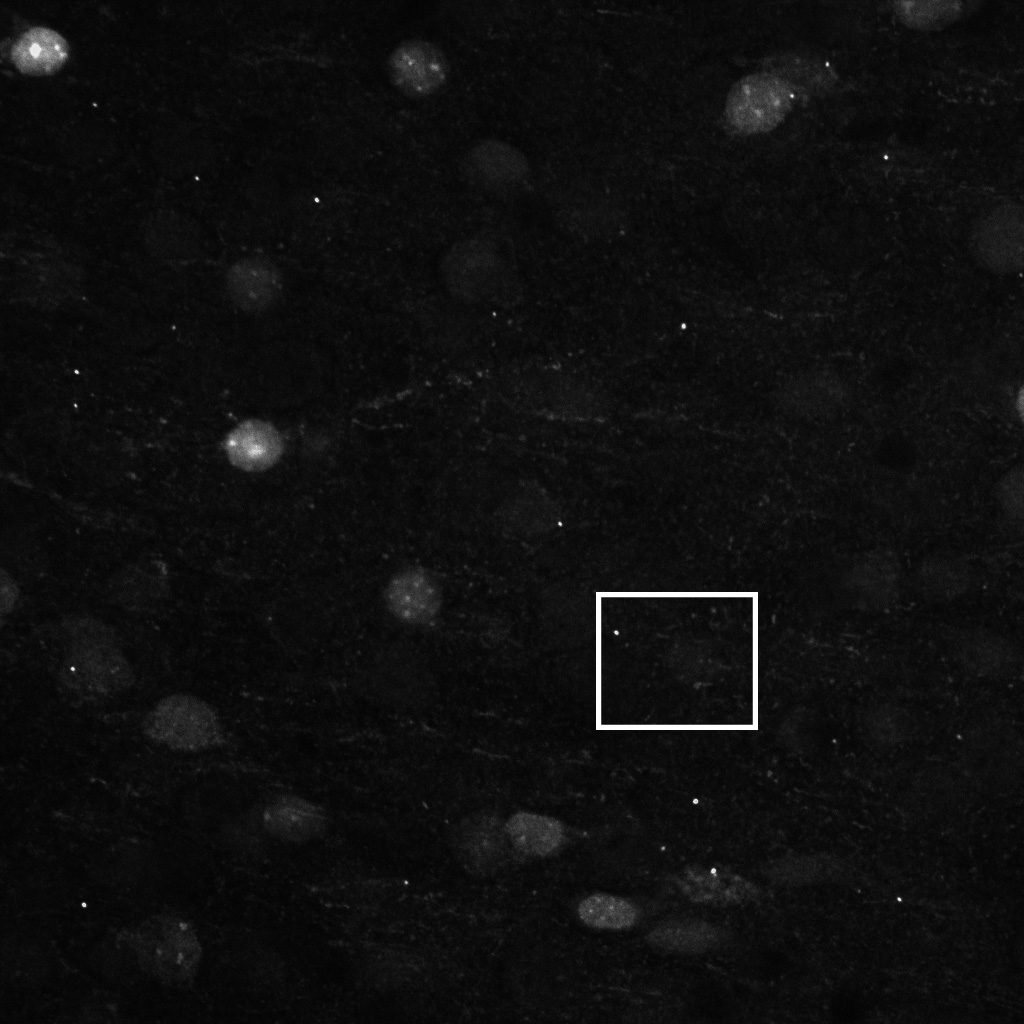

Supplement: Supplementary file 9 — Source data Fig. 1H [file 44318_2025_624_MOESM9_ESM.zip › 1H/n4/CFSE_Z stacks/8_1_C0_Z000 (1).jpg]

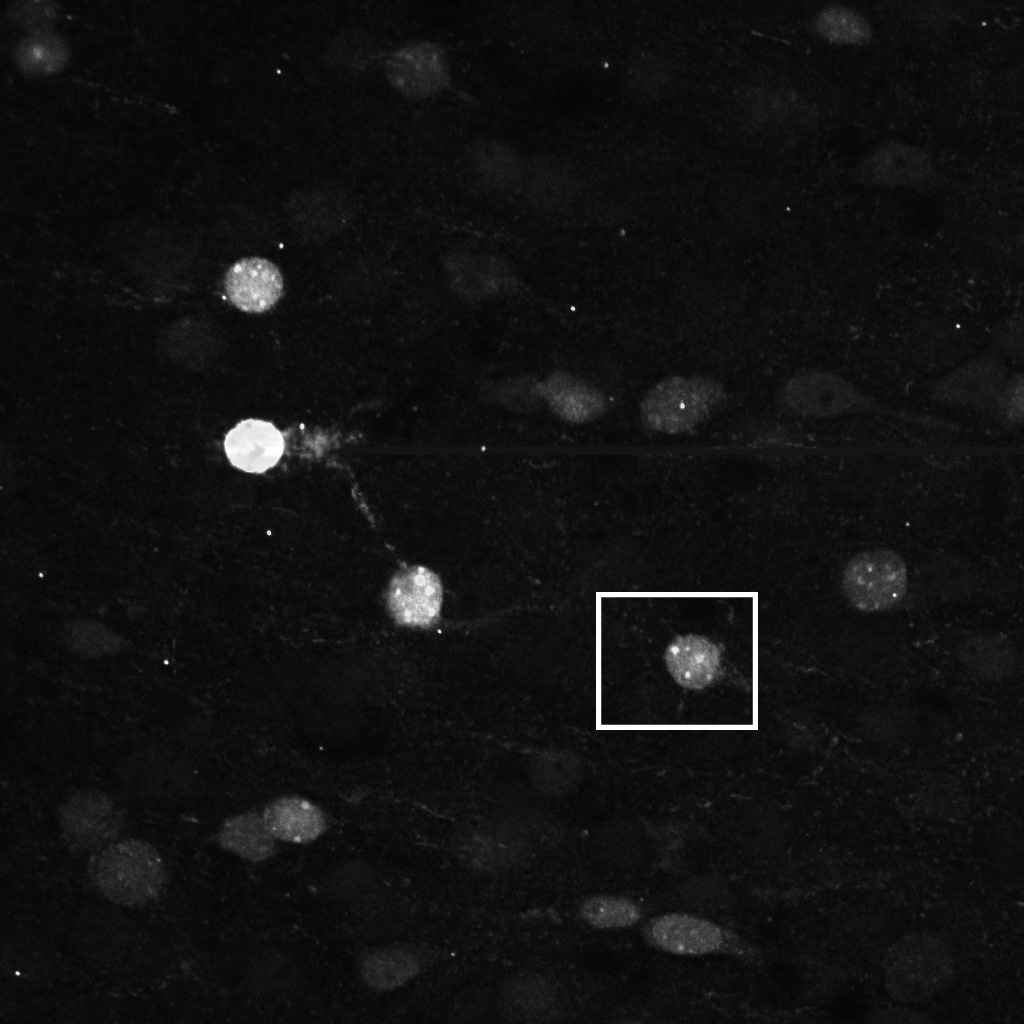

Supplement: Supplementary file 9 — Source data Fig. 1H [file 44318_2025_624_MOESM9_ESM.zip › 1H/n4/CFSE_Z stacks/8_1_C0_Z000 (10).jpg]

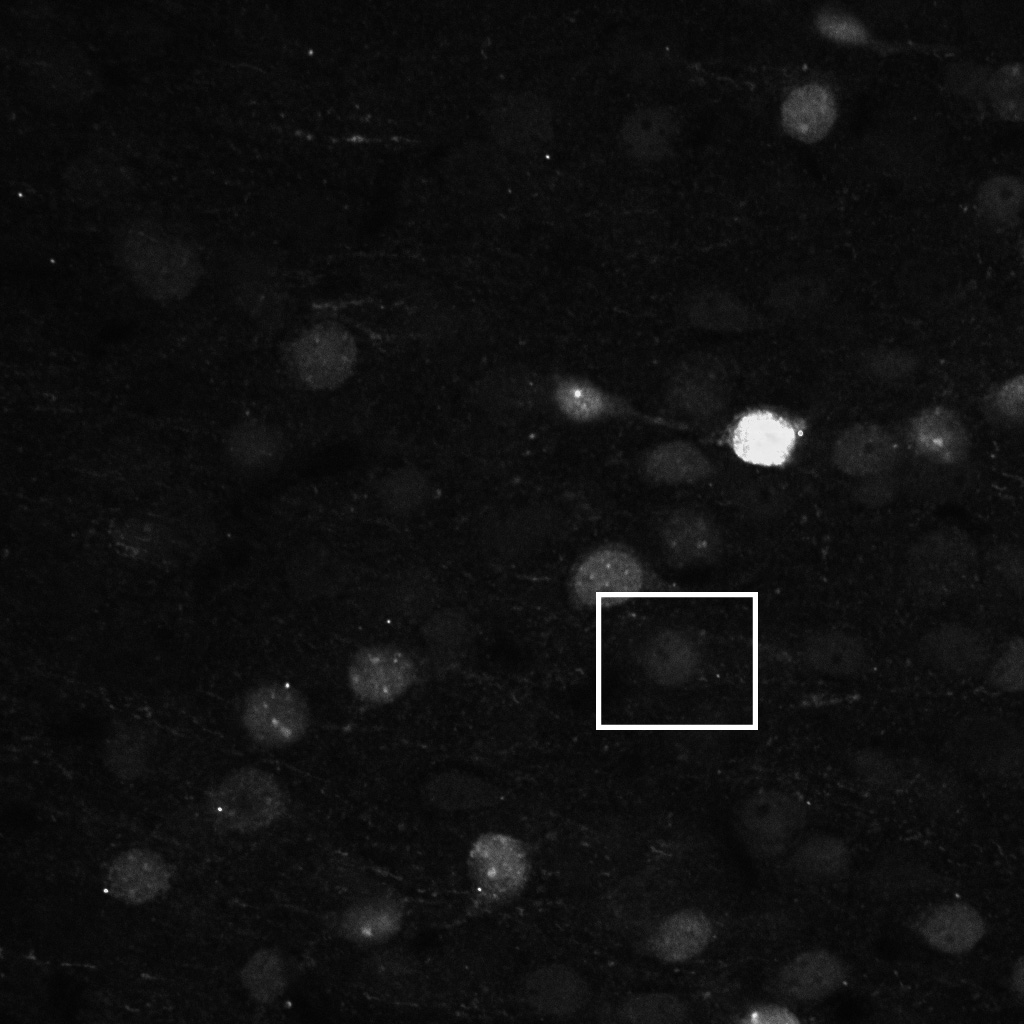

Supplement: Supplementary file 9 — Source data Fig. 1H [file 44318_2025_624_MOESM9_ESM.zip › 1H/n4/CFSE_Z stacks/8_1_C0_Z000 (30).jpg]

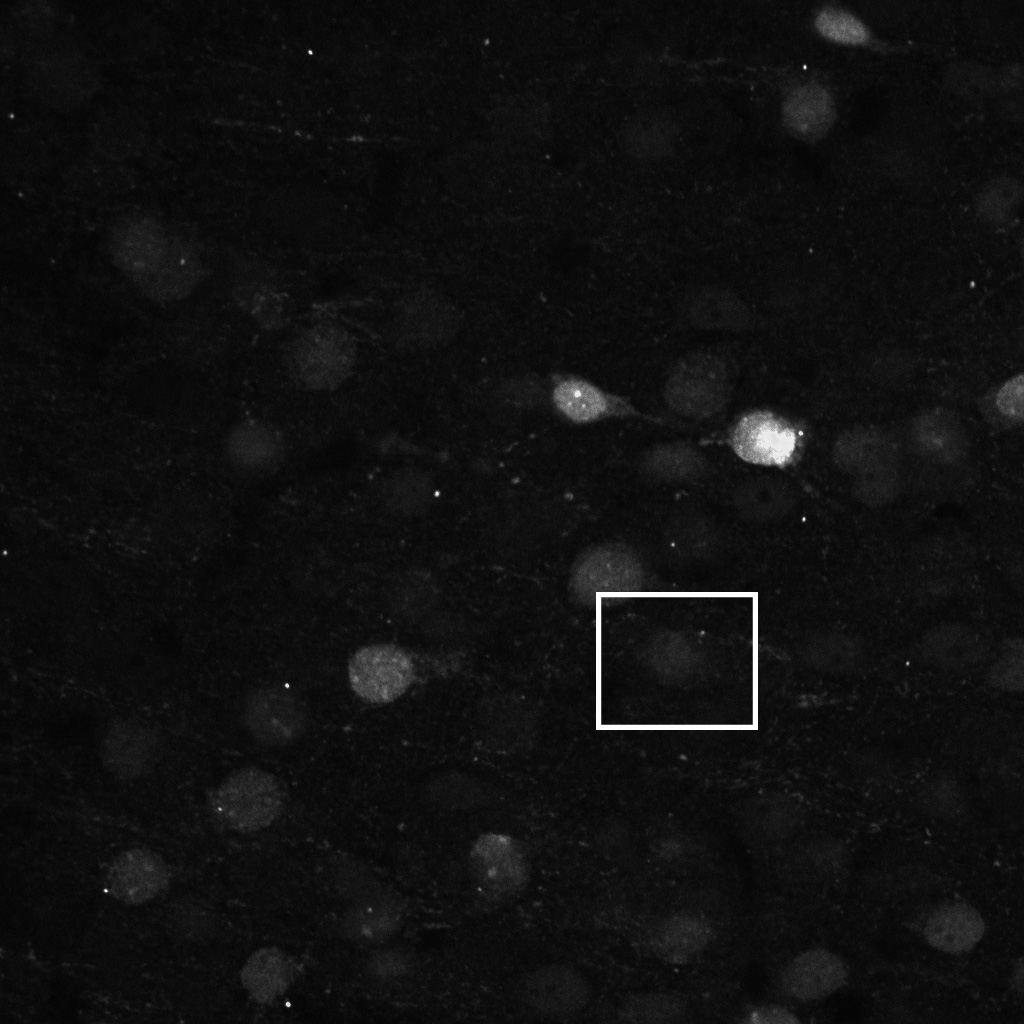

Supplement: Supplementary file 9 — Source data Fig. 1H [file 44318_2025_624_MOESM9_ESM.zip › 1H/n4/CFSE_Z stacks/8_1_C0_Z000 (26).jpg]

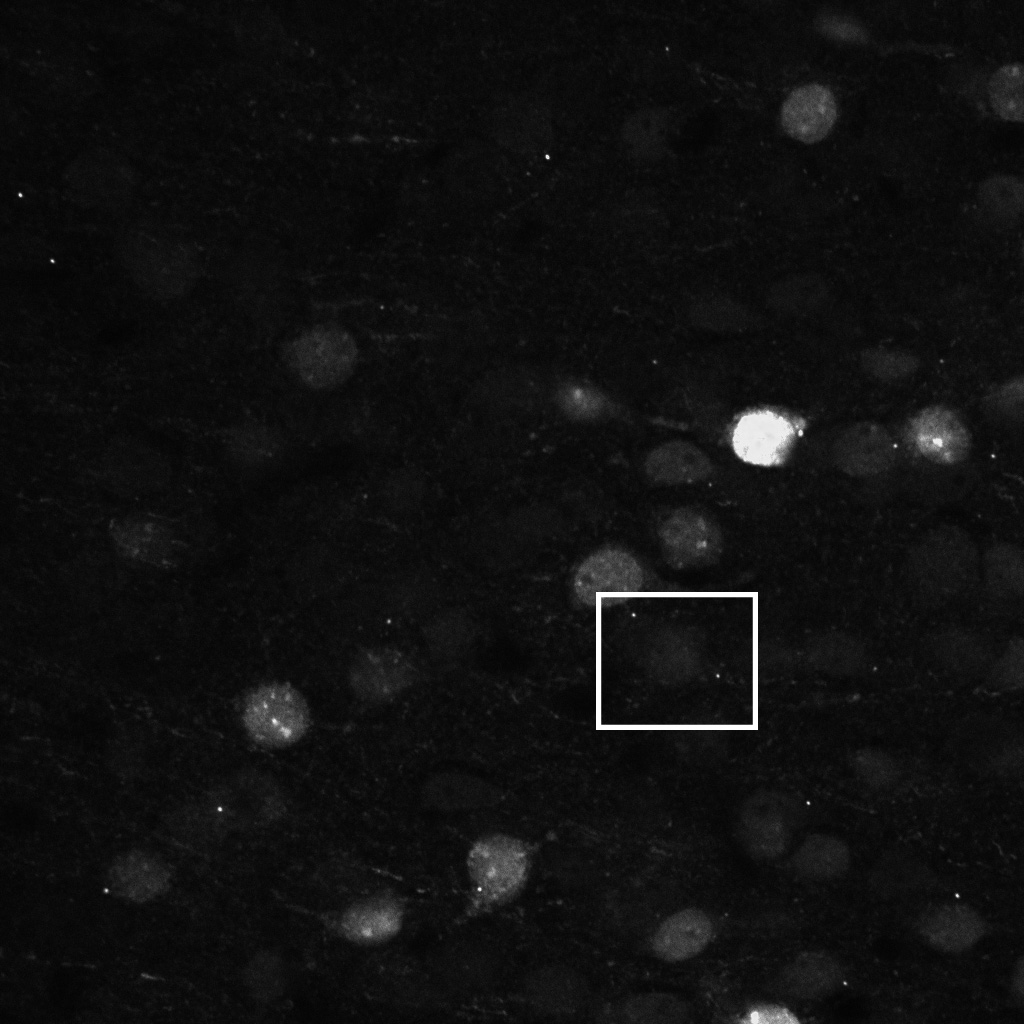

Supplement: Supplementary file 9 — Source data Fig. 1H [file 44318_2025_624_MOESM9_ESM.zip › 1H/n4/CFSE_Z stacks/8_1_C0_Z000 (33).jpg]

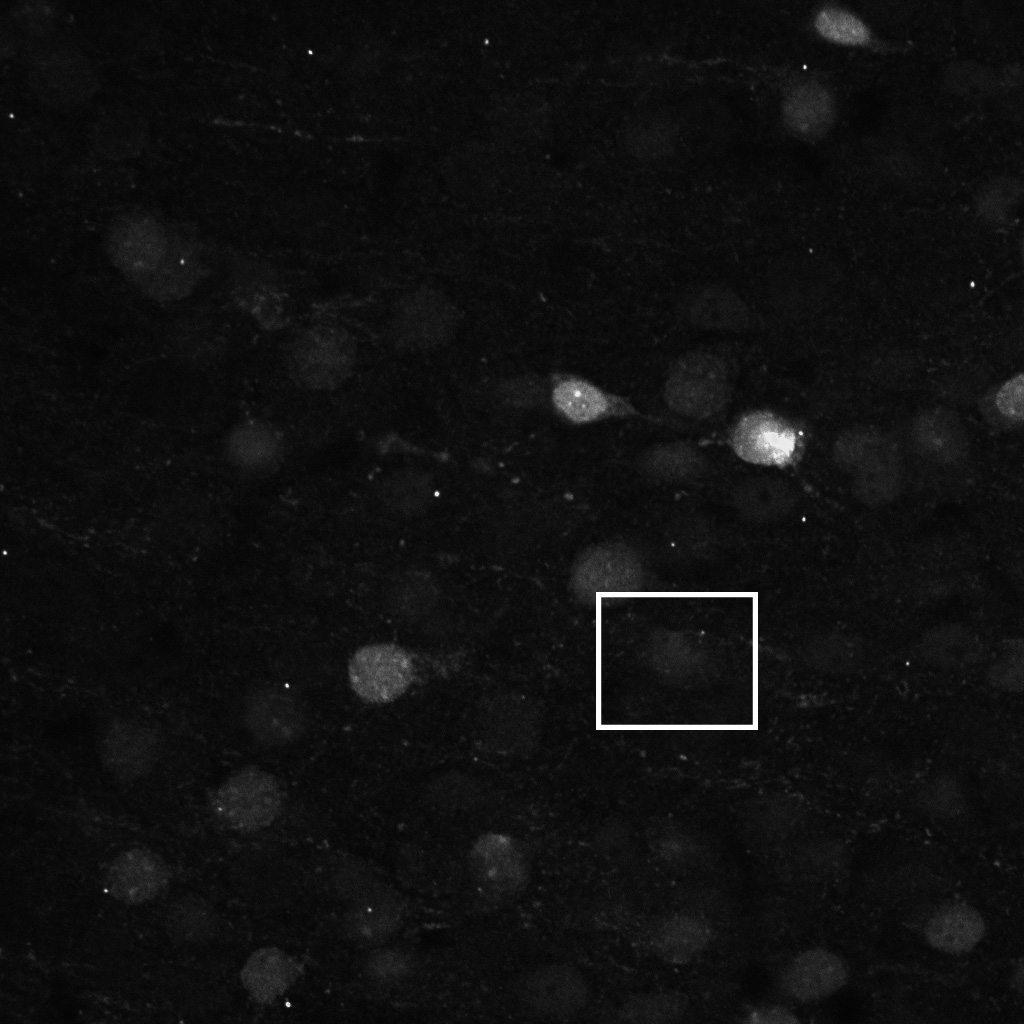

Supplement: Supplementary file 9 — Source data Fig. 1H [file 44318_2025_624_MOESM9_ESM.zip › 1H/n4/CFSE_Z stacks/8_1_C0_Z000 (25).jpg]

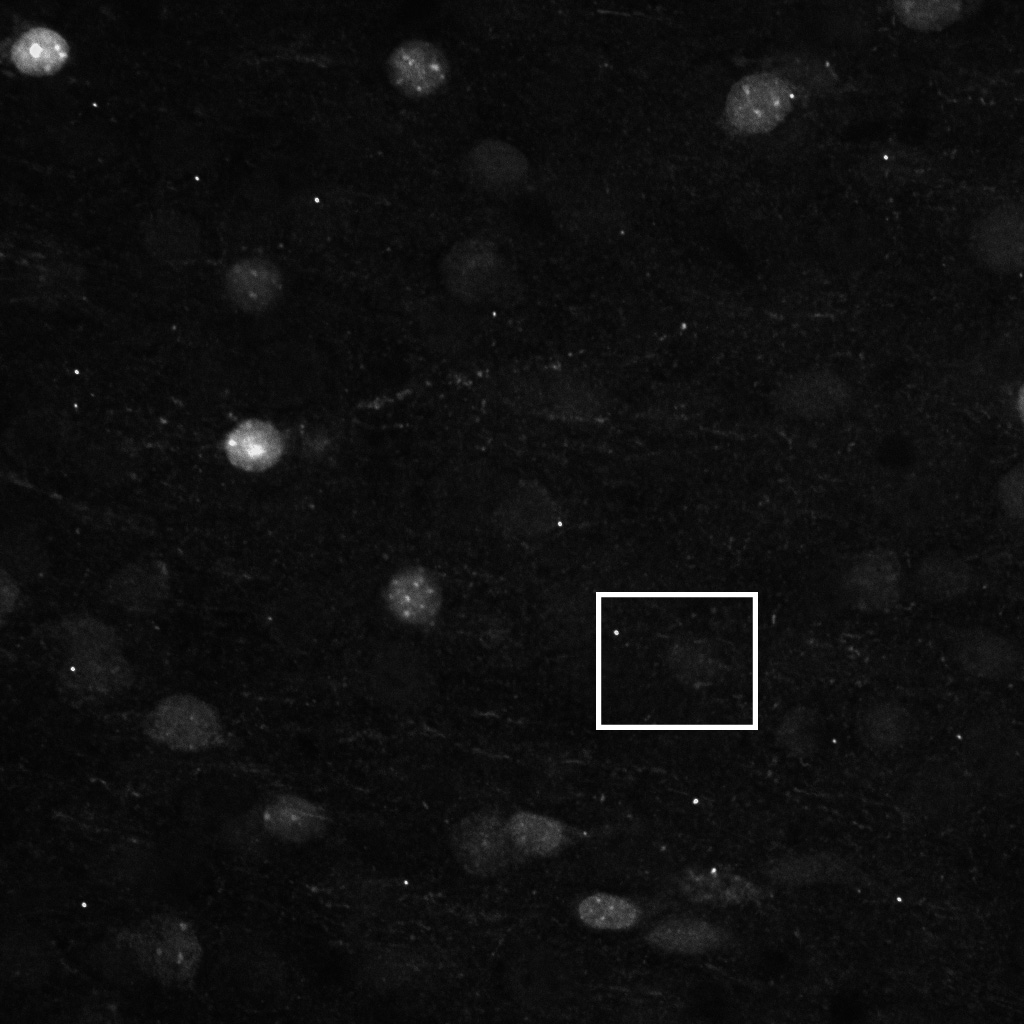

Supplement: Supplementary file 9 — Source data Fig. 1H [file 44318_2025_624_MOESM9_ESM.zip › 1H/n4/CFSE_Z stacks/8_1_C0_Z000 (2).jpg]

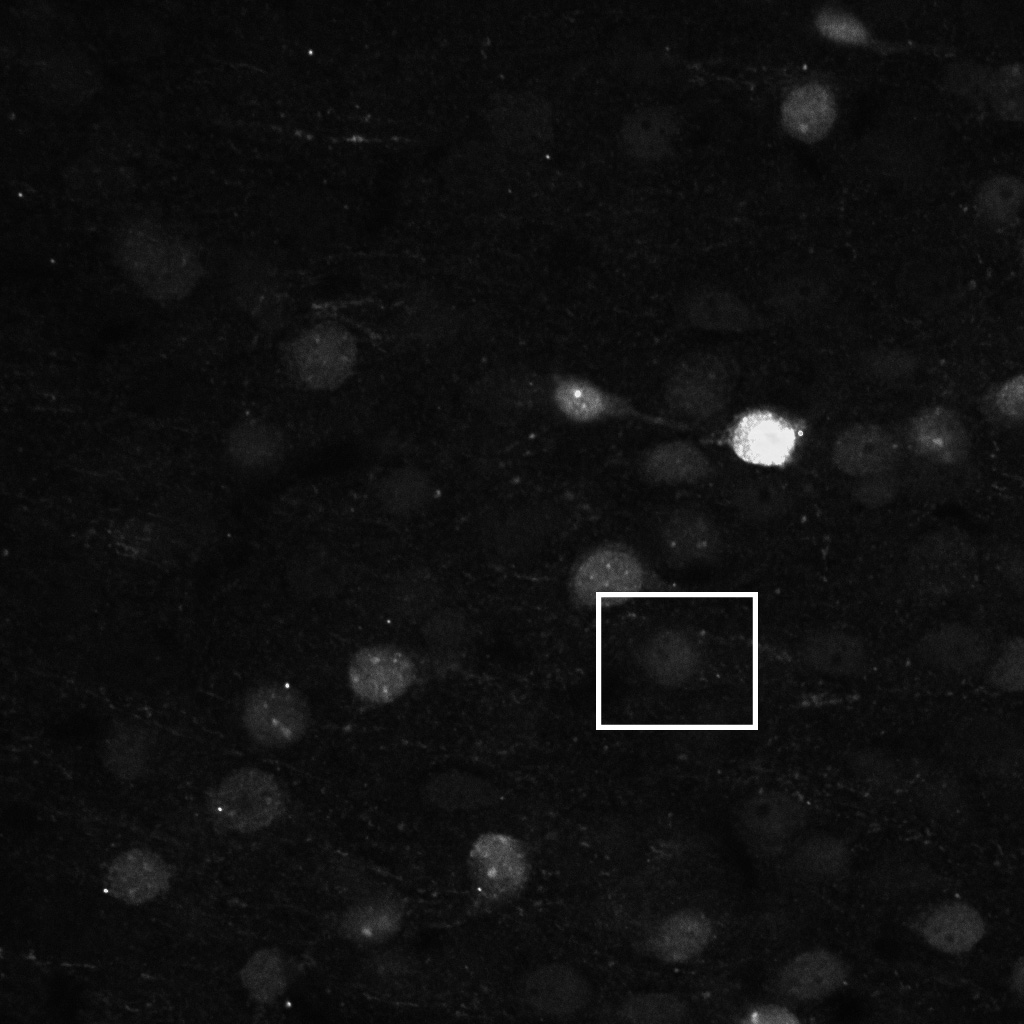

Supplement: Supplementary file 9 — Source data Fig. 1H [file 44318_2025_624_MOESM9_ESM.zip › 1H/n4/CFSE_Z stacks/8_1_C0_Z000 (29).jpg]

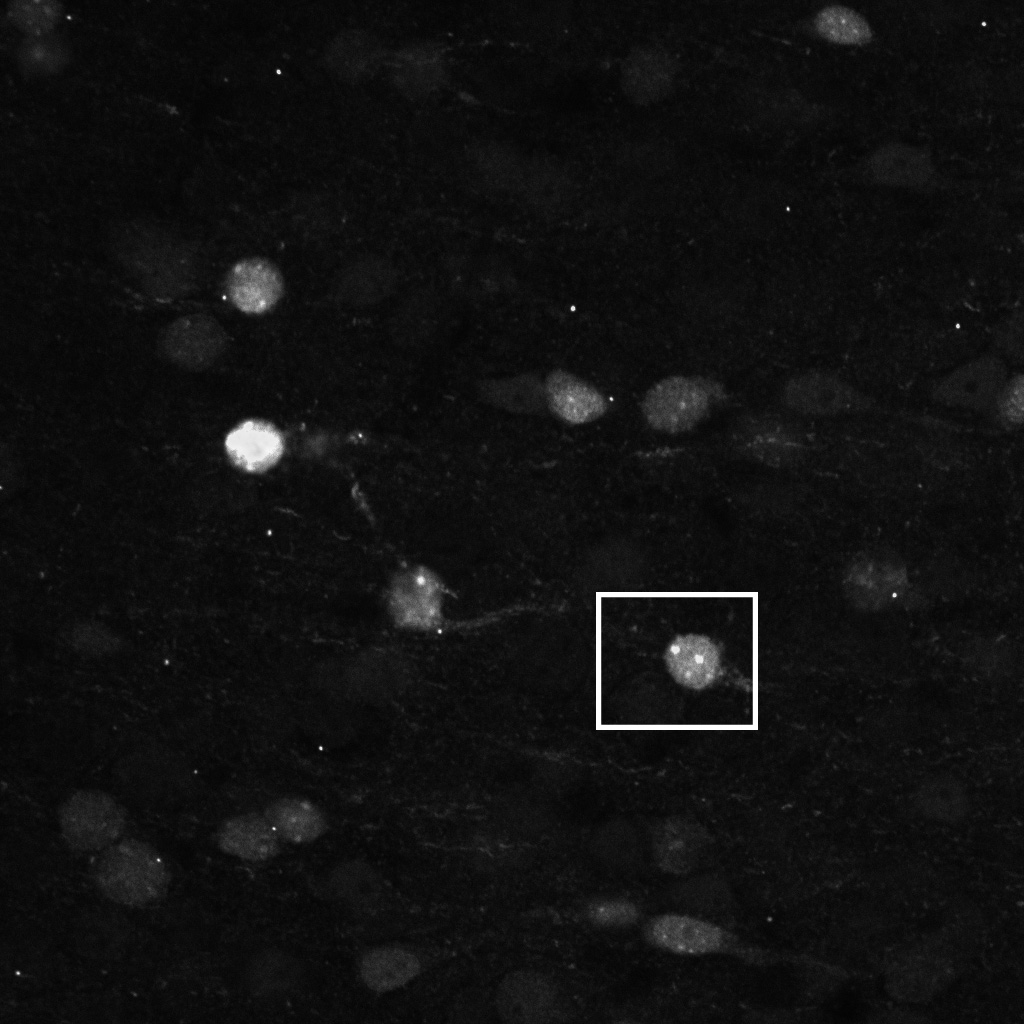

Supplement: Supplementary file 9 — Source data Fig. 1H [file 44318_2025_624_MOESM9_ESM.zip › 1H/n4/CFSE_Z stacks/8_1_C0_Z000 (13).jpg]

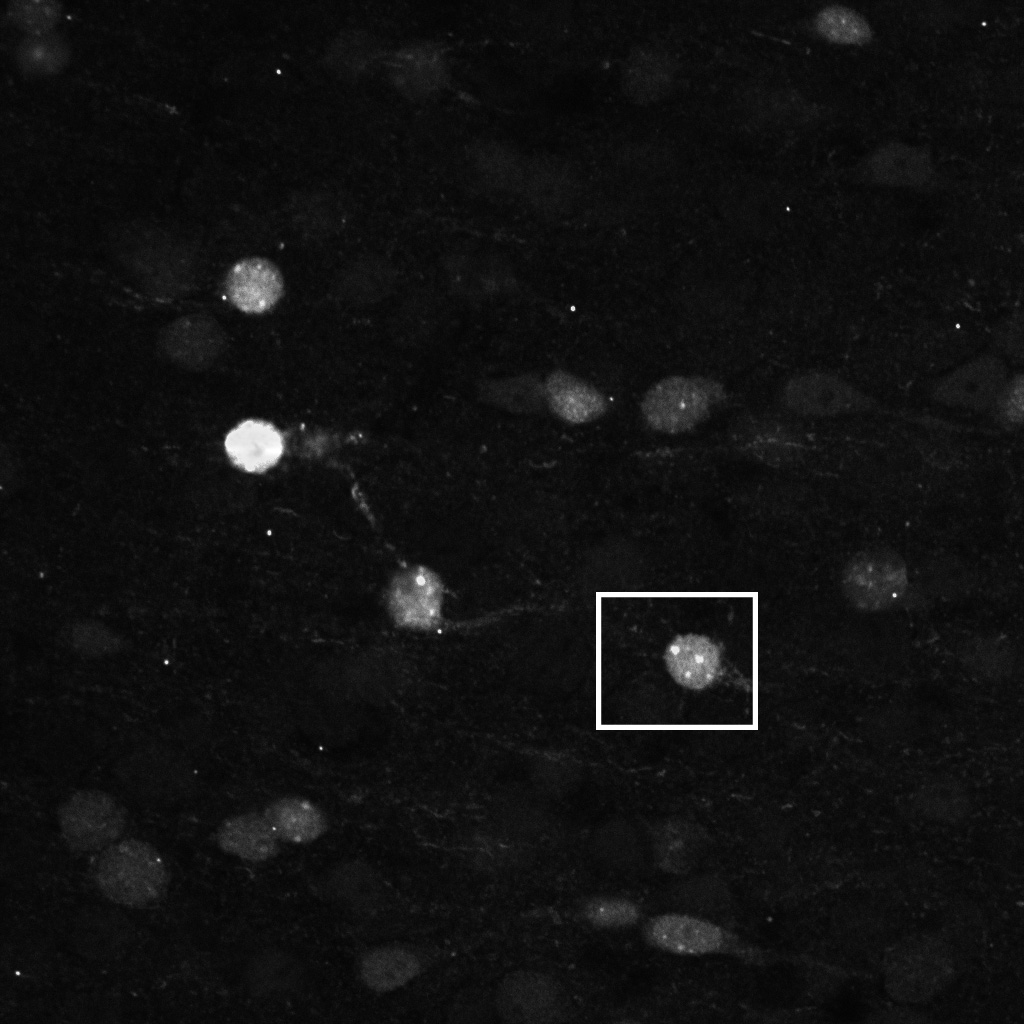

Supplement: Supplementary file 9 — Source data Fig. 1H [file 44318_2025_624_MOESM9_ESM.zip › 1H/n4/CFSE_Z stacks/8_1_C0_Z000 (12).jpg]

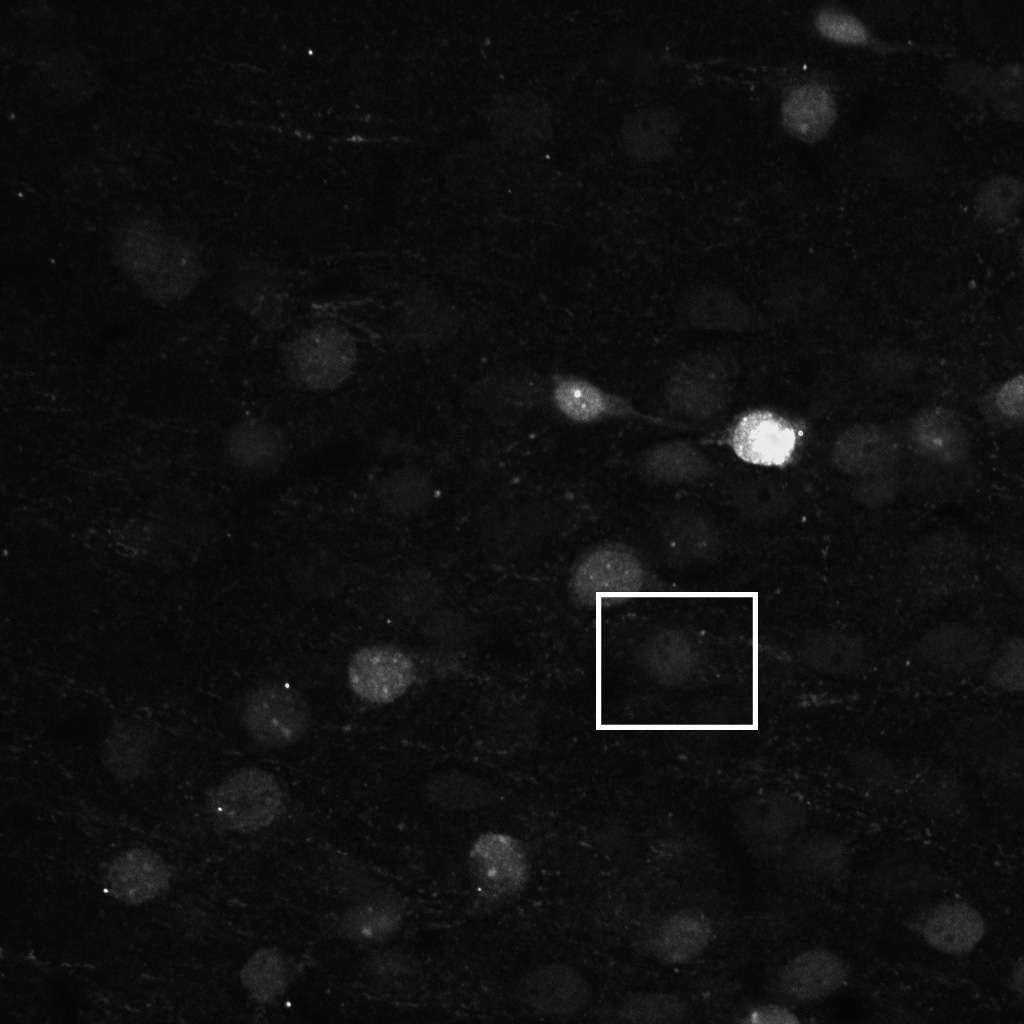

Supplement: Supplementary file 9 — Source data Fig. 1H [file 44318_2025_624_MOESM9_ESM.zip › 1H/n4/CFSE_Z stacks/8_1_C0_Z000 (28).jpg]

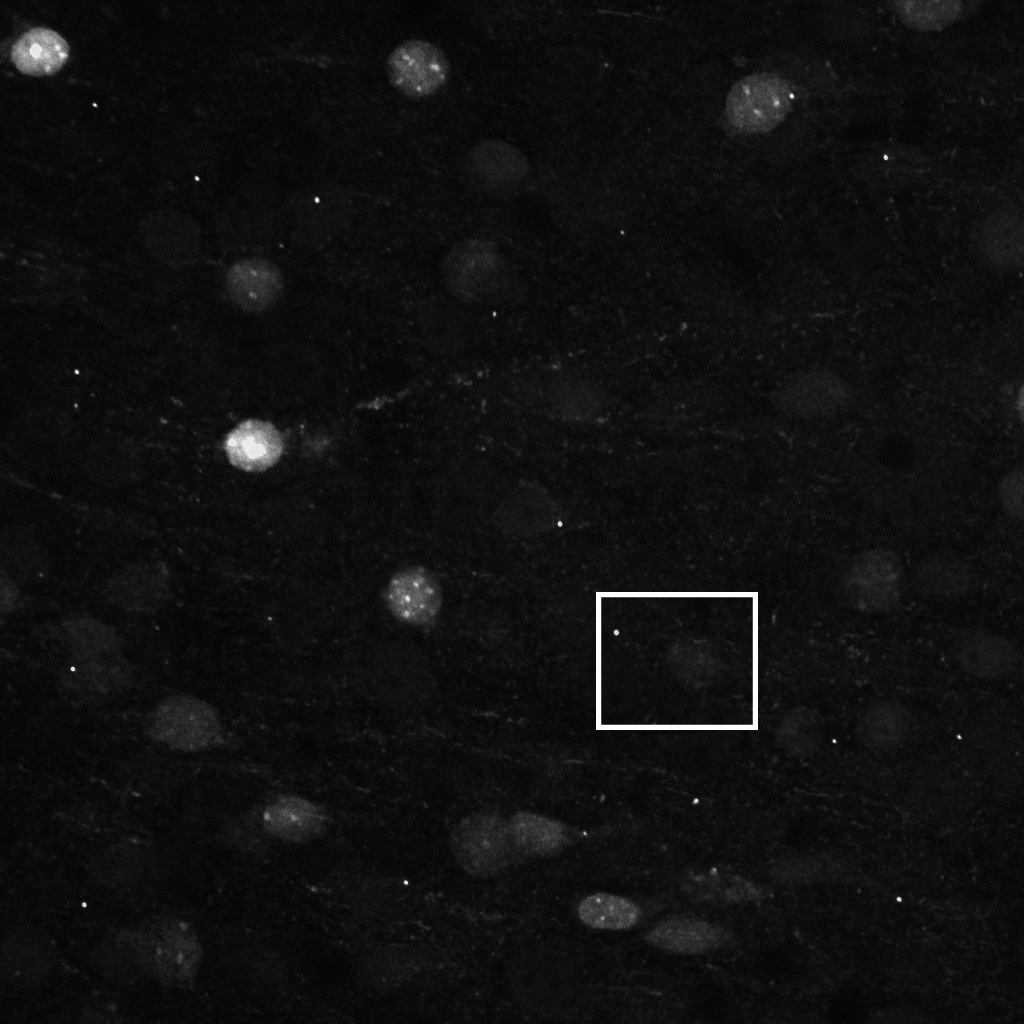

Supplement: Supplementary file 9 — Source data Fig. 1H [file 44318_2025_624_MOESM9_ESM.zip › 1H/n4/CFSE_Z stacks/8_1_C0_Z000 (3).jpg]

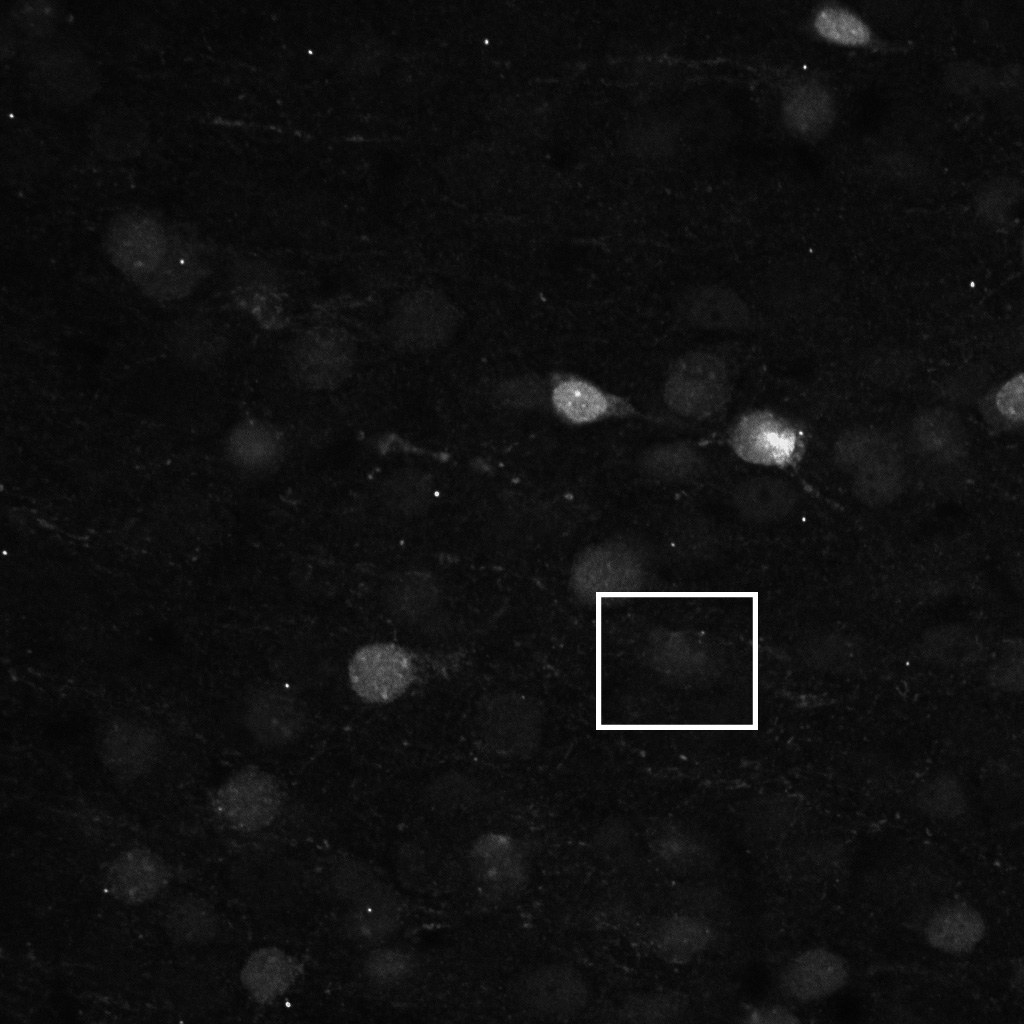

Supplement: Supplementary file 9 — Source data Fig. 1H [file 44318_2025_624_MOESM9_ESM.zip › 1H/n4/CFSE_Z stacks/8_1_C0_Z000 (24).jpg]

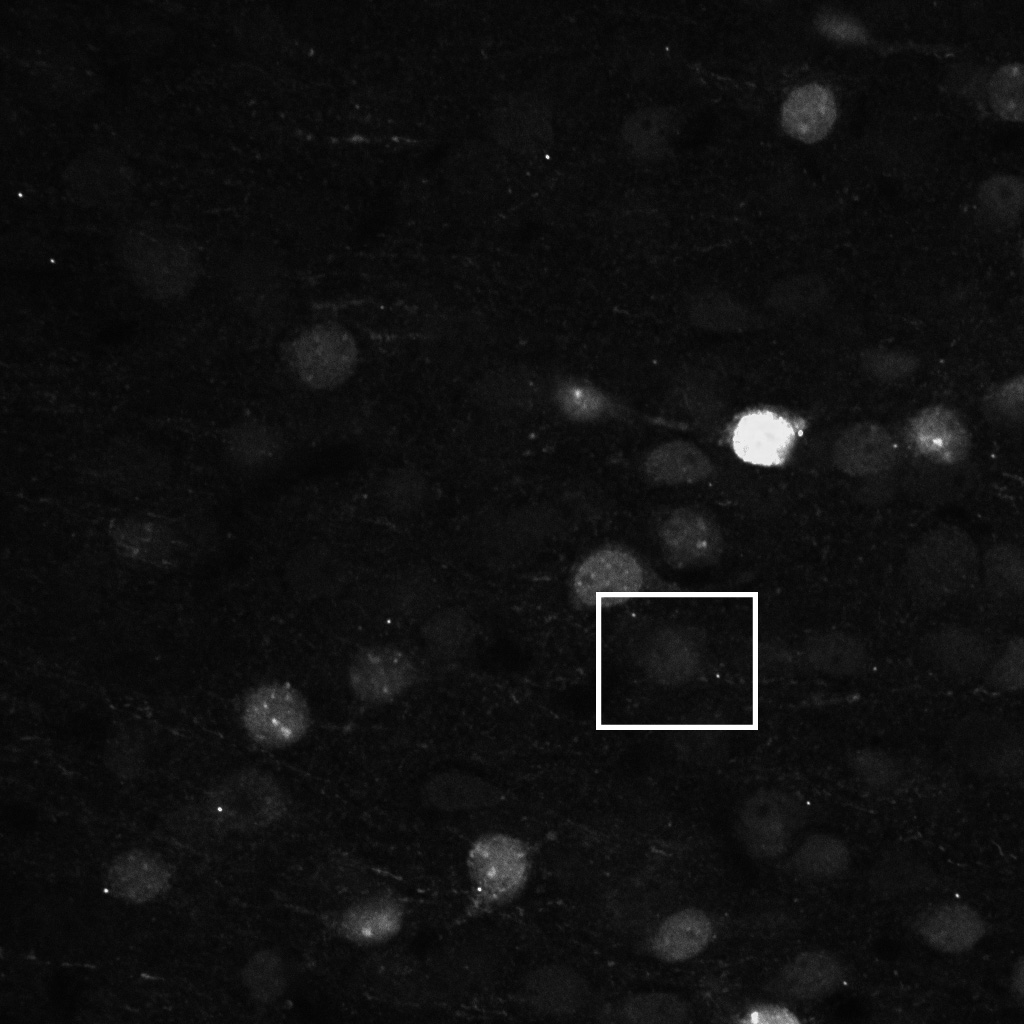

Supplement: Supplementary file 9 — Source data Fig. 1H [file 44318_2025_624_MOESM9_ESM.zip › 1H/n4/CFSE_Z stacks/8_1_C0_Z000 (32).jpg]

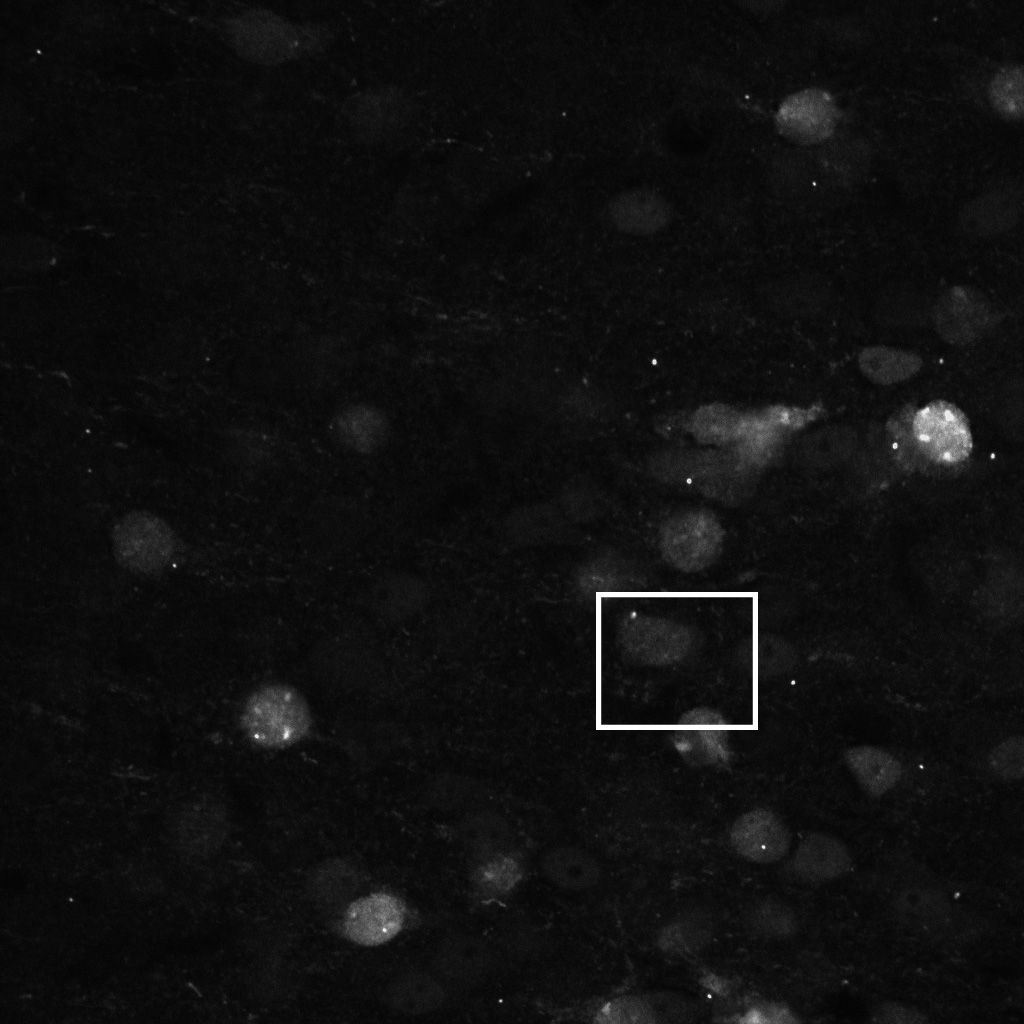

Supplement: Supplementary file 9 — Source data Fig. 1H [file 44318_2025_624_MOESM9_ESM.zip › 1H/n4/CFSE_Z stacks/8_1_C0_Z000 (39).jpg]

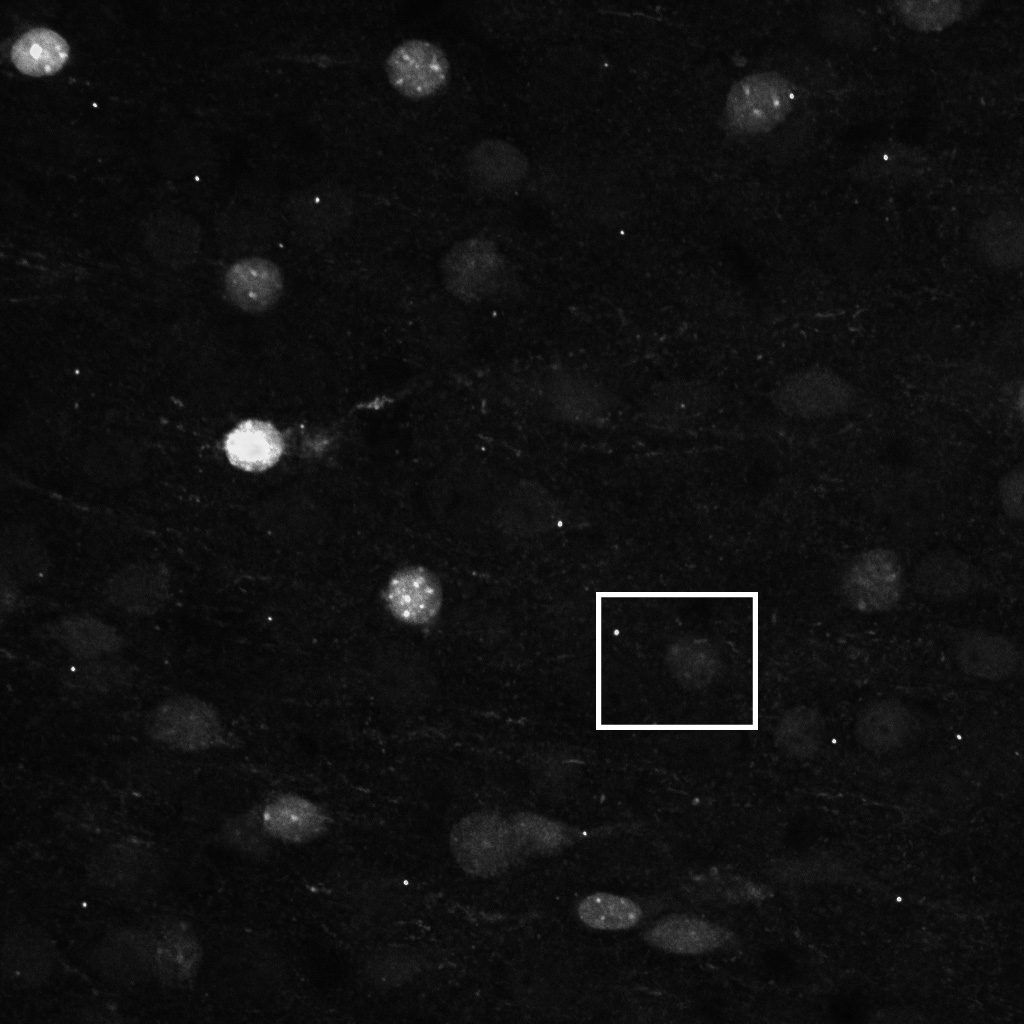

Supplement: Supplementary file 9 — Source data Fig. 1H [file 44318_2025_624_MOESM9_ESM.zip › 1H/n4/CFSE_Z stacks/8_1_C0_Z000 (4).jpg]

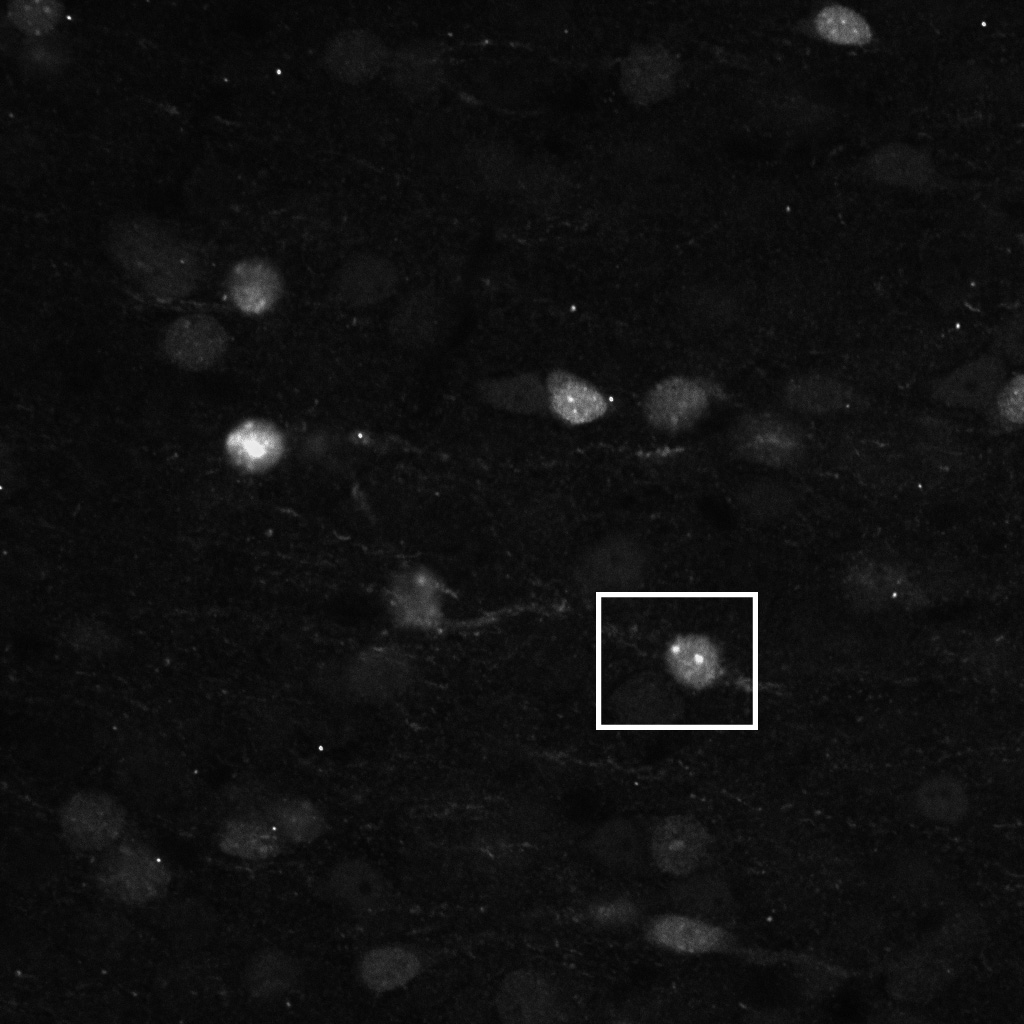

Supplement: Supplementary file 9 — Source data Fig. 1H [file 44318_2025_624_MOESM9_ESM.zip › 1H/n4/CFSE_Z stacks/8_1_C0_Z000 (15).jpg]

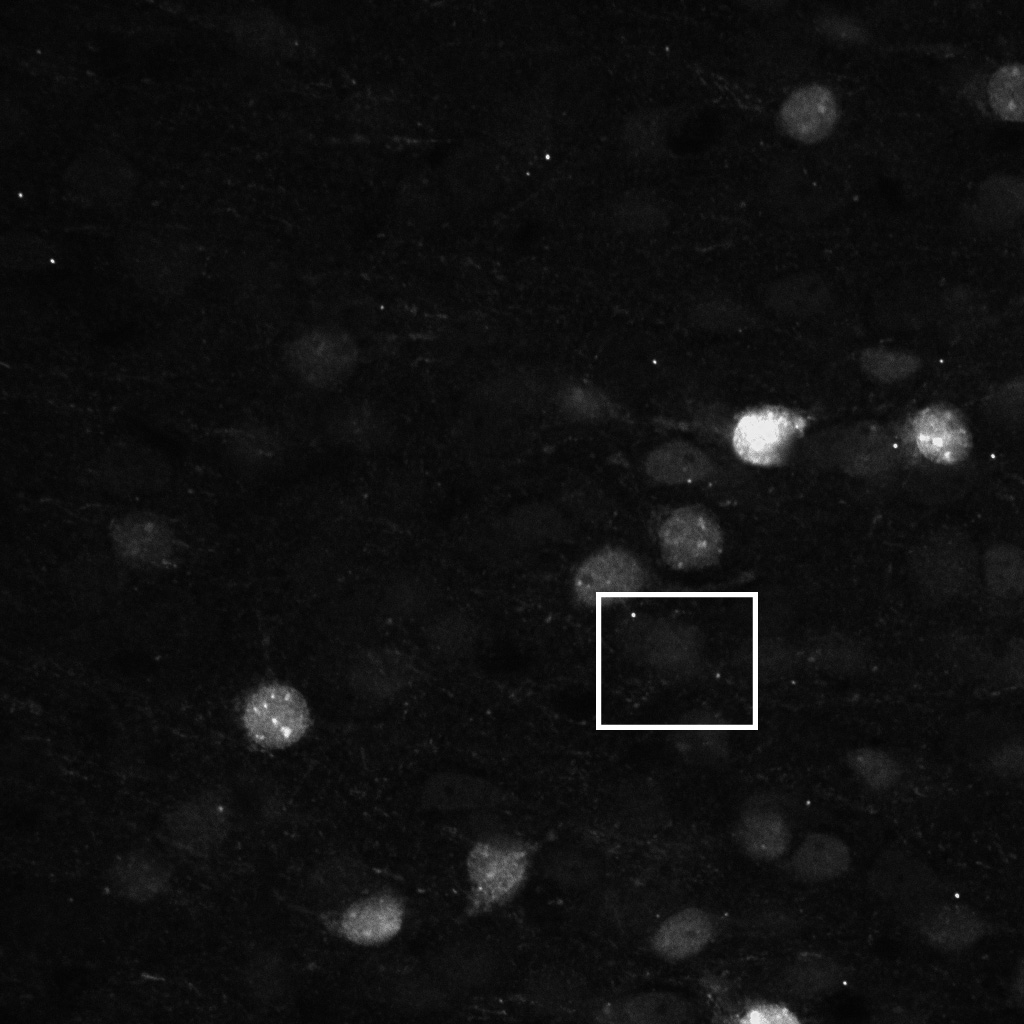

Supplement: Supplementary file 9 — Source data Fig. 1H [file 44318_2025_624_MOESM9_ESM.zip › 1H/n4/CFSE_Z stacks/8_1_C0_Z000 (35).jpg]

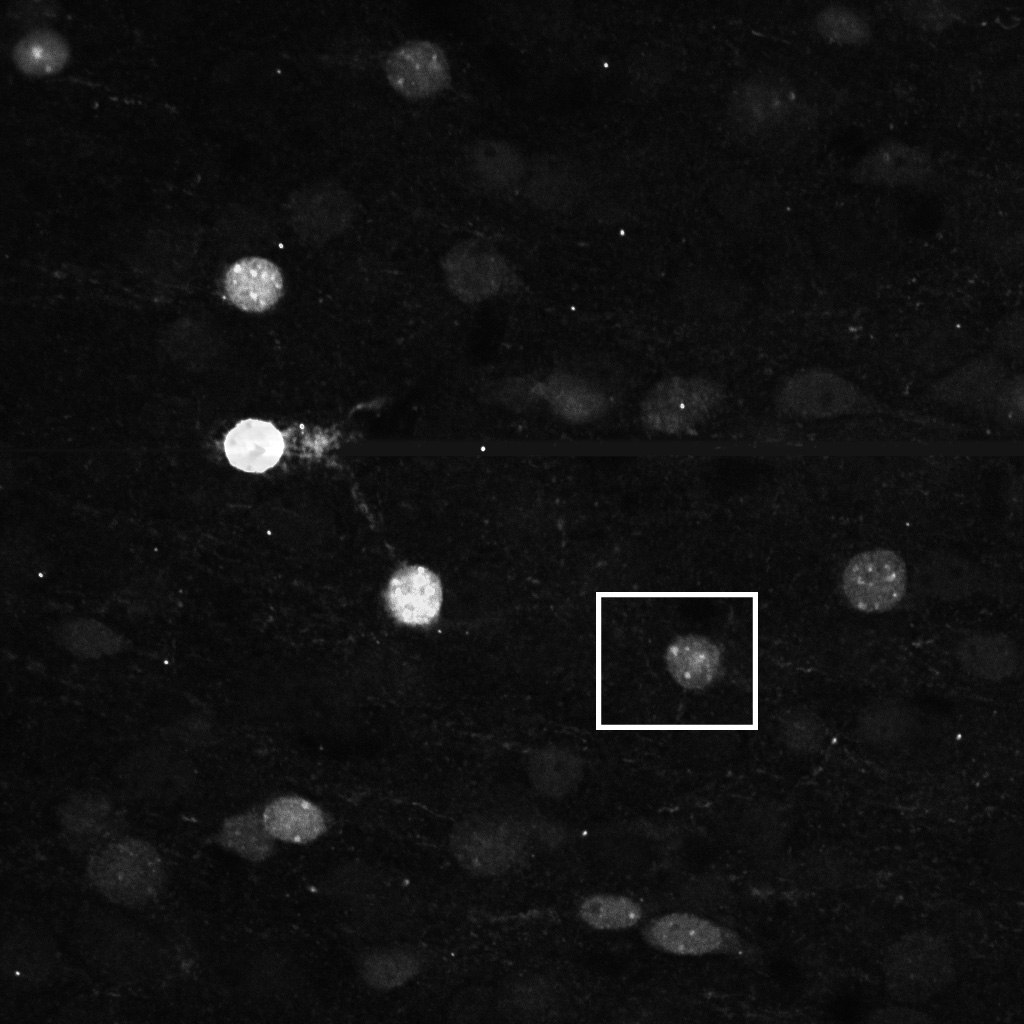

Supplement: Supplementary file 9 — Source data Fig. 1H [file 44318_2025_624_MOESM9_ESM.zip › 1H/n4/CFSE_Z stacks/8_1_C0_Z000 (8).jpg]

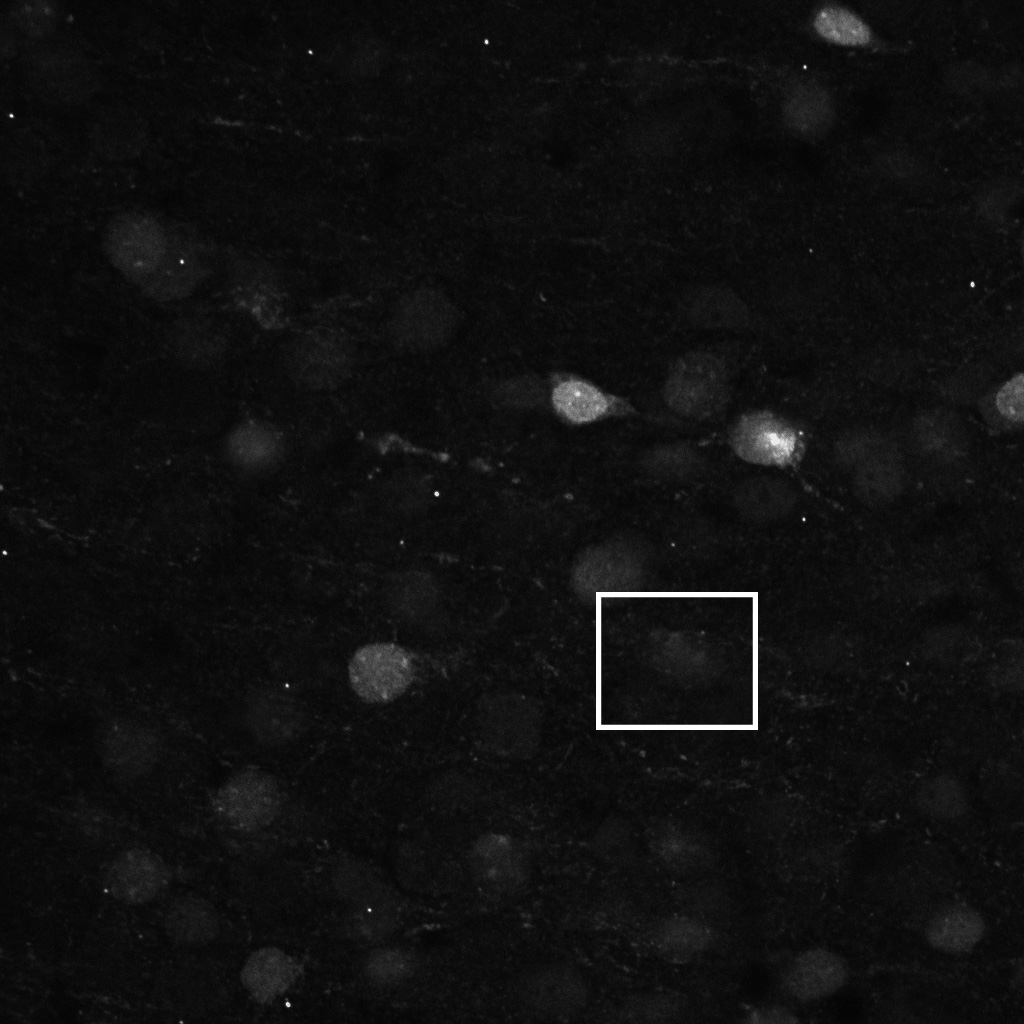

Supplement: Supplementary file 9 — Source data Fig. 1H [file 44318_2025_624_MOESM9_ESM.zip › 1H/n4/CFSE_Z stacks/8_1_C0_Z000 (23).jpg]

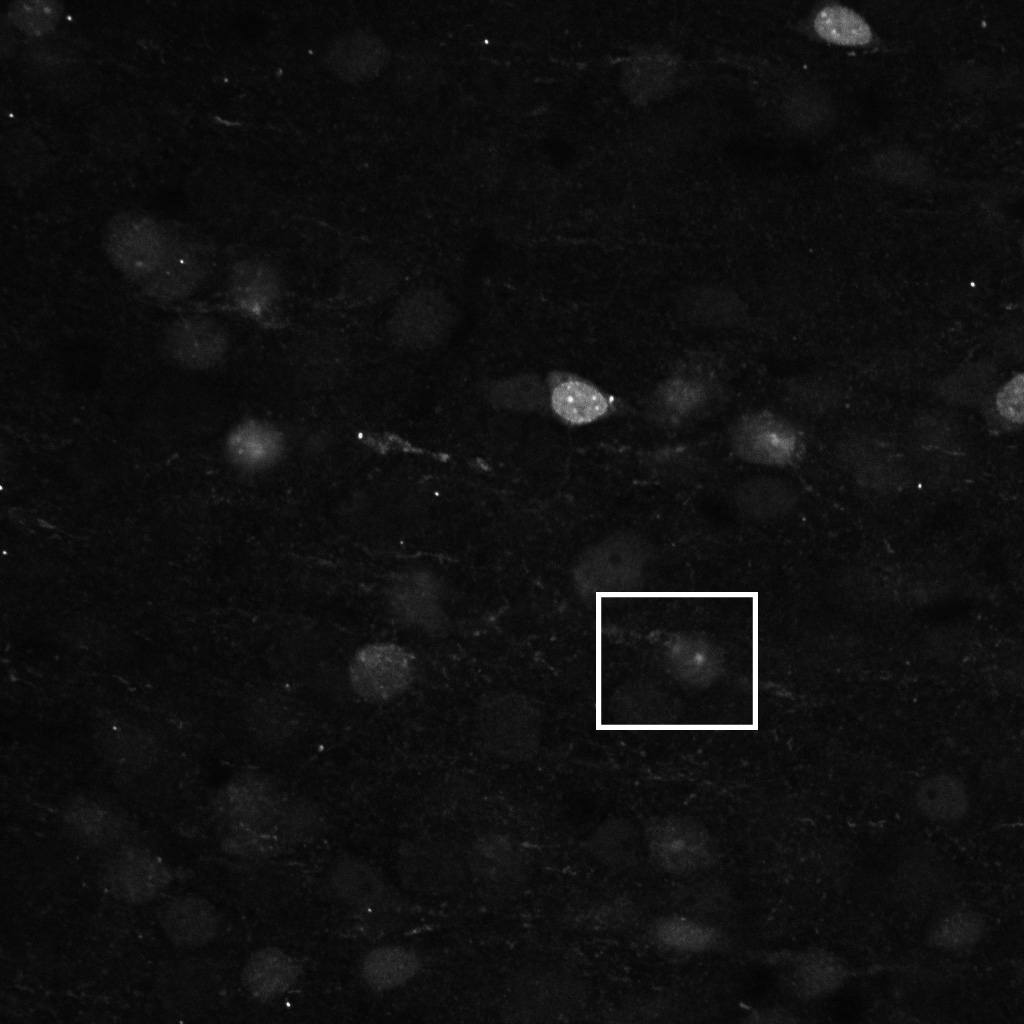

Supplement: Supplementary file 9 — Source data Fig. 1H [file 44318_2025_624_MOESM9_ESM.zip › 1H/n4/CFSE_Z stacks/8_1_C0_Z000 (19).jpg]

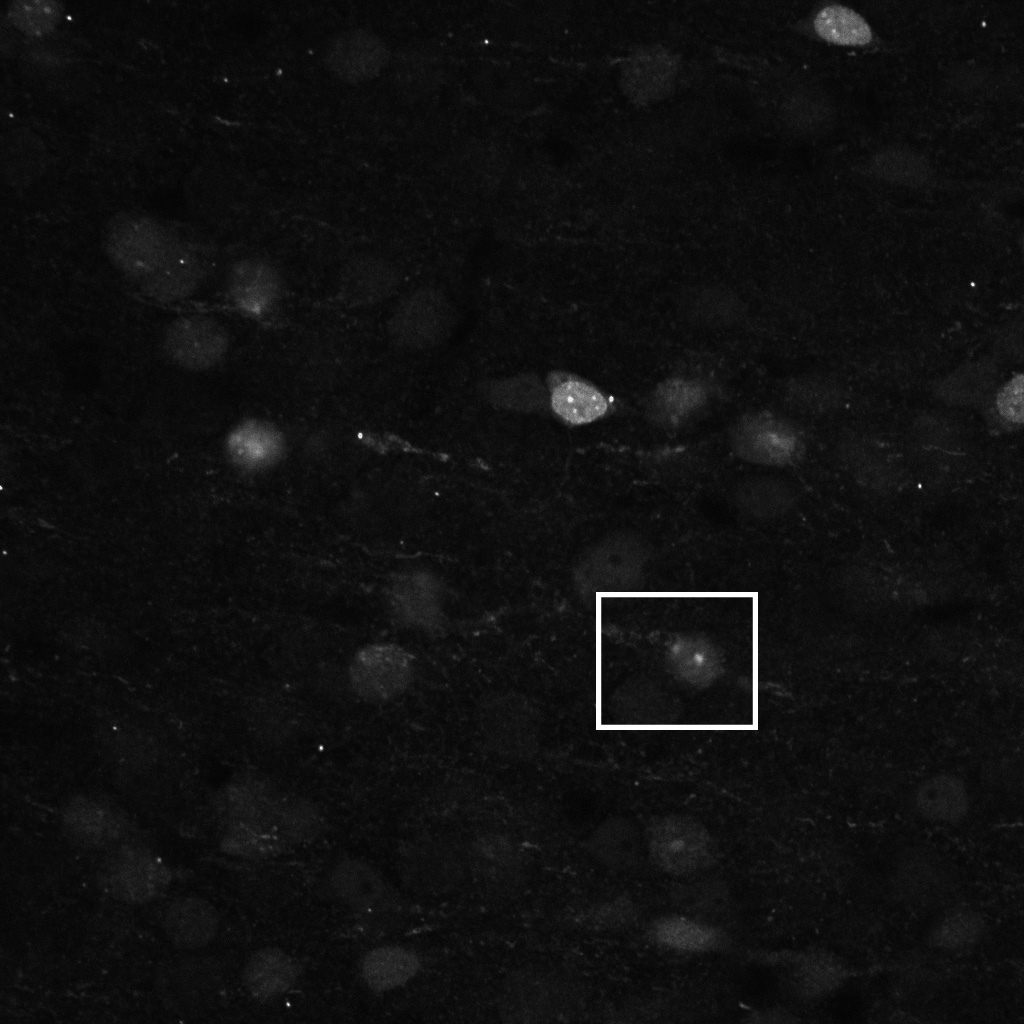

Supplement: Supplementary file 9 — Source data Fig. 1H [file 44318_2025_624_MOESM9_ESM.zip › 1H/n4/CFSE_Z stacks/8_1_C0_Z000 (18).jpg]

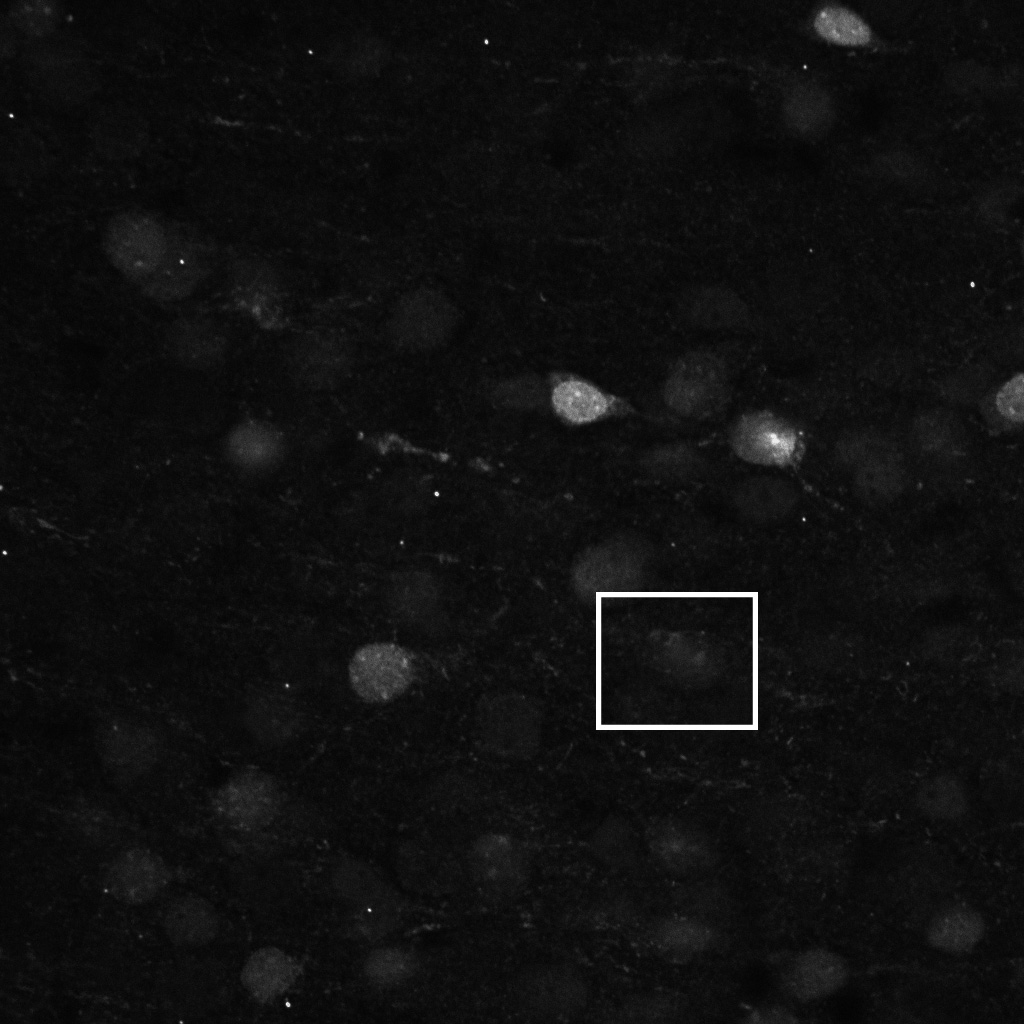

Supplement: Supplementary file 9 — Source data Fig. 1H [file 44318_2025_624_MOESM9_ESM.zip › 1H/n4/CFSE_Z stacks/8_1_C0_Z000 (22).jpg]

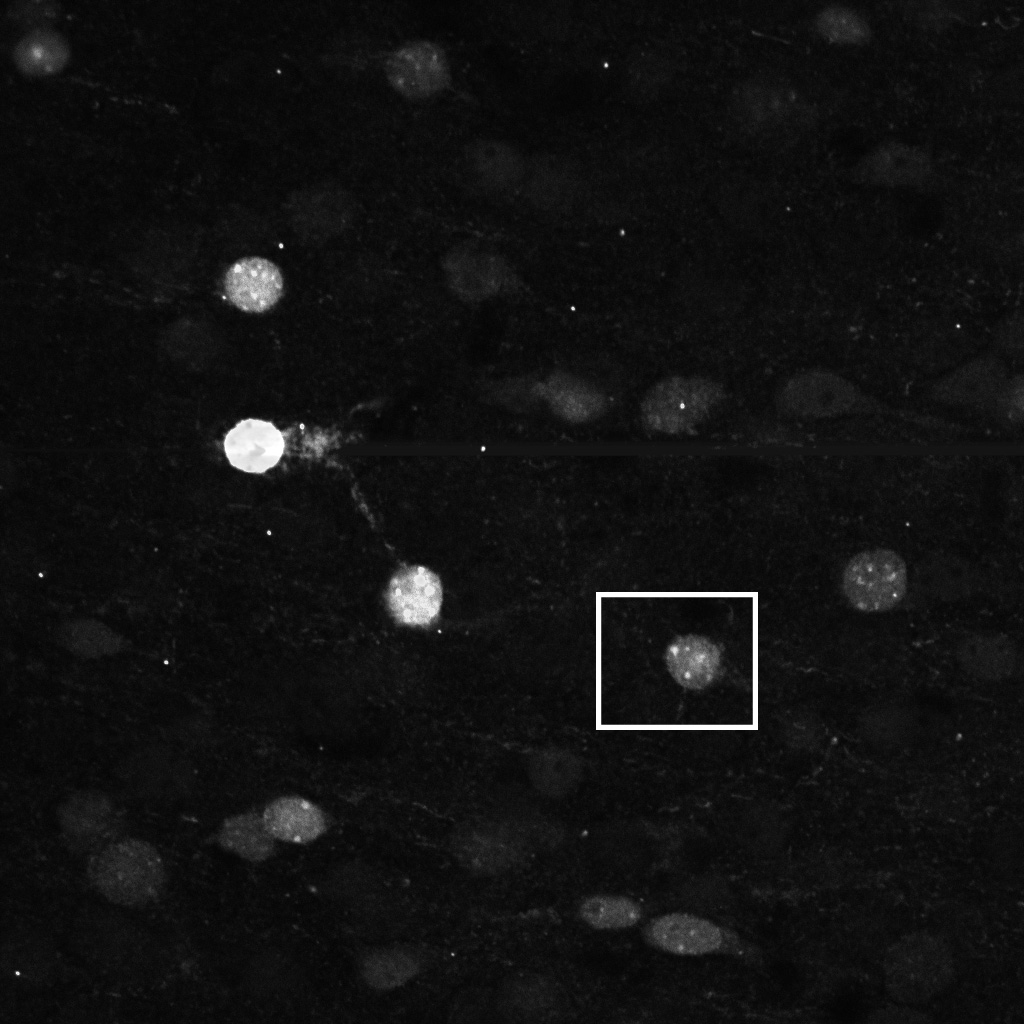

Supplement: Supplementary file 9 — Source data Fig. 1H [file 44318_2025_624_MOESM9_ESM.zip › 1H/n4/CFSE_Z stacks/8_1_C0_Z000 (9).jpg]

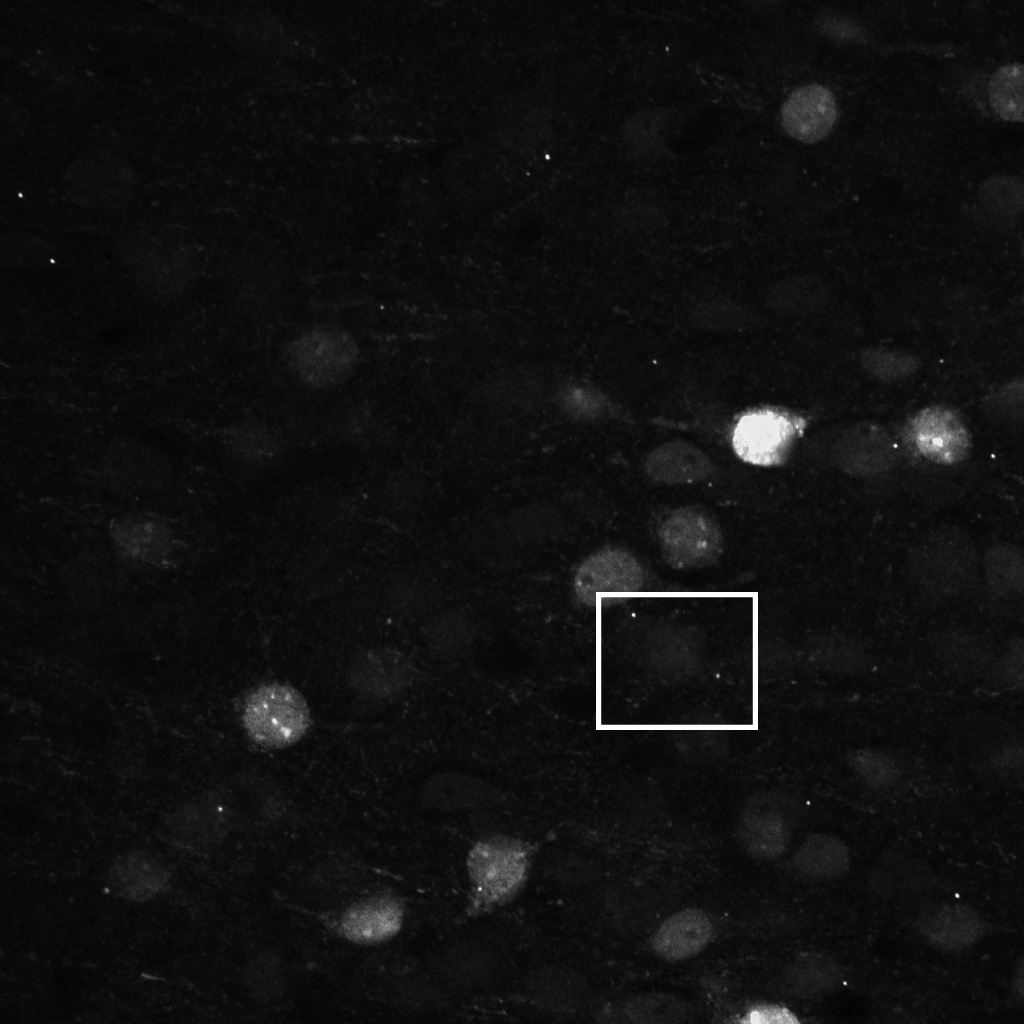

Supplement: Supplementary file 9 — Source data Fig. 1H [file 44318_2025_624_MOESM9_ESM.zip › 1H/n4/CFSE_Z stacks/8_1_C0_Z000 (34).jpg]

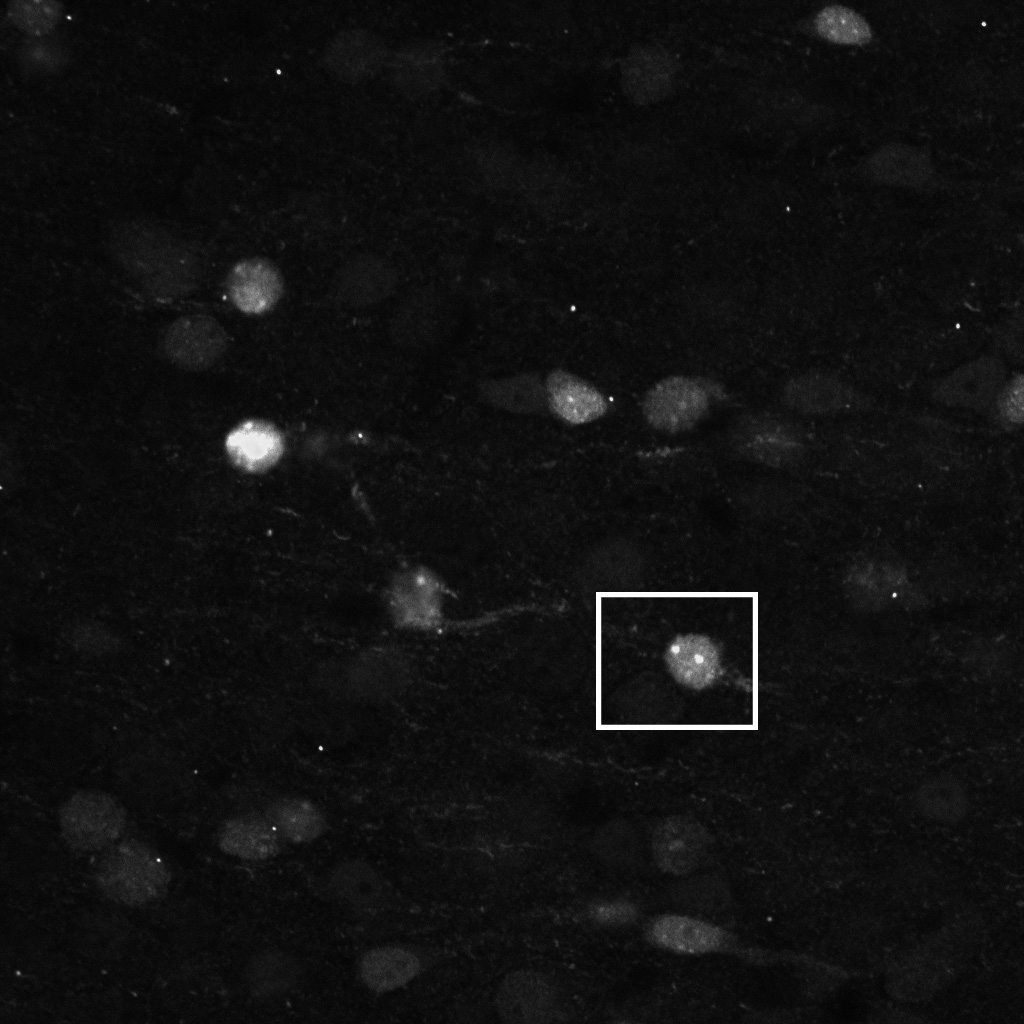

Supplement: Supplementary file 9 — Source data Fig. 1H [file 44318_2025_624_MOESM9_ESM.zip › 1H/n4/CFSE_Z stacks/8_1_C0_Z000 (14).jpg]

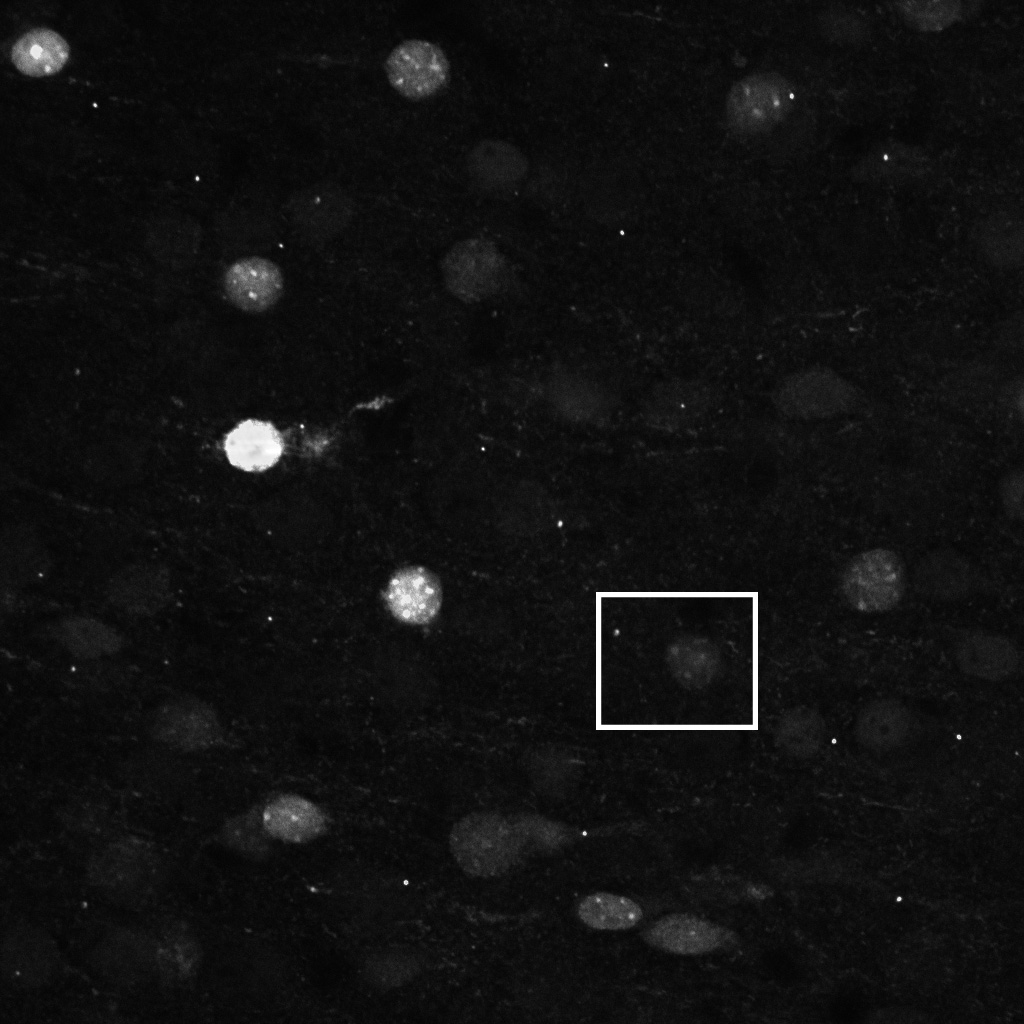

Supplement: Supplementary file 9 — Source data Fig. 1H [file 44318_2025_624_MOESM9_ESM.zip › 1H/n4/CFSE_Z stacks/8_1_C0_Z000 (5).jpg]

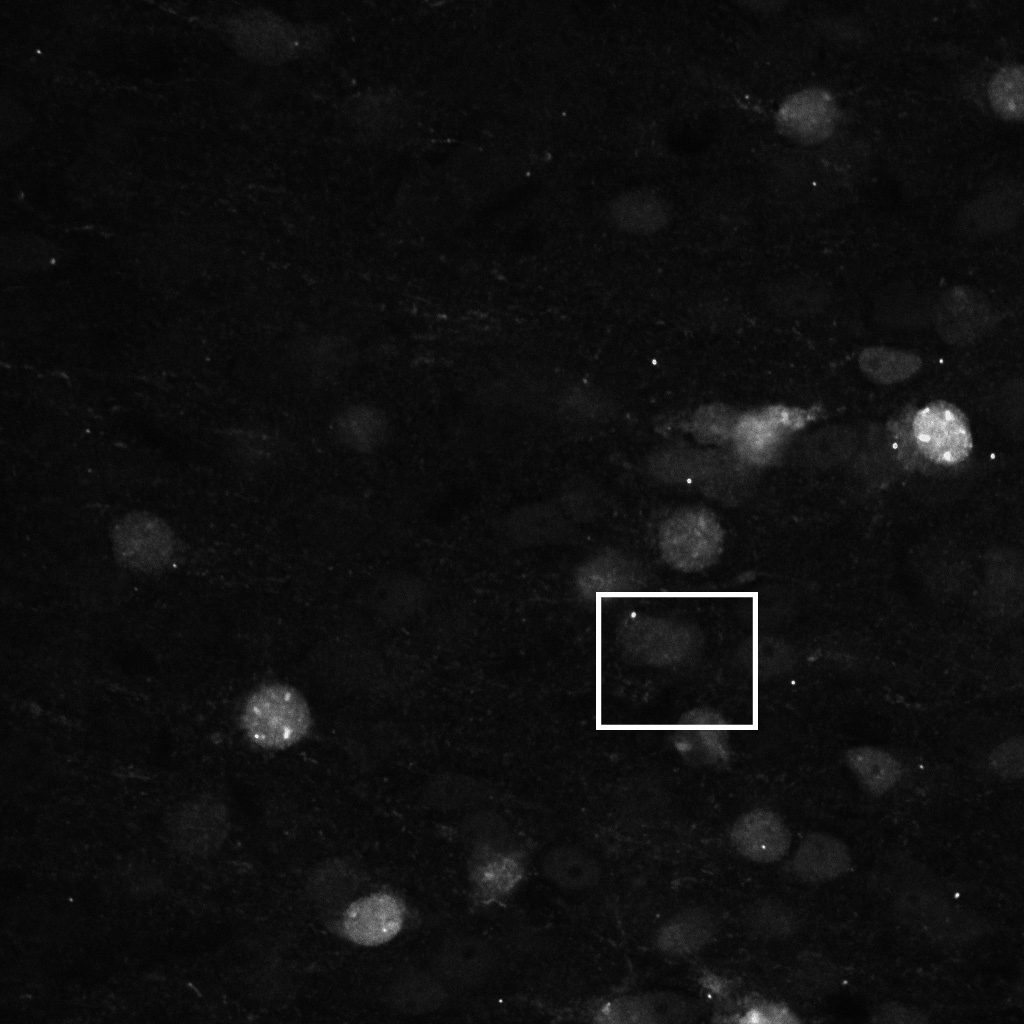

Supplement: Supplementary file 9 — Source data Fig. 1H [file 44318_2025_624_MOESM9_ESM.zip › 1H/n4/CFSE_Z stacks/8_1_C0_Z000 (38).jpg]

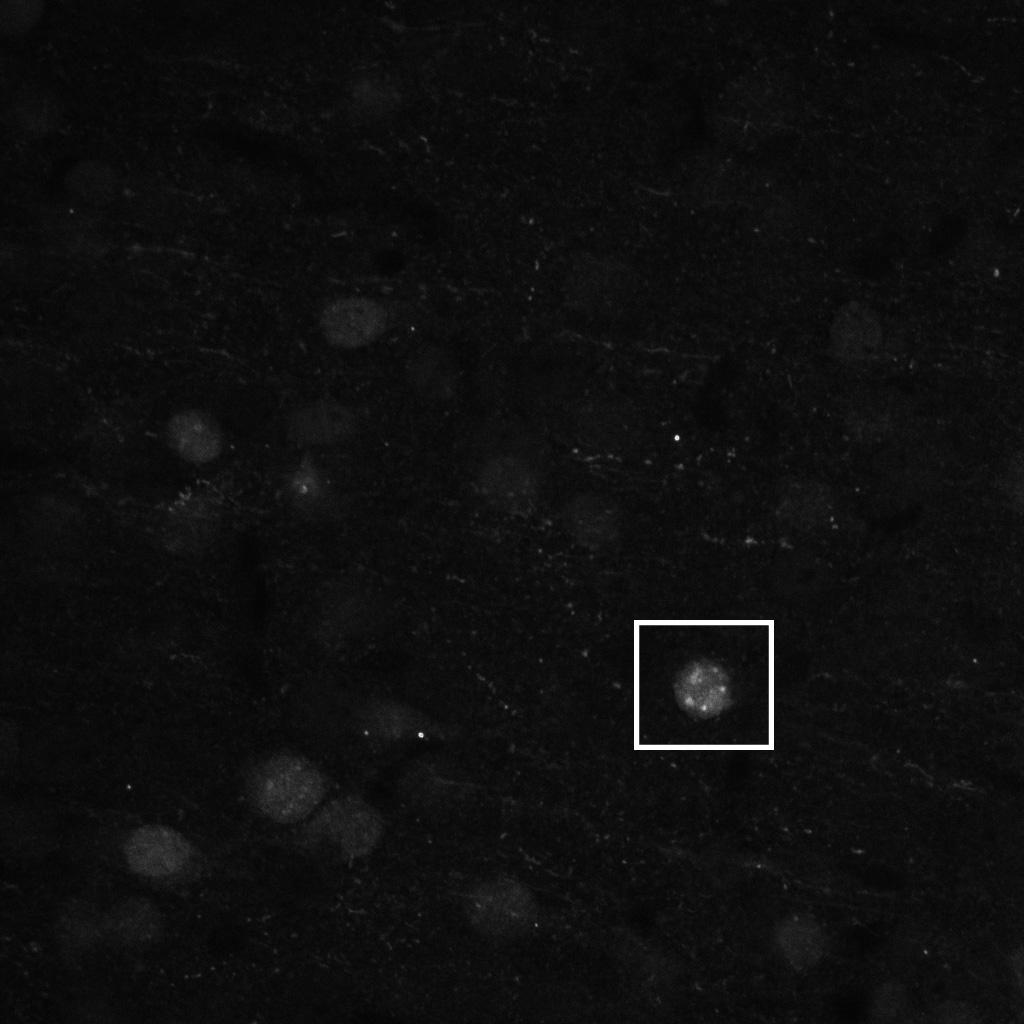

Supplement: Supplementary file 9 — Source data Fig. 1H [file 44318_2025_624_MOESM9_ESM.zip › 1H/n3/CFSE_Z stacks/11_1_C0_Z000 (44).jpg]

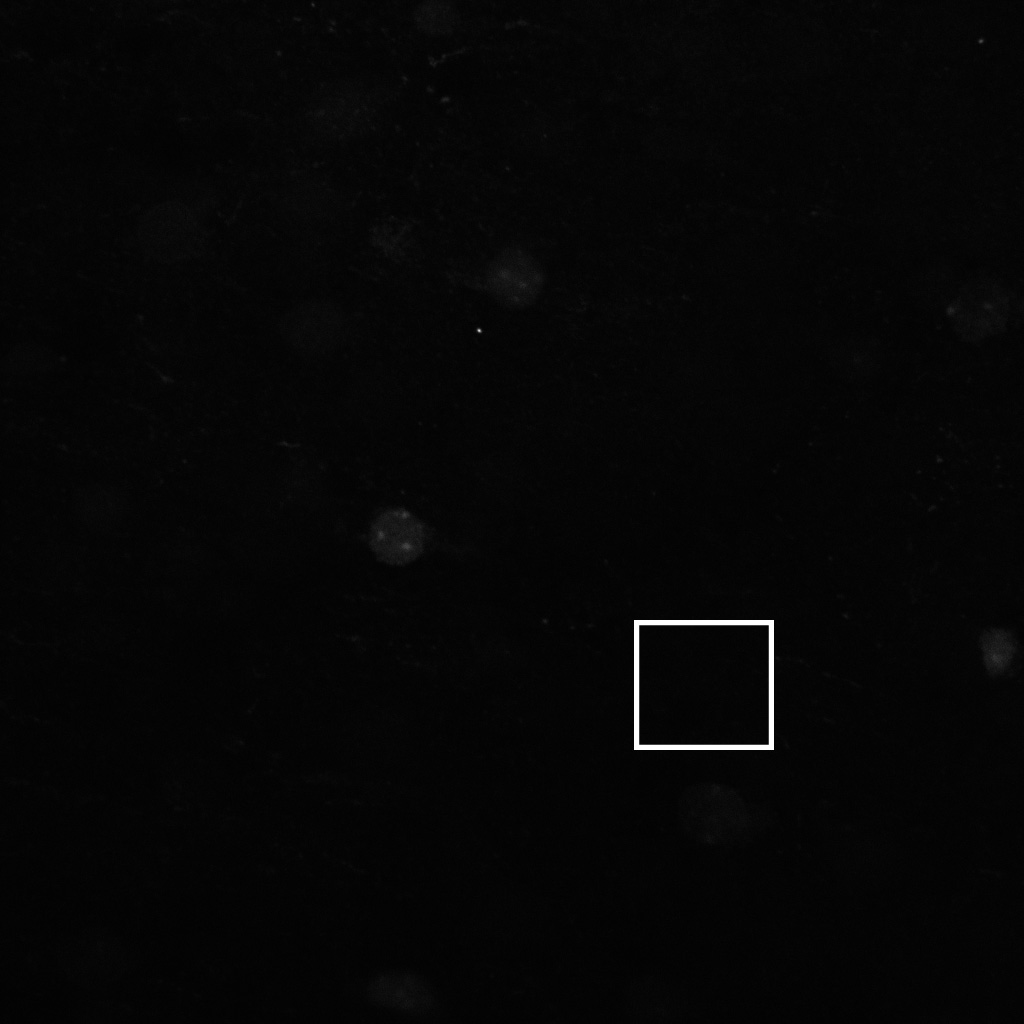

Supplement: Supplementary file 9 — Source data Fig. 1H [file 44318_2025_624_MOESM9_ESM.zip › 1H/n3/CFSE_Z stacks/11_1_C0_Z000 (6).jpg]

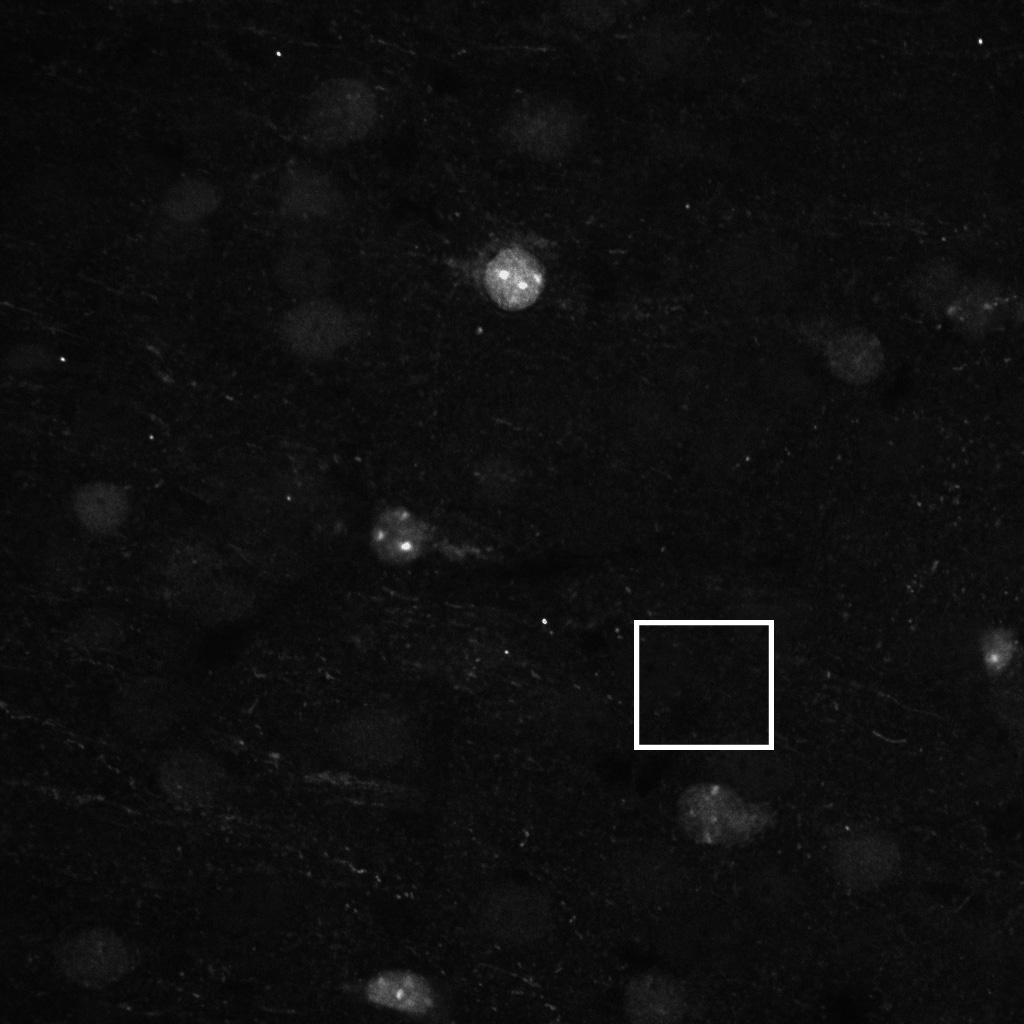

Supplement: Supplementary file 9 — Source data Fig. 1H [file 44318_2025_624_MOESM9_ESM.zip › 1H/n3/CFSE_Z stacks/11_1_C0_Z000 (13).jpg]

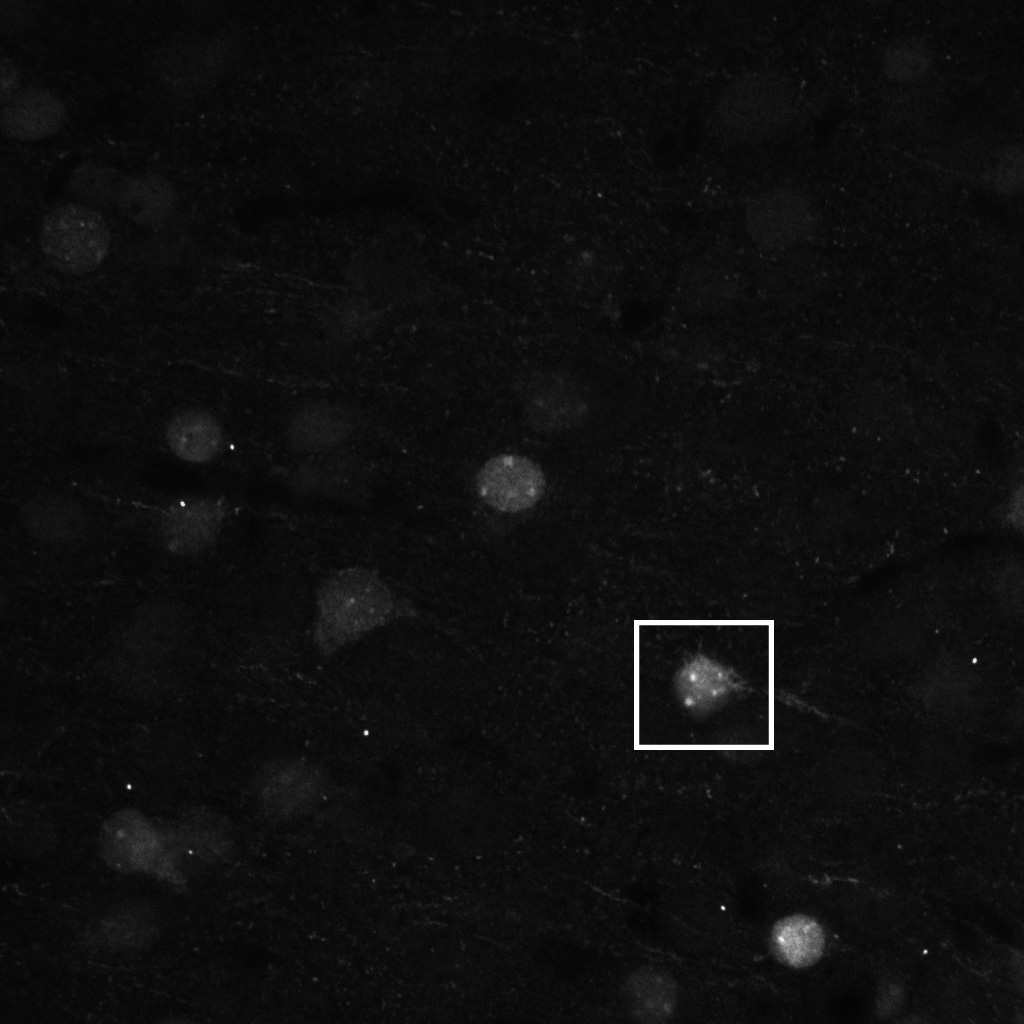

Supplement: Supplementary file 9 — Source data Fig. 1H [file 44318_2025_624_MOESM9_ESM.zip › 1H/n3/CFSE_Z stacks/11_1_C0_Z000 (52).jpg]

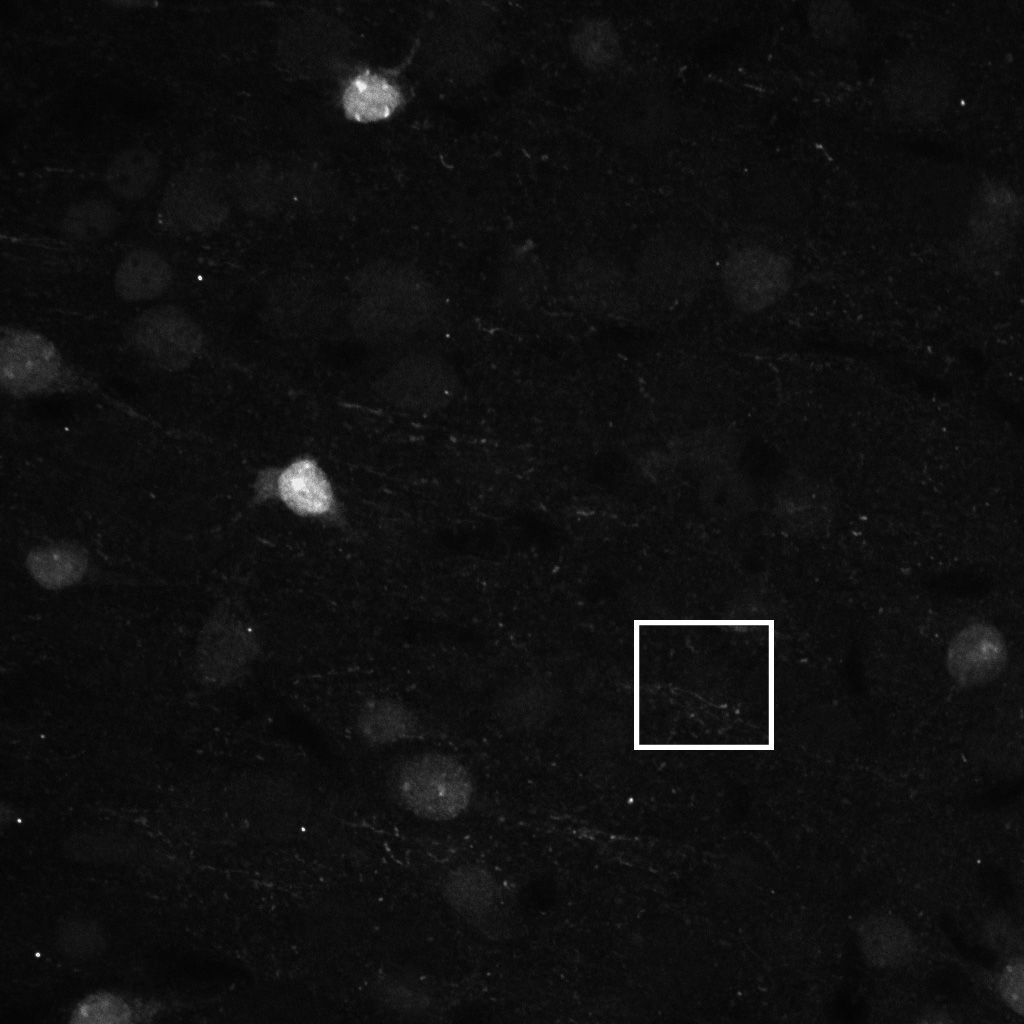

Supplement: Supplementary file 9 — Source data Fig. 1H [file 44318_2025_624_MOESM9_ESM.zip › 1H/n3/CFSE_Z stacks/11_1_C0_Z000 (29).jpg]

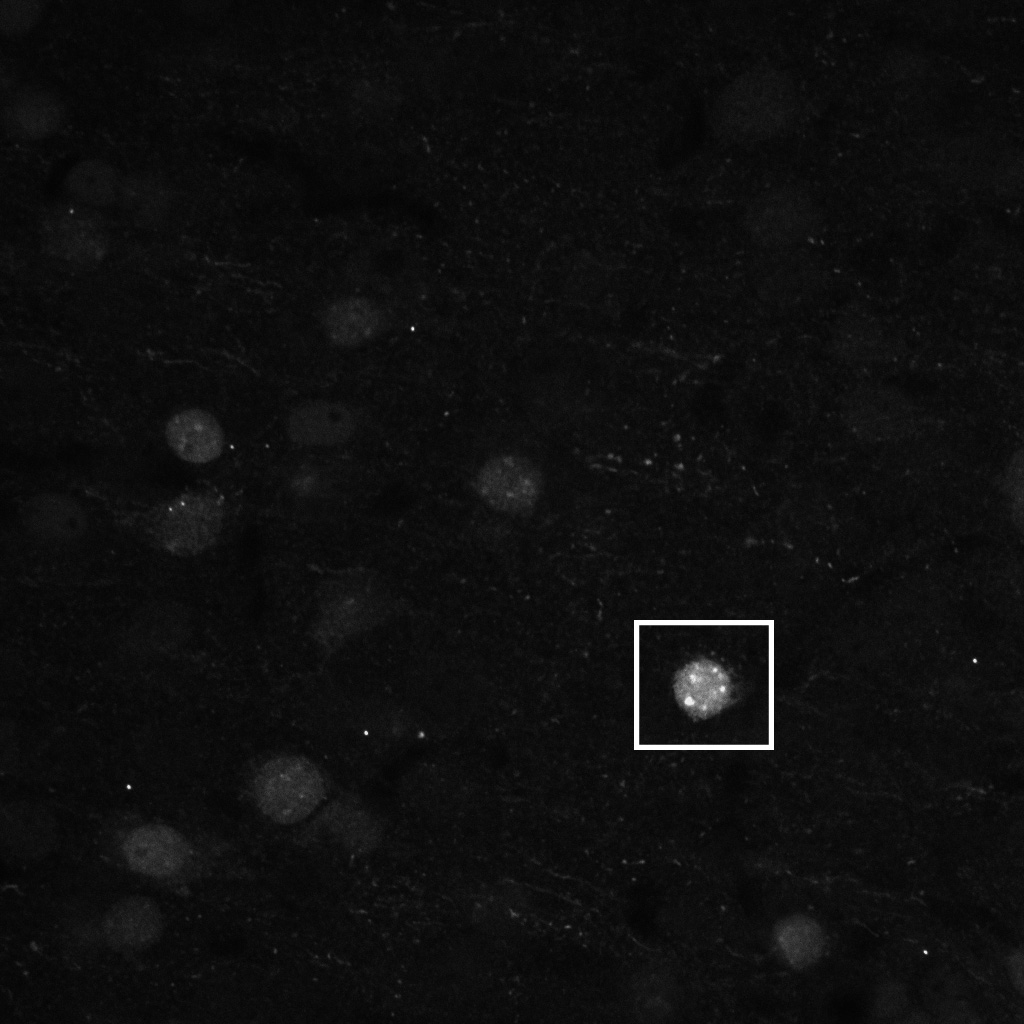

Supplement: Supplementary file 9 — Source data Fig. 1H [file 44318_2025_624_MOESM9_ESM.zip › 1H/n3/CFSE_Z stacks/11_1_C0_Z000 (48).jpg]

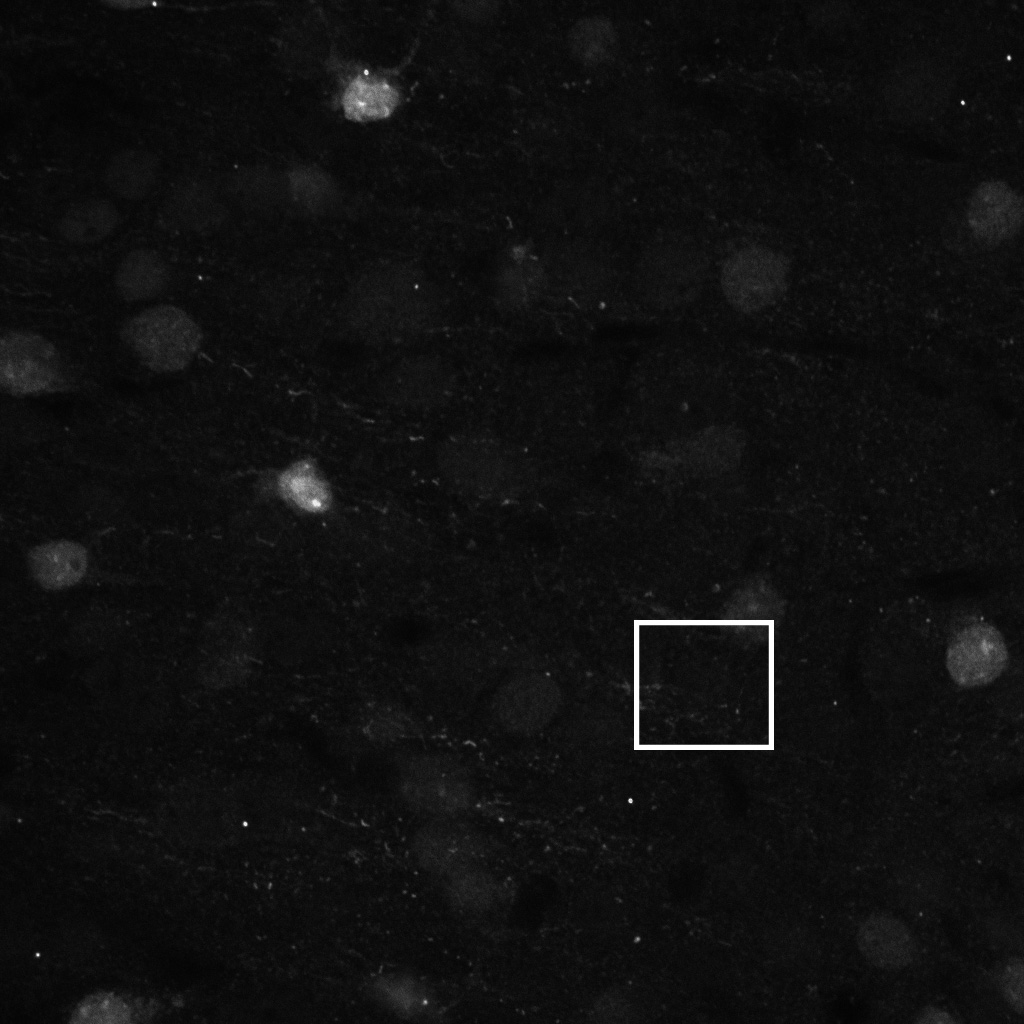

Supplement: Supplementary file 9 — Source data Fig. 1H [file 44318_2025_624_MOESM9_ESM.zip › 1H/n3/CFSE_Z stacks/11_1_C0_Z000 (25).jpg]

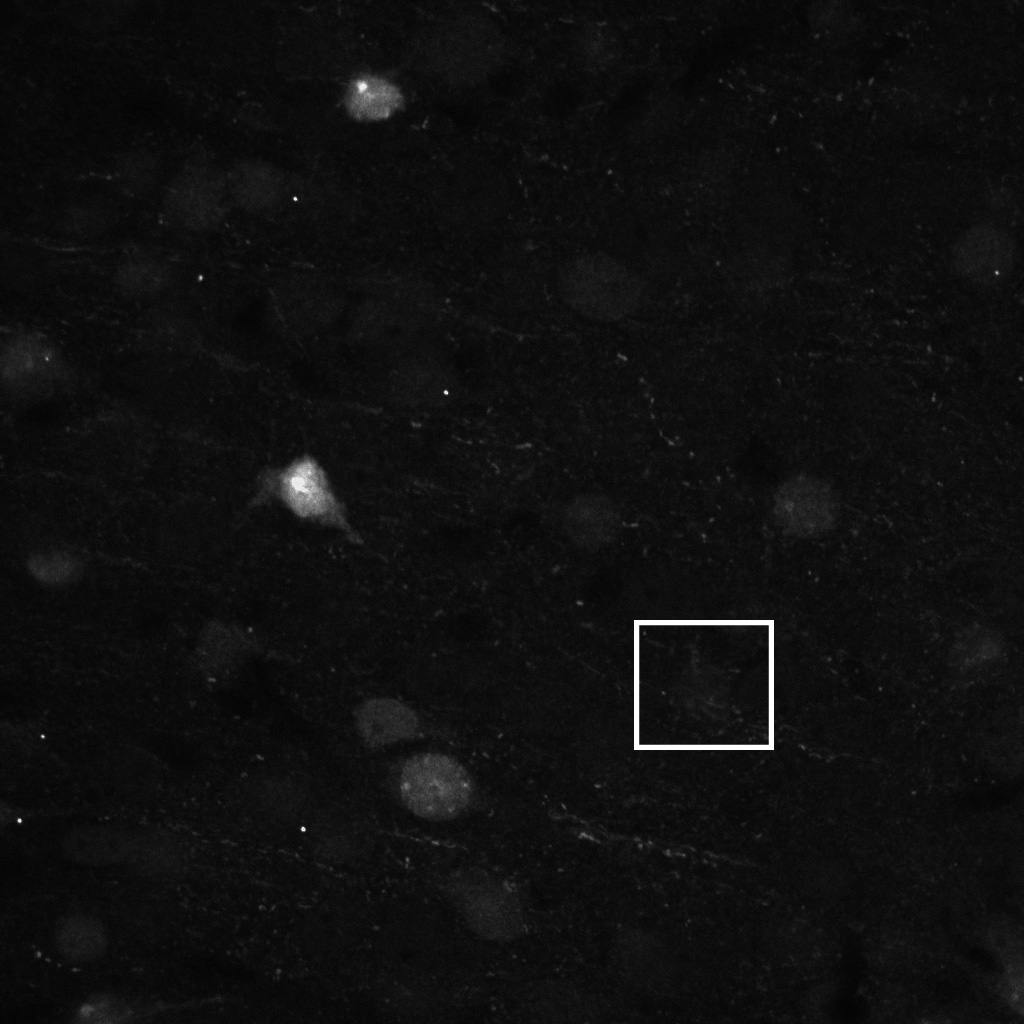

Supplement: Supplementary file 9 — Source data Fig. 1H [file 44318_2025_624_MOESM9_ESM.zip › 1H/n3/CFSE_Z stacks/11_1_C0_Z000 (33).jpg]

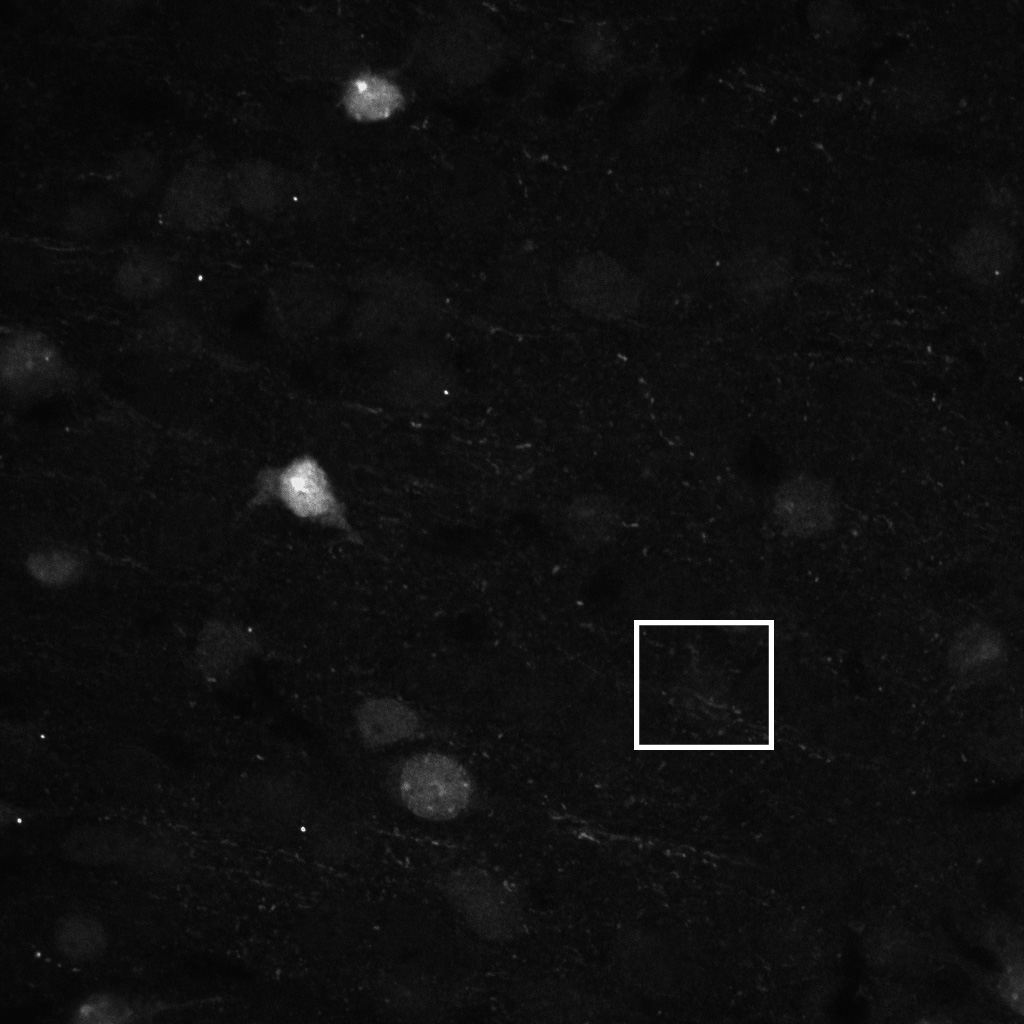

Supplement: Supplementary file 9 — Source data Fig. 1H [file 44318_2025_624_MOESM9_ESM.zip › 1H/n3/CFSE_Z stacks/11_1_C0_Z000 (32).jpg]

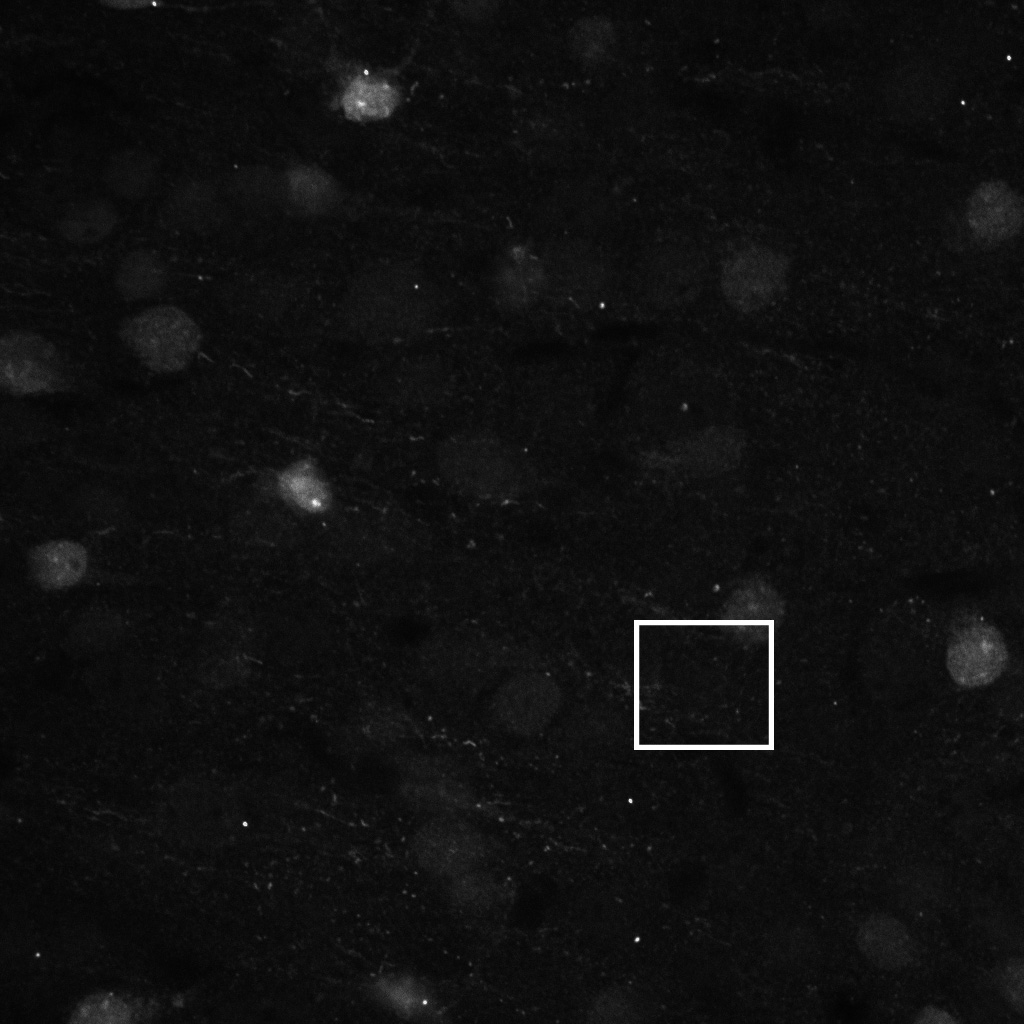

Supplement: Supplementary file 9 — Source data Fig. 1H [file 44318_2025_624_MOESM9_ESM.zip › 1H/n3/CFSE_Z stacks/11_1_C0_Z000 (24).jpg]

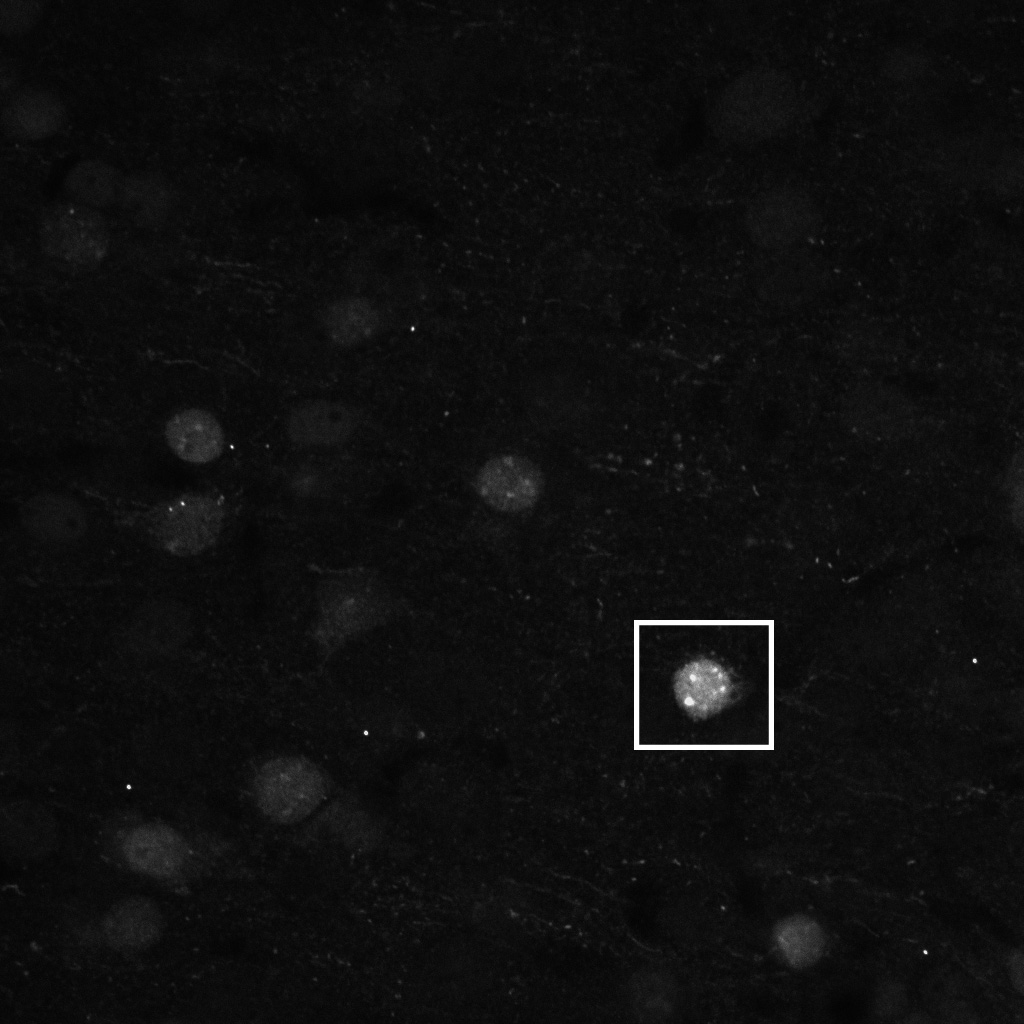

Supplement: Supplementary file 9 — Source data Fig. 1H [file 44318_2025_624_MOESM9_ESM.zip › 1H/n3/CFSE_Z stacks/11_1_C0_Z000 (49).jpg]

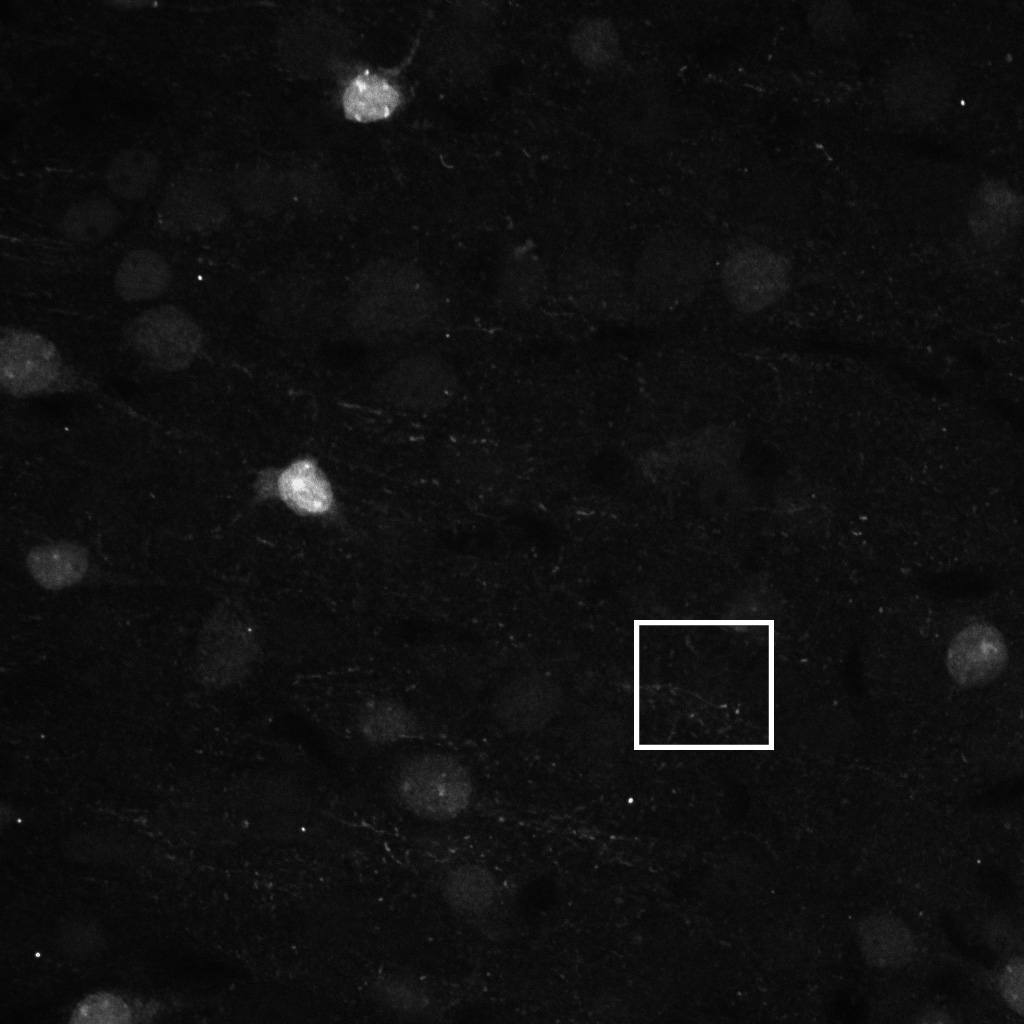

Supplement: Supplementary file 9 — Source data Fig. 1H [file 44318_2025_624_MOESM9_ESM.zip › 1H/n3/CFSE_Z stacks/11_1_C0_Z000 (28).jpg]

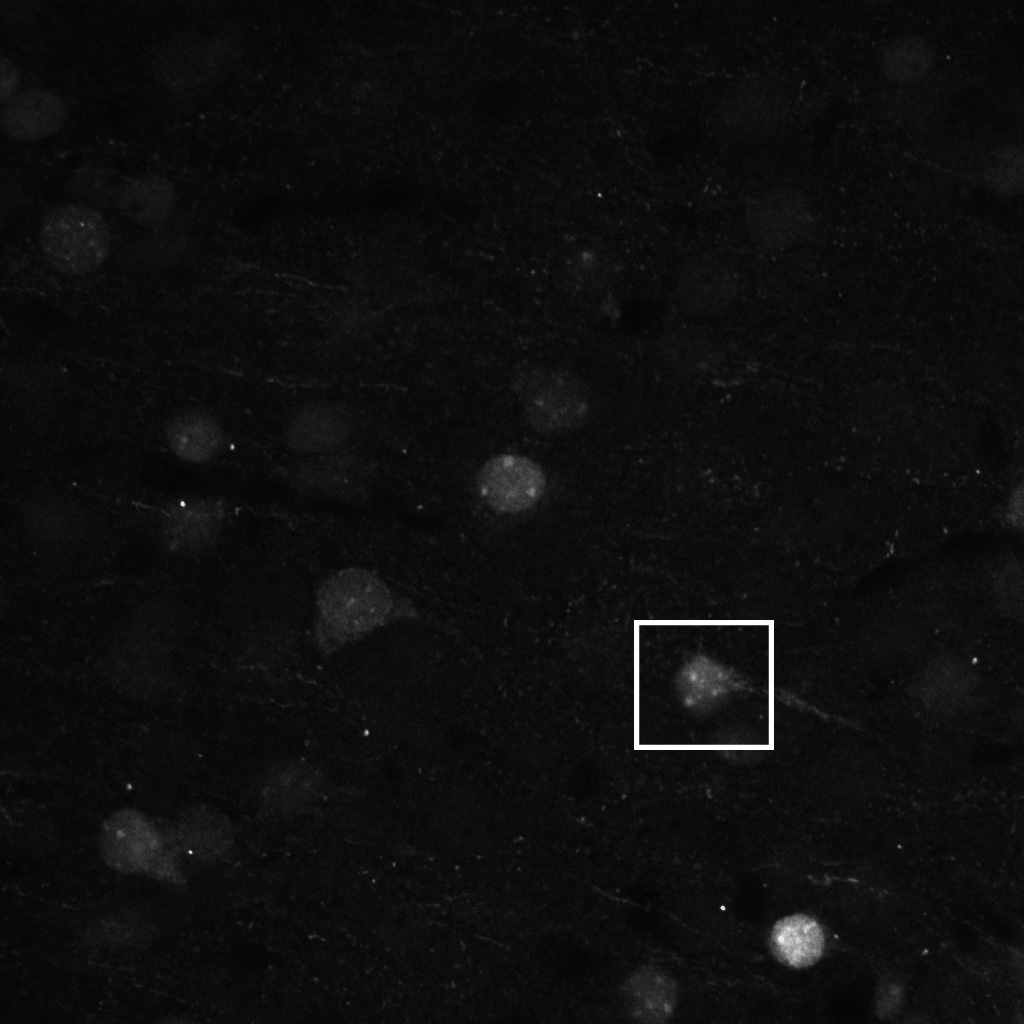

Supplement: Supplementary file 9 — Source data Fig. 1H [file 44318_2025_624_MOESM9_ESM.zip › 1H/n3/CFSE_Z stacks/11_1_C0_Z000 (53).jpg]

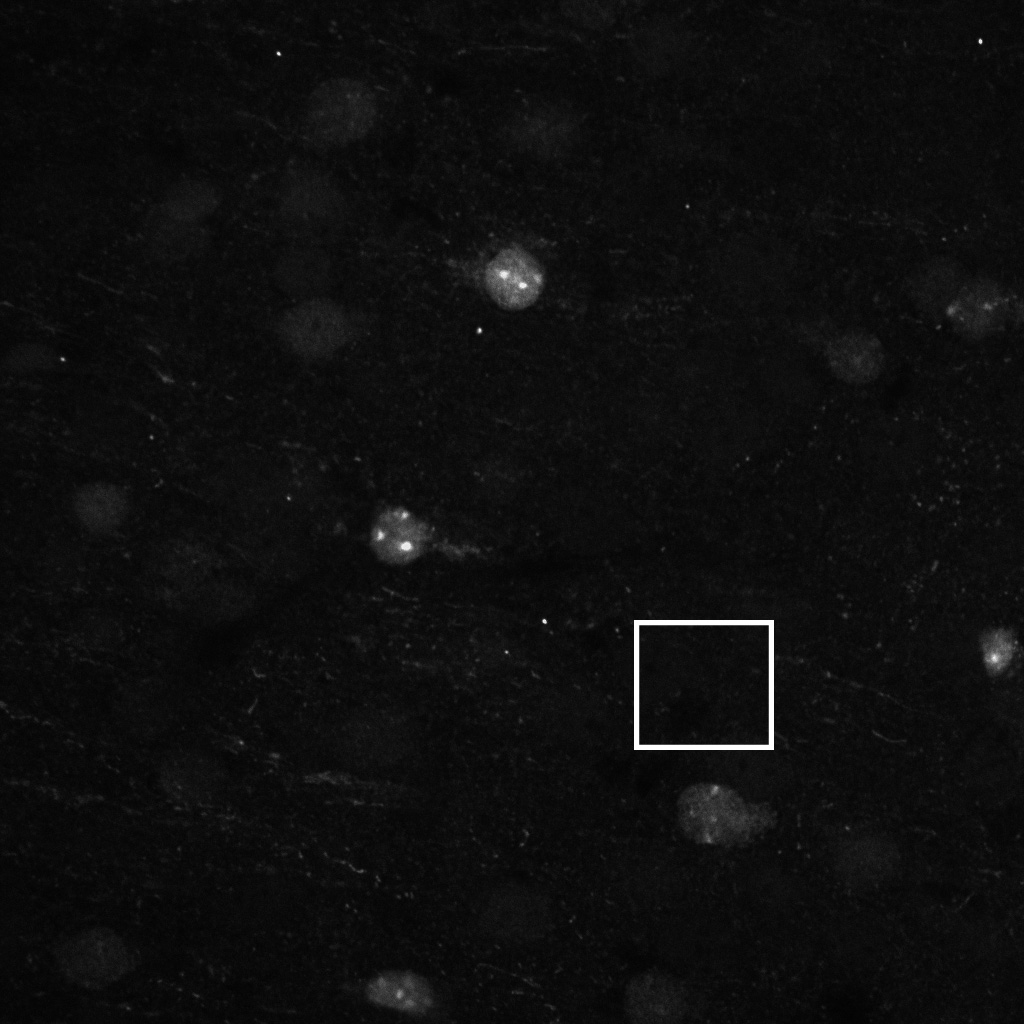

Supplement: Supplementary file 9 — Source data Fig. 1H [file 44318_2025_624_MOESM9_ESM.zip › 1H/n3/CFSE_Z stacks/11_1_C0_Z000 (12).jpg]

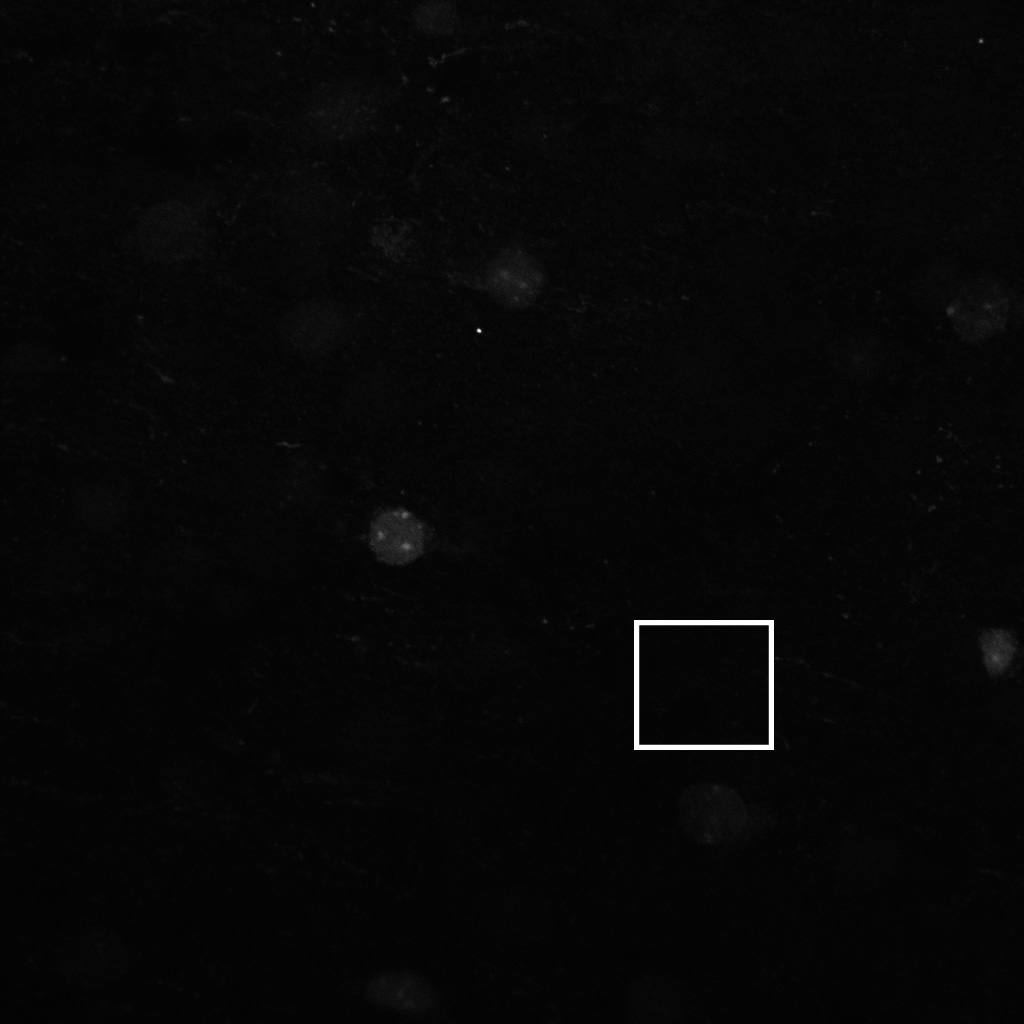

Supplement: Supplementary file 9 — Source data Fig. 1H [file 44318_2025_624_MOESM9_ESM.zip › 1H/n3/CFSE_Z stacks/11_1_C0_Z000 (7).jpg]

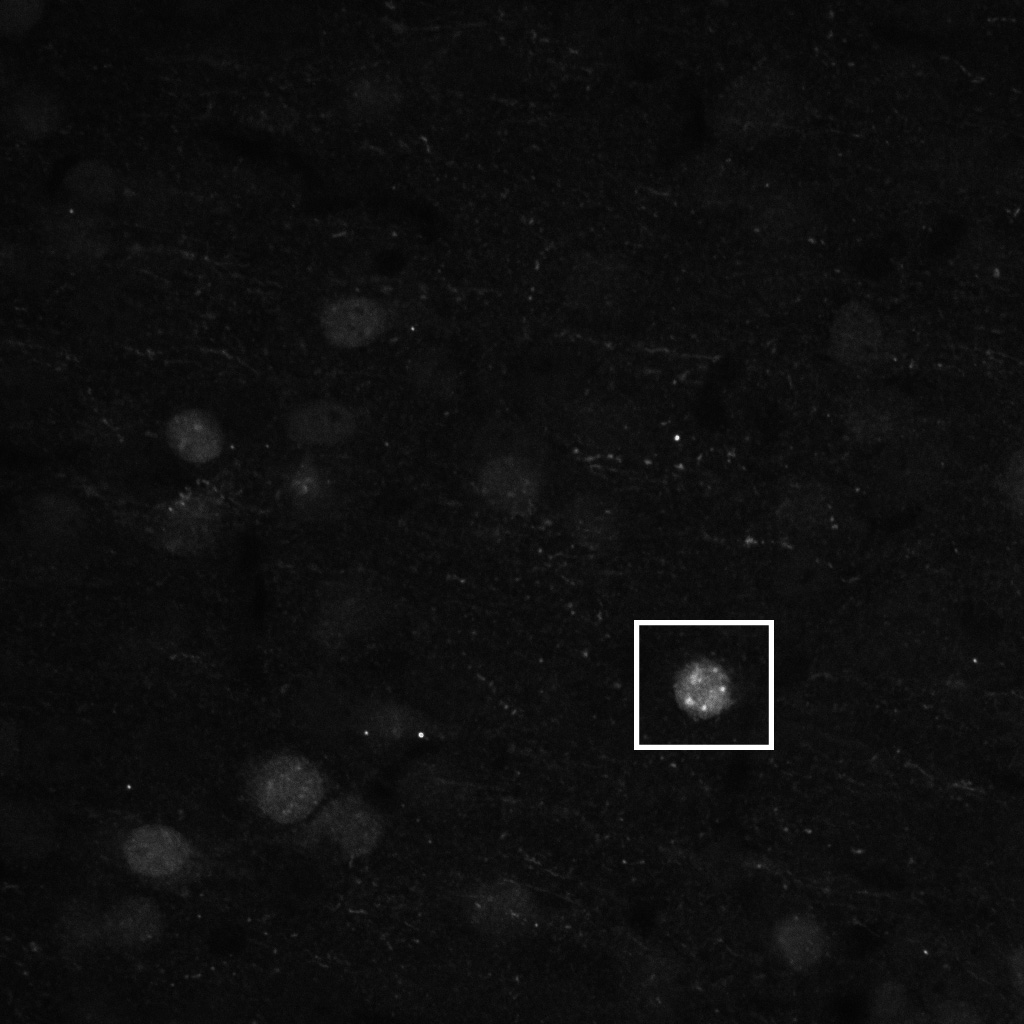

Supplement: Supplementary file 9 — Source data Fig. 1H [file 44318_2025_624_MOESM9_ESM.zip › 1H/n3/CFSE_Z stacks/11_1_C0_Z000 (45).jpg]

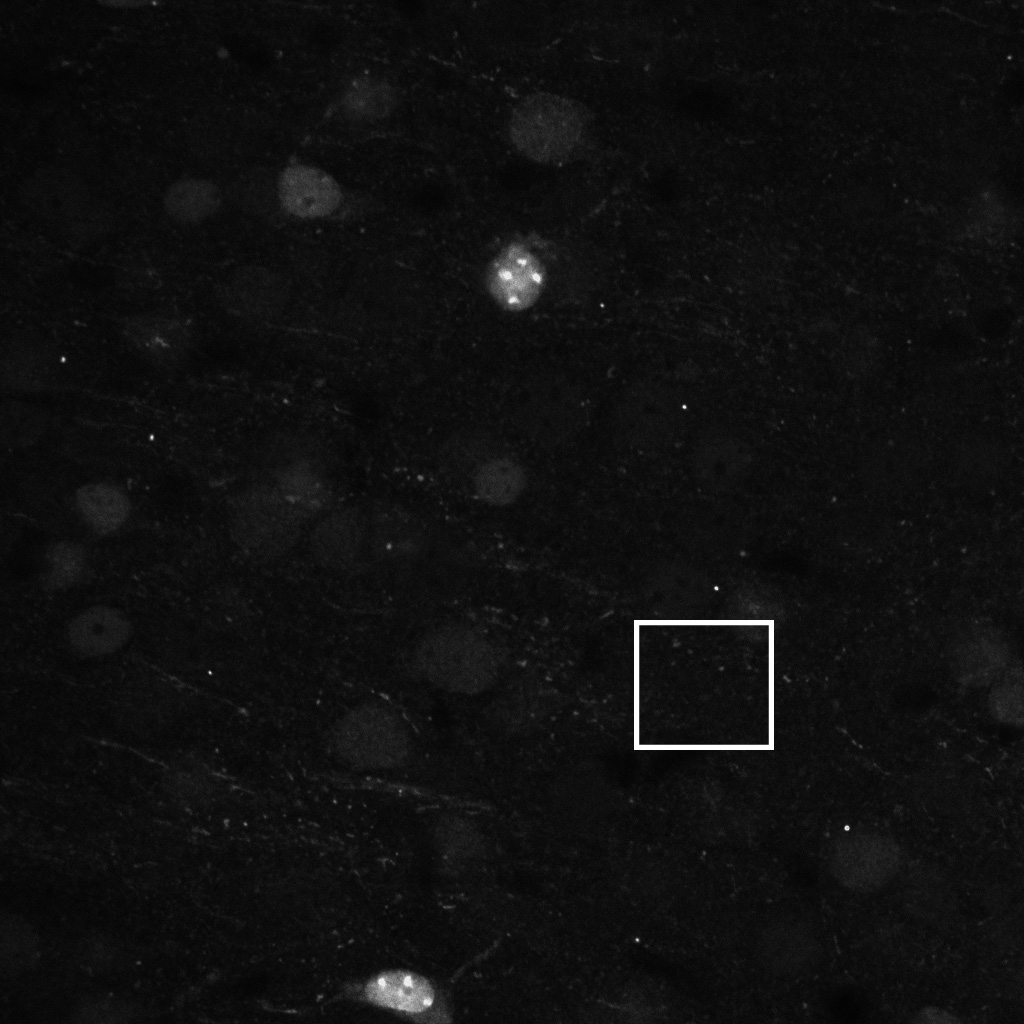

Supplement: Supplementary file 9 — Source data Fig. 1H [file 44318_2025_624_MOESM9_ESM.zip › 1H/n3/CFSE_Z stacks/11_1_C0_Z000 (19).jpg]

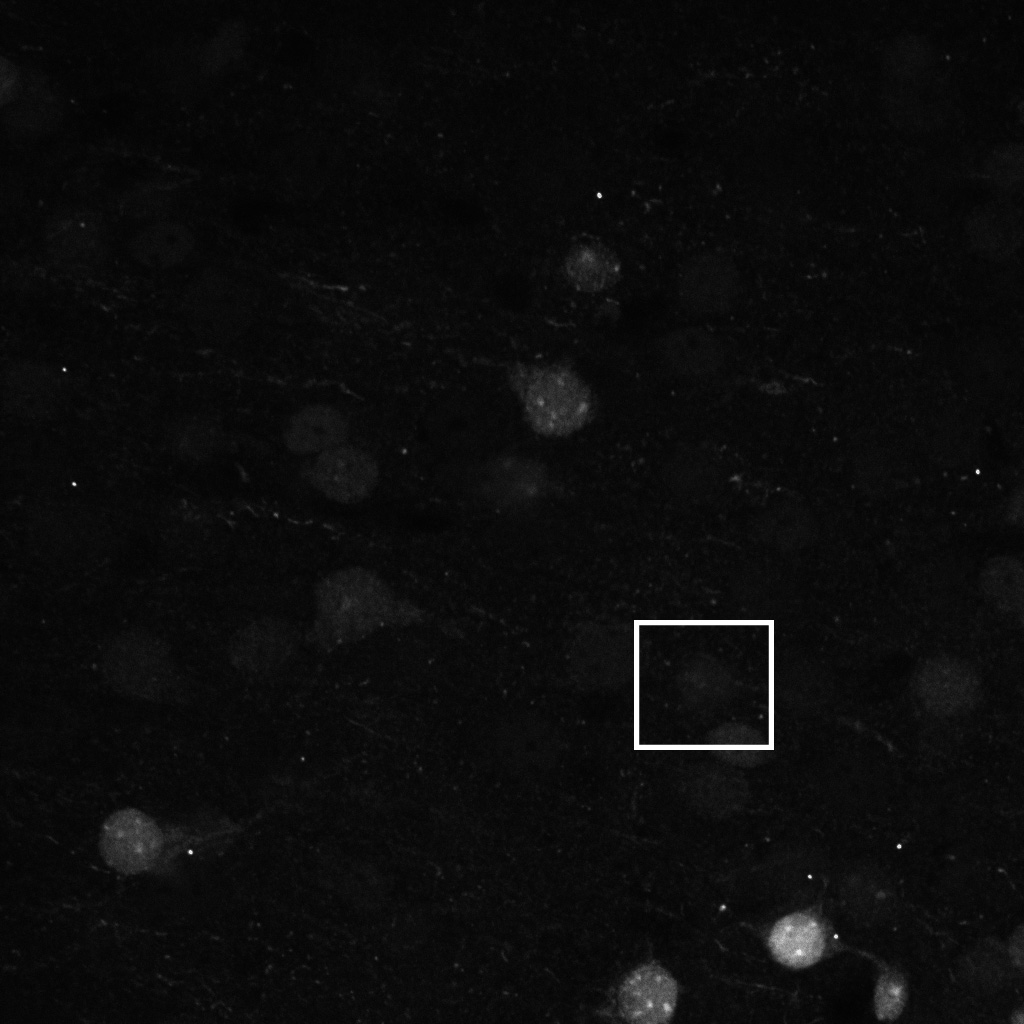

Supplement: Supplementary file 9 — Source data Fig. 1H [file 44318_2025_624_MOESM9_ESM.zip › 1H/n3/CFSE_Z stacks/11_1_C0_Z000 (58).jpg]

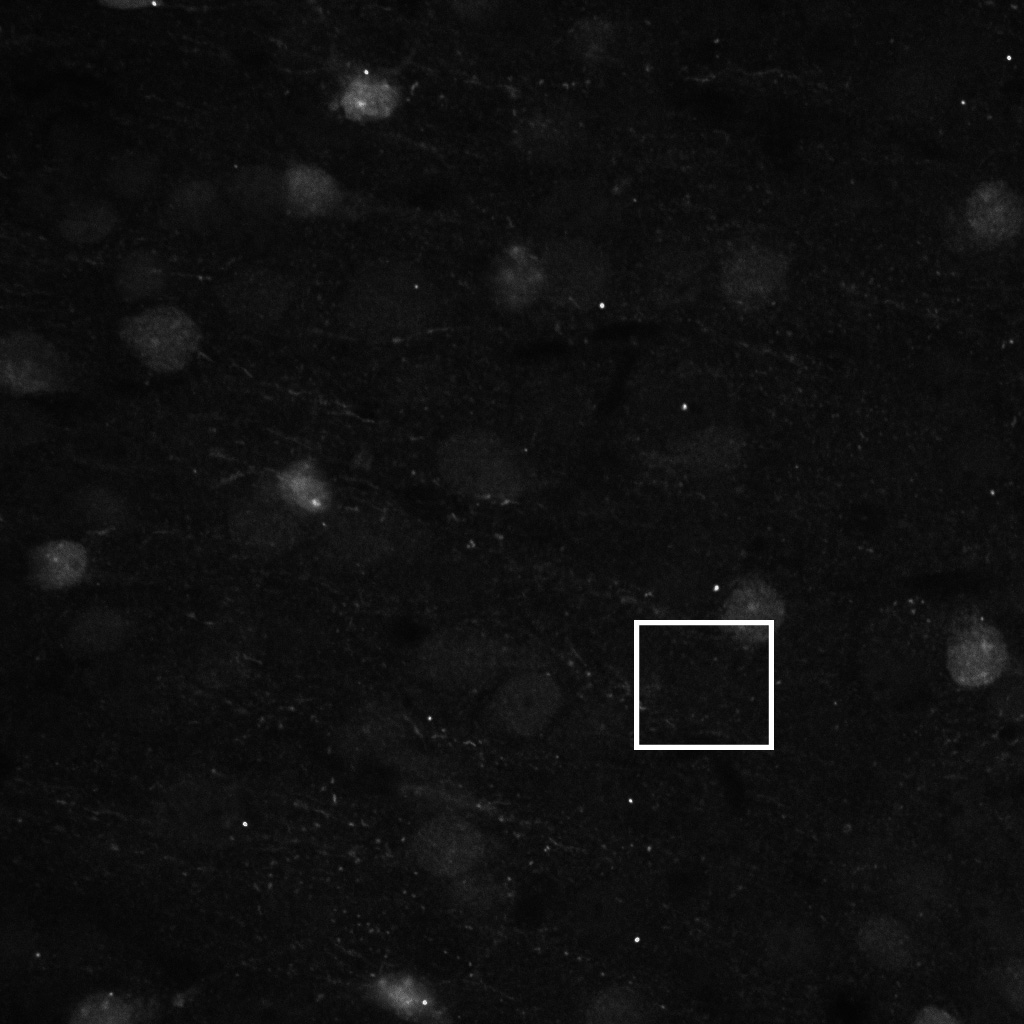

Supplement: Supplementary file 9 — Source data Fig. 1H [file 44318_2025_624_MOESM9_ESM.zip › 1H/n3/CFSE_Z stacks/11_1_C0_Z000 (23).jpg]

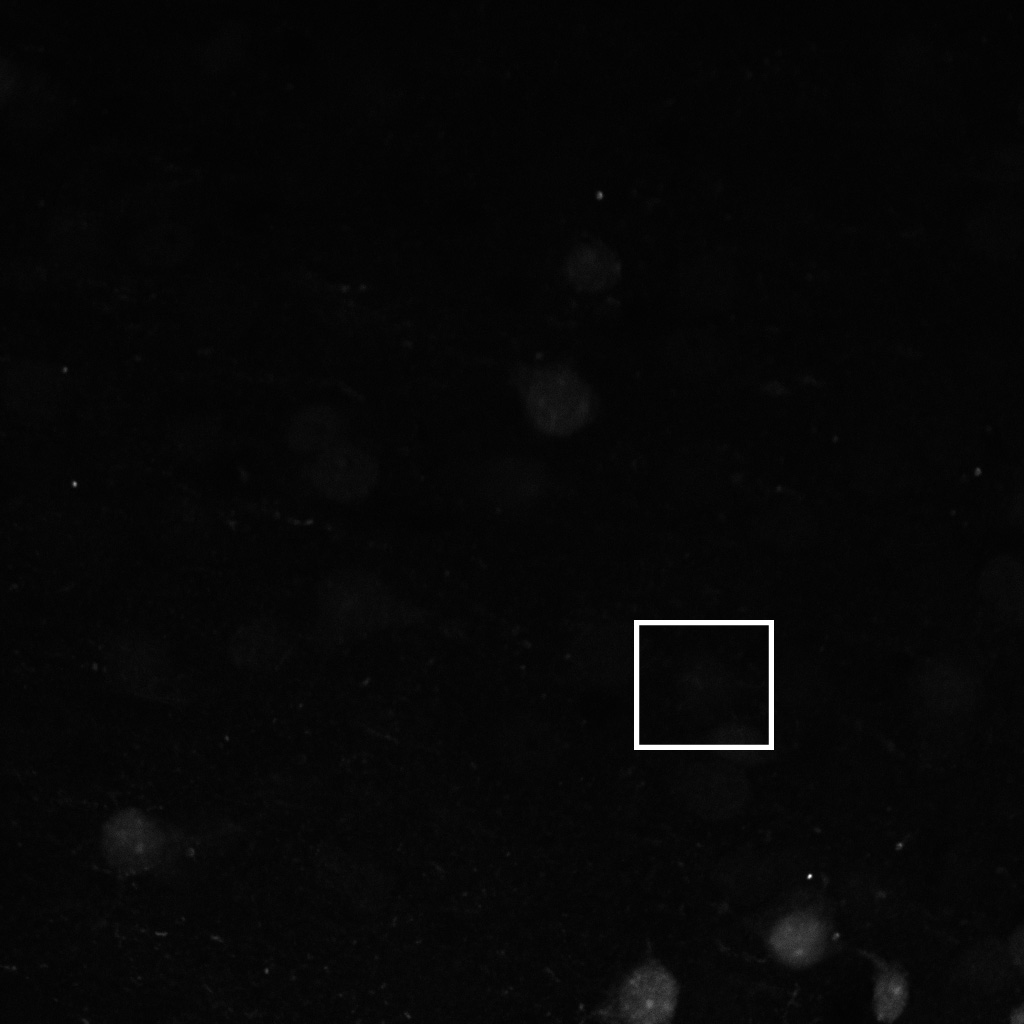

Supplement: Supplementary file 9 — Source data Fig. 1H [file 44318_2025_624_MOESM9_ESM.zip › 1H/n3/CFSE_Z stacks/11_1_C0_Z000 (62).jpg]

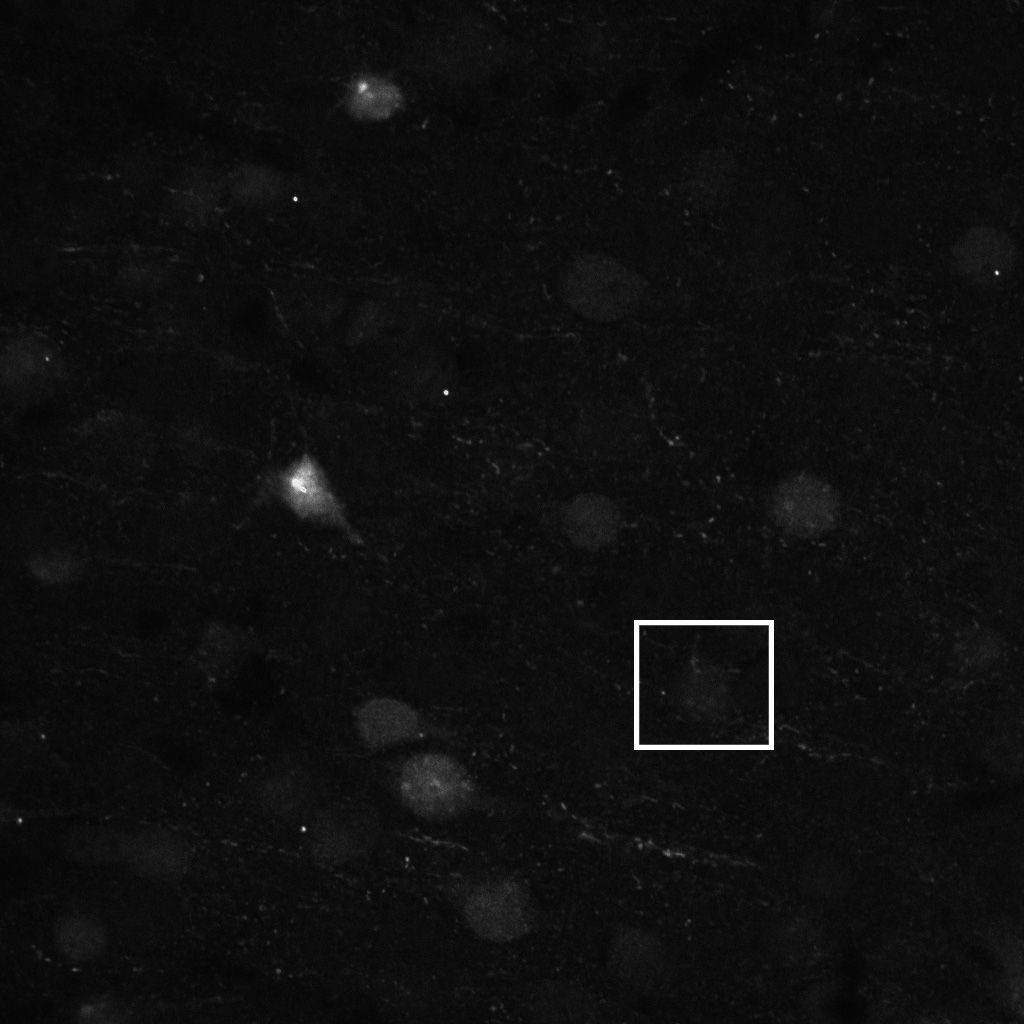

Supplement: Supplementary file 9 — Source data Fig. 1H [file 44318_2025_624_MOESM9_ESM.zip › 1H/n3/CFSE_Z stacks/11_1_C0_Z000 (35).jpg]

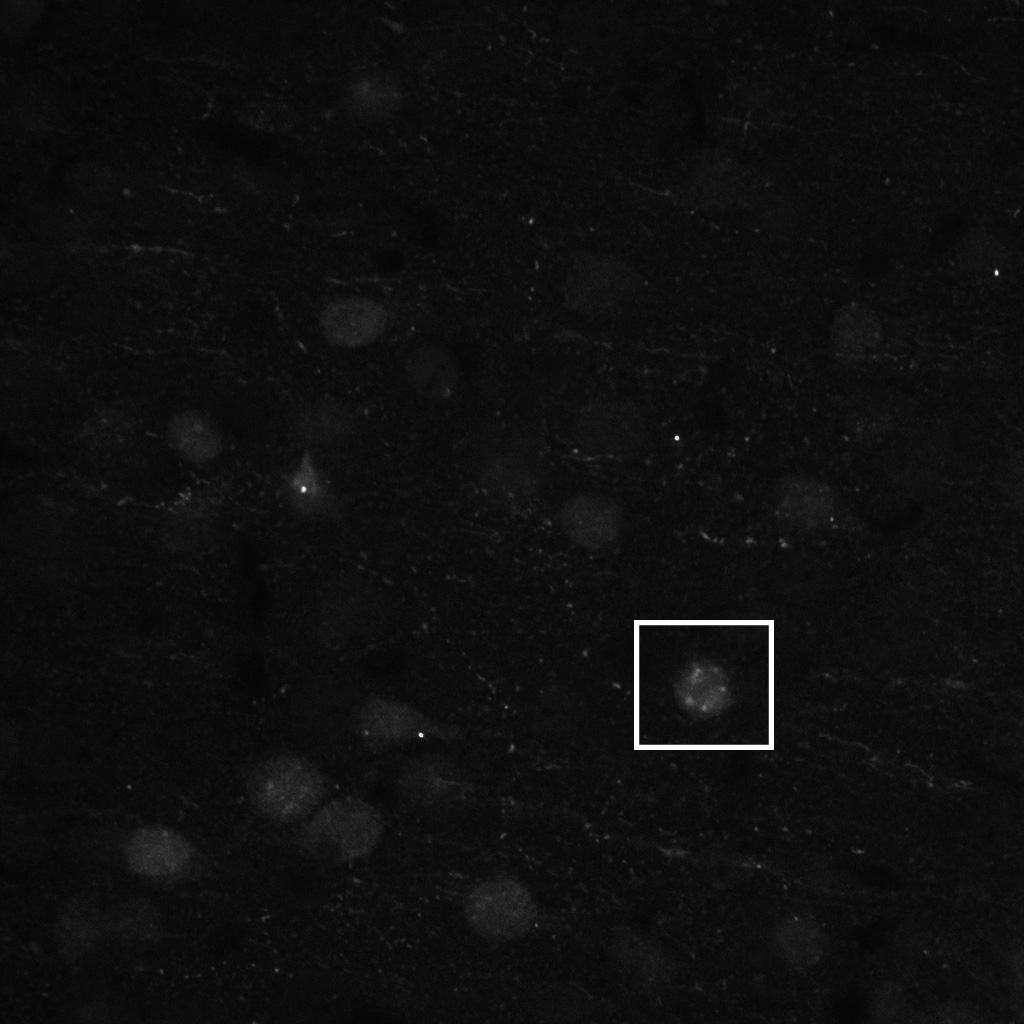

Supplement: Supplementary file 9 — Source data Fig. 1H [file 44318_2025_624_MOESM9_ESM.zip › 1H/n3/CFSE_Z stacks/11_1_C0_Z000 (42).jpg]

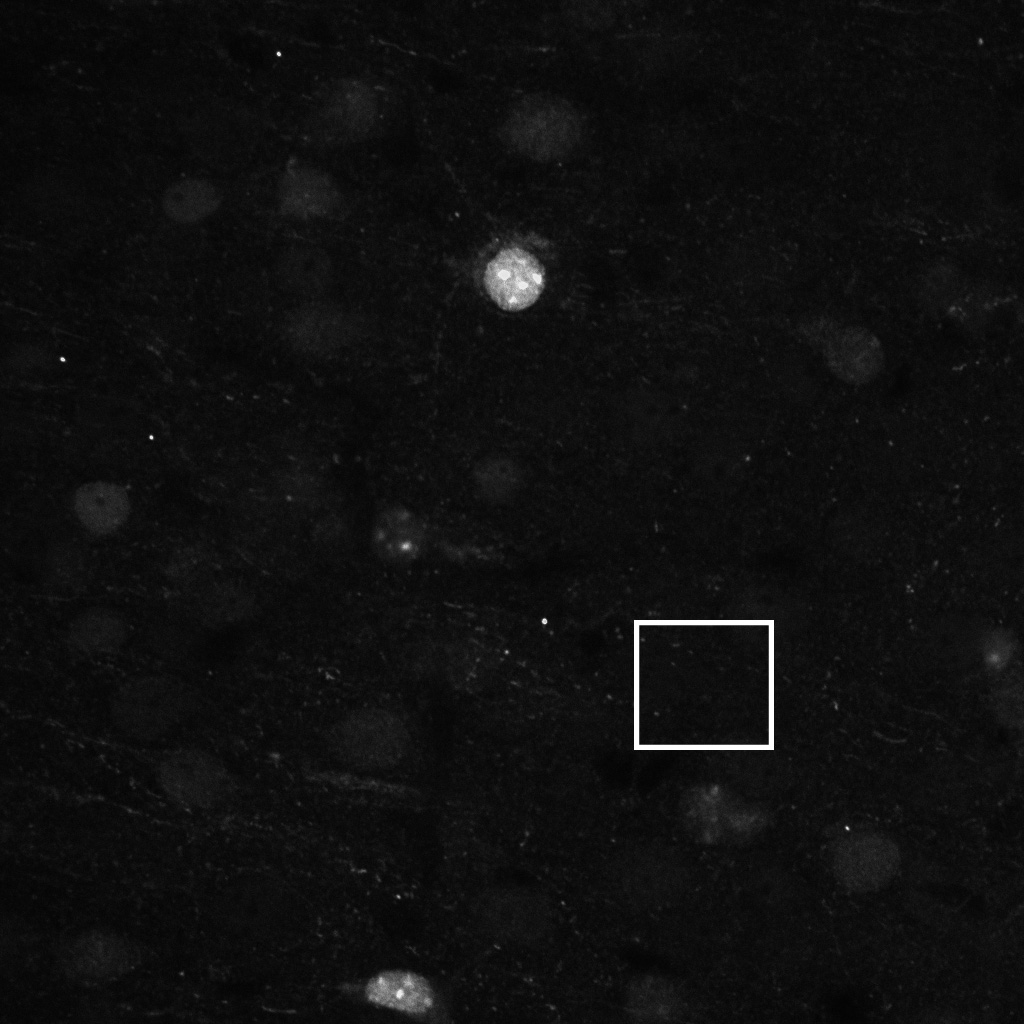

Supplement: Supplementary file 9 — Source data Fig. 1H [file 44318_2025_624_MOESM9_ESM.zip › 1H/n3/CFSE_Z stacks/11_1_C0_Z000 (15).jpg]

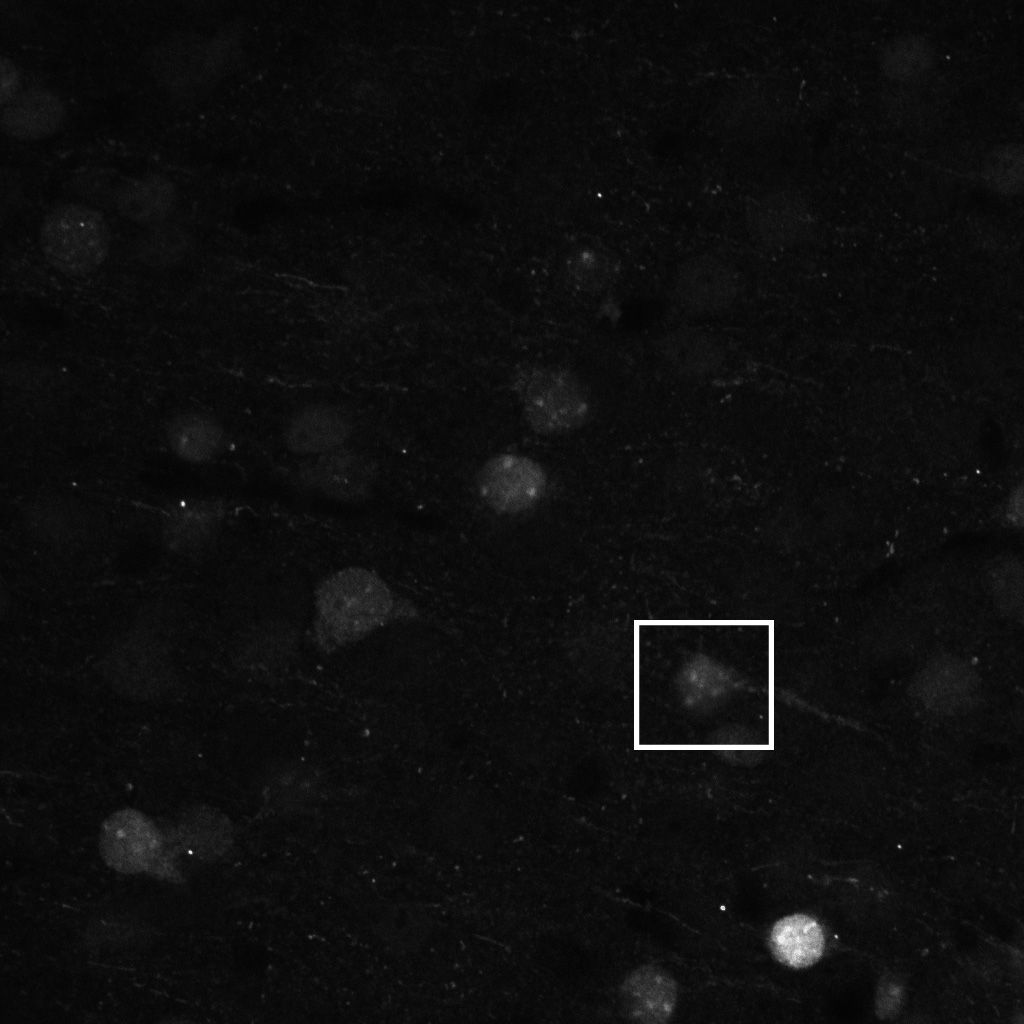

Supplement: Supplementary file 9 — Source data Fig. 1H [file 44318_2025_624_MOESM9_ESM.zip › 1H/n3/CFSE_Z stacks/11_1_C0_Z000 (54).jpg]

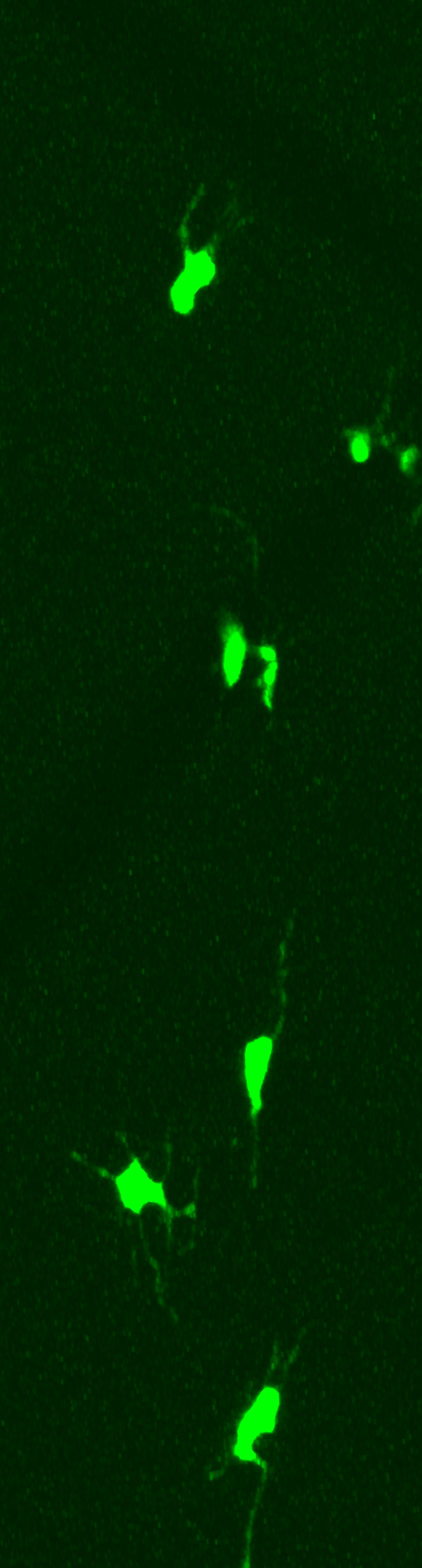

Supplement: Supplementary file 12 — Source data Fig. 4 [file 44318_2025_624_MOESM12_ESM.zip › Figure 4/4B/12h.tif]

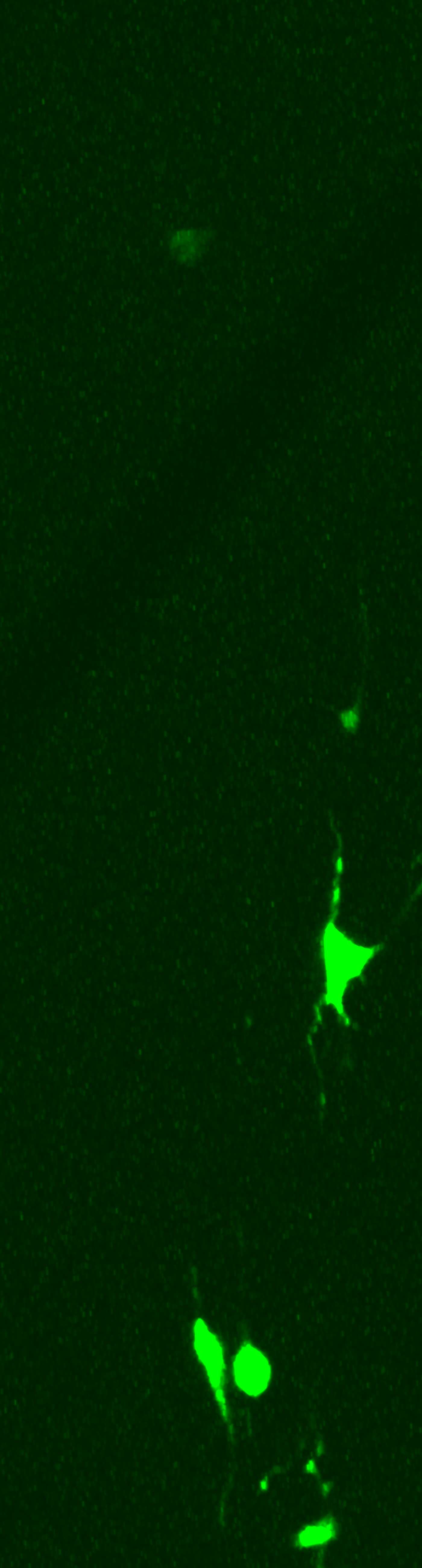

Supplement: Supplementary file 12 — Source data Fig. 4 [file 44318_2025_624_MOESM12_ESM.zip › Figure 4/4B/0.5h.tif]

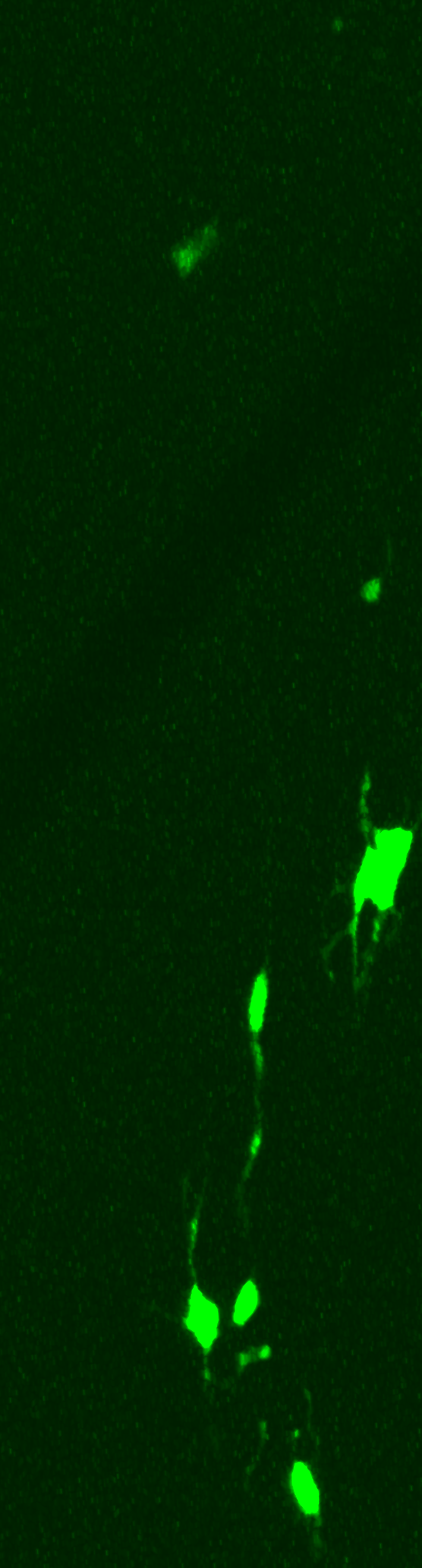

Supplement: Supplementary file 12 — Source data Fig. 4 [file 44318_2025_624_MOESM12_ESM.zip › Figure 4/4B/3h.tif]

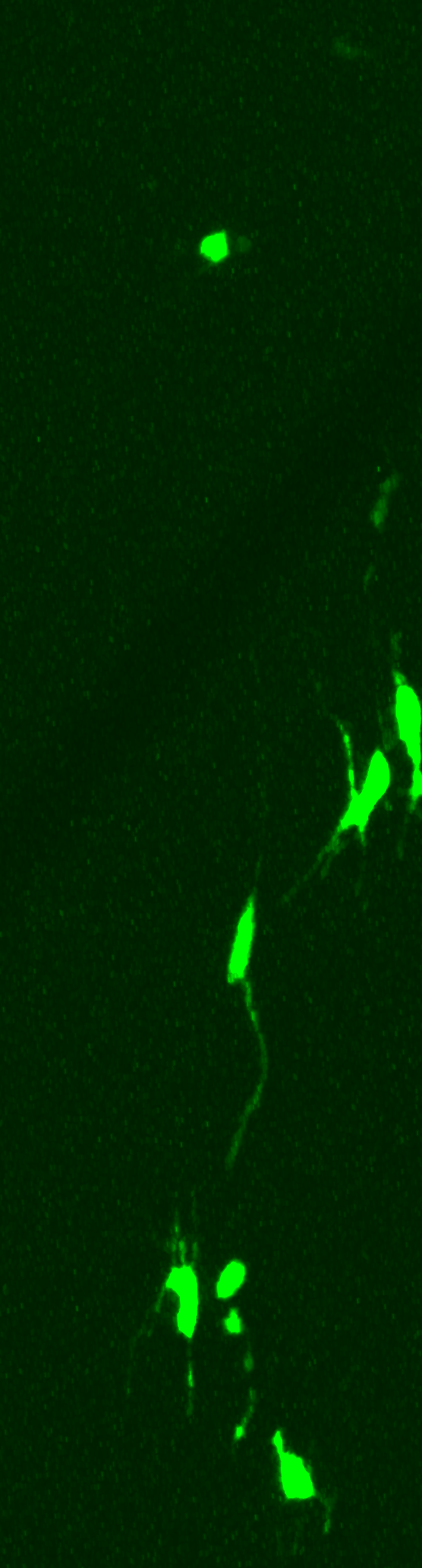

Supplement: Supplementary file 12 — Source data Fig. 4 [file 44318_2025_624_MOESM12_ESM.zip › Figure 4/4B/5h.tif]

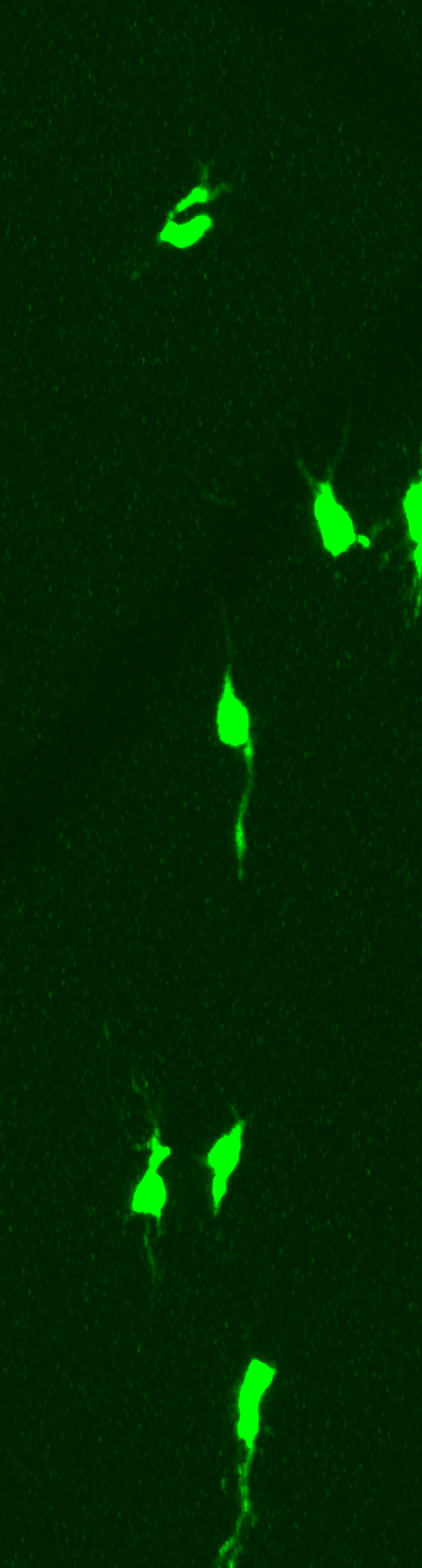

Supplement: Supplementary file 12 — Source data Fig. 4 [file 44318_2025_624_MOESM12_ESM.zip › Figure 4/4B/9h.tif]

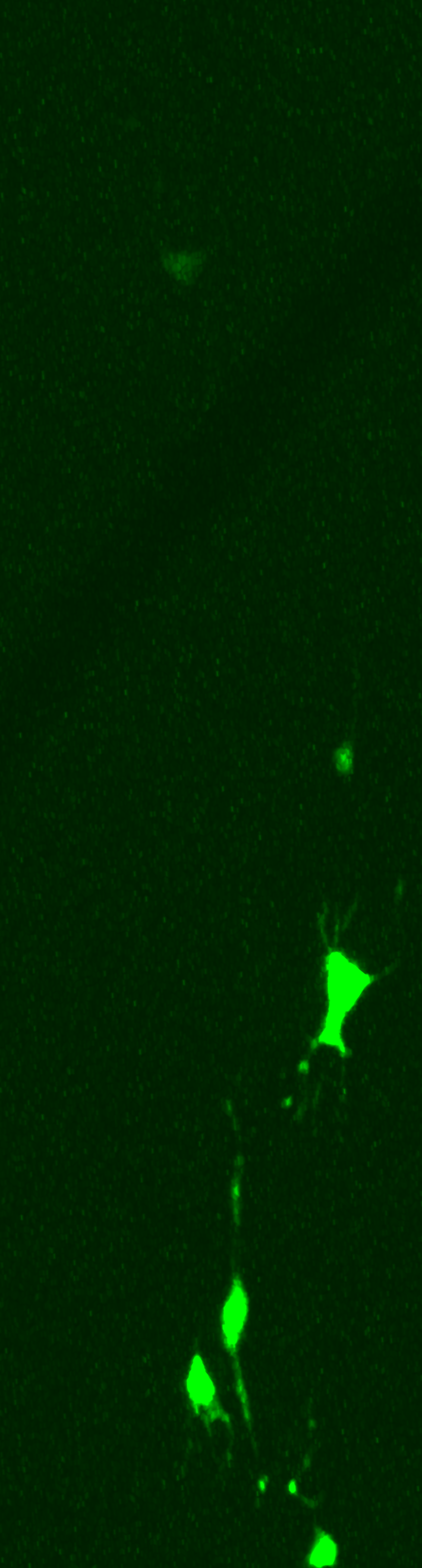

Supplement: Supplementary file 12 — Source data Fig. 4 [file 44318_2025_624_MOESM12_ESM.zip › Figure 4/4B/0.0h.tif]

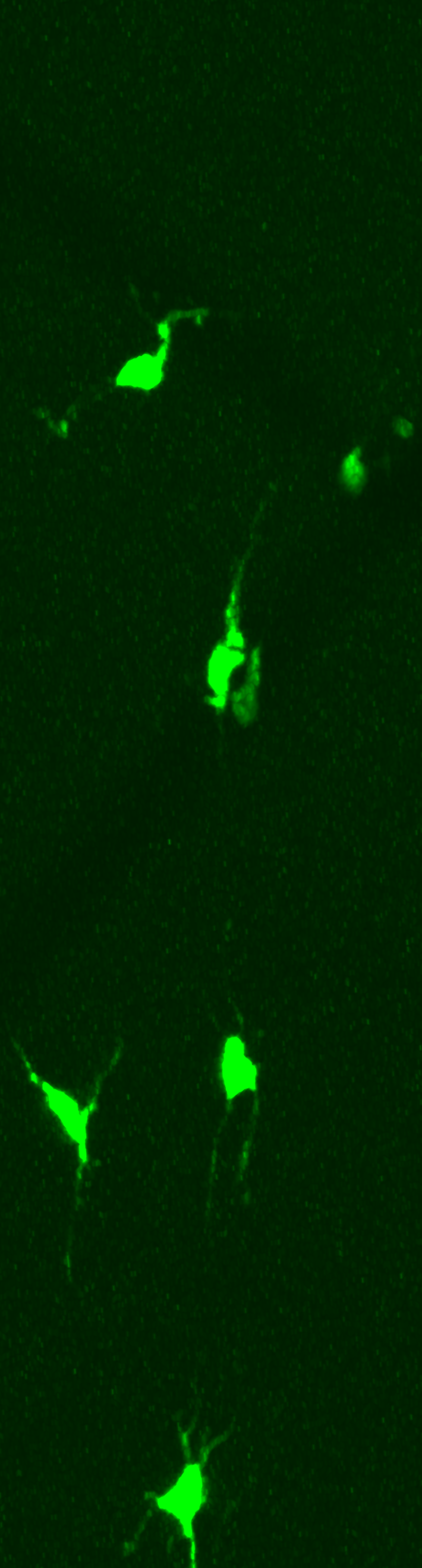

Supplement: Supplementary file 12 — Source data Fig. 4 [file 44318_2025_624_MOESM12_ESM.zip › Figure 4/4B/17h.tif]

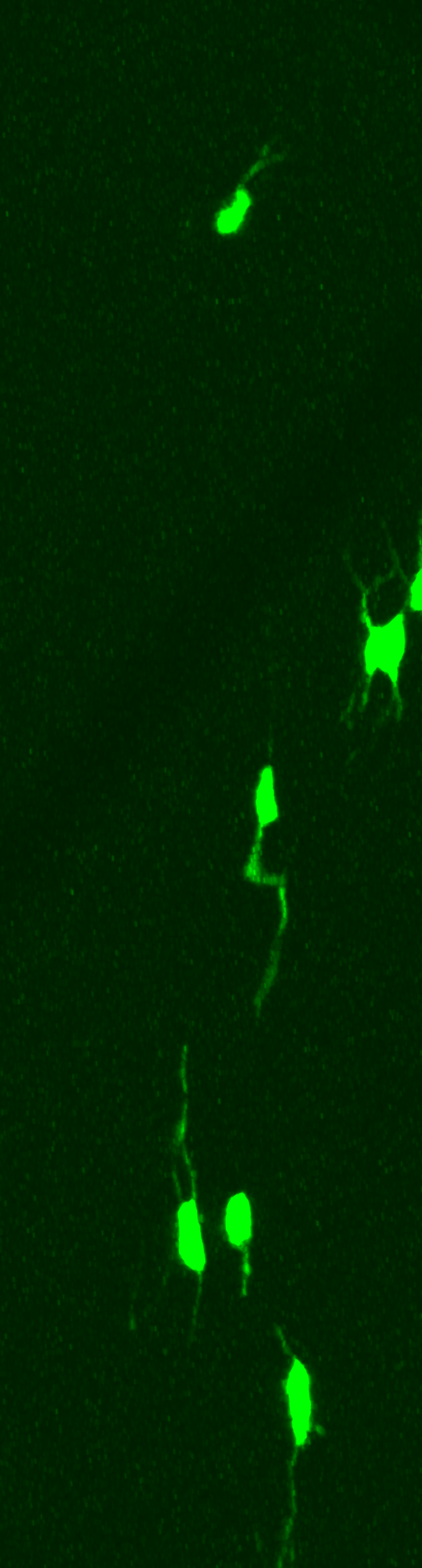

Supplement: Supplementary file 12 — Source data Fig. 4 [file 44318_2025_624_MOESM12_ESM.zip › Figure 4/4B/7h.tif]

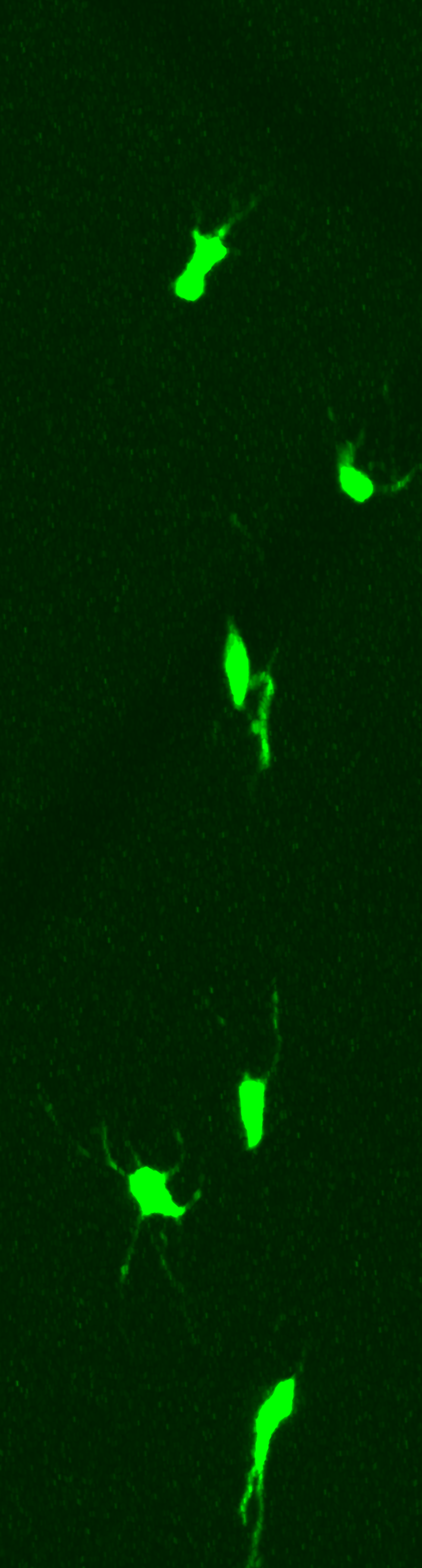

Supplement: Supplementary file 12 — Source data Fig. 4 [file 44318_2025_624_MOESM12_ESM.zip › Figure 4/4B/11h.tif]

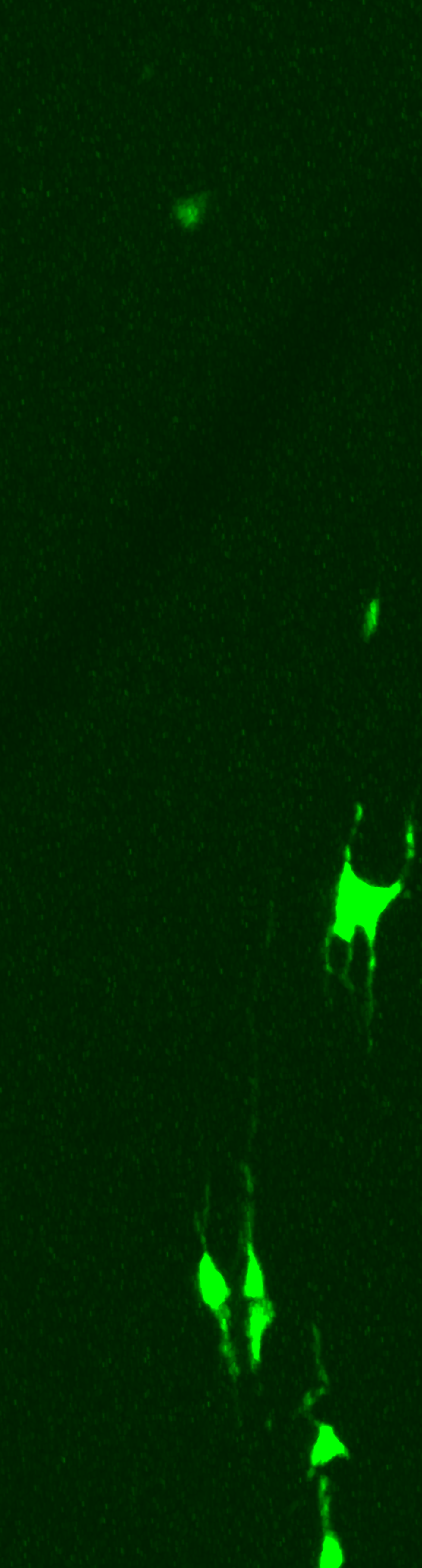

Supplement: Supplementary file 12 — Source data Fig. 4 [file 44318_2025_624_MOESM12_ESM.zip › Figure 4/4B/1h.tif]

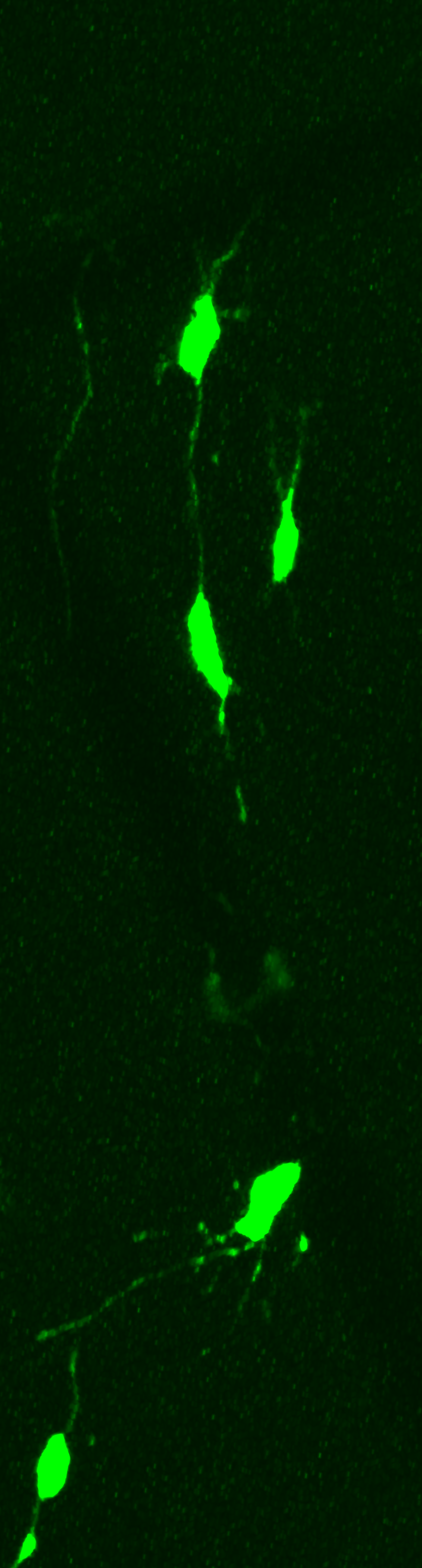

Supplement: Supplementary file 12 — Source data Fig. 4 [file 44318_2025_624_MOESM12_ESM.zip › Figure 4/4D/28h.tif]

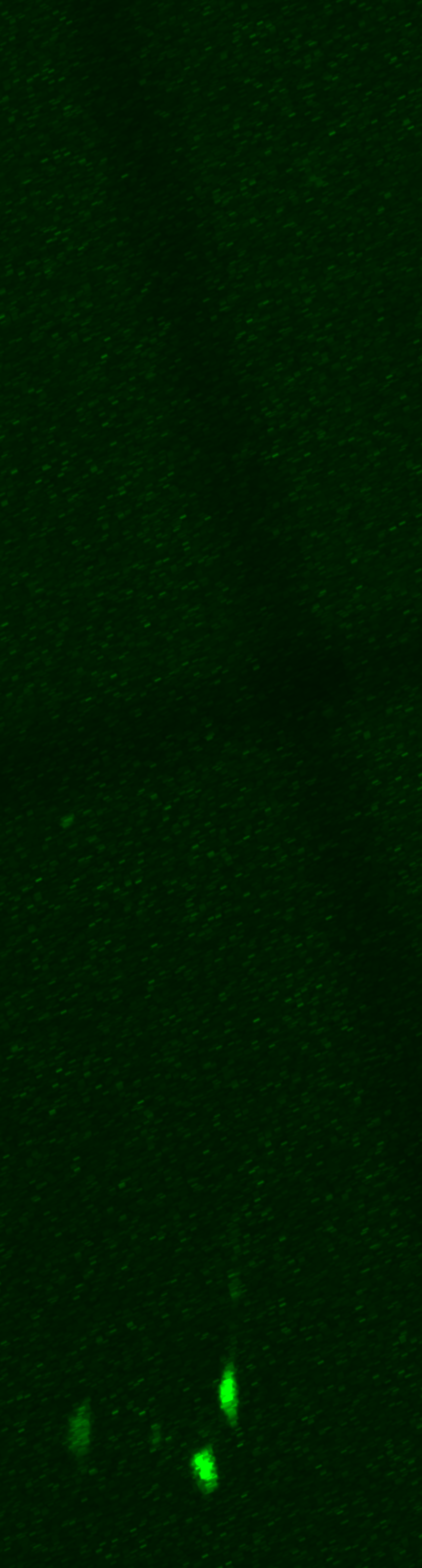

Supplement: Supplementary file 12 — Source data Fig. 4 [file 44318_2025_624_MOESM12_ESM.zip › Figure 4/4D/2.5h.tif]

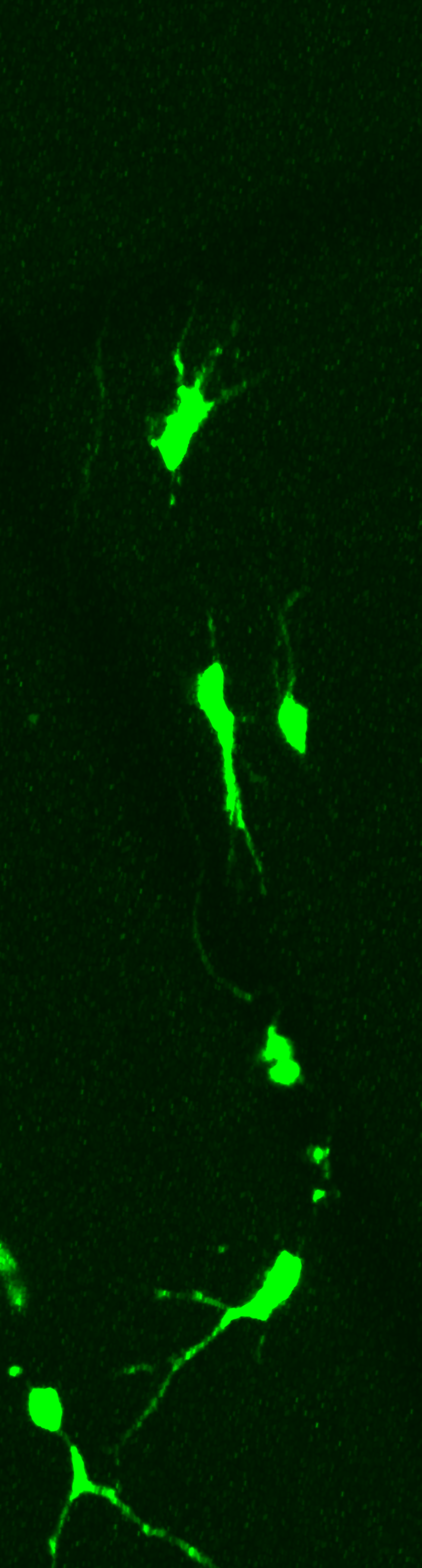

Supplement: Supplementary file 12 — Source data Fig. 4 [file 44318_2025_624_MOESM12_ESM.zip › Figure 4/4D/25h.tif]

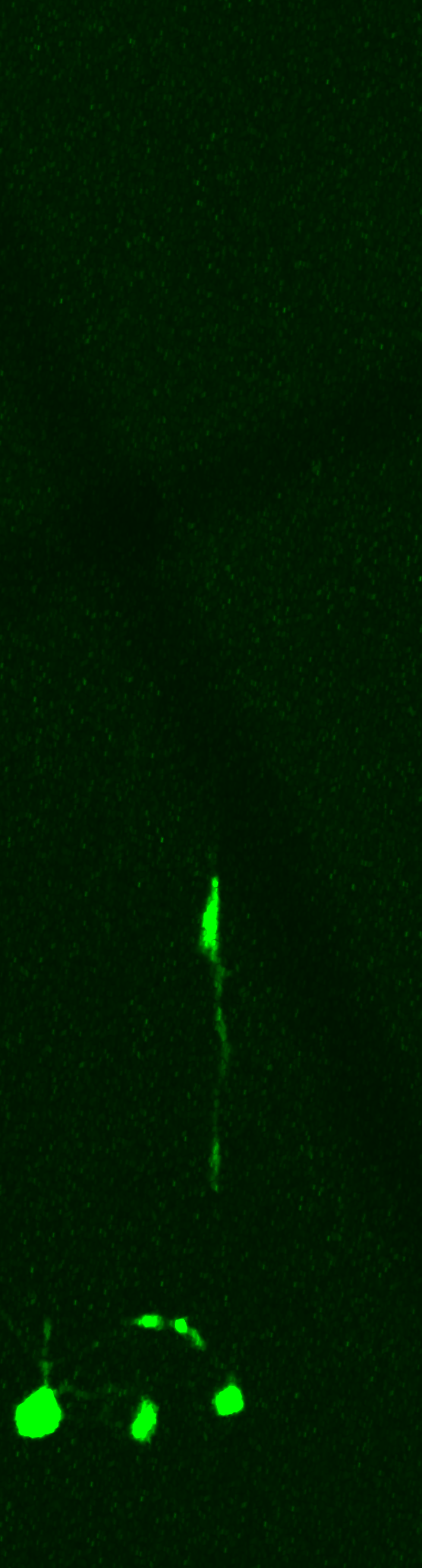

Supplement: Supplementary file 12 — Source data Fig. 4 [file 44318_2025_624_MOESM12_ESM.zip › Figure 4/4D/7.0h.tif]

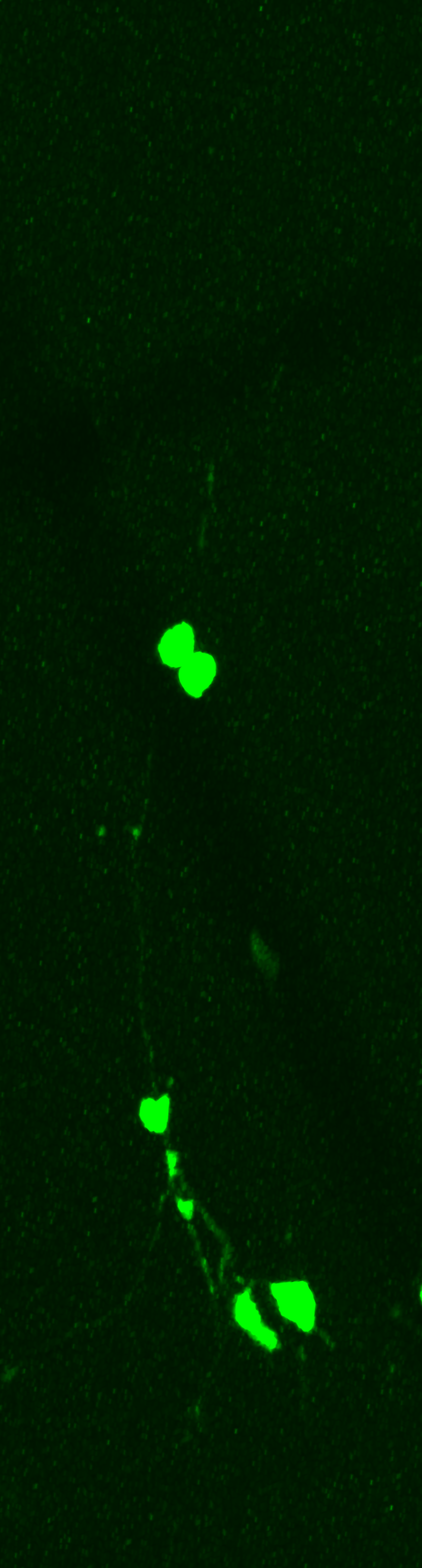

Supplement: Supplementary file 12 — Source data Fig. 4 [file 44318_2025_624_MOESM12_ESM.zip › Figure 4/4D/14h.tif]

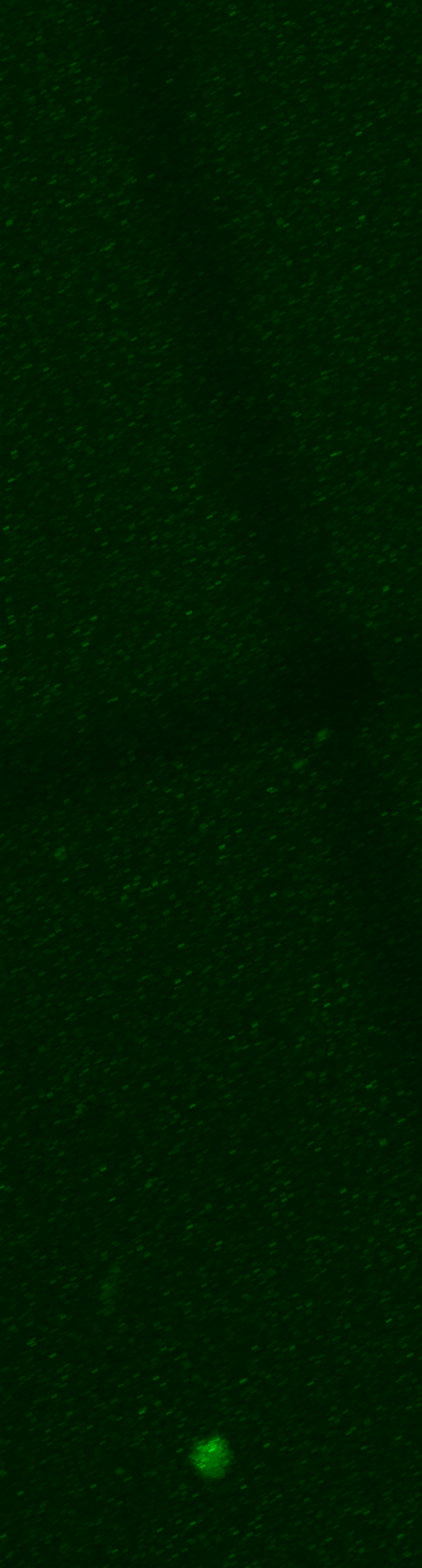

Supplement: Supplementary file 12 — Source data Fig. 4 [file 44318_2025_624_MOESM12_ESM.zip › Figure 4/4D/0.0h.tif]

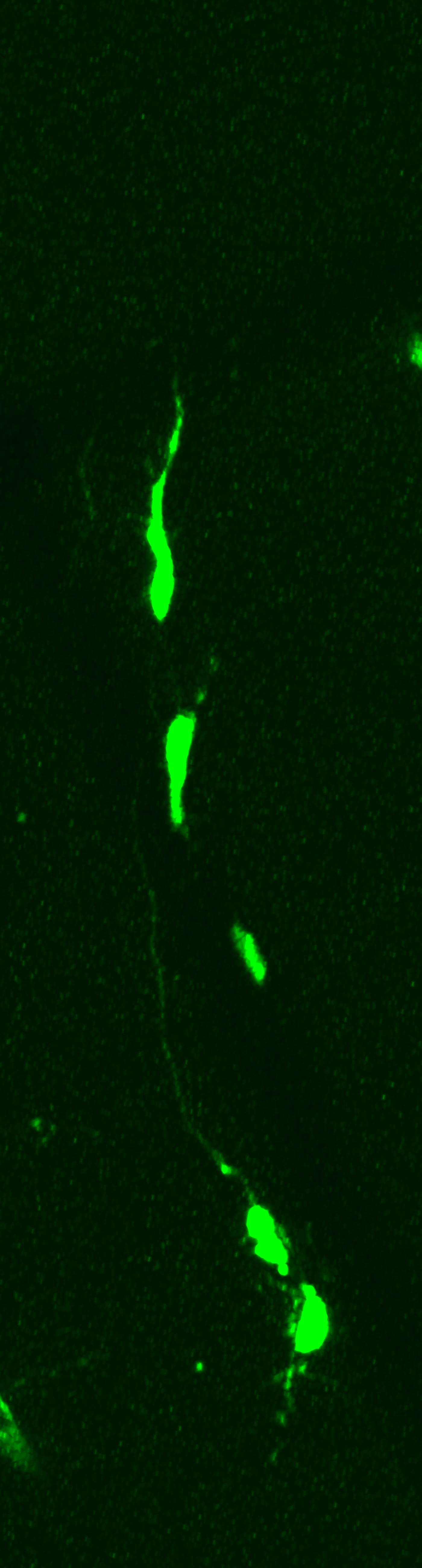

Supplement: Supplementary file 12 — Source data Fig. 4 [file 44318_2025_624_MOESM12_ESM.zip › Figure 4/4D/20.5h.tif]

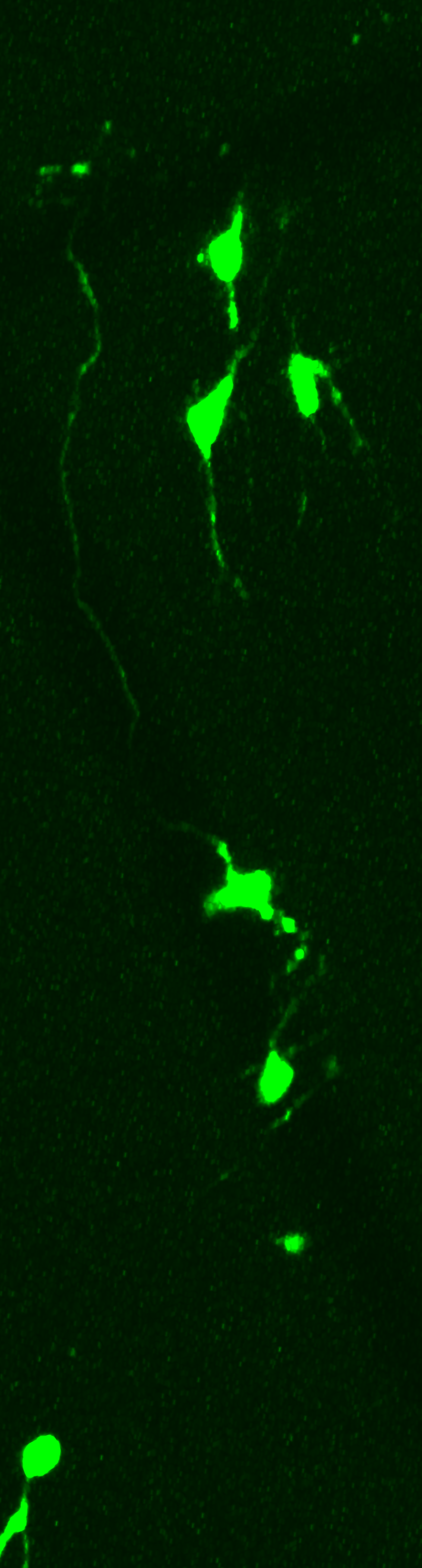

Supplement: Supplementary file 12 — Source data Fig. 4 [file 44318_2025_624_MOESM12_ESM.zip › Figure 4/4D/32.5h.tif]

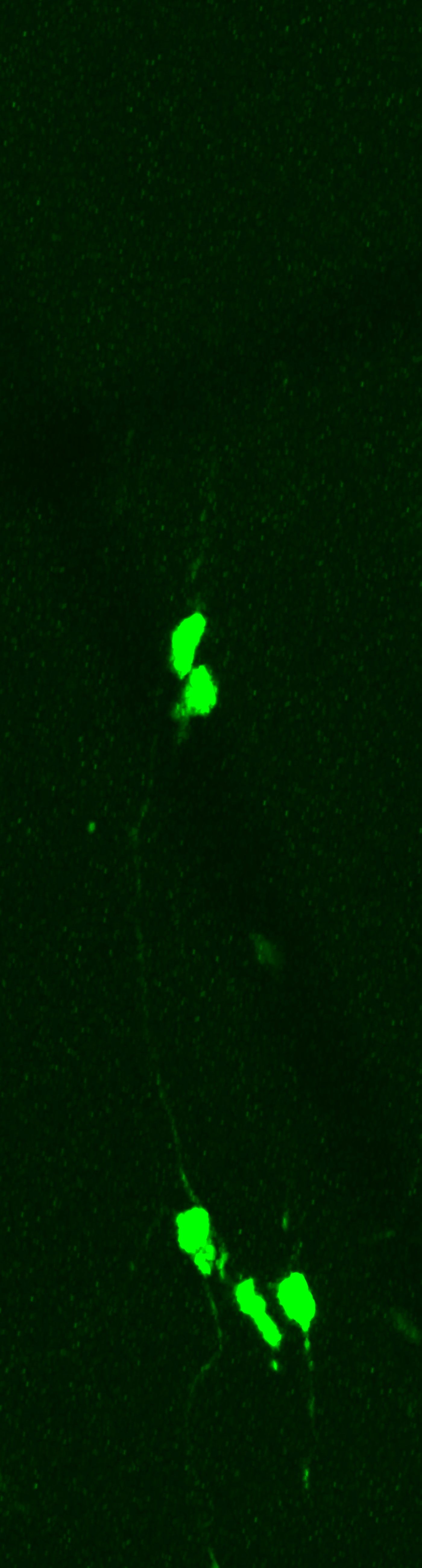

Supplement: Supplementary file 12 — Source data Fig. 4 [file 44318_2025_624_MOESM12_ESM.zip › Figure 4/4D/14.5h.tif]

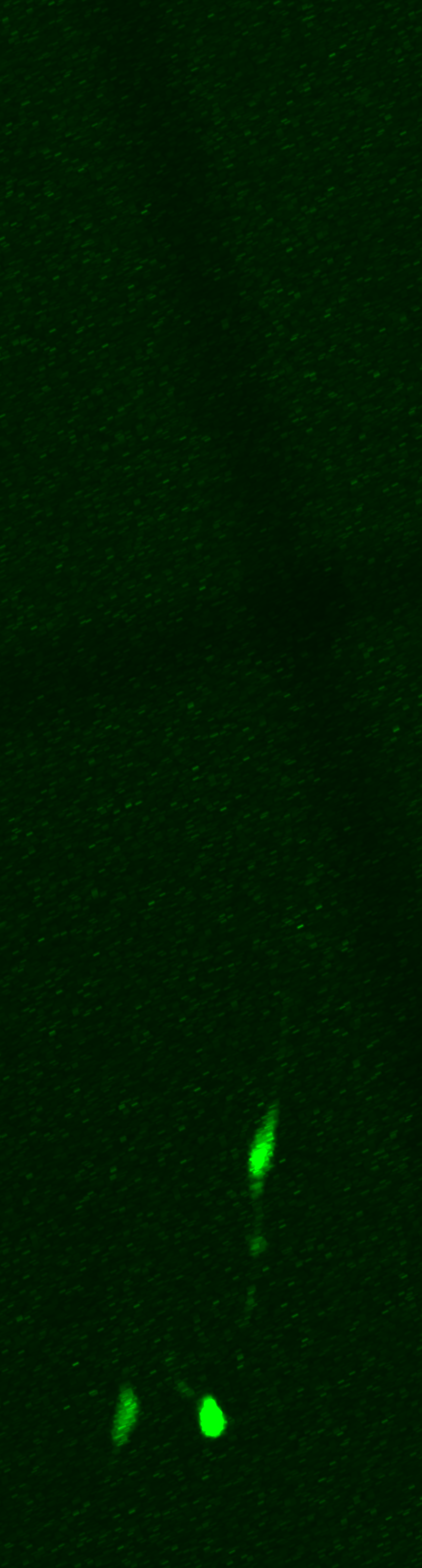

Supplement: Supplementary file 12 — Source data Fig. 4 [file 44318_2025_624_MOESM12_ESM.zip › Figure 4/4D/4.0h.tif]

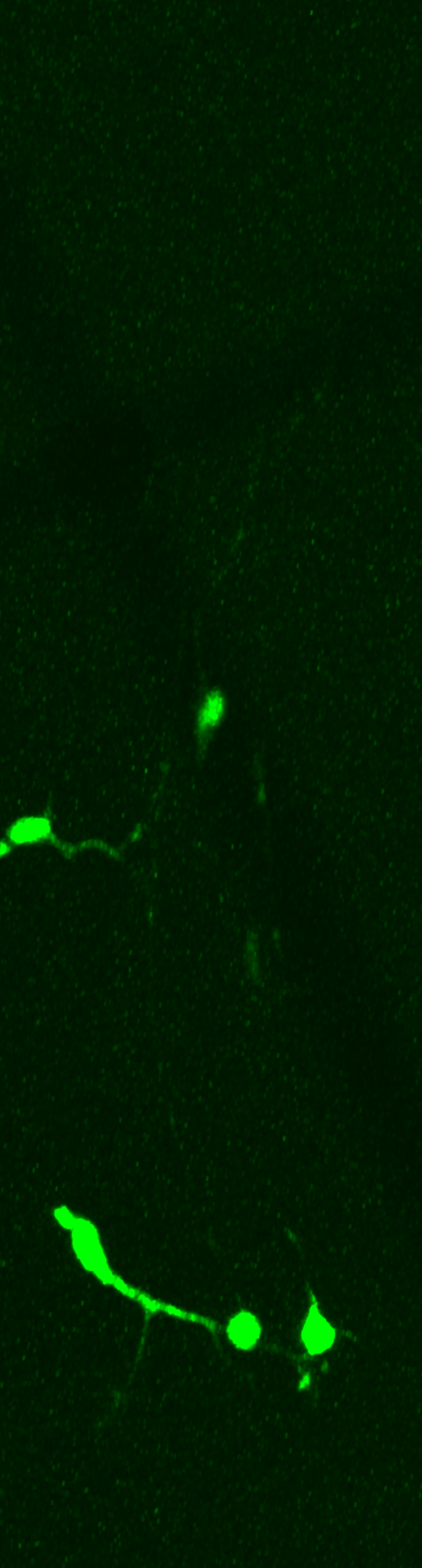

Supplement: Supplementary file 12 — Source data Fig. 4 [file 44318_2025_624_MOESM12_ESM.zip › Figure 4/4D/11h.tif]

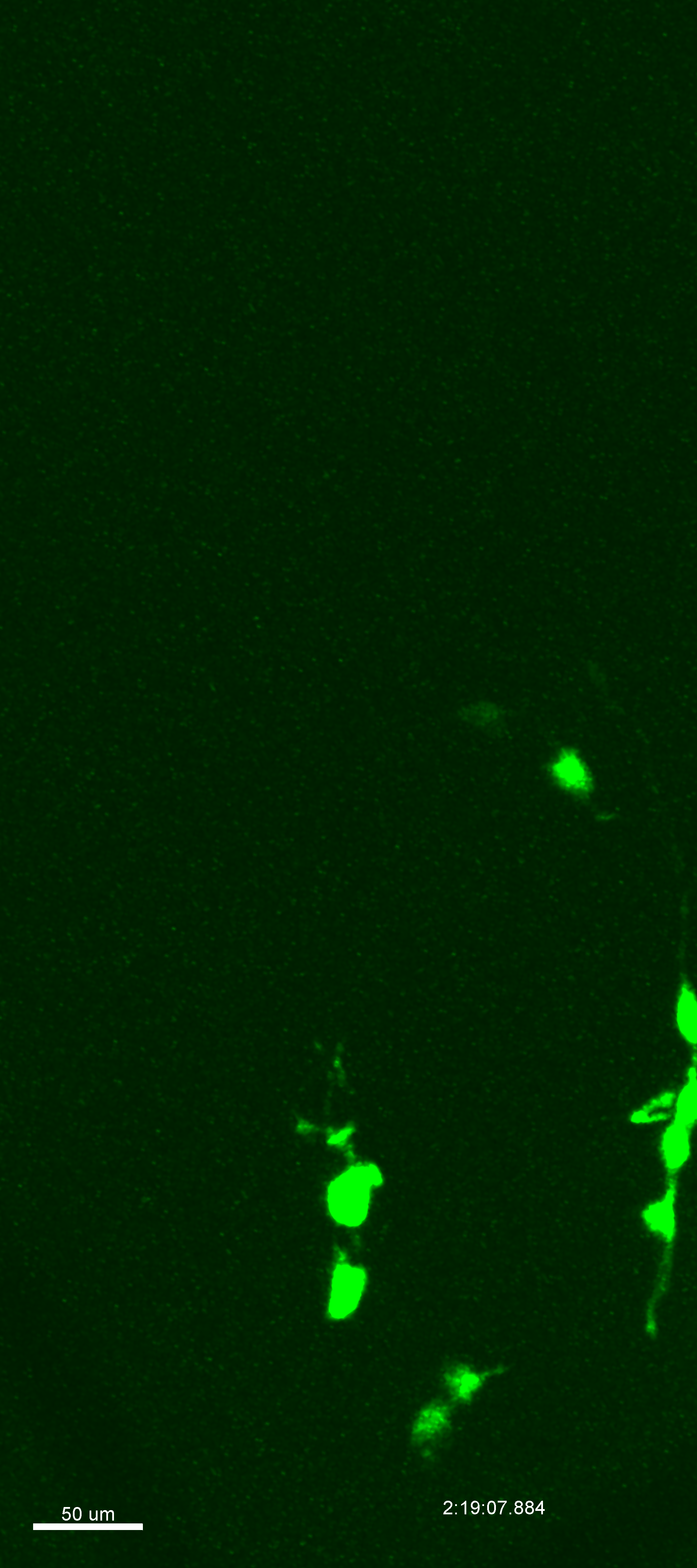

Supplement: Supplementary file 12 — Source data Fig. 4 [file 44318_2025_624_MOESM12_ESM.zip › Figure 4/4F/2.5h.tif]

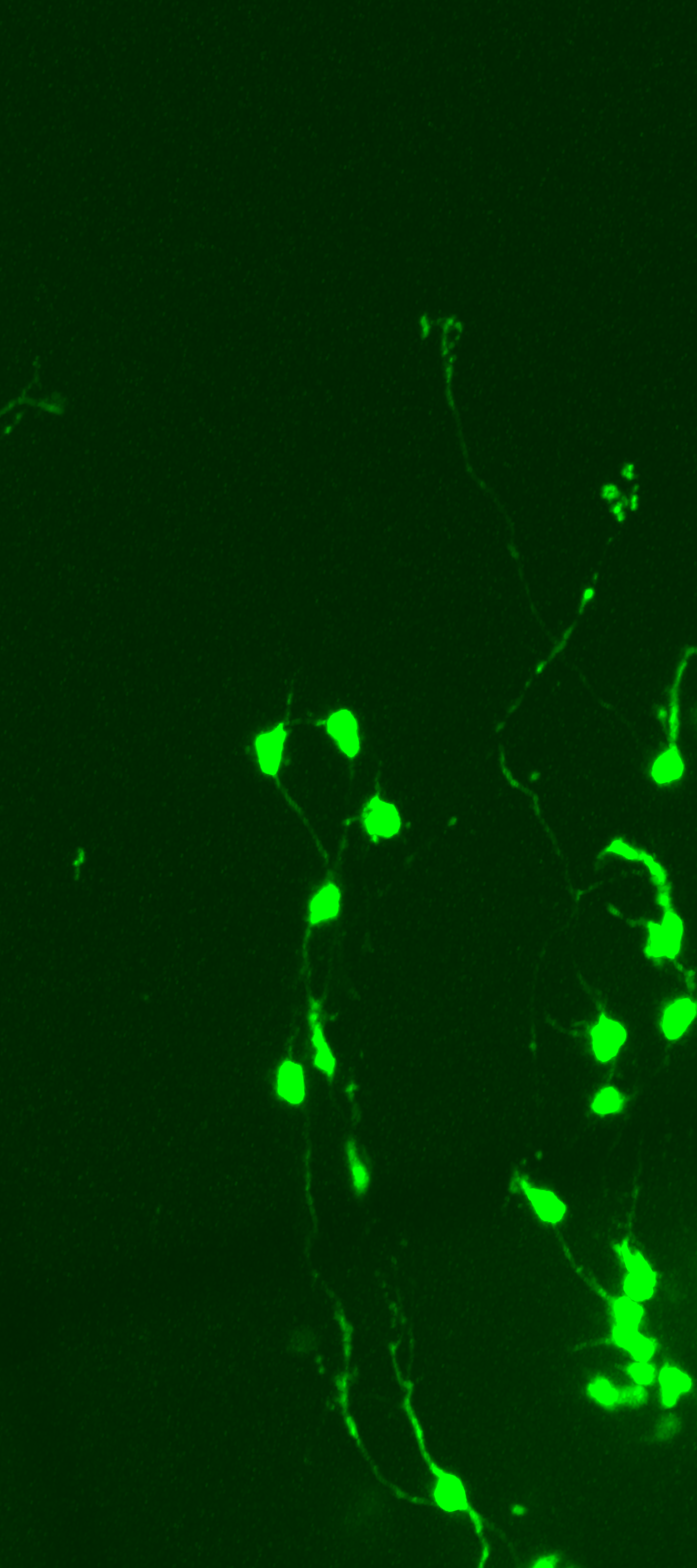

Supplement: Supplementary file 12 — Source data Fig. 4 [file 44318_2025_624_MOESM12_ESM.zip › Figure 4/4F/43.0h.tif]

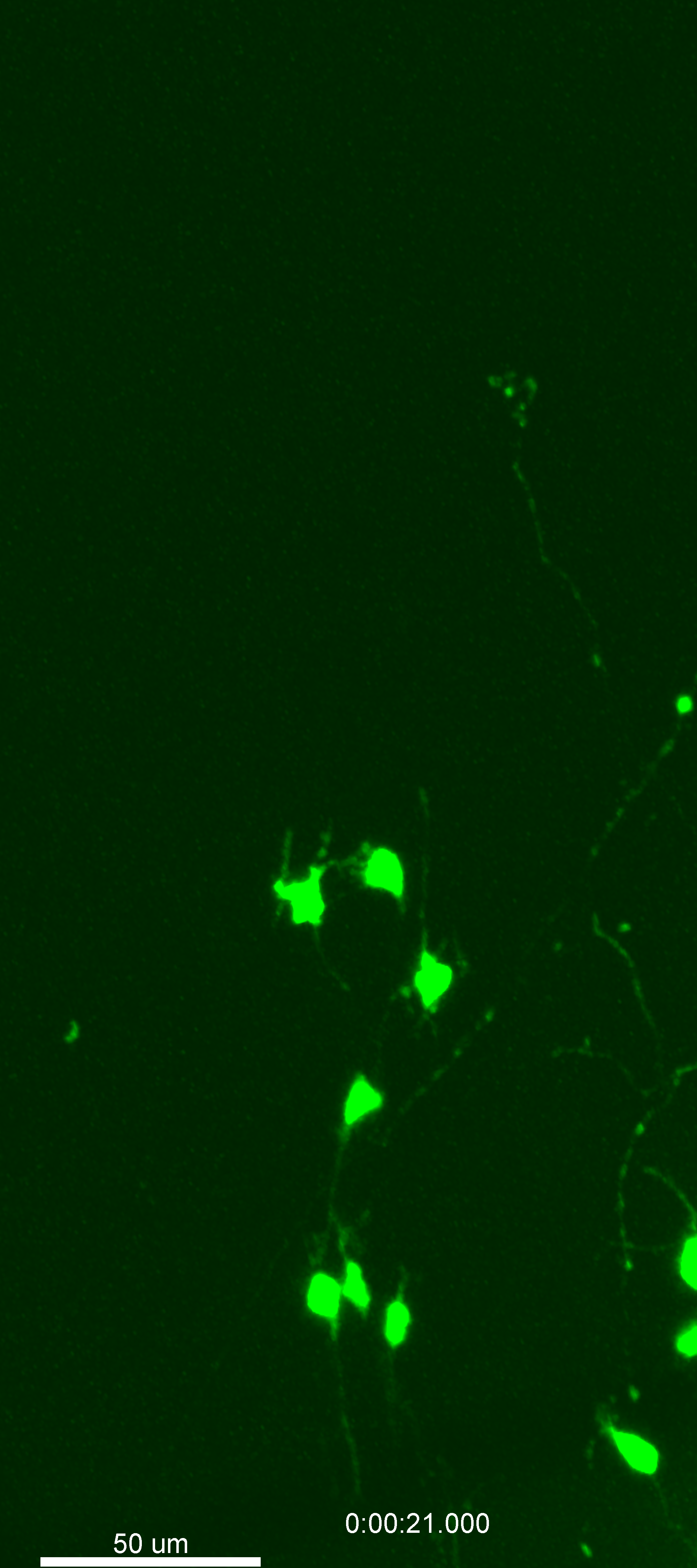

Supplement: Supplementary file 12 — Source data Fig. 4 [file 44318_2025_624_MOESM12_ESM.zip › Figure 4/4F/40.5h.tif]

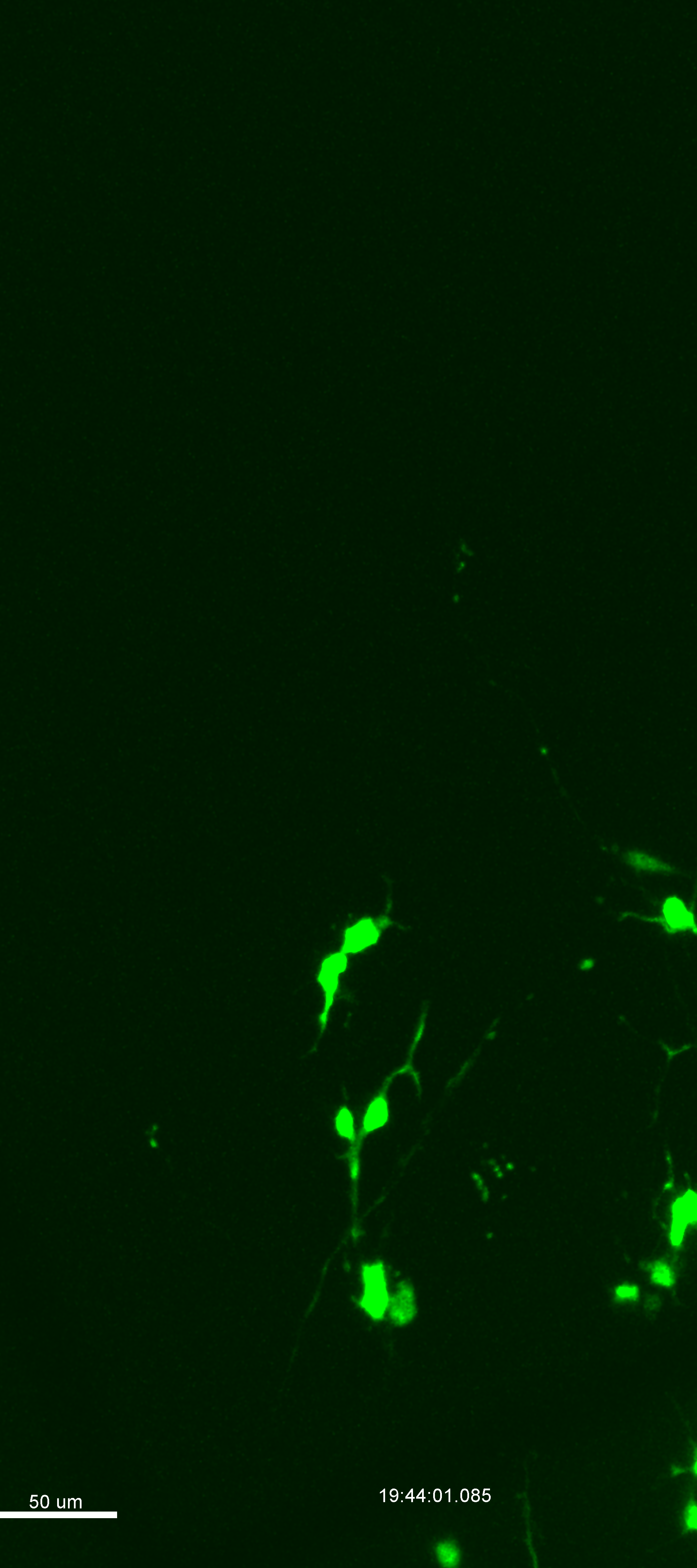

Supplement: Supplementary file 12 — Source data Fig. 4 [file 44318_2025_624_MOESM12_ESM.zip › Figure 4/4F/28.0h.tif]

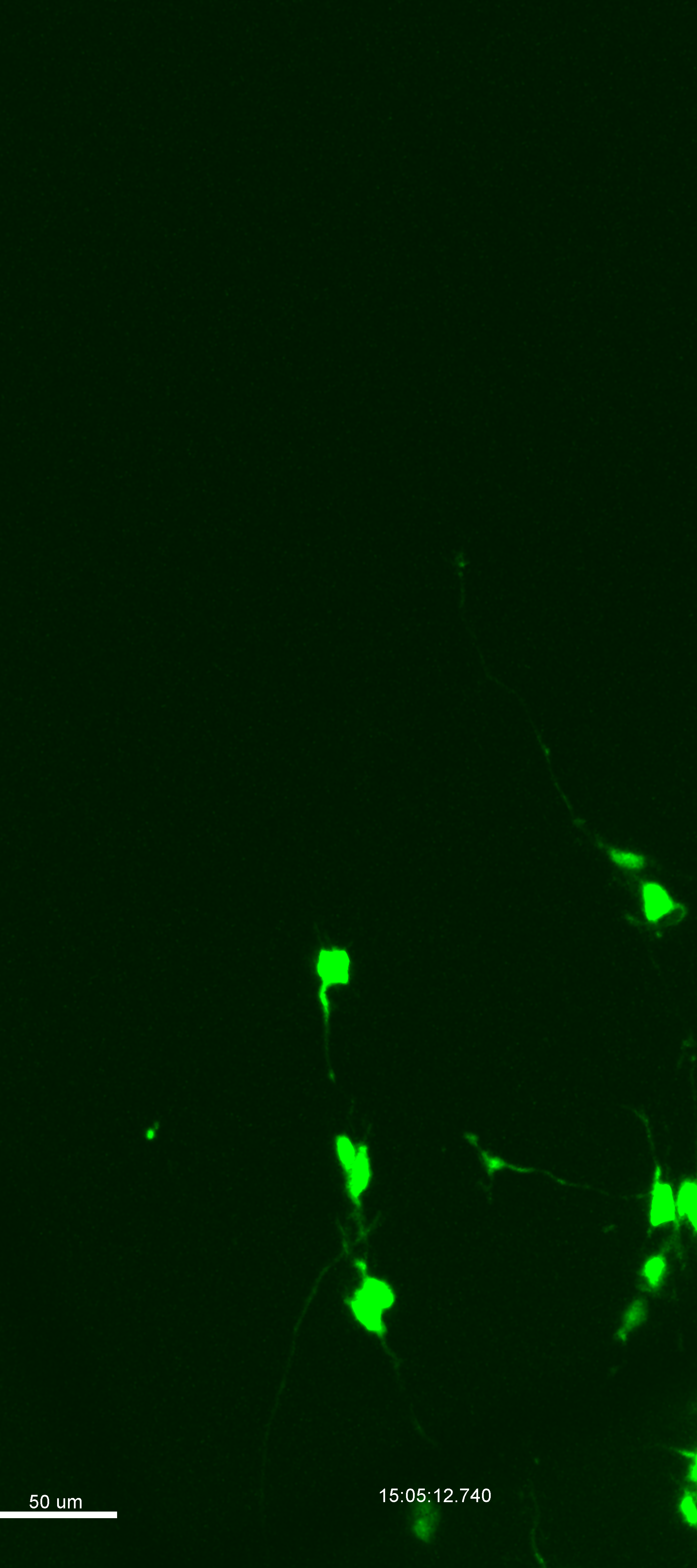

Supplement: Supplementary file 12 — Source data Fig. 4 [file 44318_2025_624_MOESM12_ESM.zip › Figure 4/4F/24.0h.tif]

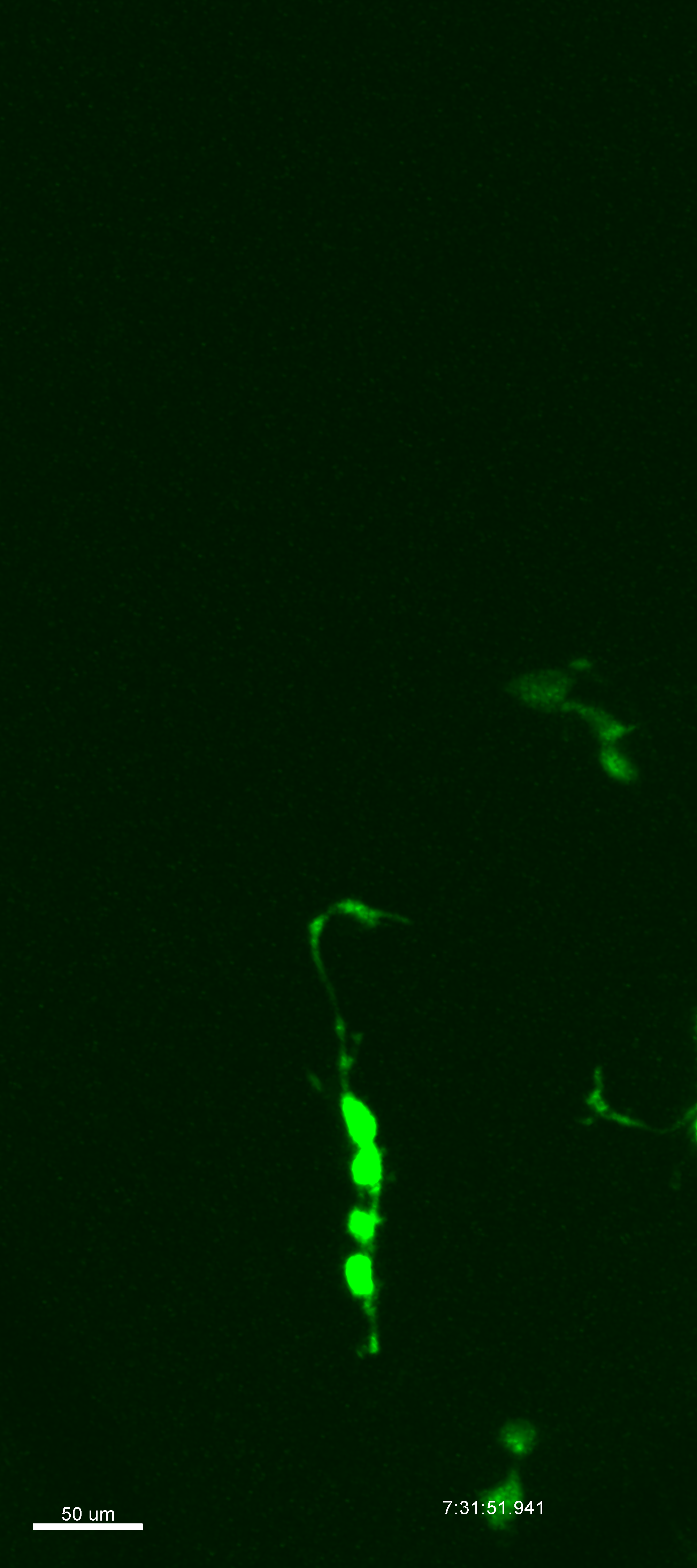

Supplement: Supplementary file 12 — Source data Fig. 4 [file 44318_2025_624_MOESM12_ESM.zip › Figure 4/4F/6.5h.tif]

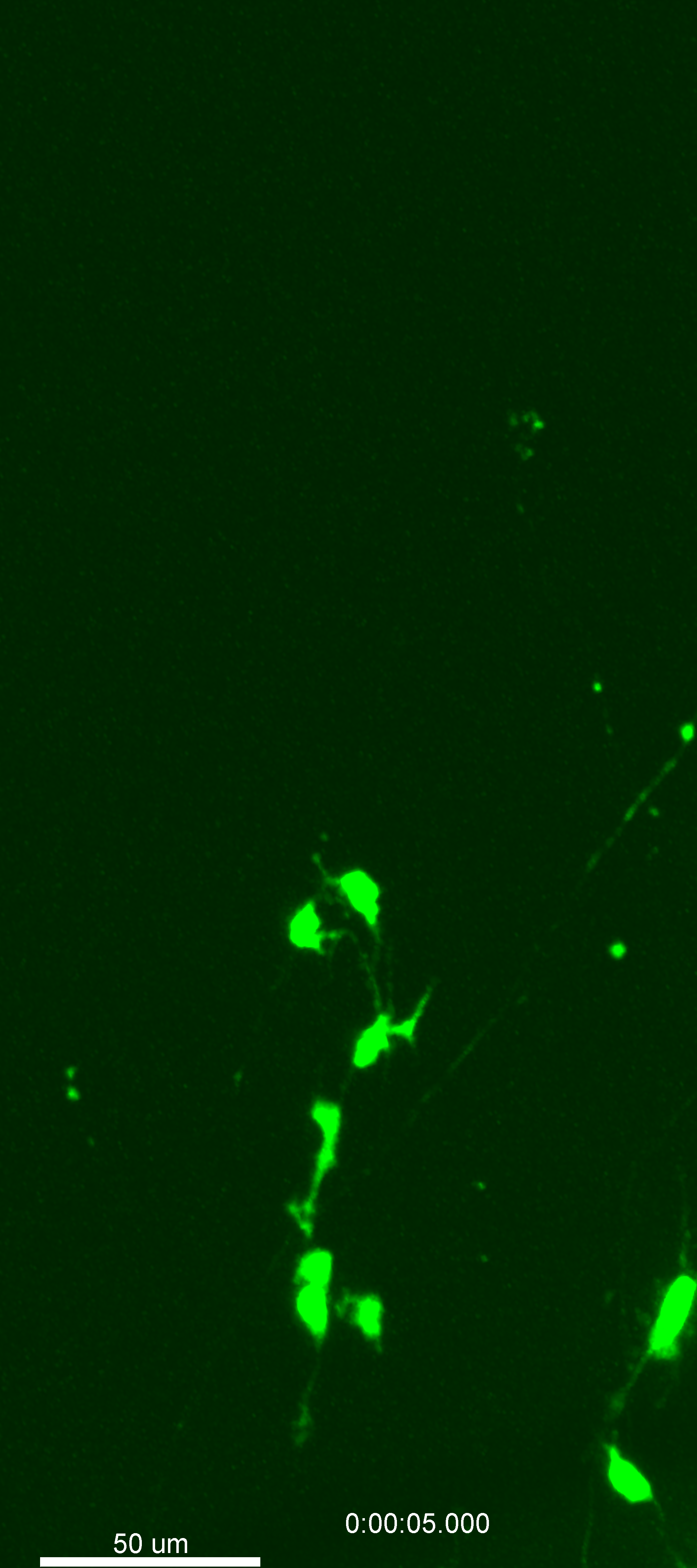

Supplement: Supplementary file 12 — Source data Fig. 4 [file 44318_2025_624_MOESM12_ESM.zip › Figure 4/4F/32.5h.tif]

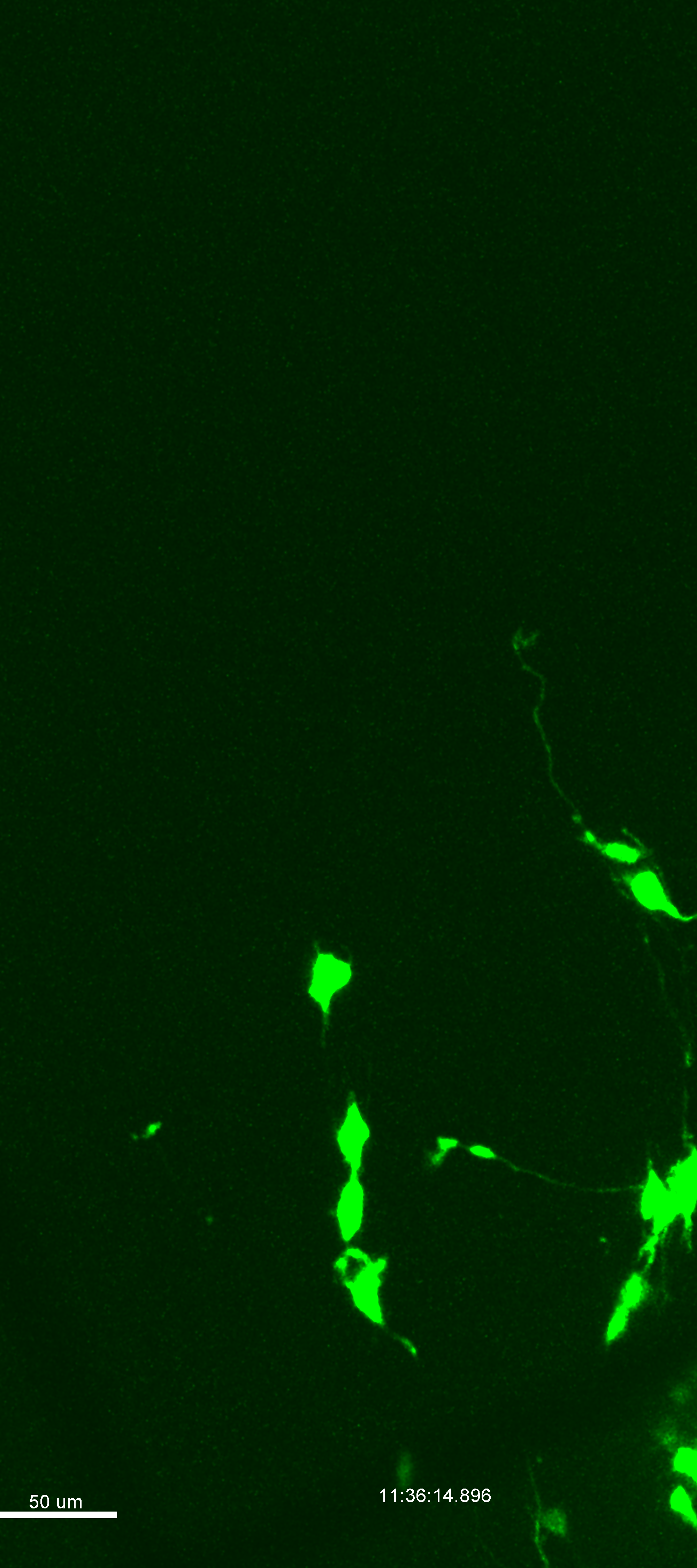

Supplement: Supplementary file 12 — Source data Fig. 4 [file 44318_2025_624_MOESM12_ESM.zip › Figure 4/4F/21.0h.tif]

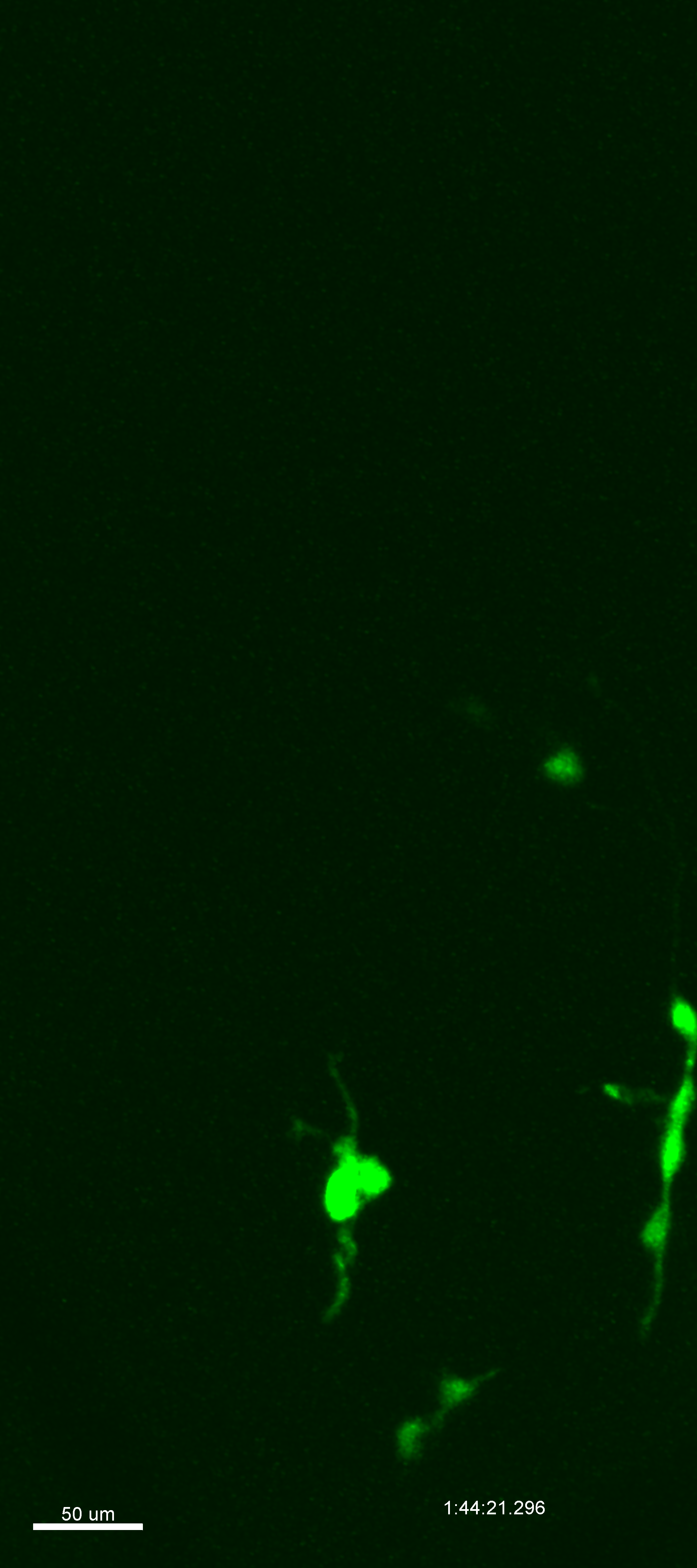

Supplement: Supplementary file 12 — Source data Fig. 4 [file 44318_2025_624_MOESM12_ESM.zip › Figure 4/4F/1.5h.tif]

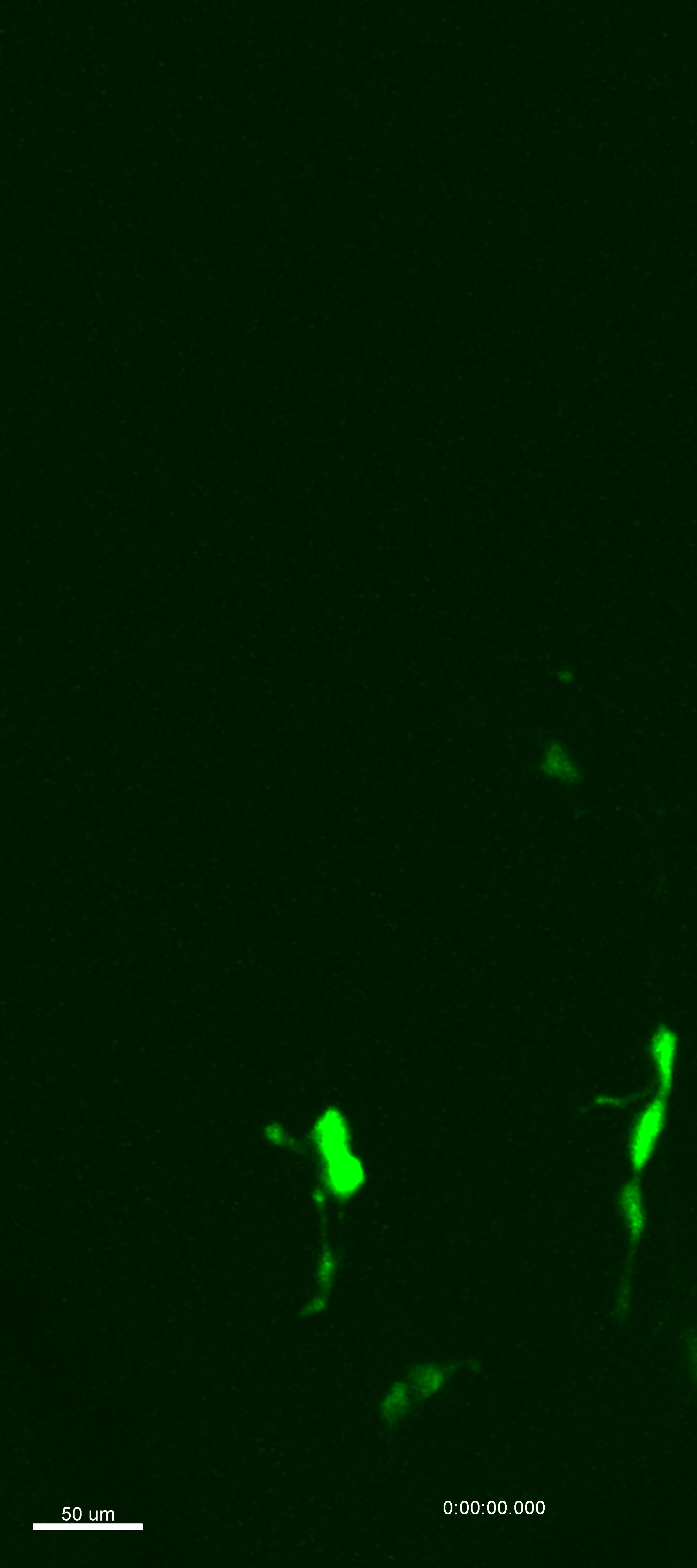

Supplement: Supplementary file 12 — Source data Fig. 4 [file 44318_2025_624_MOESM12_ESM.zip › Figure 4/4F/0h.tif]

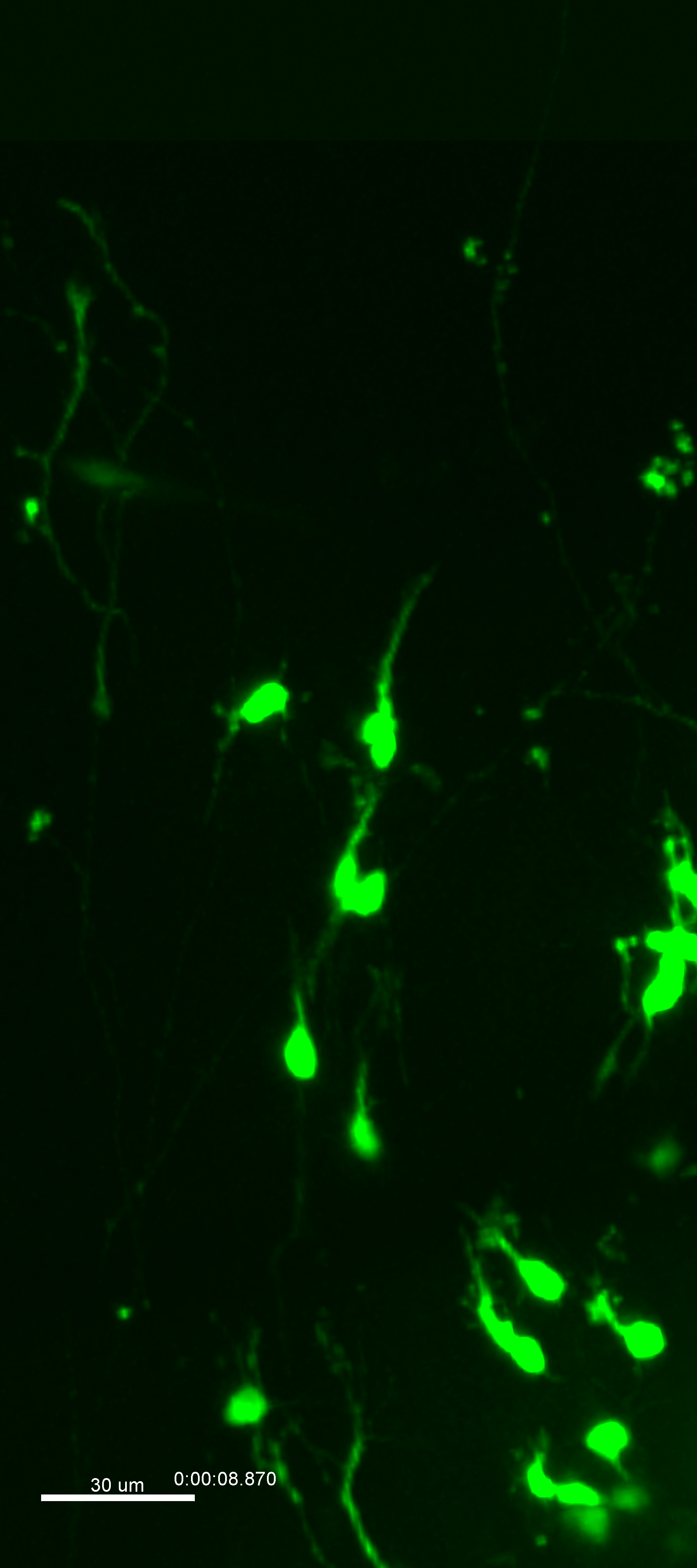

Supplement: Supplementary file 12 — Source data Fig. 4 [file 44318_2025_624_MOESM12_ESM.zip › Figure 4/4F/58.0h.tif]

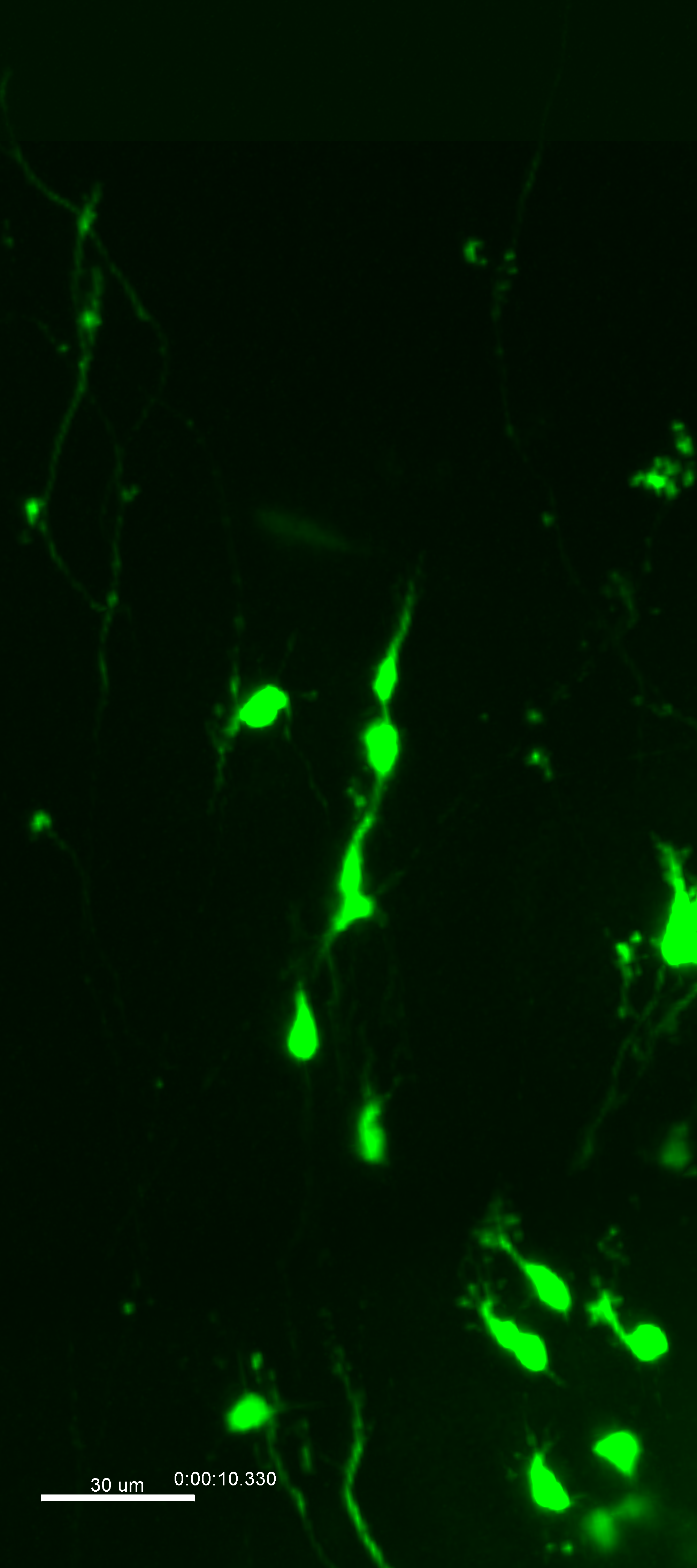

Supplement: Supplementary file 12 — Source data Fig. 4 [file 44318_2025_624_MOESM12_ESM.zip › Figure 4/4F/60.0h.tif]
