# Supplementary material for: Direct and indirect neurogenesis from radial glial progenitor cell clones in the mouse neocortex
Source: EMBO J. 2025 Nov 20;45(1):182–209. doi: 10.1038/s44318-025-00624-9 (PMC12759082; doi:10.1038/s44318-025-00624-9)
Supplement: Supplementary file 14 — Expanded View Figures [file 44318_2025_624_MOESM14_ESM.pdf]

Expanded View Figures

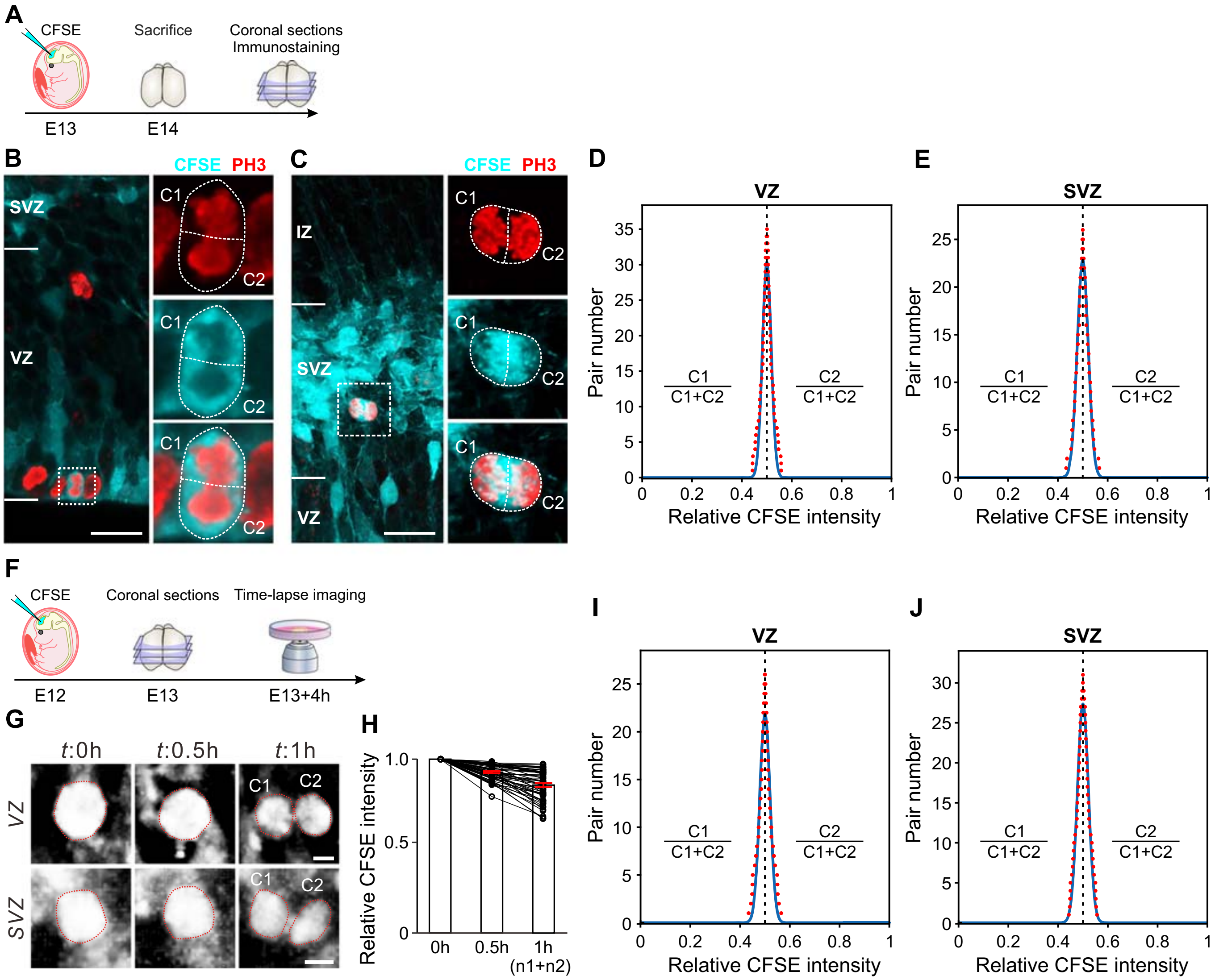

Figure EV1. CFSE fluorescence intensities of progenitors were halved in daughter pairs in the embryonic neocortex.

(**A**) Experimental paradigm of labeling dividing progenitors with CFSE. (**B**) Images of a CFSE/PH3-positive dividing progenitor cell in the VZ of E14 neocortex. C1: cell 1, C2: cell 2. Scale bar: 20 µm. (**C**) Image of a CFSE/PH3-positive dividing progenitor in the SVZ of E14 neocortex. C1: cell 1, C2: cell 2. Scale bar: 20 µm. (**D, E**) Quantification of relative CFSE fluorescence intensities for sister pairs in the VZ (D) and SVZ (E), fitted with Gaussian curves. (**F**) Experimental paradigm of time-lapse imaging of CFSE progenitors. (**G**) Time-lapse imaging of CFSE-labeled dividing progenitors in the VZ and SVZ. C1 and C2 represent daughter pairs generated by an RGP. Scale bar: 5 µm. (**H**) Quantification of relative CFSE fluorescence intensities of dividing progenitors and sister pairs, showing a decrease in fluorescence intensities over the course of imaging ($n = 57$). Data are shown as mean ± SEM. (**I, J**) Quantification of relative CFSE fluorescence intensities for sister pairs in the VZ (I) and SVZ (J) in time-lapse experiments, fitted with Gaussian curves.

 © The Author(s)

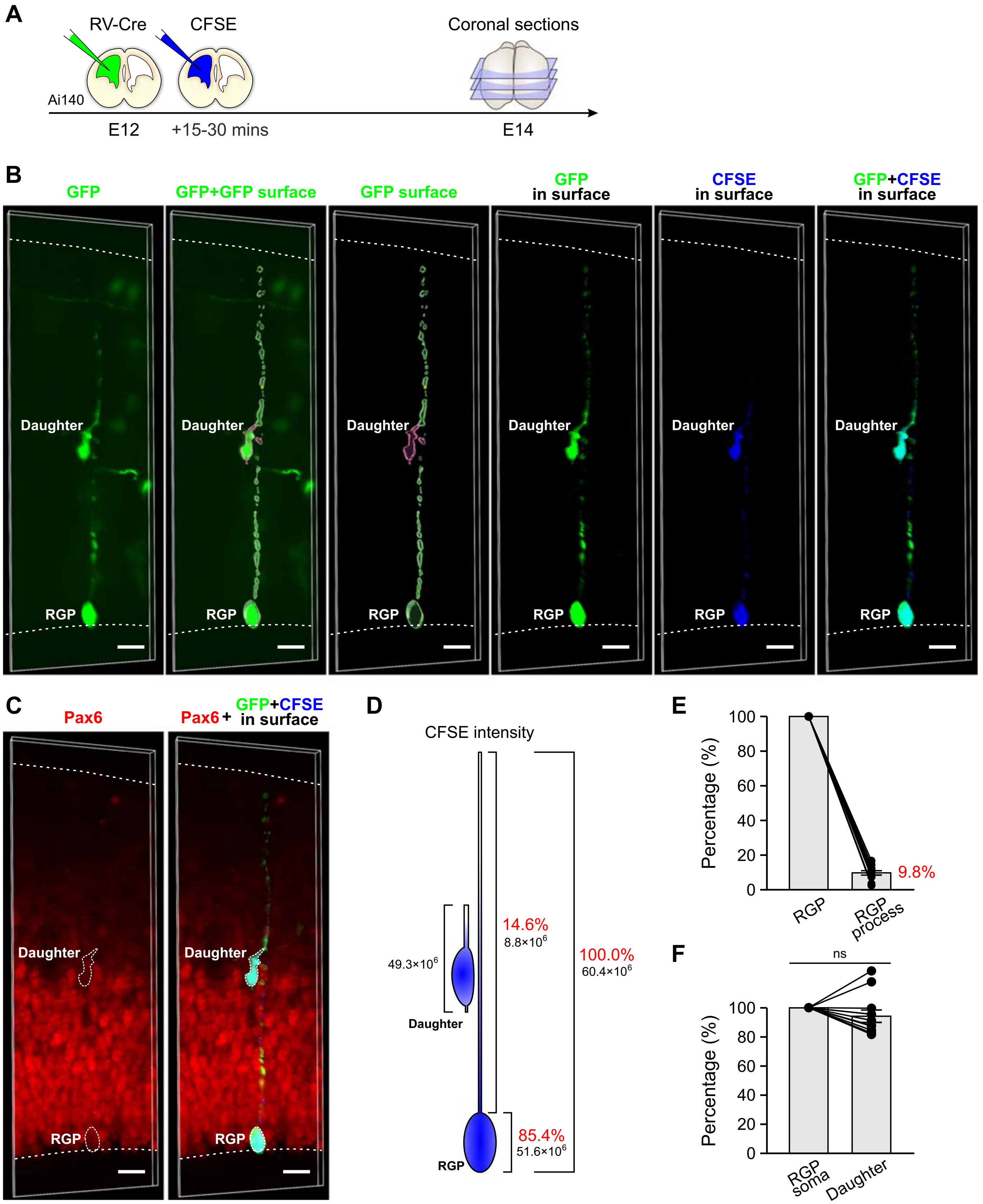
A
RV-Cre
CFSE
Coronal sections
Ai140
E12
+15-30 mins
E14
B
GFP
GFP+GFP surface
GFP surface
GFP in surface
CFSE in surface
GFP+CFSE in surface
Daughter
RGP
C
Pax6
Pax6 + GFP+CFSE in surface
D
CFSE intensity
49.3×10^6
Daughter
14.6%
8.8×10^6
100.0%
60.4×10^6
85.4%
51.6×10^6
RGP
E
Percentage (%)
9.8%
RGP
RGP process
F
ns
RGP soma
Daughter

Figure EV2. Sparse distribution of CFSE fluorescence signals in RGP processes.

(**A**) Experimental paradigm for labeling RGPs and their processes using Rv-FlashTag. (**B**) Confocal images of a lineage, depicting the soma and process of an RGP and its first-generation daughter cell, both labeled with RV-cre and CFSE. The cells were outlined using Surface function in Imaris software. Scale bar: 15 µm. (**C**) Immunohistochemical staining for Pax6 in lineage. Scale bar: 15 µm. (**D**) CFSE fluorescence intensity measurements for RGP soma, RGP process and daughter cell. Black numbers indicate CFSE fluorescence intensity and red numbers indicate the percentages of CFSE fluorescence in RGP soma, process, and the entire sample. (**E**) Proportions of CFSE fluorescence intensities in RGP processes ($n = 57$). Data are shown as mean ± SEM. (**F**) Relative fluorescence intensity comparison between RGP somas and daughter cells ($n = 57$). Data are shown as mean ± SEM. $P = 0.2783$ (paired Student's *t* test followed by Wilcoxon matched-pairs signed rank test).

 © The Author(s)

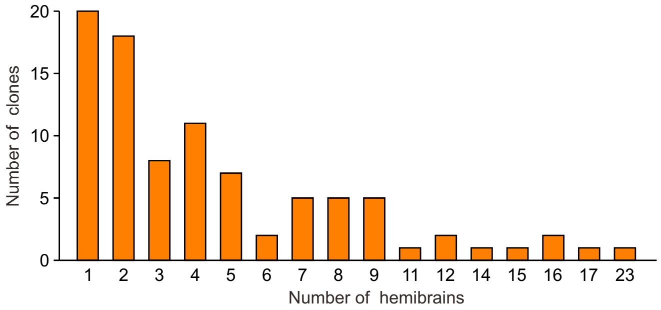

Figure EV3. Clones were sparsely labeled in the neocortex by Rv-FlashTag.

Quantification of the number of Rv-FlashTag labeled clones in individual hemibrains.

© The Author(s)

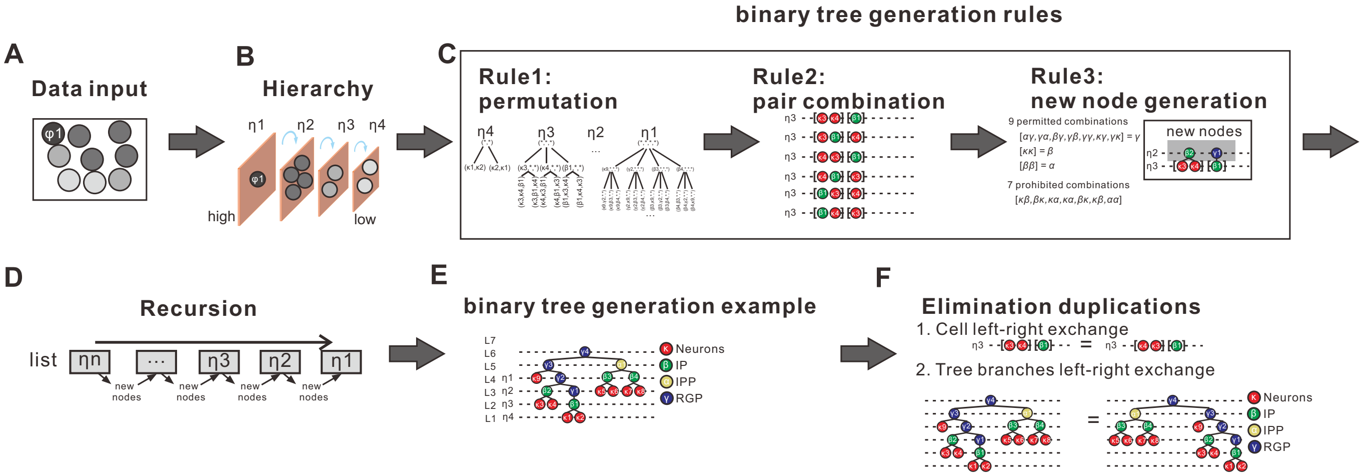

Figure EV4. Automated lineage tree reconstruction.

(**A**) Input data of CFSE fluorescence intensity values of lineages. (**B**) Hierarchical organization of neurons based on varying CFSE intensities. (**C**) Three rules of binary tree generation: permutation, pair combination, and new node generation. (**D**) Recursive application of the three rules from the lowest to the highest CFSE intensity hierarchy. (**E**) Sample binary tree generation. (**F**) Removal of duplicated lineages.

 © The Author(s)

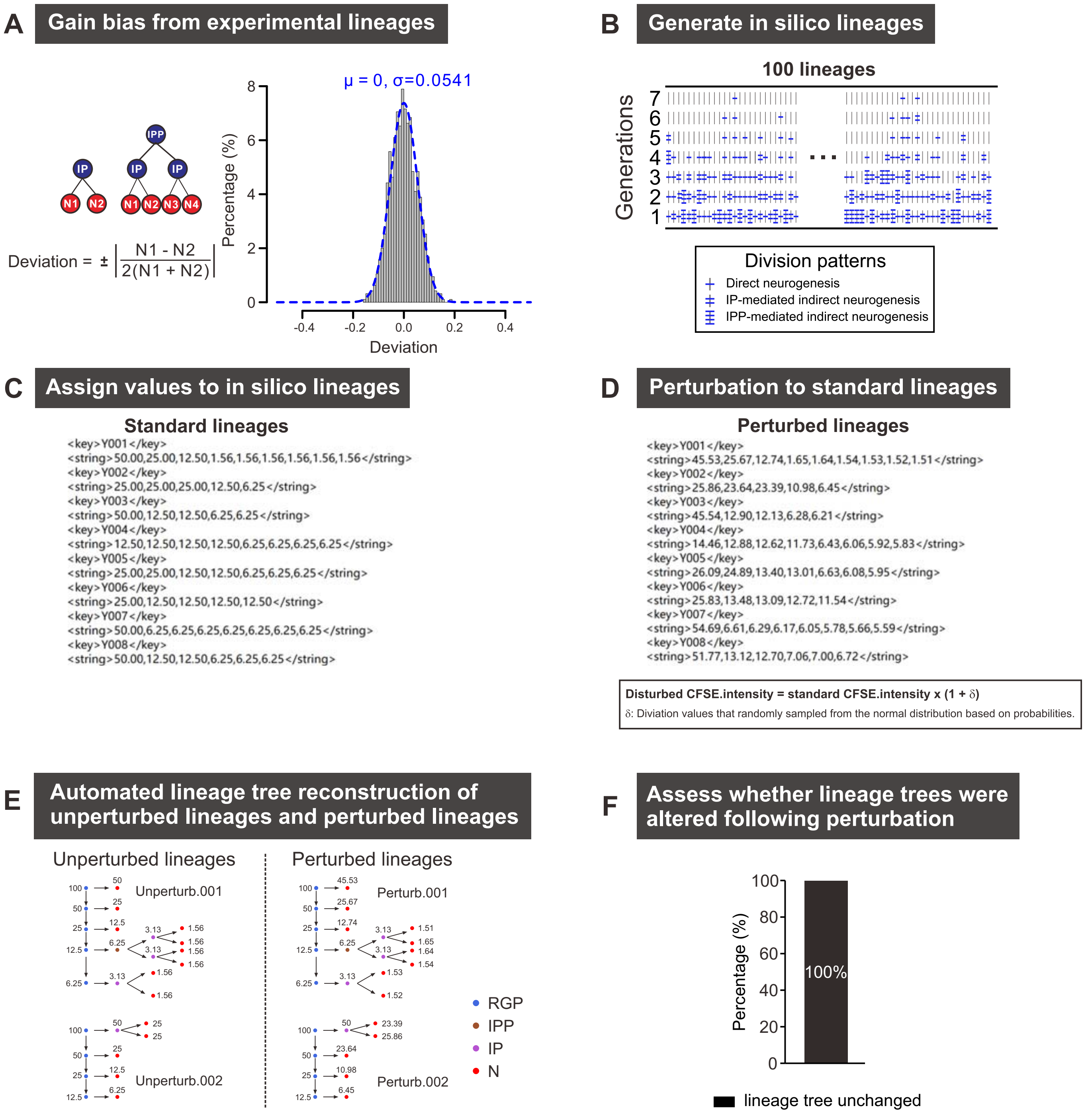

Figure EV5. Validation of CFSE distribution deviations on lineage reconstruction accuracy.

(**A**) CFSE fluorescence deviations between daughter cells follow a normal distribution with mean (μ) = 0, standard deviation (σ) = 0.0541, and 99% confidence interval = ±0.139. The deviation is calculated as ±|(N1 - N2)/2(N1 + N2)|, where N1 and N2 represent CFSE intensities in daughter cells. (**B**) 100 in silico lineage trees with different division patterns. Generations 1-7 are shown vertically. (**C**) Control dataset: unperturbed CFSE intensity values for representative in lineage trees, formatted in XML with unique identifiers. Initial CFSE fluorescence intensity is normalized to 100 for the founding cell. (**D**) Test dataset: lineage data after applying experimentally-derived fluorescence perturbations. Perturbed CFSE intensity = standard intensity × (1 + δ), where δ values are randomly sampled from the experimentally observed normal distribution. (**E**) Automated lineage reconstruction comparison demonstrates identical tree topologies for unperturbed (left) versus perturbed (right) datasets. (**F**) Quantitative assessment of reconstruction accuracy across all 100 simulated lineage trees. Bar graph shows 100% of lineage trees remained unchanged following perturbation.

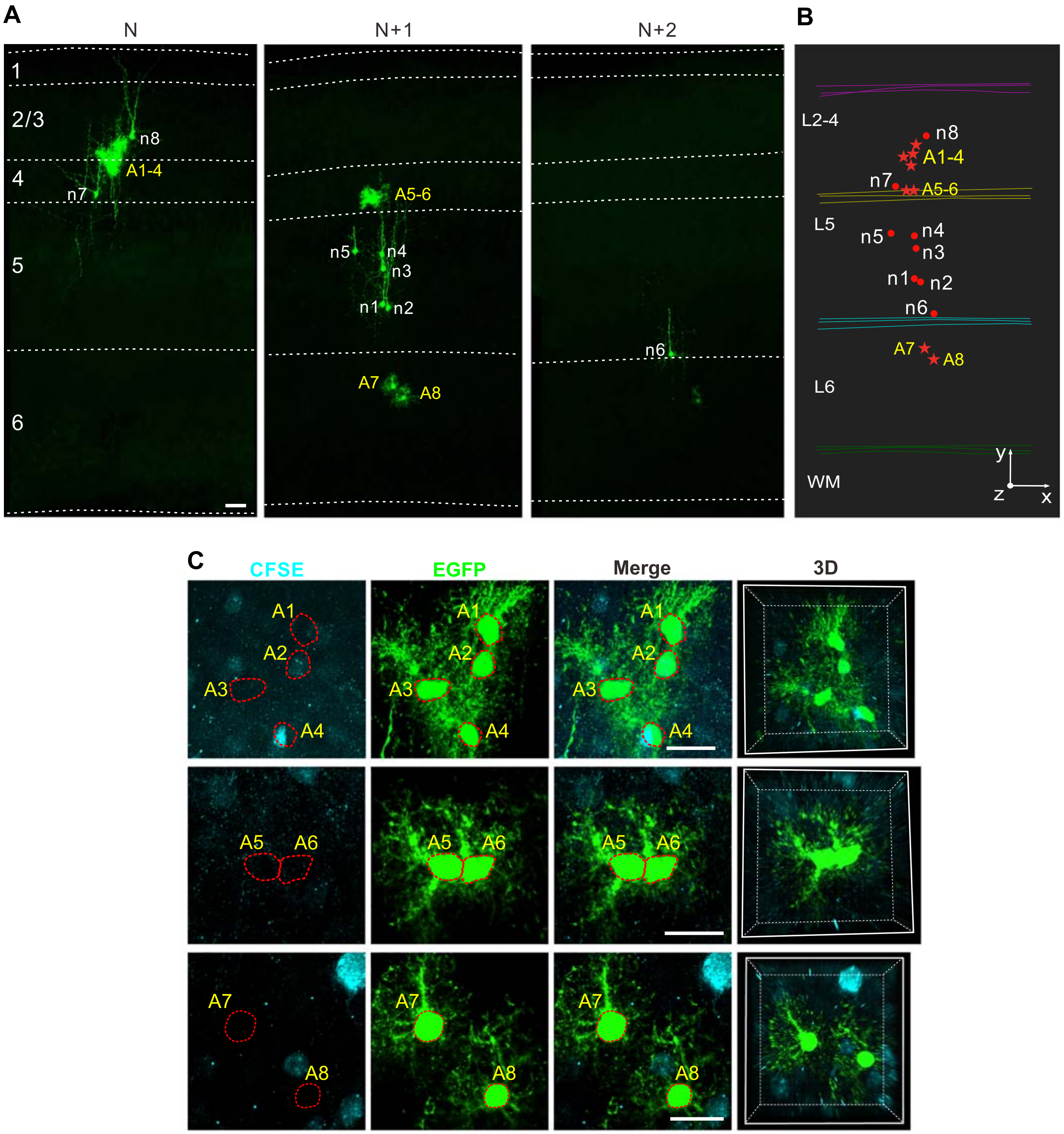

Figure EV6. Rv-FlashTag labeled glial lineages.

(**A**) Confocal images of an Rv-FlashTag labeled clone that contains eight sister neurons (n1-n8) and eight glial cells (A1-A8). Scale bar: 50 μm. (**B**) 3D reconstruction of the clone in (**A**). Red dots represent neurons and red stars represent astrocytes. WM white matter. (**C**) High-magnification images of glial cells in this clone. Except for A2, other glial cells were unlabeled with CFSE. Scale bar: 20 μm.

 © The Author(s)

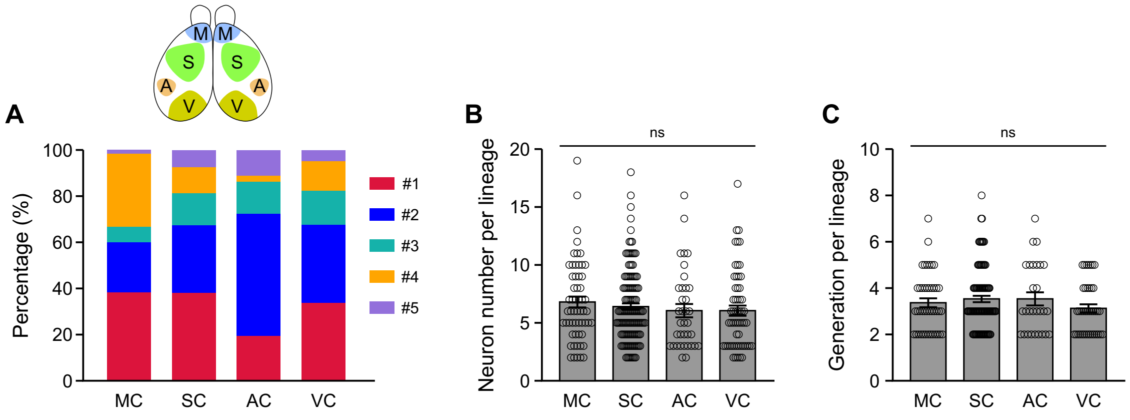

Figure EV7. Characteristics of lineages across cortical regions.

(**A**) Proportions of lineage clusters in motor cortex (MC), somatosensory cortex (SC), auditory cortex (AC), and visual cortex (VC). The schematic diagram above illustrates the relative positions of four cortical regions in the brain. (**B**) Clone sizes in MC ($n = 56$), SC ($n = 130$), AC ($n = 36$) and VC ($n = 58$). Data are shown as mean ± SEM. $P = 0.4477$ (Kruskal–Wallis one-way ANOVA). (**C**) Number of generations per lineage in the MC ($n = 43$), SC ($n = 97$), AC ($n = 28$) and VC ($n = 40$). Data are shown as mean ± SEM. $P = 0.4694$ (Kruskal–Wallis one-way ANOVA).

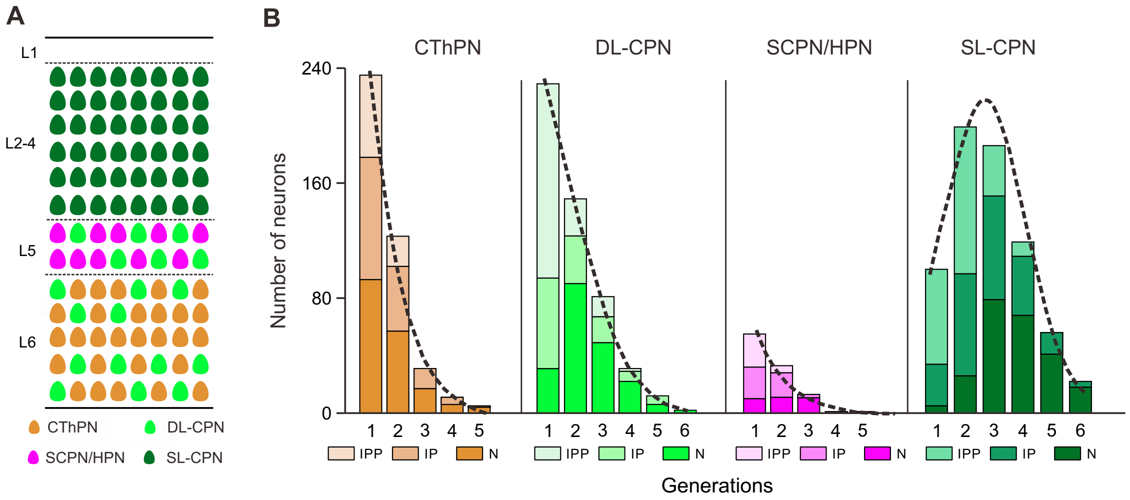

Figure EV8. CThPN, DL-CPN, SCPN/HPN and SL-CPN neuronal output across generations.

(**A**) Layer distribution of different types of pyramidal neurons. (**B**) CThPN, DL-CPN, SCPN/HPN and SL-CPN numbers generated in G1 to G5/6 in all lineages. The fitting curves of CThPN, DL-CPN and SCPN/HPN are the exponential decay. The fitting curve of SL-CPN is a Gaussian distribution.

 © The Author(s)
